# Mechanistic insights into cobalt(ii/iii)-catalyzed C–H oxidation: a combined theoretical and experimental study†
†Electronic supplementary information (ESI) available: Complete Gaussian 09 reference, computational data, alternative methanol-assisted proton transfer pathways, the details of control experiments, EPR spectrum, characterization of products, NMR and mass spectra. See DOI: 10.1039/c5sc01807b


**DOI:** 10.1039/c5sc01807b

**Published:** 2015-09-09

**Authors:** Xiao-Kang Guo, Lin-Bao Zhang, Donghui Wei, Jun-Long Niu

**Affiliations:** a The College of Chemistry and Molecular Engineering , Zhengzhou University , Zhengzhou , Henan Province 450001 , P. R. China . Email: donghuiwei@zzu.edu.cn ; Email: niujunlong@zzu.edu.cn

## Abstract

Cobalt-mediated C–H functionalization has been the subject of extensive interest in synthetic chemistry, but the mechanisms of many of these reactions (such as the cobalt-catalyzed C–H oxidation) are poorly understood. In this paper, possible mechanisms including single electron transfer (SET) and the concerted metalation–deprotonation (CMD) pathways of the Co^II^/Co^III^-catalyzed alkoxylation of C(sp^2^)–H bonds have been investigated for the first time using the DFT method. Co^II^(OAc)_2_ has been employed as an efficient catalyst in our previous experimental study, but the calculated results unexpectedly indicated that the intermolecular SET pathway with Co^III^ as the actual catalyst might be the most favorable pathway. To support this theoretical prediction, we have explored a series of Cp*Co^III^(CO)I_2_ catalyzed C(sp^2^)–H bond alkoxylations, extending the application of cobalt-catalyzed functionalization of C–H bonds. Furthermore, kinetic isotope effect (KIE) data, electron paramagnetic resonance (EPR) data, and TEMPO inhibition experiments also support the SET mechanism in both the Co-catalyzed alkoxylation reactions. Thus, this work should support an understanding of the possible mechanisms of the Co^II^/Co^III^-catalyzed C(sp^2^)–H functionalization, and also provide an example of the rational design of novel catalytic reactions guided by theoretical calculations.

## Abstract 2

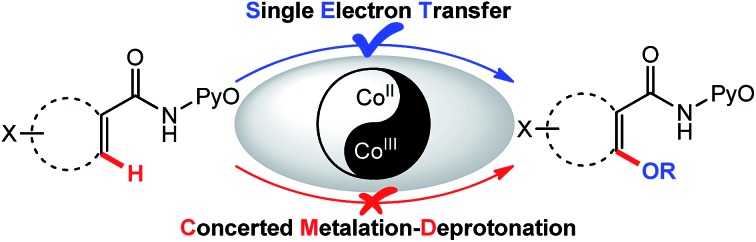
The detailed mechanism of the cobalt(ii/iii)-catalyzed C–H oxidation has been investigated by using both theoretical and experimental methods.

## Introduction

Transition-metal-catalyzed C–H bond functionalization has attracted widespread attention due to its high efficiency and atom economy.^1^ However, most of these transformations have been accomplished with the aid of transition metals such as palladium,1l,^2^ rhodium,^3^ and ruthenium.^4^ Recent improvements in sustainable catalysis have focused on the development of cheaper and naturally abundant first-row transition-metal alternatives with comparable catalytic efficacies.^5^ Cobalt as a catalyst for C–H bond functionalization has been greatly developed with significant progress being achieved.^6^ For example, electron-rich Co^I^ catalysts have been used in C–H activation/coupling reactions with alkynes, olefins, imines, alkyl/aryl halides, and phenol derivatives. Subsequently, high-valent Cp*Co^III^ complexes such as Cp*Co(CO)I_2_, [Cp*Co^III^(arene)](PF_6_)_2_, and [Cp*Co(C_6_H_6_)][B(C_6_F_5_)_4_]_2_ were also discovered by several groups as effective catalysts with which to activate C–H bonds for addition to unsaturated compounds (imines, enones, and aldehydes) and amidation, cyanation, allylation, and halogenations.^7^ Additionally, Daugulis reported a new method for Co^II^-catalyzed C–H bond alkenylation, in which aminoquinoline is utilized as a directing group.^8^

**Scheme 1:**
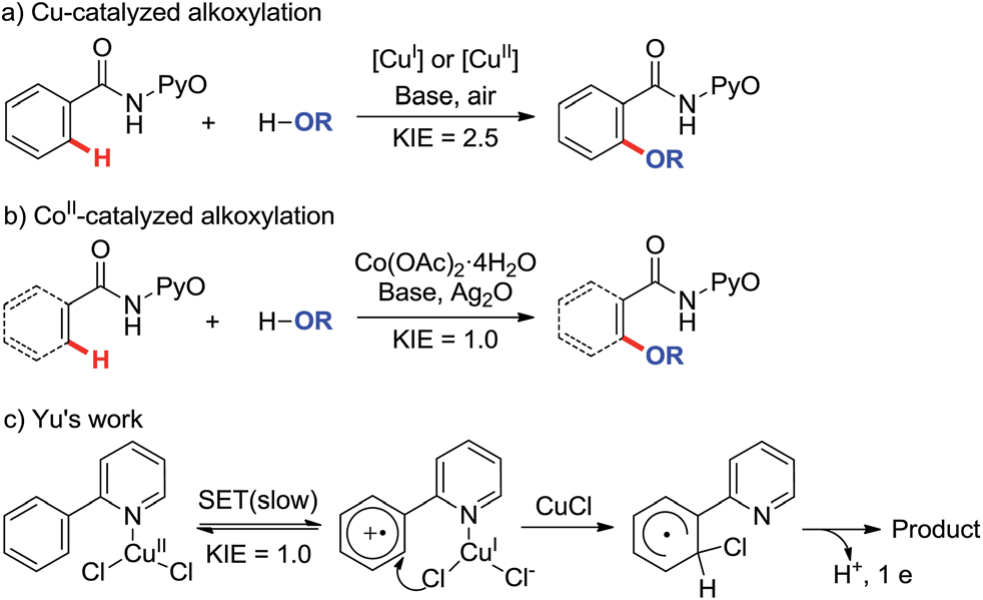
Copper- and cobalt-catalyzed C(sp^2^)–H functionalization.

The formation of C–O bonds is more difficult than C–C bond generation by C–H activation, especially when alcohols are used as alkoxylation reagents^9^ and alkoxyl metal intermediates are prone to undergo β-hydride elimination to form the corresponding aldehydes, ketones, or carboxylic acids.^10^ So far, most of the cases that involve transition-metal-catalyzed C–H activation are focused on palladium catalysts^11^ but a few reports on copper catalyzed systems have also been disclosed.^12^ A Cu-catalyzed alkoxylation of arenes under basic and aerobic conditions (Scheme 1a) was also developed in this laboratory.^13^ Recently, we reported the first Co^II^-catalyzed alkoxylation of C(sp^2^)–H bonds under basic and oxidizing conditions (Scheme 1b).^14^ Due to the similarity between cobalt- and copper-catalyzed C–H functionalization, it might be expected that these reactions share a similar reaction mechanism, particularly in the early steps.

For the Cu catalyzed reaction shown in Scheme 1a, a high kinetic isotope effect (KIE = 2.5) is observed, indicating that the C–H bond cleavage is the rate-determining step. It is generally accepted that the C–H bond is activated *via* a concerted metalation–deprotonation (CMD) mechanism.^15^ The similarity between the starting materials and reaction conditions depicted in Scheme 1a and b may imply a similar reaction mechanism for the two reactions but, unexpectedly, a preliminary mechanistic study of the Co^II^ catalytic reaction depicted in Scheme 1b revealed that KIE ≈ 1, indicating that the C–H bond cleavage does not proceed by the same mechanism as the Cu catalyzed reaction. This phenomenon is similar to the Cu^II^-catalyzed example reported by Yu's group (Scheme 1c),^16^ in which an intramolecular single electron transfer (SET) route, not the CMD route, was invoked. The similarity of the KIE results implies that our Co^II^-catalyzed system may also follow the SET mechanism. Stahl *et al.* observed a switch between SET-based and CMD-based pathways upon changing from acidic to basic reaction conditions in Cu^II^-mediated aerobic C–H oxidation, in which a KIE is observed after the system is made alkaline.^17^ Consequently, a comparative study on both the SET-based and CMD-based routes is necessary to determine the detailed reaction mechanism of the Co^II^-catalyzed alkoxylation.

**Scheme 2:**
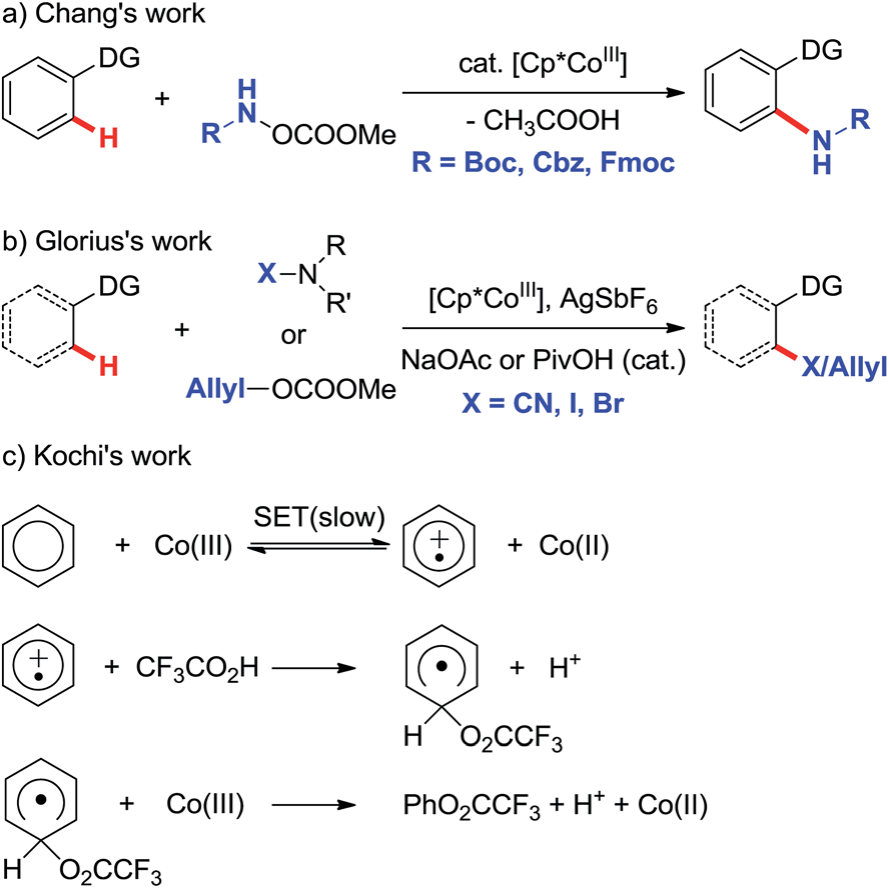
Co^III^-catalyzed C(sp^2^)–H functionalization.

Despite the great progress that has been made in understanding cobalt catalysis, the detailed reaction mechanisms for these reactions remain poorly understood. In particular, Co^II^ in the reaction system can be oxidized to Co^III^ by the excess oxidant additive (Scheme 1b), and it is unclear whether the exact oxidation state of the catalytic Co species is Co^II^ or Co^III^. In recent months, Co^III^-catalyzed C(sp^2^)–H functionalization reactions have been reported (Scheme 2a and b),7e,h but the mechanisms of these reactions continue to be hotly debated. On the one hand, recent literature studies report that Co^III^ compounds are capable of catalyzing many chelate-directed C(sp^2^)–H functionalization reactions.7c,e,f,h,^18^ None of these works indicate the existence of a radical intermediate, all of them supporting organometallic mechanisms, even when the C–H activation is shown by KIE assessment to be not always rate-limiting. On the other hand, the oxidation of a π system by cobalt complexes is a well-known process which involves the intermediacy of carbon-centered radicals.^19^ For example, Kochi employed the intermolecular SET concept to elucidate the Co^III^(TFA)_3_-mediated oxidation of aryl C–H bonds (Scheme 2c)^20^ and concluded that an aromatic cation-radical intermediate is formed after an arene electron is transferred to Co^III^. Likewise, it is reasonable to speculate that the intermolecular SET mechanism may be appropriate in the Co^III^-catalyzed C(sp^2^)–H functionalization described above.

No computational study on the mechanism of Co^II^/Co^III^-catalyzed C(sp^2^)–H alkoxylation appears to have been performed, and experiments on the Co^III^-catalyzed C(sp^2^)–H alkoxylation of arenes and olefins have not been reported. Several questions on the possible mechanisms of this kind of reaction need however to be resolved: (a) which mechanism (CMD or SET) is favored when Co^II^ is the pre-catalyst? (b) In which state (Co^II^ or Co^III^) is the actual catalyst in high-valency Co-catalyzed C(sp^2^)–H alkoxylation? (c) Can the Co^III^ compound catalyze the C(sp^2^)–H alkoxylation? If so, which mechanism (CMD or SET) is favorable?

All of these questions led us to study the detailed mechanisms of Co-catalyzed C(sp^2^)–H alkoxylation not only in theory but also experimentally. In the present study, we first conducted a theoretical investigation of the detailed mechanism of Co^II^- and Co^III^-catalyzed C(sp^2^)–H alkoxylation. This predicted that the Co^III^ compound might also catalyze the C–H alkoxylation. Subsequently, experimental results of the Cp*Co^III^(CO)I_2_-catalyzed C(sp^2^)–H alkoxylation were found to confirm and support the theoretical prediction.

For practical assessment of the energetic viability of the possible routes, the Co^II^(OAc)_2_·4H_2_O catalyzed C(sp^2^)–H alkoxylation reaction between methanol and (*E*)-2-(2-methylbut-2-enamido)pyridine-1-oxide (denoted as **R**) was chosen for the theoretical investigation. Ag_2_O acts as the oxidant and NaOPiv as the base. It should be noted that Ag_2_O can react with methanol to generate AgOMe by the equation:Ag_2_O + 2MeOH = 2AgOMe + H_2_Oand we believe AgOMe may be the actual oxidant in this reaction. This is supported by our observation that AgOTf can also work as the oxidant.^14^ The calculations were performed at the M06-L level of density functional theory (DFT),^21^ incorporating solvation effects *via* the appropriate SMD continuum solvation model.^22^

## Results and discussion

Several possible reaction pathways of Co^II^/Co^III^-catalyzed C(sp^2^)–H alkoxylation have been studied by DFT, and further experimental studies have been performed to support the results predicted by the theoretical study.

### 1. Possible pathways of Co^II^-catalyzed C(sp^2^)–H alkoxylation

**Scheme 3:**
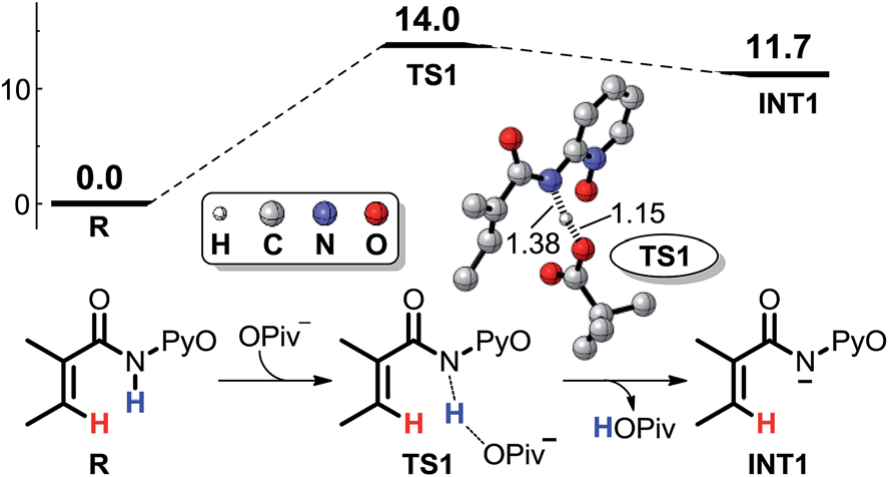
Extraction of proton by the base additive.

**Scheme 4:**
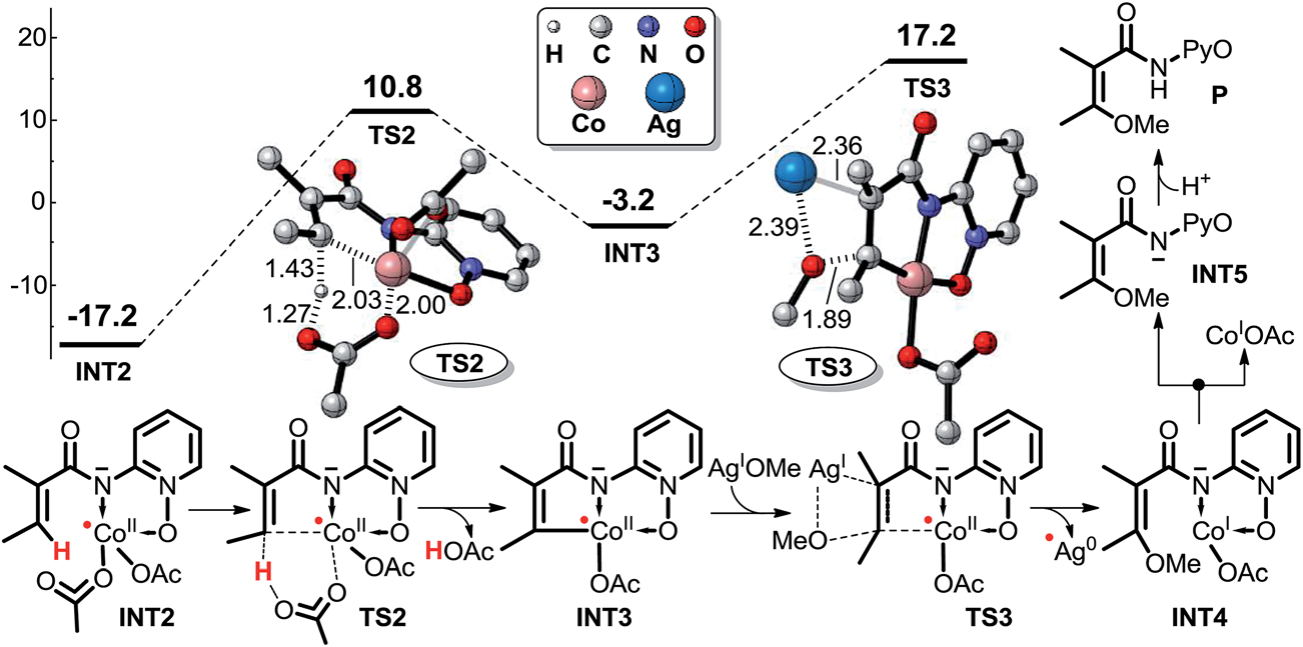
Possible CMD mechanism (pathway 1) of Co^II^-catalyzed C(sp^2^)–H alkoxylation (energy: kcal mol^–1^, distance: Å).

In order to coordinate with Co, the nitrogen in the reactant **R** should initially be deprotonated. According to our calculated results shown in Scheme 3, the base OPiv^–^ in the reaction system can abstract the proton attached to the nitrogen easily *via* transition state **TS1**, whose formation requires only 14.0 kcal mol^–1^. The carboxamide anion intermediate **INT1** can coordinate with Co^II^ forming **INT2**, as shown in Scheme 4. Two kinds of mechanisms, including SET and CMD, to activate the C–H bond are possible. We set the energy of reactant **R** at 0.0 kcal mol^–1^ in the energy profiles, and all the energies of the other minima discussed below are relative to that unless otherwise specified.

#### 1.1. Concerted metalation–deprotonation mechanism for Co^II^-catalyzed C(sp^2^)–H alkoxylation

The CMD mechanism of Co^II^-catalyzed C(sp^2^)–H alkoxylation (pathway 1) consists of two key reaction steps (Scheme 4). The first step proceeds through a six-membered ring transition state **TS2** leading to the formation of intermediate **INT3**, in which the C(sp^2^)–H bond is activated and the proton is transferred to ligand OAc^–^. The second step is OMe group transfer from AgOMe to the carbon atom *via* transition state **TS3** affording intermediate **INT4**. After the dissociation of intermediate **INT4** to **INT5** and Co^I^OAc, the Co^I^ is oxidized to Co^II^ by AgOMe, and the protonation of **INT5** produces the product **P**. The energy barriers for transition states **TS2** and **TS3** in the CMD pathway are 28.0 and 20.4 kcal mol^–1^ (Scheme 4), respectively. Apparently, the C–H activation step associated with **TS2** is rate-determining, which is not consistent with the previous KIE (≈1.0) results and experimental electron spin resonance (ESR) results. Thus, we can conclude that the CMD mechanism will not be the favorable pathway.^14^ In addition, the energy difference between the lowest and the highest stationary points in the energy profile is 34.4 kcal mol^–1^, indicating that this pathway is quite unlikely and it is unnecessary to explore the details after transition state **TS3**.

#### 1.2. Possible single electron transfer pathways for Co^II^-catalyzed C(sp^2^)–H alkoxylation

##### 1.2.1 Intramolecular SET pathways

**Scheme 5:**
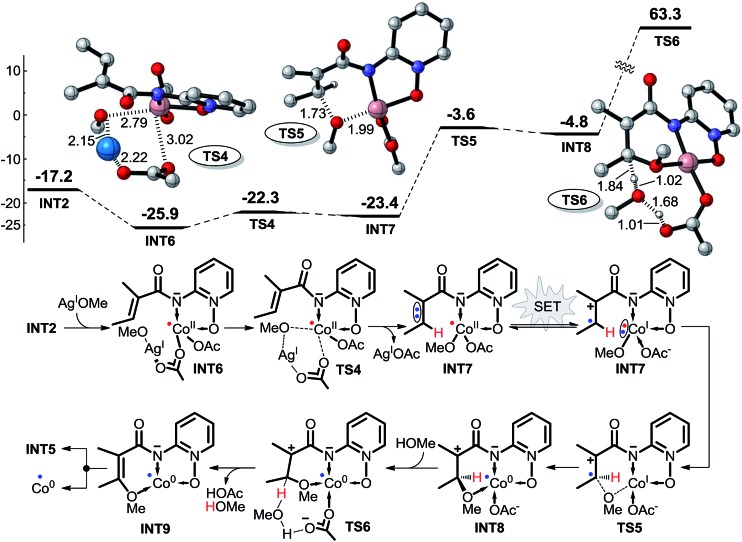
Possible intramolecular SET pathway (pathway 2) of Co^II^-catalyzed C(sp^2^)–H alkoxylation (energy: kcal mol^–1^, distance: Å).

As described above, C–H bond activation may follow the SET mechanisms, and here two possible intramolecular SET pathways (including pathways 2 and 3) were explored. As shown in Scheme 5, pathway 2 consists of four steps: the ligand exchange between Co^II^ and AgOMe *via* transition state **TS4**, the intramolecular single electron transfer of intermediate **INT7**, the OMe group transfer from Co^I^ to the electron-deficient carbon *via* transition state **TS5**, and the solvent MeOH-assisted proton transfer from the olefinic carbon to the ligand OAc^–^*via* transition state **TS6**. The structure of intermediate **INT7** still has only one unpaired electron, a doublet, after the intramolecular SET, and the two structures of intermediate **INT7**, before and after the SET, as in Scheme 5, resemble resonance structures of one another. We assumed that the SET may occur in this reaction pathway, and the details of this SET process are discussed in the next section. With the exception of the SET step, the Gibbs free energy barriers of the first and third steps in pathway 1 are only 3.6 and 19.8 kcal mol^–1^ (Scheme 5), respectively. However, the energy barrier of transition state **TS6** for C–H bond breaking is extremely high (68.1 kcal mol^–1^), suggesting that pathway 2 is not energetically favorable.

**Scheme 6:**
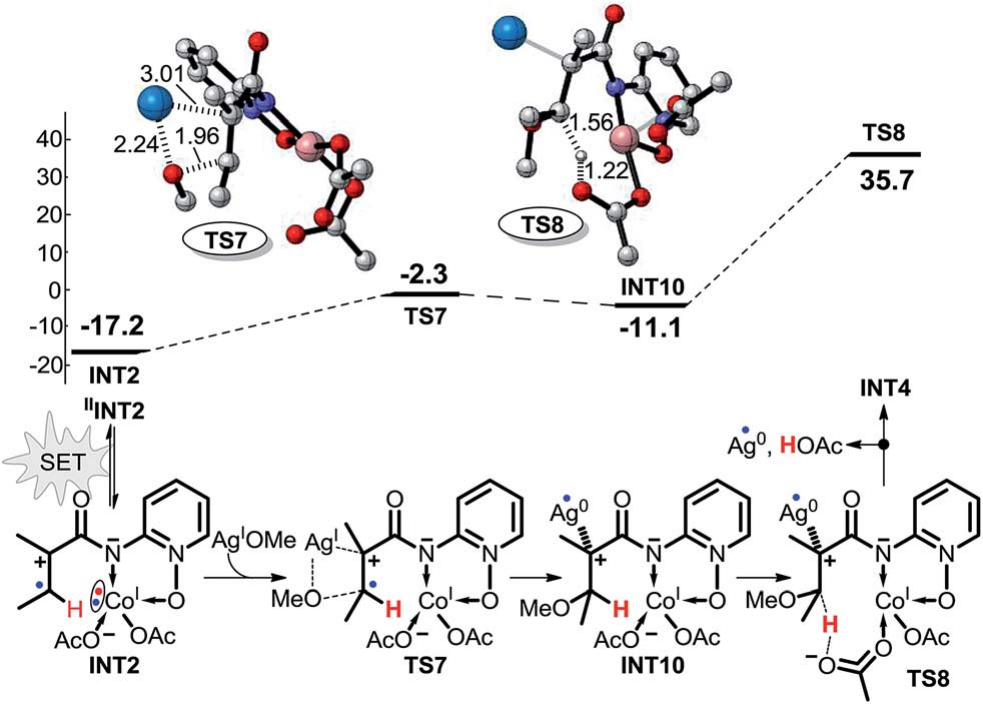
Possible intramolecular SET pathway (pathway 3) of Co^II^-catalyzed C(sp^2^)–H alkoxylation (energy: kcal mol^–1^, distance: Å).

As shown in Scheme 6, pathway 3 consists of three reaction steps. The first is the intramolecular SET step for **INT2**. Here, we also assumed that the SET can occur in this reaction pathway. The OMe group can also be transferred directly from AgOMe to **INT2***via* transition state **TS7**. The last step is a direct proton transfer process *via* transition state **TS8**. The energy barrier of the second step is only 14.9 kcal mol^–1^, but the energy barrier for the proton transfer is as high as 46.8 kcal mol^–1^, indicating that pathway 3 is unlikely to occur under the experimental conditions.

**Fig. 1:**
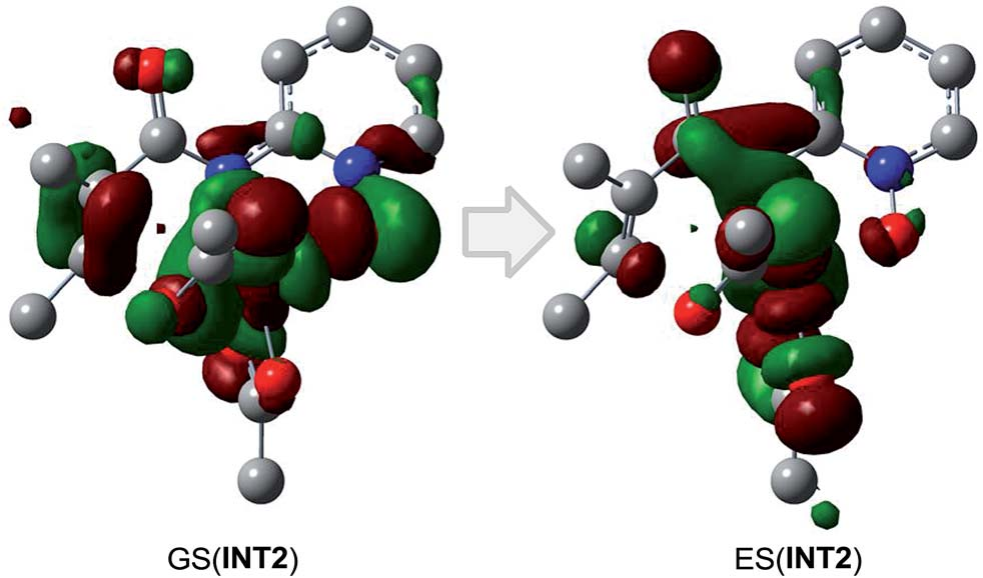
The lowest excited-state doublet derives from excitation of an α-electron from the singly occupied SOMO (GS(**INT2**)) into the nominally unoccupied LUMO (ES(**INT2**), d_*z*^2^_). The resulting excited state is 37.2 kcal mol^–1^ above the ground state, corresponding to a photon energy of 906.95 nm.

**Fig. 2:**
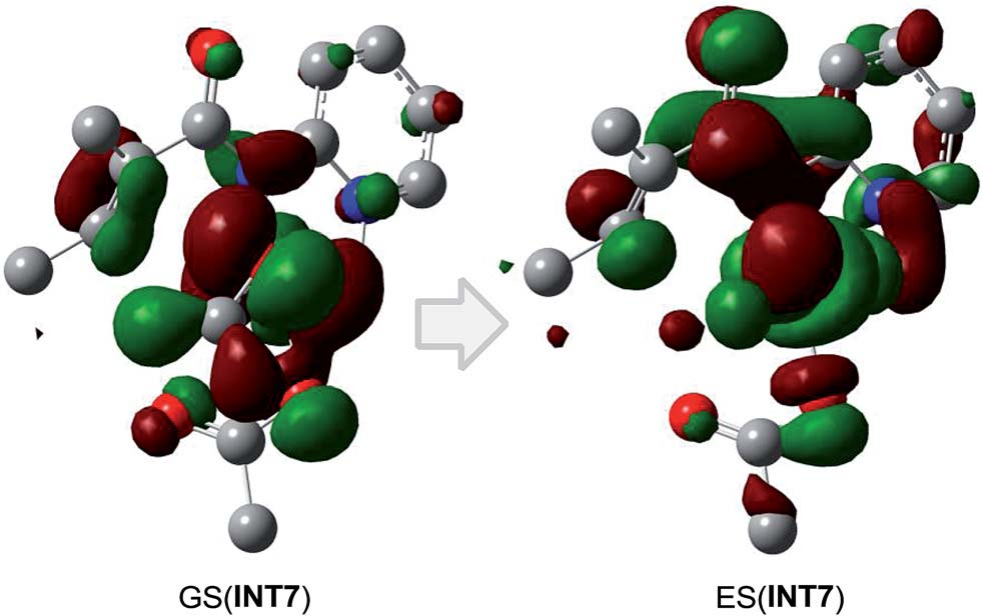
The lowest excited-state doublet derives from excitation of an alpha electron from the singly occupied SOMO (GS(**INT7**)) into the nominally unoccupied LUMO (ES(**INT7**), d_*z*^2^_). The resulting excited state is 44.3 kcal mol^–1^ above the ground state, corresponding to a photon energy of 761.09 nm.

According to the free energy profiles shown in Schemes 5 and 6, the two intramolecular SET pathways will not occur even if it is assumed that the SET process in **INT2** and **INT7** can take place. In an effort to find out whether the intramolecular SET can really happen, we performed time-dependent density functional theory (TD-DFT) calculations to evaluate the transition energy from the ground-state (GS) to the lowest excited state (ES). As shown in Fig. 1 and 2, the lowest-energy internal SET product arises from transfer of a α-spin electron from the olefin π electrons, the singly occupied orbital in **INT2**/**INT7**, to the lowest unoccupied orbital of the Co. The excitation energies for the transitions are 37.2 and 44.3 kcal mol^–1^ for **INT2** and **INT7** respectively, indicating that the intramolecular SET processes of Co^II^-catalyzed C(sp^2^)–H alkoxylation cannot occur.

##### 1.2.2 Intermolecular SET pathways

**Scheme 7:**
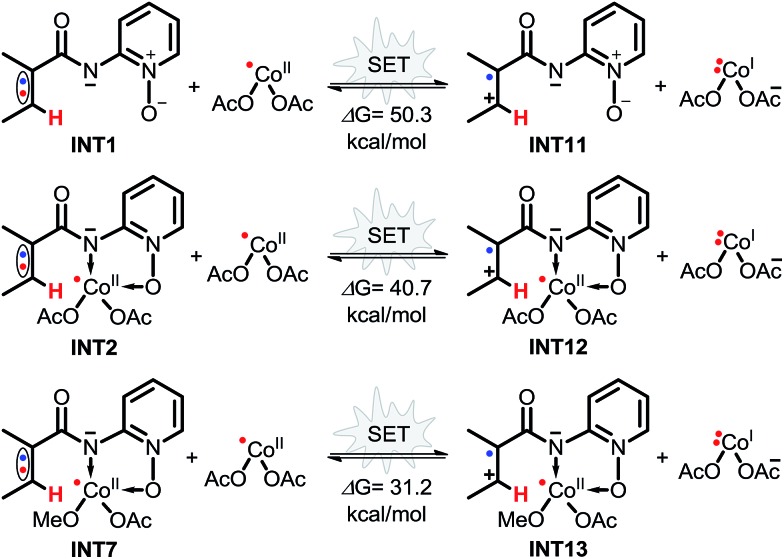
Possible intermolecular SET processes of Co^II^-catalyzed C(sp^2^)–H alkoxylation.

Inspired by the intermolecular SET mechanism of Cu^II^-catalyzed C(sp^2^)–H functionalization reported by Stahl and Ertem,^17^ we considered the possible intermolecular SET transition processes for this reaction. As shown in Scheme 7, the Gibbs free energy differences for intermolecular SET processes range from 31.2 to 50.3 kcal mol^–1^, demonstrating that it would be difficult for the intermolecular SET to occur.

### 2. The possible pathways of Co^III^-catalyzed C(sp^2^)–H alkoxylation

**Scheme 8:**
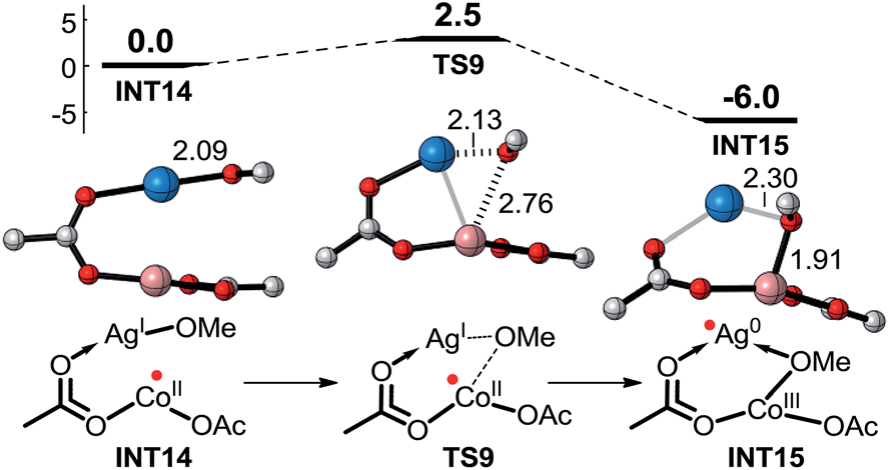
The oxidation process from Co^II^ to Co^III^ by AgOMe.

We have explored almost all the possible mechanisms for Co^II^-catalyzed C–H alkoxylation under basic conditions, but the above calculated results fail to explain the experimental results reasonably. As mentioned above, Co^II^ might be oxidized to Co^III^ by AgOMe, and thus the Co^III^-catalyzed C(sp^2^)–H functionalization reaction pathway would be feasible. As shown in Scheme 8, the energy barrier for oxidation of Co^II^ to Co^III^ by AgOMe is only 2.5 kcal mol^–1^, showing that it would be very facile. Then, starting from **Co^III^(OAc)_2_(OMe)**, there are also two kinds of reaction mechanisms including SET and CMD starting with the Co^III^ catalyst and **INT1**.

#### 2.1 Concerted metalation–deprotonation pathway for Co^III^-catalyzed C(sp^2^)–H alkoxylation

**Scheme 9:**
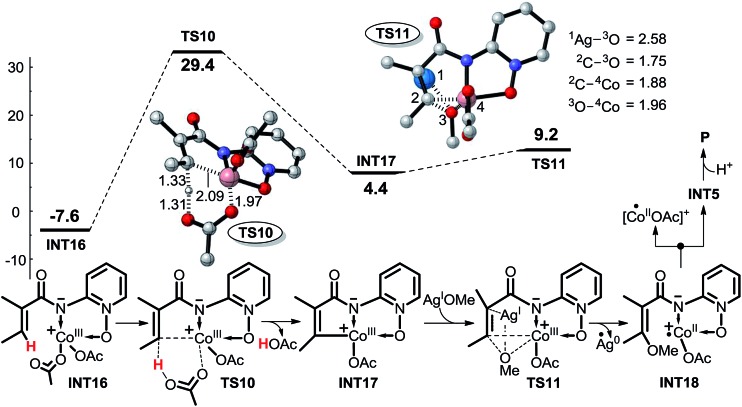
Possible CMD mechanism (pathway 4) of Co^III^-catalyzed C(sp^2^)–H alkoxylation (energy: kcal mol^–1^, distance: Å).

To facilitate comparison, the ligands around the cobalt atom are unchanged from **INT2** to **INT16**. Two key transition states, **TS10** associated with concerted metalation–deprotonation process and **TS11** associated with the OMe transfer, are assigned to the CMD mechanism of Co^III^-catalyzed C(sp^2^)–H alkoxylation, shown as pathway 4 depicted in Scheme 9. The structure of transition state **TS11** indicates that the OMe is transferred by a three membered (C–O–Co) ring, which is remarkably different from the direct OMe transfer from AgOMe in transition state **TS3** of pathway 1. Obviously, the first energy barrier *via* the CMD pathway, at 37.0 kcal mol^–1^ (Scheme 9), is extremely high, indicating that C–H activation *via* this pathway is very energy-demanding.

#### 2.2 Possible single electron transfer pathways for Co^III^-catalyzed C(sp^2^)–H alkoxylation

**Scheme 10:**
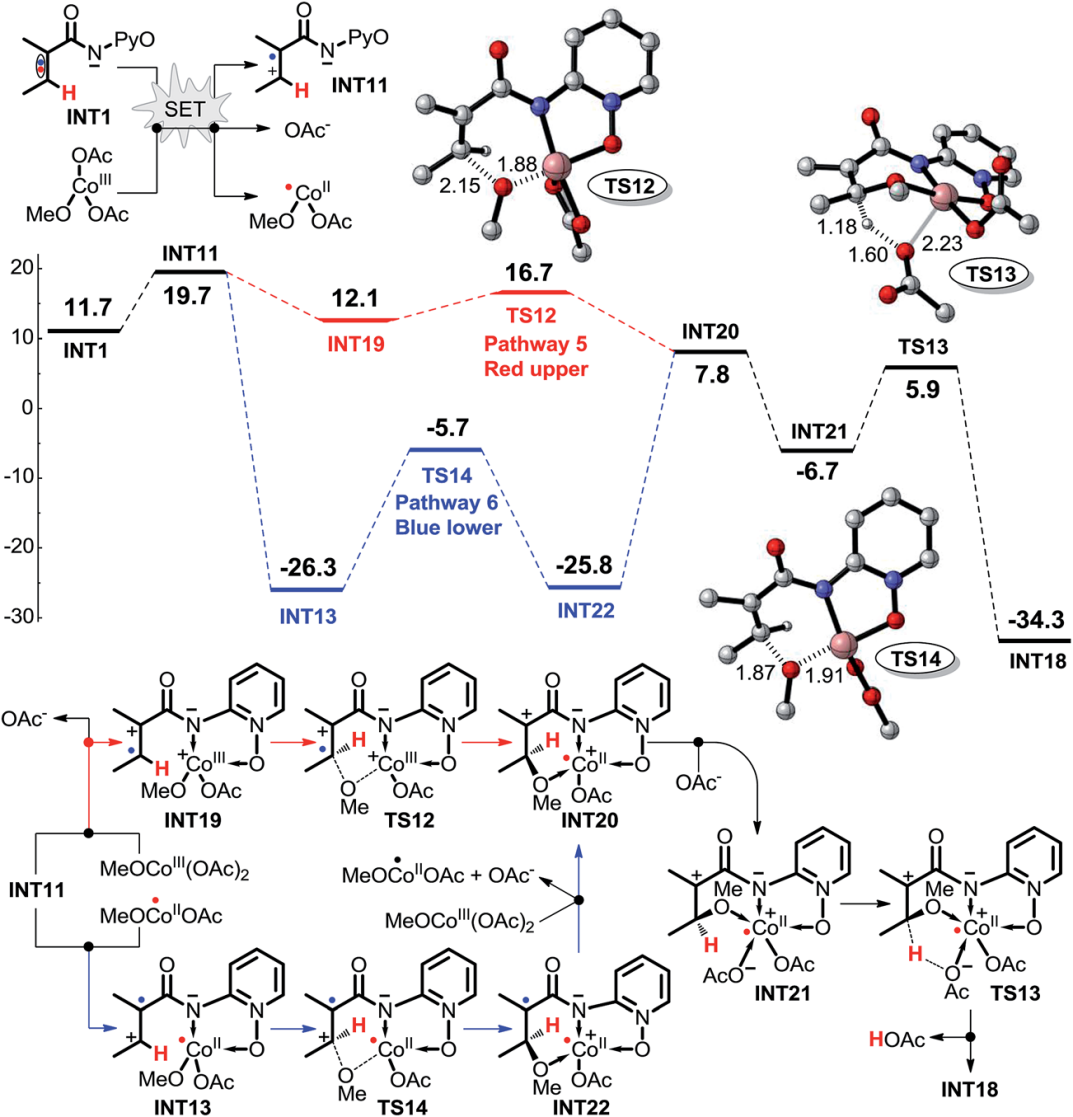
Possible intermolecular SET pathways 5 (red, upper) and 6 (blue, lower) of Co^III^-catalyzed C(sp^2^)–H alkoxylation (energy: kcal mol^–1^, distance: Å).

For Co^III^–**INT1** complexes such as **INT16** depicted in Scheme 9, the spin multiplicity of an intermediate would change from singlet to triplet *via* the intramolecular SET. This would appear to be theoretically impossible, and it would seem to be unnecessary to study the intramolecular SET pathways for Co^III^-catalyzed C(sp^2^)–H alkoxylation. As shown in Scheme 10, the intermolecular SET should be possible for Co^III^ (*i.e.***Co^III^(OAc)_2_(OMe)**) due to the low energy difference of 8.0 kcal mol^–1^ in the SET process. We have suggested and have investigated four possible intermolecular SET pathways, including pathways 5, 6, 7, and 8, starting from **INT11**.

As shown in Scheme 10, pathway 5 comprises three key reaction steps: coordination of intermediate **INT11** with **Co^III^(OAc)_2_(OMe)**, the transfer of ligand OMe from Co^III^ to electron-deficient carbon *via* transition state **TS12**, and the proton transfer from the olefinic carbon to OAc^–^*via* transition state **TS13**. Furthermore, Co^II^ is oxidized to Co^III^ by AgOMe and simultaneously **INT18** dissociates, completing the cycle. Another OAc^–^ coordinates with Co^II^ to form **INT21** before the proton transfer process takes place. The Gibbs free energy barriers for **TS12** and **TS13** are 4.6 and 12.6 kcal mol^–1^, respectively.

In pathway 6 depicted in Scheme 10 the coordination of **INT11** with the **Co^II^(OAc)(OMe)** generated in the SET step affords intermediate **INT13**. There are still two transition states, **TS14** and **TS13** in pathway 6, which shares the same proton transfer mechanism with pathway 5. The energy barrier of the OMe transfer process *via* transition state **TS14** (20.6 kcal mol^–1^) is higher than that (4.6 kcal mol^–1^) in pathway 5, indicating that pathway 5 should be more favorable.

**Scheme 11:**
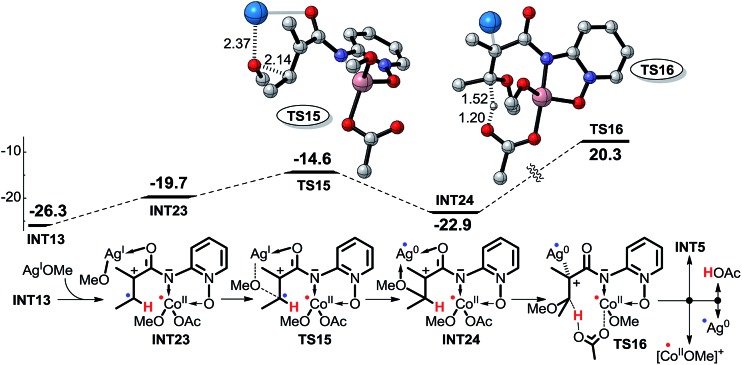
Possible intermolecular SET pathway 7 of Co^III^-catalyzed C(sp^2^)–H alkoxylation (energy: kcal mol^–1^, distance: Å).

**Scheme 12:**
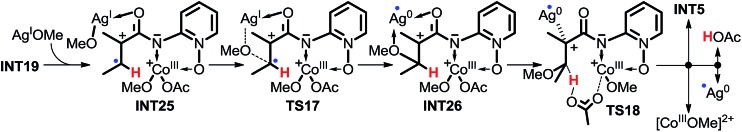
Possible intermolecular SET pathway 8 of Co^III^-catalyzed C(sp^2^)–H alkoxylation (energy: kcal mol^–1^, distance: Å).

In pathway 7, depicted in Scheme 11, intermediate **INT23** is first formed by the weak interaction between intermediate **INT13** and AgOMe, and then the OMe group is transferred directly from AgOMe to the olefinic carbon *via* transition state **TS15**. Subsequently, the OAc^–^ anion abstracts the proton of the olefinic carbon *via* transition state **TS16**. The high energy barrier of 43.2 kcal mol^–1^ for transition state **TS16** excludes this pathway. Furthermore, for pathway 8, depicted in Scheme 12, the other olefinic carbon forms a covalent bond automatically with the OMe group which renders it impossible for us to locate the structures of transition state **TS17** and intermediate **INT26**, so we cannot explore this pathway in any more detail.

If the pre-catalyst Co^II^(OAc)_2_ were to be oxidized to a Co^III^ intermediate, then the intermolecular SET pathway 5 becomes the most energetically favorable among all the eight possible pathways being considered, and we believe that the Co^III^ should be the actual catalyst for this kind of C(sp^2^)–H alkoxylation irrespective of the Co^II^ or Co^III^ compound that was added to the reaction system. The **INT11** has the highest energy (19.7 kcal mol^–1^) in the energy profile of pathway 5, so we can conclude that the intermolecular SET step rather than the C–H activation should be rate-determining in this reaction.

### 3. Combined experimental and theoretical exploration of Co^III^-catalyzed C(sp^2^)–H alkoxylation

**Scheme 13:**
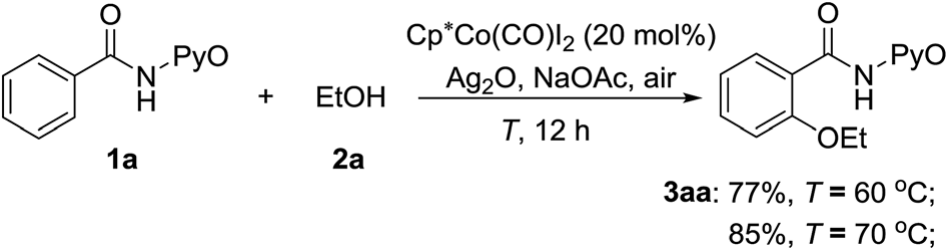
Co^III^-catalyzed ethoxylation of **1a** with ethanol.

The above computational results prompt us to further explore whether the SET mechanism for the direct Co^III^-catalyzed alkoxylation of C(sp^2^)–H bond is experimentally feasible. To confirm the plausibility of this prediction, experimental research was initiated with the reaction between ethanol and 2-benzamidopyridine 1-oxide (**1a**) catalyzed by Cp*Co(CO)I_2_. When a solution of benzamide (**1a**) in ethanol was treated at 70 °C with Cp*Co(CO)I_2_ (20 mol%), Ag_2_O (2 equiv.) and NaOAc (1 equiv.) under an air atmosphere (Scheme 13), the desired ethoxylated product **3aa** was obtained in a good yield.

**Scheme 14:**
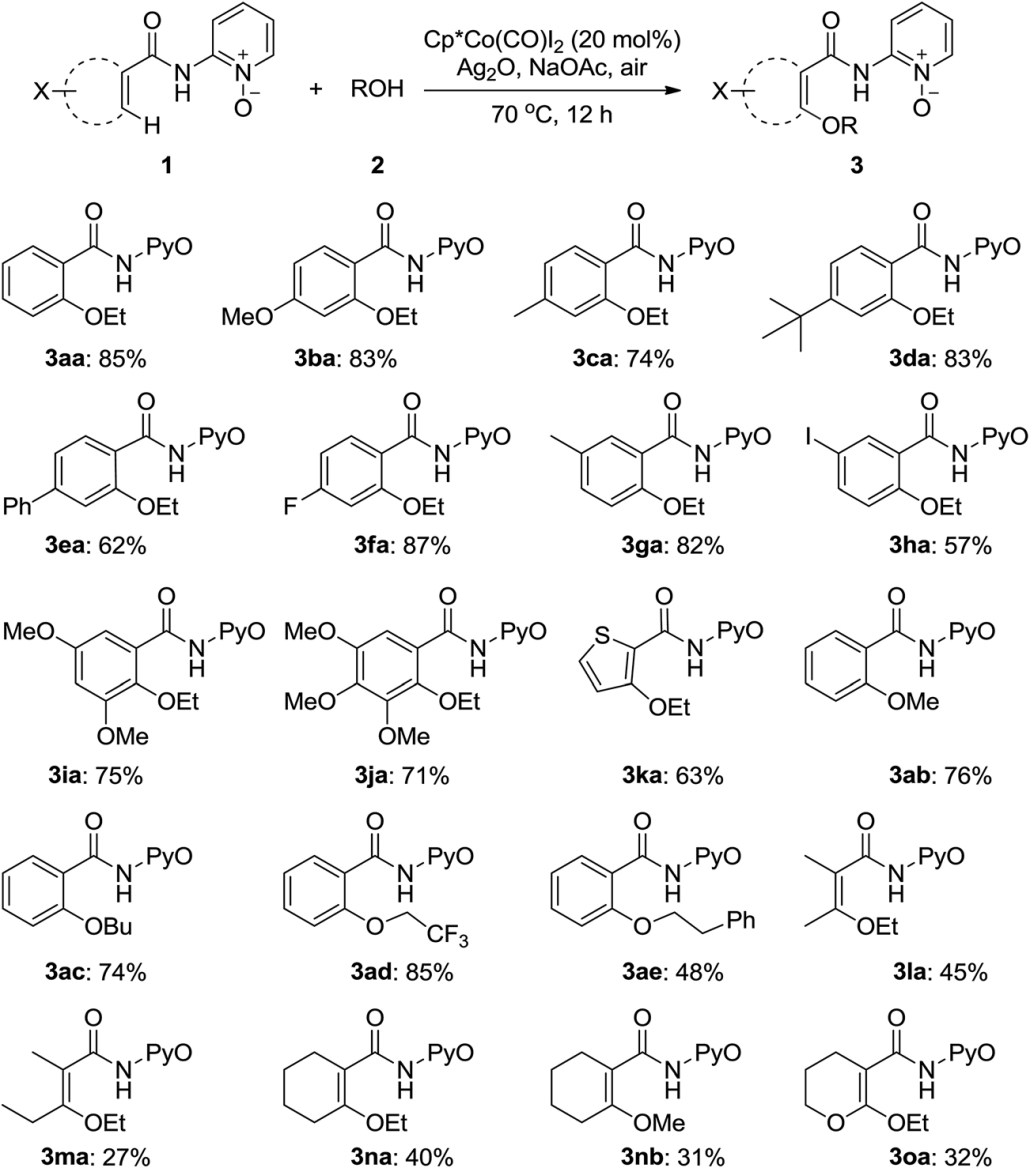
Co^III^-catalyzed alkoxylation of benzamide derivatives with alcohols.

Diversely substituted amides were tolerated under the Cp*Co(CO)I_2_-catalyzed alkoxylation to furnish the desired products (**3**) in moderate to good yields (Scheme 14). The aryl substrates possessing electron-rich and electron-deficient functional groups underwent the transformation successfully (**3aa–3ka**). For *meta*-substituted amides (**1g**, **1h**), the reaction tended to take place at the less hindered position and an iodo group on the aryl ring was also compatible. The heterocyclic substrate (**1k**) afforded the corresponding ethoxylated product in 63% yield. Several alcohols were tested and demonstrated to give the products in yields ranging from 48–85% (**3ab–3ae**). Olefinic carboxamides were also found to follow the protocol under the reaction conditions (**3la–3oa**).

**Scheme 15:**
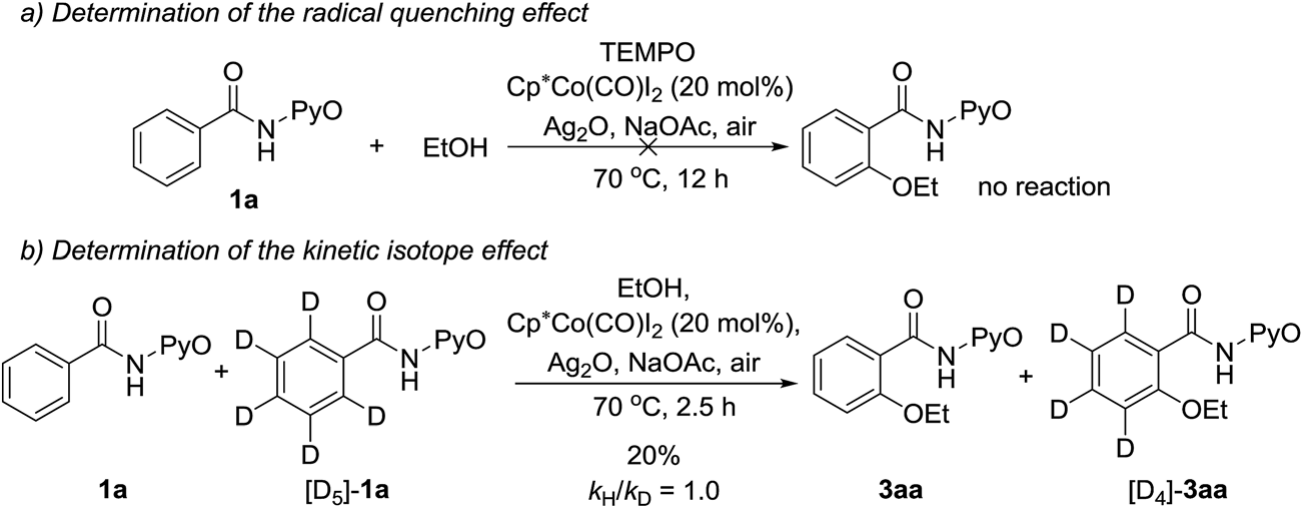
Controlled experiments for Co^III^-catalyzed C(sp^2^)–H alkoxylation.

In addition, control experiments revealed that the addition of 2,2,6,6-tetramethylpiperidine-*N*-oxyl (TEMPO, 1.5 equiv.) as a radical quencher completely inhibits the reaction (Scheme 15a). This result indicates that a radical pathway (SET mechanism) is involved. A 1 : 1 mixture of **1a** and [D5]-**1a** was then treated with ethanol. No kinetic isotope effect (KIE = 1.0) was obtained (Scheme 15b), suggesting that C–H bond cleavage of arenes is not the rate-limiting step, in agreement with our calculated results. Additionally, the electron paramagnetic resonance (EPR) spectrum of the reaction system demonstrated the existence of the single electron (*g* = 2.23003, see the ESI†). The above experiments can be explained by our computational study, which they support.

**Scheme 16:**
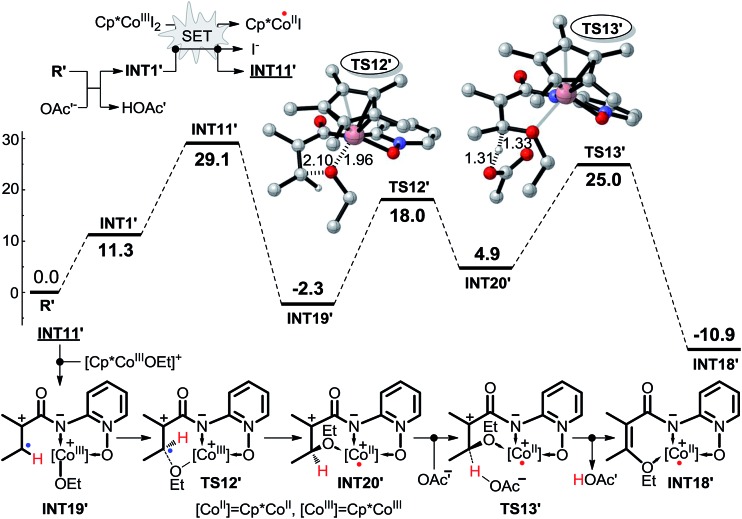
The mechanism of Cp*Co^III^ complex catalyzed C(sp^2^)–H alkoxylation obtained at the M06-L(SMD, ethanol)/6-311++G(2df, 2pd)//SDD level (energy: kcal mol^–1^, distance: Å).

**Scheme 17:**
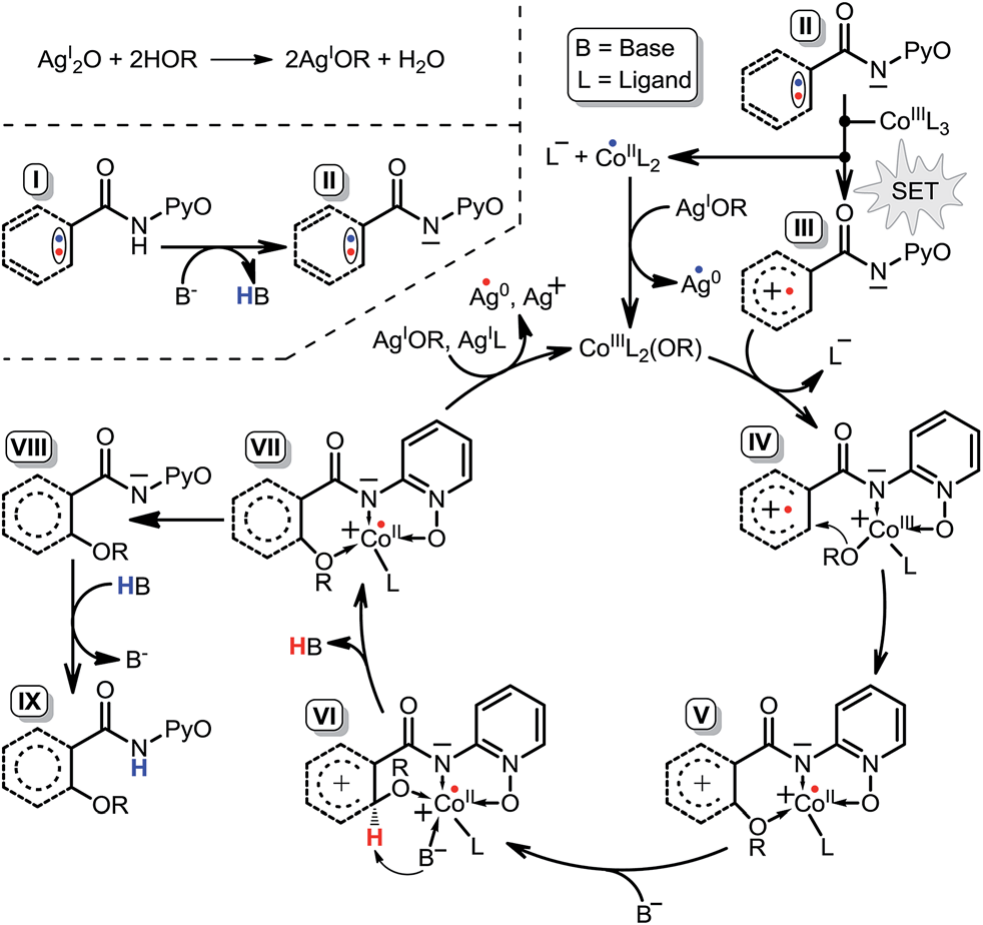
Fundamental reaction pathway of Co^III^-catalyzed C(sp^2^)–H alkoxylation.

We have performed a theoretical study on the mechanism of Cp*Co^III^ complex catalyzed C–H activation. As shown in Scheme 16, it is very similar to the favorable pathway 5 catalyzed by the Co^III^ complex, and the SET process is still the rate-determining step based on the energy profile, which is also in agreement with the KIE and EPR experiments. Based on the above computational and experimental results, we can propose the general mechanism for not only the Co^II^- but also the Co^III^-catalyzed C(sp^2^)–H alkoxylation reaction. As shown in Scheme 17, AgOR (OR = alkoxy group) is generated and is the actual oxidant, and the reactant **I** is transformed to intermediate **II** by proton transfer to the base. First, it is an intermolecular SET process between intermediate **II** and Co^III^L_3_ (L = ligand) generating intermediate **III**, L^–^, and Co^II^L_2_, which can be oxidized to Co^III^L_2_(OR) by AgOR. Second, intermediate **IV** is formed by the coordination of intermediate **III** with Co^III^L_2_(OR). Subsequently, the OR group is transferred from the Co to the substrate leading to the formation of intermediate **V**. Intermediate **VI** is formed by the coordination of base B^–^. As shown in Scheme 16, it should be noted that the coordination would not occur when the catalyst is Cp*Co^III^. The next step is the proton transfer to base ligand and, finally, intermediate **VII** dissociates to **VIII** and Co^II^. Co^II^ can be oxidized again to Co^III^ which catalyzes the next cycle, and intermediate **VIII** continues to be converted to the product **IX**. Although the tridentate chelate **VII** should be very stable, we still think it can be dissociated directly, because the structural transformation to **VIII** (*i.e.***INT18** in Scheme 10 or **INT18′** in Scheme 16) is a highly exothermic process, which can compensate for the energy for the direct dissociation.

## Conclusions

We have explored multiple possible SET and CMD mechanisms of Co^II^/Co^III^-catalyzed alkoxylation of C(sp^2^)–H bond in theory, and found that the intermolecular SET pathway in which the Co^III^ works as the actual catalyst (pathway 5) should be the most favorable pathway, even when the Co^II^(OAc)_2_ was added as a catalyst. Generally, there are three key steps in the favorable pathway of the high-valent Co-catalyzed C(sp^2^)–H alkoxylation, *i.e.* the intermolecular SET between the reactant and Co^III^, the alkoxylation and, finally, the breaking of C(sp^2^)–H bond. The calculated results indicate that the SET step, rather than the C(sp^2^)–H activation, is rate-determining. Guided by the computed mechanism, the first Co^III^-catalyzed C(sp^2^)–H alkoxylation is reported. A variety of amides with electron-rich and electron-poor functional groups were experimentally suitable for the Cp*Co(CO)I_2_-catalyzed alkoxylation, thus extending the application for cobalt-catalyzed functionalization of C–H bonds. All observations in the experiment can be explained by our computational results.

This work provides another special SET-based example in the context of chelate-directed C(sp^2^)–H functionalization. The detailed Co^II^/Co^III^-catalyzed SET and CMD models and pathways should be helpful for chemists to understand the general mechanism and the roles of the additives and catalysts in the Co^II^/Co^III^-catalyzed C–H functionalization, and thus provide valuable insights into rational prediction and design of the more efficient catalysts in this kind of reaction.

## Computational and experimental details

All of the calculations were performed using Gaussian 09.^23^ Computed structures are illustrated using CYLView.^24^ The density functional theory (DFT) method was applied since it has been successfully used in many studies of organocatalysis,^25^ organometallic catalysis,^26^ and biological reaction mechanisms.^27^ The density functional theory calculations were carried out with the M06-L functional in the presence of the SMD continuum solvation model with methanol (or ethanol) as the solvent. All the structures were completely optimized using a combined basis set: the LanL2DZ basis set^28^ was used for Co, I, and Ag along with the 6-31G(d, p) basis set for C, N, H, and O. The frequency calculations were performed at the same level at 298 K and 1 atm, and vibrational analysis was performed to confirm the optimized stationary points as true minima with no imaginary frequency, or transition states with one and only one imaginary frequency, on the potential energy surface and to obtain the thermodynamic data. On the basis of the optimized structures at the M06-L/6-31G(d, p)//LanL2DZ level, the energies were then refined by M06-L/6-311++G(2df, 2pd)//SDD^29^ single-point calculations with the same solvent effects. We checked the *S*^2^ values of all the stationary points obtained above, and found that all the differences between the obtained *S*^2^ value and the normal *S*^2^ value are less than 7.5%, so spin contamination can safely be ignored.

We chose to conduct discussions based on Gibbs free energies rather than Born–Oppenheimer energies, which are the electronic (including nuclear-repulsion) energies plus ZPEs. Free energy contributions were added to single-point M06-L electronic energies computed with the SDD basis set on Co, I, and Ag and the 6-311++G(2df, 2pd) basis set on all other atoms to arrive at final, composite free energies.

TD-DFT calculations were performed to predict the UV/visible electronic excitations of postulated intermediates. The M06-L density functional, the LanL2DZ pseudopotential basis set on Co, I, and Ag, and the 6-311++G(2df, 2pd) basis set on all other atoms were used for the TD-DFT calculations in methanol with the SMD continuum solvation model.

**Scheme 18:**
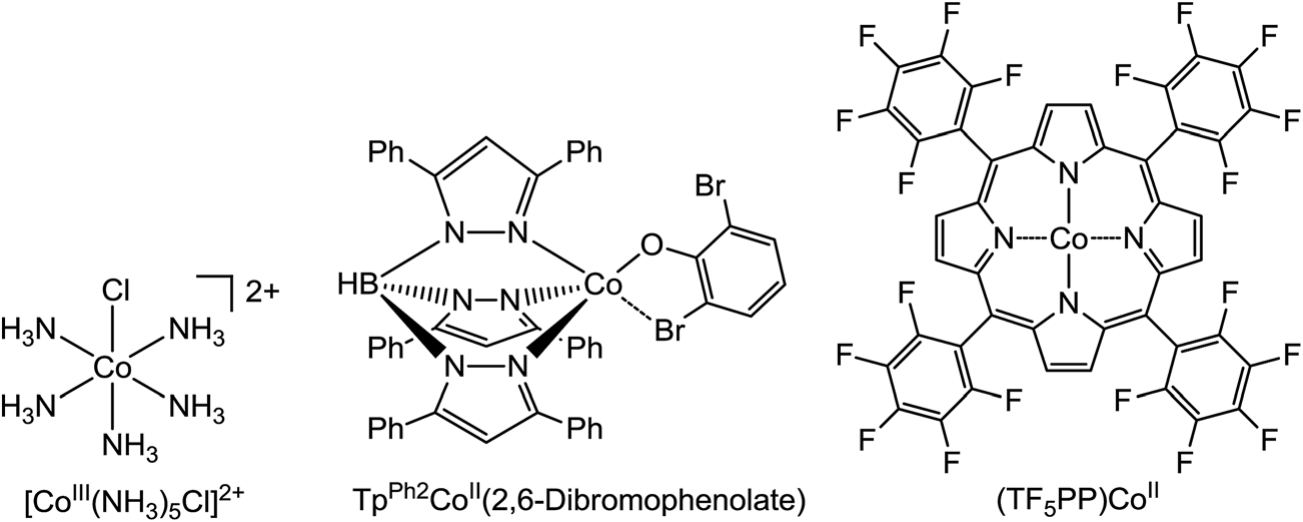
The structures of representative Co complexes.

Bakac *et al.* have performed TD-DFT calculations on several Co^III^ complexes, and confirmed that the computational results are close to the experimental results.^30^ In order to test the reliability of M06-L functional and the TD-DFT calculations in this paper, the structures of **[Co^III^(NH_3_)_5_Cl]^2+^**,^31^**Tp^Ph2^Co^II^(2,6-dibromophenolate)**,^32^ and **(TF_5_PP)Co^II^** depicted in Scheme 18 33 have been chosen and completely optimized at the M06-L(SMD, water)/6-31G(d, p)//LanL2DZ, M06-L(SMD, chloroform)/6-31G(d, p)//LanL2DZ, and M06-L(SMD, dichloromethane)/6-31G(d, p)//LanL2DZ levels, respectively. It should be noted that the bond lengths of the optimized structures of **Tp^Ph2^Co^II^(2,6-dibromophenolate)** and **(TF_5_PP)Co^II^** have tiny differences (<0.05 Å) with those of the corresponding X-ray crystal structures reported in the experiments,^32^,^33^ indicating that the M06-L method is suitable for the Co complexes. Then, TD-DFT calculations based on the optimized structures have been carried out at the M06-L(SMD, water)/6-311++G(2df, 2pd)//LanL2DZ, M06-L(SMD, chloroform)/6-311++G(2df, 2pd)//LanL2DZ, and M06-L(SMD, dichloromethane)/6-311++G(2df, 2pd)//LanL2DZ levels, respectively. As summarized in Table 1, the computational *λ*_max_ values for the three Co complexes are close to those reported in experiments,^31^–^33^ which demonstrates that the results of TD-DFT calculations should be reliable.

**Table 1:** Computational and experimental *λ*_max_/nm (eV)

	M06-L(SMD)	Experimental
**[Co^III^(NH_3_)_5_Cl]^2+^**	455.35 (2.72)	474 (2.62)
	322.54 (3.84)	338 (3.67)
**Tp^Ph2^Co^II^(2,6-dbp)**	1380.07 (0.90)	1475 (0.84)
	810.68 (1.53)	800 (1.55)
	670.29 (1.85)	660 (1.88)
	586.77 (2.11)	570 (2.18)
**(TF_5_PP)Co^II^**	542.92 (2.28)	552 (2.25)
	527.16 (2.35)	524 (2.37)

**Scheme 19:**
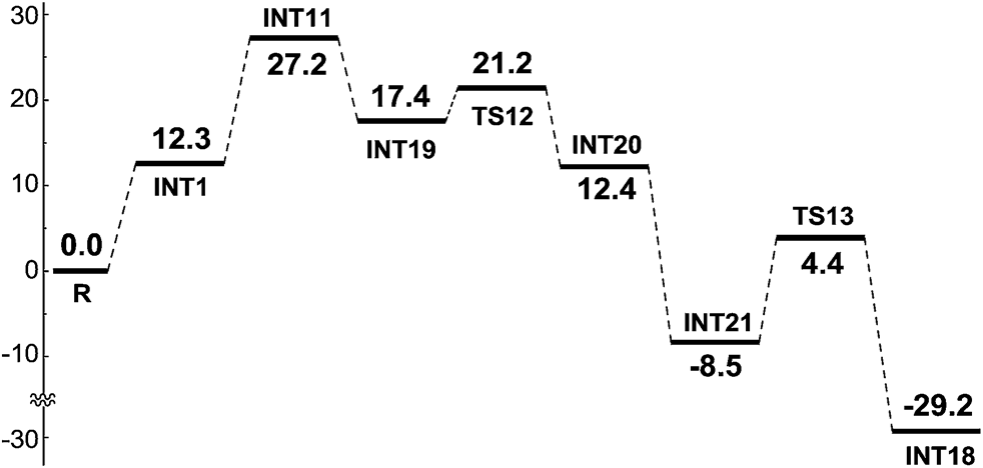
Gibbs free energy Δ*G*_50%_ for SET pathways 5 of Co^III^-catalyzed C(sp^2^)–H alkoxylation (energy: kcal mol^–1^).

We have calculated and compared the free energy Δ*G*_tot_ and the Δ*G*_50%_ (the entropy is cut by 50%) profiles of the favorable reaction pathway (pathway 5). As shown in Schemes 10 and 19, although a computational error does exist in the entropy calculation for a multimolecular reaction step (such as a SET process), the calculated results can still predict exactly that the SET process is the rate-determining step, which is in agreement with the KIE experiment. Therefore, we think that the calculated results can reliably explain the experimental results.

Recently, Singleton *et al.* investigated the mechanism of alcohol-mediated Morita–Baylis–Hillman (MBH) reactions using the most popular DFT methods, *i.e.* B3LYP and M06-2X.^34^ They found that the calculated results by the two methods are remarkably different, and the rate-determining step cannot be predicted correctly by using the M06-2X method, so they concluded that the computations aid in interpreting observations but fail utterly as a replacement for experiment. In this work, similar computational tests to Singleton have been performed for the key C–H activation in the favorable reaction pathway (pathway 5) by using B3LYP and M06-L methods. As summarized in Table 2, the calculated results indicate that the free energy barriers ΔΔ*G*_tot_, ΔΔ*G*_50%_, and ΔΔ*G*_explicit_ (calculated in the explicit solvents without implicit model) are close and the free energy barriers obtained by the different DFT methods are not significantly different, which is remarkably different from Singleton's work. As mentioned above, we believe that the computational errors in this system are not very significant and the calculated results using the M06-L method are consistent with experiment. In addition, multiple pathways involving explicit methanol for this kind of reaction^35^ are considered in both implicit and explicit models, but the H atom of HOMe returns automatically when we try to locate the possible structures of the transition states **TS19**, **TS20**, **TS21**, and intermediate **INT27** (depicted in Scheme S1 of the ESI†), which is mainly because the basicity of OMe^–^ is stronger than that of OAc^–^, so we did not further explore details of these pathways.

**Table 2:** Gibbs free energy barrier for the C–H activation step calculated at the DFT(SMD, methanol)/6-311++G(2df, 2pd)//SDD level

Method	kcal mol^–1^
M06-L(ΔΔ*G*_tot_[**TS13–INT21**])	12.6
M06-L(ΔΔ*G*_50%_[**TS13–INT21**])	12.9
M06-L(ΔΔ*G*[**TS13_explicit_–INT21_explicit_**])^*a*^	13.5
B3LYP(ΔΔG_tot_[**TS13_B3LYP_–INT21_B3LYP_**])	8.0
B3LYP(ΔΔ*G*_50%_[**TS13_B3LYP_–INT21_B3LYP_**])	9.4

^*a*^The transition state **TS13_explicit_** has firstly been located in the 10 Å box of the explicit solvents at the ONIOM(M06-L/6-31G(d, p)//LanL2DZ:UFF) level, it should be noted that additional five methanol molecules were putted into the high level, and all the other methanol molecules were putted into the low level. Then IRC calculation was performed to locate the corresponding intermediate **INT21_explicit_**. The single-point energies of the stationary points were refined at the higher ONIOM(M06-L/6-311++G(2df, 2pd)//SDD:UFF) level.

The M06-L method has been successfully used in the theoretical report on the mechanism of transition-metal catalyzed C–H oxidation,^17^ and the above computational tests also indicate that it should be suitable for investigating the systems including Co complexes.

Alcohol (1.2 mL) was added to a mixture of amide (0.15 mmol), Ag_2_O (69 mg, 0.3 mmol, 2 equiv.), NaOAc (12 mg, 0.15 mmol, 1 equiv.), Cp*Co(CO)I_2_ (14 mg, 20 mol%). Then, the reaction system was stirred at 70 °C for 12 h under an air atmosphere. After cooling to RT, the alcohol was evaporated under reduced pressure, and 2 N HCl (5 mL) was added to the residue. The mixture was extracted with CH_2_Cl_2_ and the organic layer was dried over anhydrous Na_2_SO_4_. Purification of the residue gave the product **3**.

## Supplementary Material

Supplementary information

## References

[cit1] Cheng C., Hartwig J. F. (2015). Chem. Rev..

[cit2] For selected reviews, see: Chen X. Engle K. M. Wang D. H. Yu J. Q., Angew. Chem., Int. Ed., 2009, 48 , 5094 . 10.1002/anie.200806273 PMC2722958 19557755

[cit3] Song G., Wang F., Li X. (2012). Chem. Soc. Rev..

[cit4] Ackermann L. (2014). Acc. Chem. Res..

[cit5] Mousseau J. J., Charette A. B. (2013). Acc. Chem. Res..

[cit6] (b) Ackermann L., J. Org. Chem., 2014, 79 , 8948 , , For selected examples, see: . 25102352 10.1021/jo501361k

[cit7] (a) Yoshikai N., ChemCatChem, 2015, 7 , 732 , , For selected examples, see: .

[cit8] Grigorjeva L., Daugulis O. (2014). Angew. Chem., Int. Ed..

[cit9] Liu B., Shi B.-F. (2015). Tetrahedron Lett..

[cit10] Gowrisankar S., Sergeev A. G., Anbarasan P., Spannenberg A., Neumann H., Beller M. (2010). J. Am. Chem. Soc..

[cit11] Desai L. V., Malik H. A., Sanford M. S. (2006). Org. Lett..

[cit12] Bhadra S., Matheis C., Katayev D., Goossen L. J. (2013). Angew. Chem., Int. Ed..

[cit13] Zhang L. B., Hao X. Q., Zhang S. K., Liu K., Ren B., Gong J. F., Niu J. L., Song M. P. (2014). J. Org. Chem..

[cit14] Zhang L. B., Hao X. Q., Zhang S. K., Liu Z. J., Zheng X. X., Gong J. F., Niu J. L., Song M. P. (2015). Angew. Chem., Int. Ed..

[cit15] Ackermann L. (2011). Chem. Rev..

[cit16] Chen X., Hao X. S., Goodhue C. E., Yu J. Q. (2006). J. Am. Chem. Soc..

[cit17] Suess A. M., Ertem M. Z., Cramer C. J., Stahl S. S. (2013). J. Am. Chem. Soc..

[cit18] Li J., Ackermann L. (2015). Angew. Chem., Int. Ed..

[cit19] Iqbal J., Bhatia B., Nayyar N. K. (1994). Chem. Rev..

[cit20] Kochi J. K., Tang R. T., Bernath T. (1973). J. Am. Chem. Soc..

[cit21] Zhao Y., Truhlar D. G. (2006). J. Chem. Phys..

[cit22] Marenich A. V., Cramer C. J., Truhlar D. G. (2009). J. Phys. Chem. B.

[cit23] Frisch M. J., et al., Gaussian 09, revision A.02, Gaussian, Inc., Wallingford, CT, 2009.

[cit24] Legault C. Y., CYLview, 1.0b, Université de Sherbrooke, 2009, http://www.cylview.org.

[cit25] Lam Y. H., Houk K. N., Scheffler U., Mahrwald R. (2012). J. Am. Chem. Soc..

[cit26] Wang T., Liang Y., Yu Z. X. (2011). J. Am. Chem. Soc..

[cit27] Xu D. G., Wei Y. S., Wu J. B., Dunaway-Mariano D., Guo H., Cui Q., Gao J. L. (2004). J. Am. Chem. Soc..

[cit28] Wadt W. R., Hay P. J. (1985). J. Chem. Phys..

[cit29] Fuentealba P., Preuss H., Stoll H., Vonszentpaly L. (1982). Chem. Phys. Lett..

[cit30] Bakac A., Pestovsky O., Durfey B. L., Kristian K. E. (2013). Chem. Sci..

[cit31] Kofod P. (1995). Inorg. Chem..

[cit32] Machonkin T. E., Boshart M. D., Schofield J. A., Rodriguez M. M., Grubel K., Rokhsana D., Brennessel W. W., Holland P. L. (2014). Inorg. Chem..

[cit33] Kadish K. M., Han B. C., Franzen M. M., Araullo-McAdams C. (1990). J. Am. Chem. Soc..

[cit34] Plata R. E., Singleton D. A. (2015). J. Am. Chem. Soc..

[cit35] Winter A. (2015). Nat. Chem..

