# Supplementary material for: Mechanistic insights into cobalt(ii/iii)-catalyzed C–H oxidation: a combined theoretical and experimental study
Source: Chem Sci. 2015 Sep 9;6(12):7059–71. doi: 10.1039/c5sc01807b (PMC5947531; doi:10.1039/c5sc01807b)
Supplement: Supplementary file 1 [file SC-006-C5SC01807B-s001.pdf]

Electronic Supplementary Material (ESI) for Chemical Science.
This journal is © The Royal Society of Chemistry 2015

Supporting Information for

Mechanistic Insights into the Cobalt(II/III)-Catalyzed C–H Oxidation: A Combined Theoretical and Experimental Study

Xiao-Kang Guo, Lin-Bao Zhang, Donghui Wei*, and Jun-Long Niu*
The College of Chemistry and Molecular Engineering, Zhengzhou University, Zhengzhou, Henan Province, 450001, P. R. China

*E-mail for D.-H. Wei: donghuiwei@zzu.edu.cn
*E-mail for J.-L. Niu: niujunlong@zzu.edu.cn

Contents **Page**

- Complete Gaussian 09 Reference S2
- Computed Structures, Cartesian Coordinates and Energies S3
- Alternative methanol-assisted proton transfer pathways S168
- TD-DFT Computation of **INT2** and **INT7** Excitation S169
- Reaction Kinetic Isotope Effect Measurements S170
- Reaction System under Added TEMPO S171
- Experimental EPR spectrum of the reaction system S172
- Characterization of Products S173
- NMR and Mass Spectra S179

Complete Gaussian 09 Reference

Gaussian 09, Revision A.02, M. J. Frisch, G. W. Trucks, H. B. Schlegel, G. E. Scuseria, M. A. Robb, J. R. Cheeseman, G. Scalmani, V. Barone, B. Mennucci, G. A. Petersson, H. Nakatsuji, M. Caricato, X. Li, H. P. Hratchian, A. F. Izmaylov, J. Bloino, G. Zheng, J. L. Sonnenberg, M. Hada, M. Ehara, K. Toyota, R. Fukuda, J. Hasegawa, M. Ishida, T. Nakajima, Y. Honda, O. Kitao, H. Nakai, T. Vreven, J. A. Montgomery, Jr., J. E. Peralta, F. Ogliaro, M. Bearpark, J. J. Heyd, E. Brothers, K. N. Kudin, V. N. Staroverov, R. Kobayashi, J. Normand, K. Raghavachari, A. Rendell, J. C. Burant, S. S. Iyengar, J. Tomasi, M. Cossi, N. Rega, J. M. Millam, M. Klene, J. E. Knox, J. B. Cross, V. Bakken, C. Adamo, J. Jaramillo, R. Gomperts, R. E. Stratmann, O. Yazyev, A. J. Austin, R. Cammi, C. Pomelli, J. W. Ochterski, R. L. Martin, K. Morokuma, V. G. Zakrzewski, G. A. Voth, P. Salvador, J. J. Dannenberg, S. Dapprich, A. D. Daniels, O. Farkas, J. B. Foresman, J. V. Ortiz, J. Cioslowski, and D. J. Fox, Gaussian, Inc., Wallingford CT, 2009.

Computed Structures, Cartesian Coordinates and Energies

R	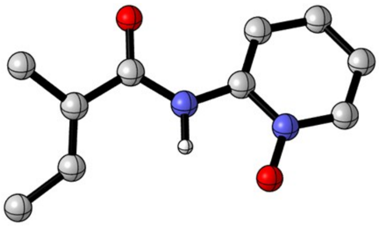	INT6	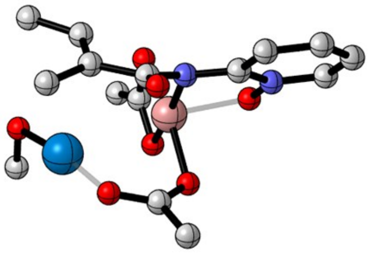
OAc^{-}	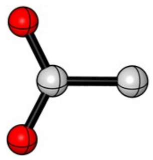	INT7	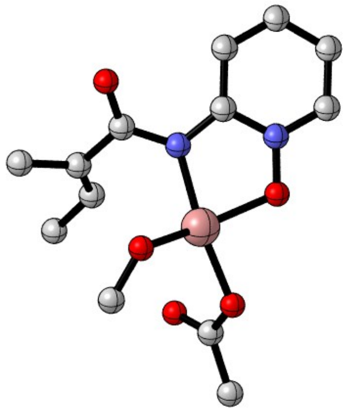
OPiv^{-}	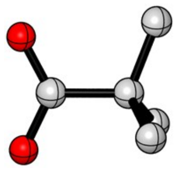	INT8	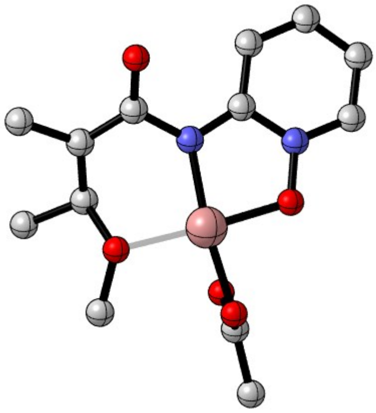
OMe^{-}	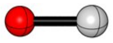	INT10	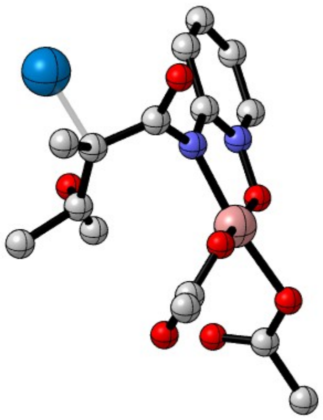

**AgOAc**	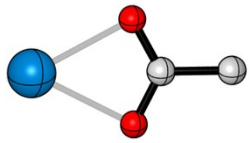	**INT11**	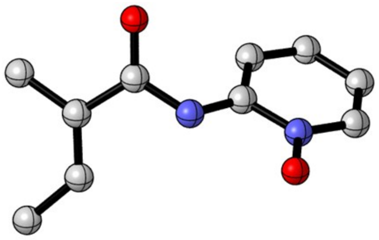
**AgOMe**	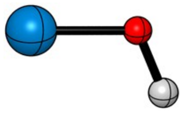	**INT12**	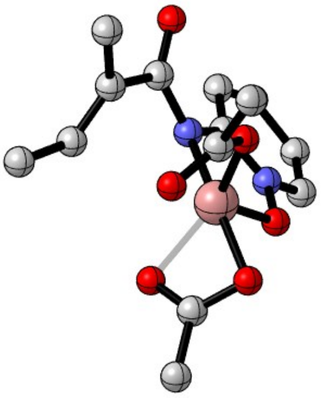
**$Co(OAc)_2$**	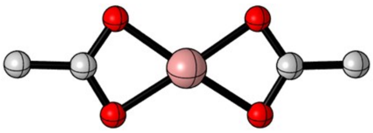	**INT13**	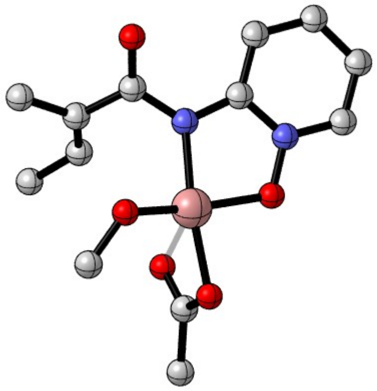
**$Co(OAc)_2^-$**	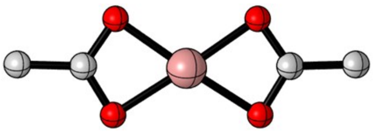	**INT16**	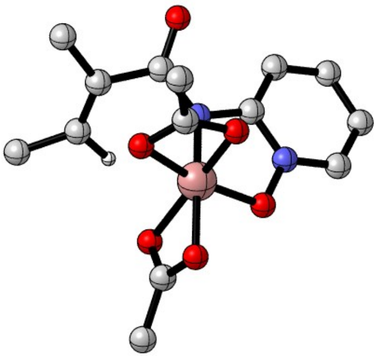
**$Co^{III}(OAc)_2OMe$**	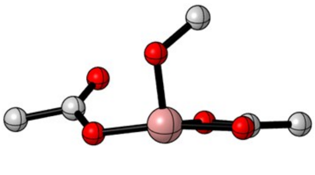	**INT17**	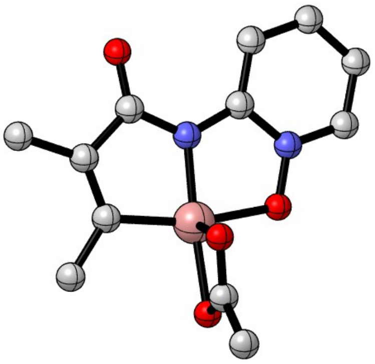

**Co^{II}(OAc)(OMe)**	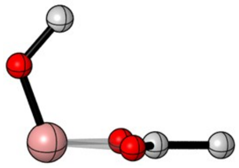	**INT18**	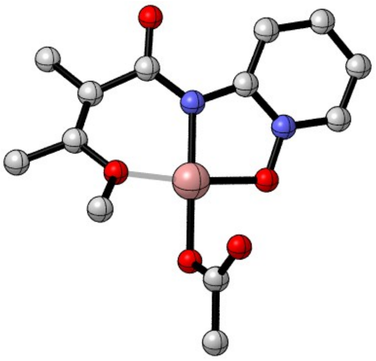
**HOAc**	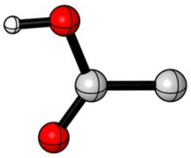	**INT19**	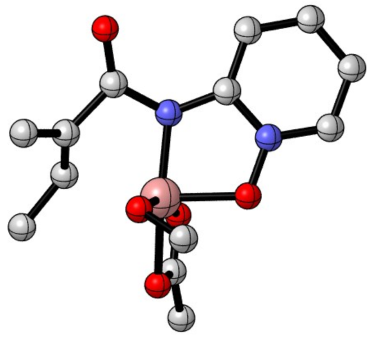
**HOMe**	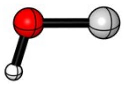	**INT20**	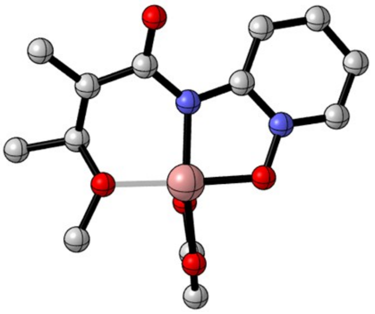
**HOPiv**	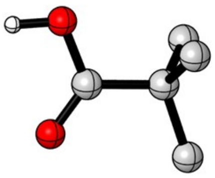	**INT21**	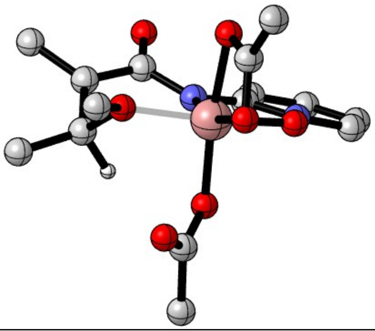
**INT1**	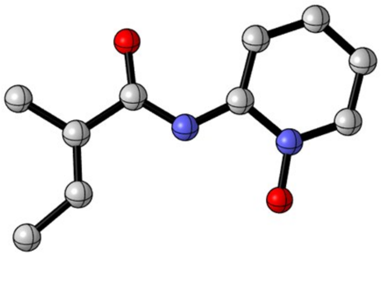	**INT22**	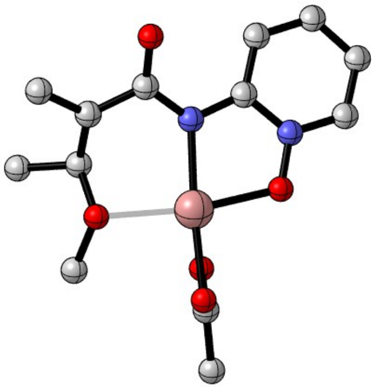

INT2	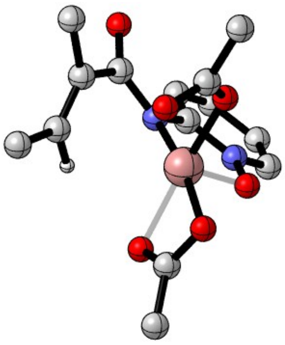	INT23	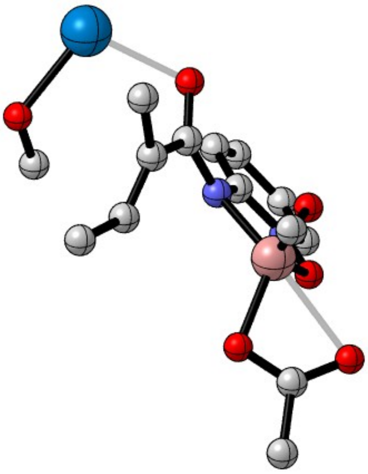
INT3	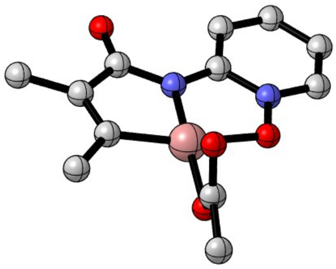	INT24	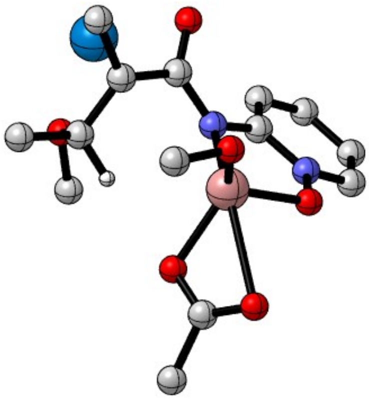

R’	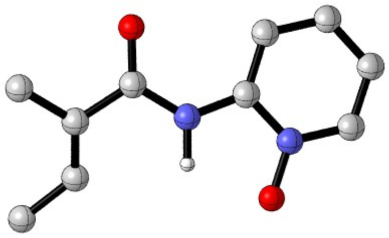	I^-	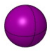
OAc’$^-$	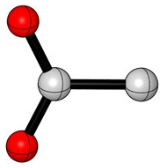	INT11’	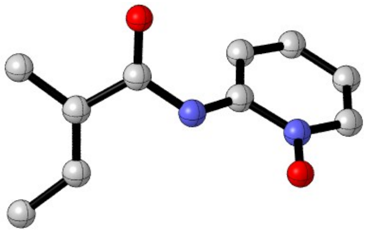
HOAc’	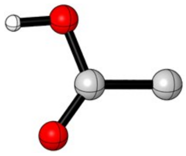	$[Cp^*Co^{III}OEt]^+$	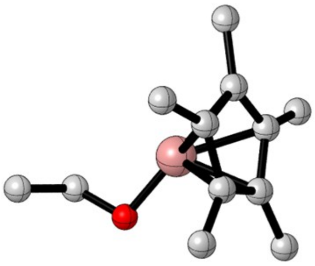

<table>
<tr><td>INT1′</td><td>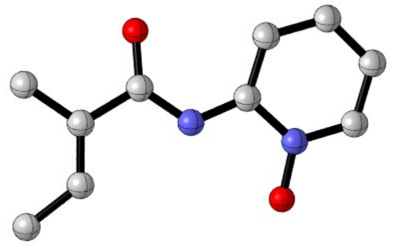</td><td>INT19′</td><td>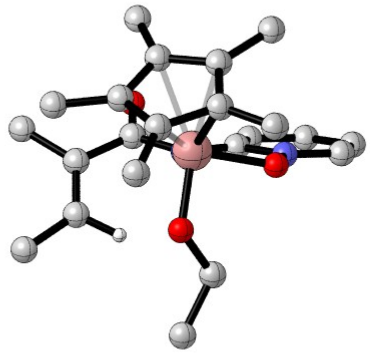</td></tr>
<tr><td>$Cp^*Co^{III}I$</td><td>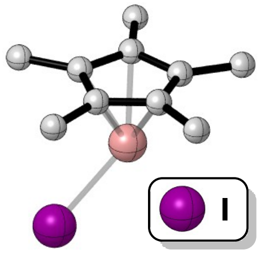</td><td>INT20′</td><td>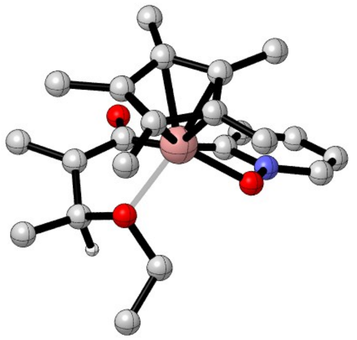</td></tr>
<tr><td>$Cp^*Co^{III}I_2$</td><td>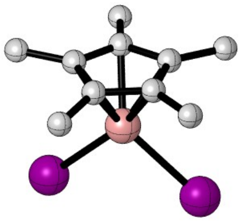</td><td>INT18′</td><td>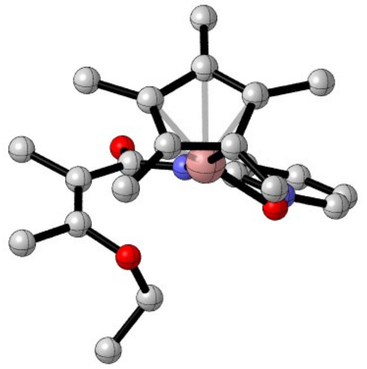</td></tr>
</table>

<table>
<tr><td>$INT21_{explicit}$</td><td>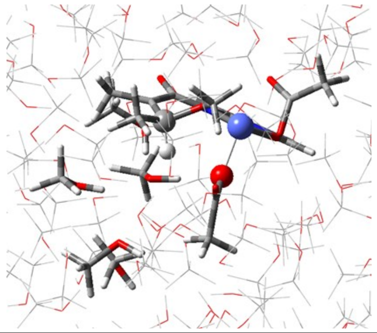</td><td>$TS13_{explicit}$</td><td>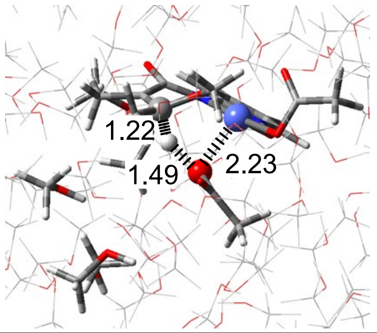</td></tr>
</table>

**INT21$_{B3LYP}$**	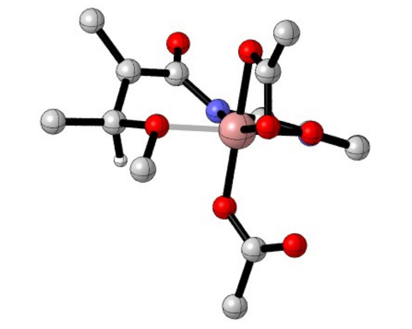	**TS13$_{B3LYP}$**	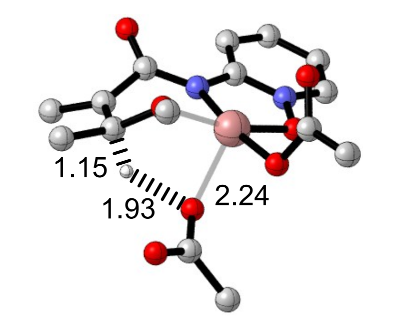

Cartesian Coordinates and Energies

R (Singlet)

E= -648.344514146 a.u.

C	4.152348	1.562521	-0.454751
C	2.815436	0.929085	-0.364497
C	2.523259	-0.310500	0.077712
C	3.526618	-1.323004	0.529800
H	4.961290	0.939890	-0.068496
H	3.586948	-2.155689	-0.178983
C	1.133178	-0.827323	0.045938
N	0.129154	0.115372	0.086759
O	0.897874	-2.038133	0.003536
C	-1.225241	-0.092788	-0.002451
C	-1.899479	-1.282390	-0.262898
C	-3.285584	-1.293499	-0.327367
H	-1.313492	-2.179128	-0.408807
C	-3.302053	1.057817	0.120974
C	-3.990835	-0.108512	-0.132358
H	-3.807916	-2.222416	-0.529291
H	-3.761173	2.024542	0.282539
H	-5.073359	-0.073492	-0.174391
O	-1.302603	2.180831	0.433159
N	-1.942989	1.076148	0.190139
H	0.342380	1.092699	0.277582
H	4.386955	1.817493	-1.495060
H	4.162149	2.513049	0.091849
H	4.525552	-0.898599	0.635217
H	3.238616	-1.759976	1.491490
H	2.000852	1.550974	-0.736092

OAc$^-$ (Singlet)

E= -228.684747791 a.u.

O	-0.748436	1.131945	0.002446
C	-0.186621	-0.000066	-0.012266

C	1.341985	-0.010375	-0.004542
H	1.700580	0.086783	1.027342
H	1.750491	-0.944076	-0.402542
H	1.757964	0.834105	-0.563091
O	-0.769217	-1.121215	0.002447

OPiv^{-} (Singlet)

E= -346.646202042 a.u.

C	-0.519280	0.010331	-0.000077
C	1.036703	0.029960	0.000130
O	1.606368	-1.099747	0.000343
O	1.621970	1.148715	0.000108
C	-0.990953	-0.744175	-1.244240
H	-0.583281	-1.760190	-1.261799
H	-2.085342	-0.816755	-1.273379
H	-0.669729	-0.240963	-2.164493
C	-0.992123	-0.737817	1.247512
H	-0.670995	-0.230310	2.165424
H	-2.086613	-0.809235	1.276322
H	-0.585545	-1.754181	1.270588
C	-1.099531	1.418408	-0.003727
H	-2.196519	1.392033	-0.003222
H	-0.778488	1.986154	0.876042
H	-0.779084	1.981463	-0.886684

OMe^{-} (Singlet)

E= -115.247133716 a.u.

O	0.000000	0.000000	0.799971
C	0.000000	0.000000	-0.542073
H	0.000000	1.013269	-1.049109
H	0.877516	-0.506634	-1.049109
H	-0.877516	-0.506634	-1.049109

AgOAc (Singlet)

E= -375.635761347 a.u.

O	-0.999120	-1.118286	-0.008089
C	-1.592508	0.002200	-0.014636
C	-3.102064	0.000641	0.005058
H	-3.443675	-0.104652	1.040103
H	-3.510719	0.933755	-0.386705
H	-3.505208	-0.845611	-0.555498
O	-0.995686	1.120801	-0.008024
Ag	1.161393	-0.000440	0.001882

AgOMe (Singlet)
E= -262.204783768 a.u.

O	-0.625702	-1.372983	0.000000
Ag	0.000000	0.684133	0.000000
C	0.447015	-2.254088	0.000000
H	1.115527	-2.169762	0.883194
H	0.092470	-3.306347	0.000000
H	1.115527	-2.169762	-0.883194

$Co(OAc)_2$ (Doublet, S**2 after annihilation: 0.7502)
E= -603.036752531 a.u.

O	1.612840	-1.082478	0.016381
C	2.297932	0.000020	0.018024
C	3.776519	-0.000317	-0.010916
H	4.110829	-0.000260	-1.053334
H	4.171678	0.896334	0.468546
H	4.171230	-0.897301	0.468297
O	1.613197	1.082663	0.016355
Co	0.000002	0.000116	0.000003
O	-1.612848	-1.082490	-0.016472
C	-2.297934	0.000011	-0.018045
C	-3.776516	-0.000307	0.011066
H	-4.110699	-0.000241	1.053526
H	-4.171712	0.896351	-0.468349
H	-4.171304	-0.897286	-0.468094
O	-1.613200	1.082657	-0.016446

$Co(OAc)_2^-$ (Singlet)
E= -603.123615488 a.u.

O	-1.660040	1.088959	0.021921
C	-2.329690	0.000858	0.021040
C	-3.819695	0.003335	-0.014954
H	-4.163373	-0.039774	-1.054593
H	-4.224402	-0.870132	0.500576
H	-4.219526	0.917009	0.428895
O	-1.663919	-1.090251	0.018589
Co	0.000006	-0.001608	-0.000357
O	1.660055	1.088925	-0.022234
C	2.329703	0.000814	-0.020968
C	3.819662	0.003317	0.016357
H	4.162406	-0.036719	1.056448
H	4.224750	-0.871604	-0.496323
H	4.219905	0.915890	-0.429423
O	1.663928	-1.090284	-0.018874

Co^{III}(OAc)$_2$OMe (Singlet)

E= -718.173227086 a.u.

Co	-0.118330	0.100246	-0.814002
C	-3.293136	-1.251659	0.715029
H	-3.074037	-2.161636	1.273469
H	-3.670122	-0.497378	1.414350
H	-4.069576	-1.433204	-0.030024
C	-2.080761	-0.717906	0.073434
O	-0.898866	-1.005361	0.505458
O	-2.094228	0.095703	-0.904642
C	3.864642	-0.564250	0.105359
H	4.071640	-1.109167	-0.817364
H	4.336413	0.419646	0.017694
H	4.315437	-1.081479	0.954100
C	2.389536	-0.376423	0.325776
O	1.883980	-0.376973	1.450097
O	1.721957	-0.209383	-0.791887
O	0.055192	1.674663	0.063762
C	-0.950711	2.084323	0.941258
H	-0.637041	3.055733	1.350827
H	-1.934448	2.223207	0.464668
H	-1.065054	1.403949	1.802887

Co^{II}(OAc)(OMe) (Doublet, S**2 after annihilation: 0.7549)

E= -489.618834714 a.u.

Co	0.742451	-0.892772	-0.000224
C	-2.877402	0.664717	-0.005928
H	-3.486460	0.229919	-0.801076
H	-3.376932	0.547189	0.956337
H	-2.783821	1.734950	-0.217381
C	-1.514095	0.054668	0.006260
O	-0.924401	-0.192546	1.107730
O	-0.921714	-0.197571	-1.094767
O	2.034547	0.421291	-0.009460
C	1.637833	1.757565	0.003118
H	1.054899	2.031999	0.901244
H	2.527805	2.407823	-0.004523
H	1.032861	2.041871	-0.877272

HOAc (Singlet)

E= -229.152320568 a.u.

C	1.379290	-0.159367	-0.002423
H	1.771499	0.055452	0.997562

H	1.610694	-1.194999	-0.255005
H	1.878264	0.520513	-0.697992
C	-0.085409	0.112166	-0.008032
O	-0.589039	1.223377	0.001129
O	-0.819356	-1.015855	-0.001084
H	-1.756588	-0.757947	0.017807

HOMe (Singlet)
E= -115.754293698 a.u.

H	-1.120214	-0.767141	-0.002084
O	-0.748689	0.122715	0.000372
C	0.659765	-0.018417	0.000209
H	1.032581	-0.537526	0.894373
H	1.030427	-0.553361	-0.885293
H	1.088130	0.986814	-0.011226

HOPiv (Singlet)
E= -347.114544083 a.u.

C	0.566502	-0.009940	-0.000252
C	-0.940126	0.179820	-0.001688
O	-1.600522	-0.992499	-0.000451
O	-1.519525	1.252648	-0.002354
C	0.962153	-0.788837	1.258608
H	0.513106	-1.785799	1.273829
H	2.050053	-0.906973	1.290030
H	0.655424	-0.261795	2.168305
C	0.966487	-0.805823	-1.247431
H	0.659496	-0.294023	-2.165695
H	2.054809	-0.920231	-1.275829
H	0.521402	-1.804635	-1.248693
C	1.243119	1.355090	-0.007653
H	2.330225	1.229646	-0.003485
H	0.972478	1.932805	-0.896567
H	0.966884	1.945022	0.871403
H	-2.552313	-0.797078	-0.000362

INT1 (Singlet)
E= -647.854472683 a.u.

C	4.159154	1.622989	0.142058
C	2.816121	0.993334	0.062597
C	2.515525	-0.318703	0.002367
C	3.537381	-1.412153	-0.004690
H	4.335518	2.282040	-0.717903
H	3.354643	-2.127737	0.804924

C	1.088175	-0.789160	-0.084603
N	0.166838	0.188143	0.006970
O	0.901155	-2.026132	-0.248722
C	-1.153474	-0.058210	0.011316
C	-1.855158	-1.279165	0.123967
C	-3.238025	-1.348900	0.160904
H	-1.252811	-2.175320	0.181607
C	-3.311981	1.025181	-0.032780
C	-3.983773	-0.174364	0.077212
H	-3.729794	-2.313277	0.254258
H	-3.802151	1.989203	-0.102995
H	-5.068244	-0.170667	0.094809
O	-1.402338	2.283091	-0.162602
N	-1.953271	1.108047	-0.063907
H	4.236518	2.268807	1.025987
H	4.982131	0.905089	0.182404
H	3.486975	-1.994388	-0.931790
H	4.558043	-1.038048	0.099912
H	1.969998	1.680210	0.047849

INT2 (Doublet, S**2 after annihilation: 0.7515)
E= -1250.96192931 a.u.

N	-0.888039	-0.601632	-0.493720
C	-2.207827	-0.342532	-0.265141
C	-3.328025	-1.067844	-0.707272
C	-4.614289	-0.636979	-0.438878
H	-3.148681	-1.972652	-1.271232
C	-3.712706	1.252108	0.705546
C	-4.811990	0.539950	0.286172
H	-5.461464	-1.213381	-0.796998
H	-3.754452	2.178186	1.264710
H	-5.801634	0.912100	0.524678
O	-1.435298	1.560115	0.864355
N	-2.450790	0.826776	0.436920
Co	0.377231	0.722256	0.224090
C	2.263628	3.879433	-0.966795
H	1.669470	4.541752	-1.599390
H	2.655857	4.428865	-0.109856
H	3.111145	3.527585	-1.564556
C	1.459664	2.702598	-0.535000
O	1.619248	2.198078	0.632228
O	0.628802	2.145159	-1.318155
C	2.151683	-1.654898	3.197663
H	2.876714	-2.443381	2.981265

H	2.583129	-1.024165	3.982252
H	1.233767	-2.097532	3.589654
C	1.901960	-0.815818	1.966751
O	2.845230	-0.559268	1.190541
O	0.689300	-0.402142	1.817137
C	2.965136	-0.918886	-2.354542
C	1.553642	-1.015356	-1.906975
C	0.991491	-1.971288	-1.140399
C	1.677018	-3.206313	-0.653040
H	3.605833	-1.709405	-1.955830
H	1.281789	-4.098624	-1.152051
C	-0.448167	-1.872405	-0.767384
O	-1.135772	-2.906825	-0.681002
H	3.043426	-0.938037	-3.448312
H	3.385456	0.046352	-2.040333
H	2.755658	-3.178658	-0.819020
H	1.500840	-3.353993	0.419356
H	0.910320	-0.193474	-2.222241

INT3 (Doublet, S**2 after annihilation: 0.7511)
E= -1021.76814410 a.u.

C	-2.956147	1.597341	-0.288330
C	-1.480244	1.414126	-0.183236
C	-0.600315	2.453599	-0.019213
C	-0.893678	3.919385	0.083803
H	-3.463546	1.081313	0.537533
H	-0.519787	4.341512	1.025835
C	0.808021	2.070118	0.072199
N	0.947466	0.686985	-0.038037
O	1.752318	2.864693	0.227802
C	2.101875	-0.014276	0.013996
C	3.429393	0.419110	0.171513
C	4.470794	-0.492066	0.195939
H	3.598892	1.482210	0.271791
C	2.905249	-2.273398	-0.090019
C	4.209487	-1.857876	0.063503
H	5.489852	-0.138024	0.318047
H	2.593683	-3.305020	-0.196783
H	4.998881	-2.600141	0.079249
O	0.638090	-1.824756	-0.259418
N	1.890578	-1.378427	-0.113896
Co	-0.639809	-0.288075	-0.261963
H	-1.963481	4.134955	0.029856
H	-0.401903	4.490044	-0.715020

H	-3.344185	1.127779	-1.201368
H	-3.292274	2.642586	-0.280777
C	-4.290090	-2.302474	-0.029089
H	-5.006925	-1.541286	-0.358279
H	-4.722723	-2.821943	0.828172
H	-4.161850	-3.001714	-0.858621
C	-2.993442	-1.622762	0.336494
O	-2.222019	-1.349281	-0.657970
O	-2.751078	-1.333047	1.526027

INT6 (Doublet, S**2 after annihilation: 0.7517)
E= -1513.20985897 a.u.

C	2.044061	-0.916827	-2.971083
C	0.741339	-0.866998	-2.263260
C	0.157869	-1.838884	-1.527411
C	0.708400	-3.217965	-1.348172
H	2.636310	-1.806307	-2.740836
H	0.156045	-3.942773	-1.957882
C	-1.185715	-1.613736	-0.930114
N	-1.537414	-0.299418	-0.706108
O	-1.901961	-2.595897	-0.662867
C	-2.837519	0.054006	-0.510571
C	-4.003099	-0.665441	-0.835271
C	-5.258921	-0.112359	-0.669905
H	-3.883972	-1.664465	-1.230160
C	-4.245716	1.884506	0.147582
C	-5.386770	1.184377	-0.165941
H	-6.138569	-0.690326	-0.934976
H	-4.229501	2.895084	0.535804
H	-6.351702	1.654431	-0.017242
O	-1.953179	2.071291	0.289822
N	-3.014499	1.336910	-0.016692
Co	-0.234567	0.913425	0.189167
H	1.898691	-0.881695	-4.058353
H	2.642756	-0.033363	-2.717142
H	1.761659	-3.281779	-1.630616
H	0.609874	-3.550370	-0.310016
H	0.192060	0.066036	-2.366952
C	2.458604	3.594328	-0.542832
H	2.800110	4.049935	0.387240
H	3.298061	3.073872	-1.015144
H	2.122729	4.368015	-1.237384
C	1.370766	2.606908	-0.306436
O	1.082910	2.210598	0.880425

O	0.714944	2.107762	-1.271104
C	-1.420136	-1.939542	3.042198
H	-1.880947	-1.279645	3.779065
H	-2.222016	-2.345364	2.414296
H	-0.926582	-2.775529	3.540366
C	-0.477460	-1.188399	2.145220
O	0.488532	-1.830102	1.637502
O	-0.751561	0.033810	1.924315
O	3.740294	0.297987	-0.325719
C	4.443408	0.997807	0.647193
H	3.827546	1.713053	1.231875
H	4.940631	0.351654	1.400708
H	5.255775	1.604592	0.197179
Ag	2.078154	-0.757112	0.498640

INT7 (Doublet, S**2 after annihilation: 0.7539)
E= -1137.54204656 a.u.

C	2.358979	2.407737	1.929305
C	1.095489	1.946650	1.299614
C	0.421645	2.523474	0.284046
C	0.834733	3.775425	-0.419262
H	2.798412	3.281588	1.441420
H	1.793028	4.159419	-0.065112
C	-0.889655	1.969261	-0.152235
N	-1.031888	0.606127	-0.036910
O	-1.767798	2.738066	-0.583118
C	-2.243898	0.007087	0.039458
C	-3.531544	0.564799	0.169238
C	-4.641468	-0.240959	0.329744
H	-3.618908	1.641799	0.140798
C	-3.245407	-2.173093	0.233529
C	-4.503352	-1.633074	0.357964
H	-5.621977	0.213533	0.431854
H	-3.021024	-3.232211	0.247321
H	-5.352830	-2.295481	0.473203
O	-0.955294	-1.954983	-0.011652
N	-2.162412	-1.373784	0.083656
Co	0.446923	-0.657766	-0.359442
H	2.202164	2.656652	2.986471
H	3.104973	1.604156	1.918886
H	0.922459	3.597193	-1.498349
H	0.085847	4.565351	-0.299094
H	0.664241	1.039064	1.725080
C	3.869874	-2.946249	0.347132

H	4.191311	-3.231173	1.352168
H	3.587405	-3.838123	-0.215215
H	4.734926	-2.491445	-0.148161
C	2.756813	-1.929561	0.415052
O	1.794480	-2.092423	-0.424098
O	2.828995	-0.992355	1.239185
O	1.250028	0.410952	-1.714805
C	2.628332	0.533304	-1.784378
H	3.171600	-0.429589	-1.842414
H	2.919884	1.104488	-2.685898
H	3.065139	1.085192	-0.924593

INT8 (Doublet, S**2 after annihilation: 0.7503)
E= -1137.51861947 a.u.

C	2.703721	2.546252	1.201580
C	1.501991	1.807693	0.666827
C	0.456005	2.660878	0.102173
C	0.770348	4.073717	-0.284668
H	3.204366	3.136632	0.427808
H	-0.115403	4.531197	-0.734536
C	-0.790008	2.128576	-0.237860
N	-0.908395	0.710512	-0.062425
O	-1.768623	2.774606	-0.733581
C	-2.116079	0.144760	0.071843
C	-3.347931	0.716998	0.480207
C	-4.473328	-0.060851	0.640264
H	-3.365214	1.780734	0.673093
C	-3.232732	-1.997479	0.005396
C	-4.426719	-1.443998	0.398703
H	-5.399757	0.401917	0.968247
H	-3.073639	-3.048517	-0.203067
H	-5.293760	-2.081755	0.520913
O	-0.991966	-1.801708	-0.582087
N	-2.137215	-1.219806	-0.160250
Co	0.466990	-0.583907	-0.492902
H	2.367287	3.243786	1.974882
H	3.438910	1.882074	1.661607
H	1.067965	4.713321	0.560791
H	1.586277	4.158975	-1.021034
H	1.129981	1.094744	1.417319
C	3.048089	-3.602319	0.324788
H	2.893657	-4.230552	1.204299
H	3.030285	-4.214352	-0.578873
H	4.047740	-3.160677	0.408442

C	2.036918	-2.492476	0.271718
O	1.693519	-2.069950	-0.894597
O	1.597154	-1.978191	1.327028
O	1.944434	0.797514	-0.469479
C	3.284229	0.316242	-0.383753
H	3.429315	-0.401480	-1.192407
H	3.992816	1.140146	-0.515882
H	3.486542	-0.181788	0.573894

INT10 (Doublet, S**2 after annihilation: 0.7501)
E= -1513.18705243 a.u.

N	0.256744	0.497886	-0.858569
C	0.805022	1.697822	-0.717548
C	2.150951	2.098486	-0.919161
C	2.550280	3.389864	-0.645418
H	2.839871	1.367132	-1.333269
C	0.316012	3.943678	0.002836
C	1.624937	4.332346	-0.168890
H	3.583922	3.678269	-0.813300
H	-0.479759	4.588850	0.355340
H	1.906578	5.353788	0.055254
O	-1.361756	2.325846	-0.102415
N	-0.068009	2.677192	-0.273360
Co	-1.662254	0.492572	-0.609444
C	-5.529494	0.989007	0.838767
H	-5.784279	1.545017	1.743430
H	-5.963446	1.483035	-0.033155
H	-5.989969	-0.001520	0.922043
C	-4.036433	0.820700	0.712266
O	-3.588100	0.749476	-0.495975
O	-3.325683	0.718807	1.731607
C	-2.608802	-3.517137	-1.519492
H	-3.675639	-3.657064	-1.722399
H	-2.073398	-3.516810	-2.470862
H	-2.285475	-4.372609	-0.919854
C	-2.415302	-2.236196	-0.742546
O	-2.782670	-2.176208	0.446449
O	-1.873358	-1.268442	-1.404133
C	0.482118	-2.783054	1.854503
C	0.487117	-1.523195	1.013989
C	1.296389	-1.617872	-0.263488
C	1.605805	-2.995541	-0.806583
H	0.007133	-3.617180	1.327001
H	2.225291	-2.919965	-1.703546

C	1.056150	-0.610258	-1.269051
O	1.480072	-0.649889	-2.448083
H	1.500559	-3.085038	2.126442
H	-0.081409	-2.616747	2.778643
H	2.133524	-3.635535	-0.094048
H	0.688484	-3.532842	-1.100386
H	-0.557439	-1.255004	0.792311
Ag	3.258837	-0.649240	0.439027
O	1.013000	-0.428839	1.824378
C	-0.001460	0.276031	2.513714
H	-0.618269	-0.383230	3.144138
H	0.490975	1.008630	3.159646
H	-0.664670	0.804052	1.812741

INT11 (Doublet, S**2 after annihilation: 0.7504)
E= -647.686770671 a.u.

C	-3.734552	1.625481	1.126716
C	-2.551212	1.033599	0.464593
C	-2.441090	-0.198520	-0.076333
C	-3.520509	-1.229194	-0.125535
H	-4.593181	0.952948	1.175865
H	-3.194887	-2.159941	0.351322
C	-1.157009	-0.627276	-0.659286
N	-0.115569	0.294684	-0.648975
O	-1.025086	-1.731080	-1.202239
C	1.054626	-0.027600	-0.176071
C	1.403723	-1.181939	0.590191
C	2.667103	-1.361573	1.076822
H	0.619218	-1.907494	0.773901
C	3.358522	0.722538	0.077651
C	3.660522	-0.390955	0.814980
H	2.909214	-2.241251	1.663315
H	4.058033	1.511446	-0.167023
H	4.670895	-0.505519	1.190835
O	1.850559	1.963181	-1.113994
N	2.099941	0.924739	-0.423918
H	-3.484213	1.937470	2.147792
H	-4.044470	2.540687	0.607922
H	-4.435480	-0.897636	0.367538
H	-3.766655	-1.488016	-1.160942
H	-1.673615	1.677163	0.407231

INT12 (Triplet, S**2 after annihilation: 2.0008)
E= -1250.81067035 a.u.

N	-0.998071	-0.684741	-0.325570
C	-2.314249	-0.389162	-0.123045
C	-3.447397	-1.187561	-0.351095
C	-4.711291	-0.647831	-0.210108
H	-3.302011	-2.212189	-0.661874
C	-3.759838	1.456441	0.403111
C	-4.873728	0.691278	0.167240
H	-5.579128	-1.267866	-0.407471
H	-3.761868	2.494412	0.707939
H	-5.853592	1.139215	0.281331
O	-1.457482	1.668964	0.554346
N	-2.530085	0.904409	0.271646
Co	0.172578	0.771654	0.167471
C	2.526676	3.570894	-1.035867
H	2.343432	3.868755	-2.069114
H	2.493317	4.438032	-0.374450
H	3.533020	3.141129	-0.976456
C	1.557098	2.539125	-0.607066
O	1.249771	2.372737	0.624325
O	1.016824	1.731227	-1.434421
C	2.910586	-1.038859	2.761743
H	3.713875	-1.720888	2.472880
H	3.353657	-0.256623	3.386421
H	2.167020	-1.570758	3.357542
C	2.320543	-0.394802	1.534220
O	3.037126	-0.026641	0.595639
O	1.023624	-0.268222	1.573757
C	2.639384	-1.501761	-2.429088
C	1.300219	-1.450337	-1.797946
C	0.854741	-2.191147	-0.761988
C	1.619461	-3.268913	-0.064343
H	3.343034	-2.153625	-1.907111
H	1.258009	-4.263665	-0.347705
C	-0.529108	-1.999690	-0.280303
O	-1.202338	-2.935646	0.160103
H	2.570588	-1.838986	-3.470164
H	3.070012	-0.492821	-2.469065
H	2.687959	-3.226064	-0.286385
H	1.493053	-3.190265	1.021789
H	0.608331	-0.725542	-2.225157

INT13 (Triplet, S**2 after annihilation: 2.0006)
E= -1137.40873766 a.u.

C	2.433319	2.316174	2.061753

C	1.187483	1.938344	1.353047
C	0.635991	2.538144	0.278955
C	1.167000	3.752108	-0.409899
H	3.003150	3.101006	1.559471
H	2.092575	4.117088	0.038507
C	-0.657395	2.039067	-0.239833
N	-0.886150	0.671480	-0.101627
O	-1.473821	2.804459	-0.765008
C	-2.135474	0.156131	0.002816
C	-3.381583	0.800136	0.132195
C	-4.528430	0.060661	0.334780
H	-3.409745	1.878705	0.069813
C	-3.256250	-1.957548	0.277203
C	-4.473404	-1.337364	0.406783
H	-5.479849	0.573016	0.436302
H	-3.087780	-3.026013	0.318046
H	-5.359950	-1.940392	0.559264
O	-0.959988	-1.864155	-0.004257
N	-2.140368	-1.215684	0.082975
Co	0.446783	-0.713210	-0.504046
H	2.217490	2.655074	3.082745
H	3.081234	1.438308	2.172632
H	1.363810	3.541152	-1.468325
H	0.434784	4.565934	-0.389067
H	0.664064	1.073385	1.759139
C	3.334352	-3.044102	0.779193
H	3.504534	-3.034187	1.856086
H	3.142966	-4.061787	0.434519
H	4.246304	-2.695014	0.282699
C	2.216768	-2.133904	0.404681
O	1.631038	-2.295488	-0.728001
O	1.850535	-1.177366	1.141700
O	1.496068	0.348319	-1.503608
C	2.880358	0.185507	-1.587872
H	3.171782	-0.792910	-1.996573
H	3.278726	0.962023	-2.256272
H	3.371329	0.304441	-0.608587

INT14 (Doublet, S**2 after annihilation: 0.7558)
E= -865.287000869 a.u.

Co	0.260726	-1.425428	-0.553208
C	-3.285352	-1.484229	0.908643
H	-3.385083	-2.053568	1.834721
H	-3.506341	-0.436324	1.137734

H	-3.997977	-1.839063	0.163528
C	-1.896158	-1.553030	0.397490
O	-0.884450	-1.471533	1.166366
O	-1.654615	-1.657256	-0.860626
C	4.236770	-0.333594	0.317803
H	4.660141	-1.190394	-0.208091
H	4.767866	0.578699	0.042915
H	4.384927	-0.495621	1.390639
C	2.767719	-0.202145	0.055672
O	2.249723	0.945534	0.124234
O	2.136746	-1.286795	-0.194807
O	-1.948794	1.721349	0.286529
C	-2.675220	1.726804	-0.899112
H	-2.358798	2.503982	-1.623275
H	-2.645613	0.767543	-1.453460
H	-3.746566	1.922417	-0.696801
Ag	0.100264	1.357355	0.116854

INT15 (Doublet, S**2 after annihilation: 0.7529)
E= -865.293377990 a.u.

Co	-0.667006	0.779949	0.618330
C	-4.202966	0.374093	-1.115730
H	-4.272007	0.741220	-2.141302
H	-4.471472	-0.687358	-1.130036
H	-4.920897	0.894646	-0.480191
C	-2.805520	0.508891	-0.601656
O	-1.814984	0.355539	-1.364237
O	-2.627683	0.757846	0.648414
C	3.122688	2.475877	-0.195767
H	3.427571	2.708240	0.827299
H	4.003267	2.271471	-0.805613
H	2.621383	3.366583	-0.586762
C	2.161359	1.320276	-0.209778
O	2.354721	0.378980	-1.021745
O	1.193957	1.397229	0.626853
O	-0.577093	-1.039678	1.205052
C	-0.046742	-1.147207	2.501215
H	-0.029027	-2.203499	2.809695
H	0.986088	-0.766602	2.583984
H	-0.658878	-0.603776	3.241552
Ag	0.930189	-1.335541	-0.511445

INT16 (Singlet)
E= -1250.81583436 a.u.

C	2.990732	-1.487196	-2.503614
C	1.667583	-1.430090	-1.838795
C	1.204724	-2.225896	-0.854752
C	1.930791	-3.385189	-0.255234
H	3.654586	-2.254649	-2.098949
H	1.394138	-4.322902	-0.433999
C	-0.170690	-2.020688	-0.348410
N	-0.644479	-0.714212	-0.384365
O	-0.841723	-2.970542	0.076430
C	-1.977776	-0.438173	-0.352160
C	-3.083586	-1.285301	-0.548331
C	-4.365278	-0.769863	-0.559199
H	-2.899326	-2.340721	-0.688937
C	-3.496869	1.419154	-0.183528
C	-4.582708	0.598548	-0.368372
H	-5.205402	-1.439553	-0.712440
H	-3.537732	2.490426	-0.034276
H	-5.576571	1.028752	-0.359435
O	-1.212641	1.755573	-0.066714
N	-2.249406	0.896852	-0.189269
Co	0.358721	0.801180	0.321596
H	2.884422	-1.662170	-3.581026
H	3.496404	-0.517339	-2.409528
H	2.941193	-3.493955	-0.652520
H	2.005241	-3.276441	0.833634
H	0.999385	-0.647963	-2.194022
C	2.422229	3.701466	-1.033129
H	1.732144	4.418359	-1.488667
H	2.963968	4.205290	-0.232296
H	3.112589	3.365076	-1.808635
C	1.645105	2.560155	-0.504911
O	1.247973	1.603372	-1.254218
O	1.304739	2.474059	0.725058
C	1.463044	-1.096907	3.423167
H	1.223856	-0.521830	4.320060
H	0.886387	-2.026432	3.467739
H	2.525905	-1.336731	3.397934
C	1.060631	-0.348763	2.214446
O	1.804248	-0.246332	1.182884
O	-0.087230	0.218232	2.122140

INT17 (Singlet)

E= -1021.62395461 a.u.

C	-3.319153	0.926803	-0.622511

C	-1.869091	1.077974	-0.365636
C	-1.218361	2.210940	-0.007539
C	-1.808033	3.567283	0.219017
H	-3.778151	0.243957	0.104177
H	-1.621169	3.912012	1.243013
C	0.238890	2.089255	0.165228
N	0.658695	0.794965	-0.107914
O	0.991206	3.019383	0.479509
C	1.915888	0.304120	0.010299
C	3.094271	0.938217	0.435969
C	4.284549	0.236467	0.475392
H	3.032799	1.978315	0.724639
C	3.160217	-1.716905	-0.320600
C	4.322469	-1.107954	0.093557
H	5.188694	0.736179	0.808460
H	3.077902	-2.749081	-0.636948
H	5.238459	-1.685650	0.115761
O	0.890345	-1.637990	-0.792128
N	1.999121	-1.022632	-0.362001
Co	-0.688499	-0.439998	-0.502301
H	-2.887055	3.581956	0.053102
H	-1.354511	4.313427	-0.443969
H	-3.502879	0.480519	-1.607090
H	-3.865406	1.876615	-0.574442
C	-2.976280	-2.856999	1.293650
H	-3.978910	-2.424281	1.372105
H	-2.595199	-3.031362	2.300041
H	-3.062685	-3.798875	0.749097
C	-2.107172	-1.920628	0.551522
O	-2.092327	-1.831320	-0.716046
O	-1.316261	-1.106518	1.162853

INT18 (Doublet, S**2 after annihilation: 0.7502)
E= -1136.80651738 a.u.

C	-3.090647	2.763320	-0.166415
C	-1.765659	2.116863	0.001683
C	-0.557067	2.705021	-0.179924
C	-0.470888	4.144175	-0.611795
H	-3.616252	2.858755	0.789719
H	-0.489201	4.836441	0.238345
C	0.770471	2.109154	0.125789
N	0.918337	0.747726	0.017030
O	1.705260	2.882120	0.408168
C	2.140478	0.139137	0.093052

C	3.400126	0.644186	0.462076
C	4.522668	-0.163409	0.436150
H	3.457968	1.677012	0.768815
C	3.186659	-1.990373	-0.310205
C	4.421411	-1.499159	0.040860
H	5.481644	0.248769	0.733682
H	2.985756	-3.007887	-0.620213
H	5.279049	-2.160237	0.012271
O	0.916066	-1.737836	-0.630868
N	2.095953	-1.190206	-0.277465
Co	-0.481622	-0.514560	-0.317689
H	-3.000223	3.763915	-0.583344
H	-3.732198	2.169040	-0.825777
H	0.466372	4.325901	-1.142045
H	-1.286094	4.425887	-1.280194
O	-1.813048	0.785588	0.390038
C	-2.985241	0.291081	1.073806
H	-2.685389	-0.667241	1.495843
H	-3.254430	0.974690	1.880186
H	-3.818457	0.157789	0.379852
C	-3.138461	-3.667583	-0.216795
H	-3.240671	-3.836663	-1.290230
H	-2.960300	-4.614912	0.295669
H	-4.091805	-3.267928	0.146828
C	-2.053667	-2.674207	0.093488
O	-1.443451	-2.709171	1.183456
O	-1.848964	-1.770961	-0.806966

INT19 (Doublet, S**2 after annihilation: 0.7501)
E= -1137.19468308 a.u.

C	-3.467381	1.019171	-0.700534
C	-2.015309	1.191936	-0.951164
C	-1.210075	2.101661	-0.297418
C	-1.660209	3.064318	0.726754
H	-3.712634	1.055367	0.364495
H	-2.651823	2.832455	1.115959
C	0.251529	2.114941	-0.622258
N	0.691020	0.820117	-0.472186
O	0.929033	3.100070	-0.867933
C	1.963922	0.349153	-0.374110
C	3.160012	0.924644	-0.808600
C	4.338963	0.218841	-0.651143
H	3.126581	1.904945	-1.266173
C	3.136838	-1.599241	0.356600

C	4.326235	-1.052944	-0.070234
H	5.273078	0.655187	-0.987270
H	3.022490	-2.565406	0.830194
H	5.235764	-1.626027	0.062100
O	0.850342	-1.411315	0.683889
N	1.995866	-0.890048	0.209733
Co	-0.629151	-0.273316	0.240758
H	-4.032741	1.818672	-1.195346
H	-3.824136	0.072749	-1.112169
H	-0.944090	3.144415	1.549435
H	-1.703642	4.056173	0.257407
H	-1.605561	0.723615	-1.845009
C	-2.507724	-3.262321	-1.251808
H	-2.057852	-3.595487	-2.186738
H	-2.501915	-4.070728	-0.517418
H	-3.552254	-2.990725	-1.432036
C	-1.791462	-2.092628	-0.709842
O	-1.998071	-1.660626	0.478618
O	-0.933784	-1.428079	-1.383513
O	-0.828608	0.432645	1.877975
C	-0.392628	-0.340991	2.956446
H	0.705012	-0.410991	2.980741
H	-0.748582	0.146497	3.871965
H	-0.796392	-1.362472	2.918156

INT20 (Doublet, S**2 after annihilation: 0.8063)
E= -1137.20622376 a.u.

C	3.549895	1.586616	0.900554
C	2.139193	1.206143	0.540758
C	1.269082	2.349578	0.124603
C	1.831401	3.703357	-0.042783
H	4.083557	2.061149	0.073680
H	2.692684	3.689921	-0.721650
C	-0.161028	2.174691	-0.073131
N	-0.640003	0.861674	-0.059454
O	-0.914995	3.143226	-0.251829
C	-1.978876	0.615460	0.049374
C	-3.008256	1.430448	0.552656
C	-4.295160	0.939794	0.638437
H	-2.765834	2.432397	0.874424
C	-3.579265	-1.160031	-0.248792
C	-4.591577	-0.366157	0.228072
H	-5.079241	1.574602	1.037488
H	-3.681067	-2.184323	-0.583324

H	-5.594456	-0.770732	0.285444
O	-1.361410	-1.439687	-0.841390
N	-2.325957	-0.654666	-0.333639
Co	0.324053	-0.723185	-0.526864
H	3.507647	2.299075	1.728461
H	4.119152	0.723514	1.248244
H	1.083420	4.396613	-0.425950
H	2.211123	4.097948	0.909110
H	1.681349	0.666696	1.382105
C	1.531992	-3.913281	1.150293
H	1.054807	-4.031559	2.123404
H	1.338534	-4.780816	0.518022
H	2.613627	-3.841600	1.304951
C	1.068678	-2.682034	0.484852
O	1.088584	-2.522293	-0.779930
O	0.642767	-1.666830	1.150228
O	2.062921	0.245609	-0.610569
C	3.233483	-0.560826	-0.854999
H	2.994621	-1.178841	-1.718334
H	4.074863	0.089828	-1.097634
H	3.477198	-1.189683	0.007098

INT21 (Doublet, S**2 after annihilation: 0.7502)
E= -1365.93720116 a.u.

C	3.076058	2.275254	0.825245
C	1.757604	1.689280	0.401891
C	0.906662	2.546928	-0.474395
C	1.456529	3.737926	-1.150960
H	3.704818	2.580130	-0.014408
H	2.315343	3.466004	-1.780257
C	-0.486775	2.179130	-0.682251
N	-0.858932	0.928521	-0.204797
O	-1.285661	2.956136	-1.238800
C	-2.172736	0.601818	-0.041582
C	-3.289279	1.446817	0.100723
C	-4.541265	0.919437	0.347583
H	-3.136048	2.512946	0.018657
C	-3.621727	-1.280604	0.326655
C	-4.717128	-0.463516	0.460327
H	-5.387158	1.589479	0.461354
H	-3.633180	-2.360506	0.396965
H	-5.685471	-0.908581	0.652925
O	-1.375813	-1.602729	-0.083821
N	-2.404951	-0.744469	0.073584

Co	0.250982	-0.672336	-0.158188
H	2.872743	3.162369	1.431168
H	3.629936	1.572211	1.451880
H	0.703074	4.220371	-1.774276
H	1.837399	4.474485	-0.431637
H	1.190060	1.397363	1.292091
C	0.968930	-0.838705	3.951144
H	-0.017001	-1.215669	4.233056
H	1.741165	-1.357507	4.520678
H	0.993469	0.224153	4.212143
C	1.210216	-0.993035	2.471339
O	2.276883	-1.427565	2.025006
O	0.196736	-0.591527	1.755601
O	1.890809	0.421666	-0.376247
C	3.200512	-0.099130	-0.637486
H	3.068734	-0.945766	-1.309992
H	3.795100	0.659327	-1.151851
H	3.696934	-0.432623	0.276031
C	1.254473	-3.161379	-2.880935
H	1.099096	-4.182531	-2.528472
H	0.692275	-2.984127	-3.798110
H	2.321051	-3.045152	-3.099317
C	0.871752	-2.189364	-1.832542
O	0.328005	-1.067521	-2.111392
O	1.097215	-2.411810	-0.592547

INT22 (Triplet, S**2 after annihilation: 2.0274)
E= -1137.41039918 a.u.

C	2.547314	2.798083	1.310781
C	1.532447	1.869304	0.684099
C	0.496335	2.585929	-0.126369
C	0.824391	3.876504	-0.773781
H	3.137629	3.330070	0.559233
H	1.783764	3.817824	-1.303131
C	-0.847016	2.037565	-0.290065
N	-0.963484	0.695707	0.007901
O	-1.777568	2.762717	-0.702973
C	-2.199841	0.137546	0.110394
C	-3.406355	0.754252	0.487271
C	-4.569291	0.022168	0.631629
H	-3.390953	1.818891	0.674471
C	-3.373155	-1.954509	0.037934
C	-4.557273	-1.354781	0.397285
H	-5.484836	0.521472	0.933068

H	-3.250479	-3.012000	-0.158198
H	-5.447625	-1.964533	0.494213
O	-1.125084	-1.865188	-0.452672
N	-2.237214	-1.227271	-0.104157
Co	0.536112	-0.662956	-0.444393
H	2.022195	3.541240	1.917298
H	3.229516	2.260236	1.972594
H	0.044160	4.169322	-1.478192
H	0.933042	4.689493	-0.042066
H	1.046423	1.273795	1.472996
C	3.101450	-3.614073	0.315847
H	2.842045	-4.176061	1.214484
H	3.179432	-4.287183	-0.539657
H	4.088666	-3.166352	0.476493
C	2.118214	-2.518480	0.053381
O	1.939659	-2.100488	-1.137940
O	1.492642	-1.971444	1.013802
O	2.143437	0.897008	-0.243568
C	3.424064	0.400622	0.154655
H	3.687884	-0.366428	-0.574124
H	4.179787	1.190860	0.123812
H	3.396826	-0.041509	1.157554

INT23 (Triplet, S**2 after annihilation: 2.0125)
E= -1399.62138994 a.u.

N	-0.223032	0.639192	-0.086540
C	-0.135334	1.996242	-0.068777
C	1.011725	2.801789	0.034616
C	0.903332	4.170777	0.174425
H	1.978293	2.313175	0.022052
C	-1.478752	3.977802	0.101949
C	-0.360955	4.769019	0.206331
H	1.799358	4.775950	0.263114
H	-2.498248	4.339467	0.111500
H	-0.484761	5.840454	0.309014
O	-2.481533	1.922700	-0.159340
N	-1.363189	2.633264	-0.042762
Co	-2.139290	-0.027846	-0.394396
C	-5.485996	-1.451984	1.493405
H	-5.232241	-1.407153	2.554293
H	-6.462947	-0.997945	1.323102
H	-5.550646	-2.511498	1.220856
C	-4.426459	-0.803899	0.648772
O	-4.721833	-0.179871	-0.395335

O	-3.205601	-0.960625	1.033610
O	-2.061907	-1.128903	-1.940668
C	0.003276	-3.346598	1.472497
C	0.071913	-1.971350	0.929639
C	0.718278	-1.586518	-0.190335
C	1.477829	-2.530151	-1.073869
H	-1.014995	-3.589335	1.796624
H	1.716865	-2.067341	-2.037659
C	0.812364	-0.146553	-0.514250
O	1.785478	0.295301	-1.175810
H	0.336687	-4.108850	0.762759
H	0.632582	-3.430424	2.369754
H	2.426795	-2.849616	-0.612624
H	0.899111	-3.442935	-1.268698
H	-0.413395	-1.196165	1.523451
Ag	3.840454	-0.389422	-0.325306
O	3.329882	-0.972486	1.740771
C	2.609023	-0.015883	2.416970
H	2.237317	-0.410856	3.382258
H	3.166021	0.917760	2.626130
H	1.685046	0.286899	1.875963
C	-2.447070	-2.453844	-1.787621
H	-1.939751	-2.962886	-0.943464
H	-3.534842	-2.583956	-1.633226
H	-2.178135	-3.029273	-2.690340

INT24 (Triplet, S**2 after annihilation: 2.0324)
E= -1399.63216355 a.u.

N	0.000122	0.690691	-0.465742
C	0.053763	2.039502	-0.277350
C	1.183056	2.825212	0.002415
C	1.066415	4.173685	0.282765
H	2.148151	2.332200	0.002403
C	-1.300629	3.993482	0.014565
C	-0.197176	4.769030	0.282462
H	1.952014	4.759244	0.507261
H	-2.319981	4.356711	-0.002237
H	-0.336518	5.822967	0.492552
O	-2.289070	1.982394	-0.507199
N	-1.179319	2.669524	-0.259847
Co	-1.941912	-0.030411	-0.587665
C	-4.567713	-1.274641	2.390608
H	-4.209757	-0.739557	3.275220
H	-5.650130	-1.159285	2.316861

H	-4.329955	-2.331081	2.547105
C	-3.865839	-0.764417	1.159251
O	-4.514590	-0.286120	0.202892
O	-2.580300	-0.852257	1.164159
O	-2.304531	-1.035220	-2.191770
C	0.602620	-3.559485	0.458402
C	0.596518	-2.051494	0.399739
C	0.977867	-1.452052	-0.918904
C	1.642243	-2.261496	-1.970596
H	-0.116075	-3.983643	-0.249539
H	1.836939	-1.666589	-2.865007
C	1.046902	0.014123	-0.998921
O	2.062076	0.546853	-1.541031
H	1.592530	-3.961458	0.222046
H	0.325927	-3.897084	1.461241
H	2.605184	-2.673067	-1.631070
H	1.028009	-3.125805	-2.251130
H	-0.380456	-1.667388	0.721309
Ag	3.421207	-0.257720	0.364819
O	1.581731	-1.550746	1.371899
C	0.985381	-1.033118	2.555087
H	0.347330	-1.785290	3.036736
H	1.797886	-0.769087	3.235672
H	0.382590	-0.138693	2.345701
C	-2.408231	-2.402619	-1.979771
H	-1.762321	-2.779305	-1.158538
H	-3.436760	-2.730461	-1.720965
H	-2.118905	-2.984429	-2.876143

TS1 (Singlet)
E= -994.984458343 a.u.
Imaginary Frequency -- -455.3372

C	-2.244689	3.081348	-0.993180
C	-0.949745	2.384332	-0.785551
C	0.081015	2.766584	-0.009445
C	0.147233	4.052677	0.751892
H	-2.340903	3.439345	-2.026359
H	0.394564	3.881452	1.806043
C	1.327908	1.944666	0.058861
N	1.164249	0.605307	-0.127273
O	2.411804	2.533197	0.267936
C	2.222979	-0.257722	-0.026582
C	3.420863	-0.084351	0.682061
C	4.384840	-1.078226	0.749266

H	3.565432	0.861643	1.184062
C	2.973956	-2.465019	-0.582444
C	4.156060	-2.289861	0.101793
H	5.298689	-0.909882	1.310555
H	2.702540	-3.368639	-1.113901
H	4.875663	-3.101077	0.121264
O	0.938546	-1.713696	-1.330494
N	2.024570	-1.487443	-0.659270
H	-3.079747	2.383562	-0.845675
H	-2.397236	3.936014	-0.329385
H	0.940404	4.695752	0.355565
H	-0.789999	4.610934	0.711059
H	-0.839548	1.454024	-1.339077
C	-3.157680	-1.425509	0.473161
C	-1.962818	-0.758184	-0.217046
O	-1.008368	-0.415741	0.606167
O	-1.921724	-0.604932	-1.443225
H	-0.067731	0.034708	0.125732
C	-2.702266	-2.821756	0.913405
H	-1.880802	-2.761183	1.634285
H	-3.529595	-3.361372	1.387792
H	-2.359704	-3.417171	0.059469
C	-3.579155	-0.614984	1.699117
H	-3.853907	0.411079	1.429589
H	-4.450531	-1.075959	2.177857
H	-2.777636	-0.562917	2.440730
C	-4.321632	-1.542073	-0.503356
H	-5.172457	-2.038268	-0.023921
H	-4.656454	-0.556687	-0.846147
H	-4.046061	-2.122560	-1.388528

TS2 (Doublet, S**2 after annihilation: 0.7504)
E= -1250.91353677 a.u.
Imaginary Frequency -- -1272.3713

C	-2.840891	1.648194	0.075726
C	-1.388327	1.425976	-0.243752
C	-0.500045	2.458649	-0.110073
C	-0.867862	3.857266	0.260814
H	-2.970878	2.041365	1.091452
H	-0.822526	4.018164	1.346997
C	0.939118	2.152329	-0.202004
N	1.143286	0.791550	-0.248585
O	1.835946	3.015072	-0.182233
C	2.342033	0.162496	-0.251774

C	3.643063	0.690262	-0.195943
C	4.743065	-0.148042	-0.209952
H	3.743704	1.765238	-0.141176
C	3.287800	-2.038887	-0.337637
C	4.567274	-1.532257	-0.283435
H	5.741075	0.275998	-0.160750
H	3.040680	-3.091554	-0.391970
H	5.404863	-2.219294	-0.295303
O	0.989130	-1.753588	-0.383901
N	2.216354	-1.213225	-0.318871
Co	-0.377205	-0.338752	-0.258340
H	-3.301840	2.377872	-0.604953
H	-3.422719	0.724682	0.003109
H	-0.171149	4.574009	-0.184895
H	-1.883147	4.110177	-0.057123
H	-1.518532	0.622909	-1.415722
C	-3.491029	-1.923165	-2.519223
H	-3.367686	-1.879766	-3.603186
H	-3.473490	-2.957783	-2.177195
H	-4.471317	-1.493281	-2.288658
C	-2.443149	-1.111709	-1.840231
O	-1.881802	-1.539965	-0.794547
O	-2.165655	0.040946	-2.344942
C	-1.228314	-1.181635	4.028794
H	-2.164016	-1.232906	4.590032
H	-0.710669	-2.141119	4.132430
H	-0.578141	-0.424576	4.476933
C	-1.467303	-0.889685	2.557903
O	-2.632847	-0.950001	2.104422
O	-0.417240	-0.611322	1.882712

TS3 (Doublet, S**2 after annihilation: 0.7504)
E= -1283.96339894 a.u.
Imaginary Frequency -- -270.9813

N	-0.775083	-0.811636	0.442183
C	-1.942534	-1.420401	0.189239
C	-2.391555	-2.720155	0.512107
C	-3.648437	-3.152687	0.138830
H	-1.712872	-3.353077	1.067456
C	-4.060560	-1.038694	-0.893057
C	-4.503384	-2.302595	-0.574688
H	-3.968748	-4.156885	0.401123
H	-4.637861	-0.304499	-1.442078
H	-5.496265	-2.607674	-0.883250

O	-2.446565	0.626431	-0.864934
N	-2.828143	-0.614806	-0.527253
Co	-0.600997	0.983912	-0.136295
C	2.032221	2.005845	0.933025
C	1.172748	0.856097	0.523895
C	1.427937	-0.432637	1.163360
C	2.434598	-0.561782	2.286889
H	1.808995	2.254706	1.979995
H	2.499329	-1.599699	2.628786
C	0.270142	-1.314941	1.223463
O	0.184433	-2.369241	1.898409
H	3.110801	1.791686	0.883811
H	1.834540	2.907111	0.344320
H	3.444928	-0.247462	2.005545
H	2.154788	0.041664	3.166980
Ag	2.460619	-1.570570	-0.633652
O	1.681113	0.590726	-1.276104
C	2.114679	1.701565	-2.008258
H	1.386720	2.529176	-1.989298
H	3.081217	2.108495	-1.656393
H	2.256441	1.428094	-3.066111
C	-1.047457	5.076530	0.269398
H	-1.880163	5.535760	0.806725
H	-0.121756	5.450270	0.722111
H	-1.056816	5.399281	-0.773824
C	-1.078102	3.573732	0.397248
O	-0.598960	2.909876	-0.591942
O	-1.518103	3.047215	1.446129

TS4 (Doublet, S**2 after annihilation: 0.7531)
E= -1513.20329387 a.u.
Imaginary Frequency -- -120.1243

C	-2.700113	-0.408690	3.192139
C	-1.338587	-0.344818	2.609686
C	-0.613936	-1.364260	2.103114
C	-1.043623	-2.799470	2.099739
H	-3.174544	-1.387425	3.085355
H	-0.642216	-3.348013	2.960657
C	0.759439	-1.114138	1.601219
N	0.978780	0.140935	1.067850
O	1.615180	-2.013598	1.661842
C	2.246375	0.590398	0.846374
C	3.470048	0.126057	1.366640
C	4.657367	0.763361	1.062891

H	3.451240	-0.744993	2.005495
C	3.461000	2.343263	-0.263993
C	4.659514	1.886009	0.228765
H	5.587628	0.385404	1.474774
H	3.339768	3.203752	-0.909679
H	5.571579	2.406248	-0.038151
O	1.164716	2.208755	-0.454311
N	2.304148	1.711420	0.048285
Co	-0.349245	1.097171	-0.036727
H	-2.684894	-0.157906	4.260313
H	-3.347382	0.338248	2.715775
H	-2.131412	-2.898960	2.122182
H	-0.674837	-3.314213	1.204986
H	-0.882708	0.645378	2.586618
C	-3.186221	3.784312	-0.716213
H	-3.533391	3.582347	-1.731056
H	-4.029291	3.766152	-0.022193
H	-2.767094	4.795511	-0.693789
C	-2.137215	2.807242	-0.289146
O	-1.474940	2.181156	-1.197947
O	-1.881463	2.604939	0.925814
C	3.097124	-1.769582	-1.825167
H	3.654382	-0.833602	-1.906135
H	3.393767	-2.294068	-0.912444
H	3.371191	-2.412380	-2.667935
C	1.606381	-1.525760	-1.832554
O	0.873940	-2.519674	-1.482797
O	1.165435	-0.402726	-2.177348
O	-2.677655	-0.439361	-0.160503
C	-3.704228	-0.033275	-1.004792
H	-3.367334	0.439565	-1.947005
H	-4.370383	-0.866024	-1.306220
H	-4.358351	0.712215	-0.507245
Ag	-1.098686	-1.600153	-1.033741

TS5 (Doublet, S**2 after annihilation: 0.7502)
E= -1137.51799956 a.u.
Imaginary Frequency -- -305.2559

C	2.570232	2.661170	1.233888
C	1.366806	1.926807	0.723144
C	0.324673	2.704046	0.122431
C	0.587445	4.116245	-0.307078
H	3.051732	3.258057	0.452989
H	0.906999	4.771596	0.516456

C	-0.891577	2.097527	-0.243459
N	-0.943536	0.682730	-0.057071
O	-1.877356	2.697046	-0.768085
C	-2.130019	0.066781	0.071841
C	-3.392279	0.594011	0.442806
C	-4.488853	-0.225909	0.596884
H	-3.460286	1.660310	0.608714
C	-3.154583	-2.120516	0.033220
C	-4.379259	-1.609890	0.387718
H	-5.440655	0.205762	0.892792
H	-2.947229	-3.167978	-0.148887
H	-5.221490	-2.281246	0.502979
O	-0.912243	-1.848922	-0.503336
N	-2.087582	-1.302076	-0.125113
Co	0.494227	-0.565250	-0.476965
H	2.253475	3.355635	2.019903
H	3.315737	1.993666	1.671600
H	1.369602	4.202648	-1.078638
H	-0.324735	4.548135	-0.727842
H	1.033645	1.140217	1.407829
C	3.234790	-3.494371	0.241182
H	3.246770	-4.060339	1.174403
H	3.099591	-4.172728	-0.603957
H	4.215539	-3.019138	0.124349
C	2.180363	-2.423321	0.267450
O	1.761772	-2.005731	-0.876854
O	1.781289	-1.944674	1.353548
O	1.910807	0.834419	-0.504851
C	3.264138	0.430939	-0.412429
H	3.481781	-0.270803	-1.223031
H	3.927348	1.297135	-0.528096
H	3.500473	-0.061978	0.542799

TS6 (Doublet, S**2 after annihilation: 0.7613)
E= -1253.18079604 a.u.
Imaginary Frequency -- -74.2149

C	-2.604154	2.492088	-0.372829
C	-1.314690	1.782494	-0.087406
C	-0.124929	2.529889	0.183286
C	-0.197299	4.028096	0.107729
H	-2.999526	3.079357	0.477712
H	-0.919682	4.453542	0.821600
C	1.148235	1.951183	0.445932
N	1.235262	0.544309	0.288492

O	2.166641	2.633559	0.803528
C	2.419976	-0.053067	0.048079
C	3.701940	0.491063	-0.218198
C	4.791114	-0.310662	-0.492321
H	3.793252	1.567205	-0.194232
C	3.413355	-2.243876	-0.281010
C	4.656194	-1.709406	-0.517081
H	5.754883	0.149633	-0.690952
H	3.184005	-3.302775	-0.293747
H	5.489936	-2.370575	-0.721965
O	1.147104	-2.029591	0.167088
N	2.347843	-1.449314	-0.012890
Co	-0.191047	-0.709579	0.484245
H	-2.499159	3.192914	-1.208292
H	-3.384986	1.770266	-0.644754
H	0.778246	4.472789	0.318326
H	-0.513935	4.392400	-0.881015
H	-1.150982	0.651343	-1.533796
O	-1.294216	-0.067754	-2.241849
O	-1.599165	0.659325	0.821188
C	-3.801982	-2.951156	0.873361
H	-4.193400	-3.648122	0.127522
H	-3.472772	-3.498348	1.755499
H	-4.627224	-2.286704	1.147830
C	-2.687203	-2.149943	0.304766
O	-1.571971	-2.087252	0.847058
O	-3.001691	-1.515427	-0.807960
C	-2.186910	0.447805	-3.221011
H	-3.200417	0.599973	-2.823354
H	-1.830399	1.401753	-3.629397
H	-2.248954	-0.272646	-4.041286
H	-2.215011	-1.025063	-1.205474
C	-1.733576	1.037748	2.200252
H	-2.543230	1.769415	2.317389
H	-1.980070	0.134305	2.763990
H	-0.798085	1.466585	2.580859

TS7 (Doublet, S**2 after annihilation: 0.7502)
E= -1513.17054913 a.u.
Imaginary Frequency -- -257.6913

N	-0.217526	-0.179977	-1.114824
C	-1.097347	-1.167234	-1.006009
C	-2.509925	-1.138754	-1.219888
C	-3.288508	-2.255139	-0.943427

H	-2.923333	-0.279986	-1.744375
C	-1.328833	-3.449049	-0.273389
C	-2.696527	-3.423806	-0.451786
H	-4.356676	-2.217807	-1.135518
H	-0.772586	-4.304812	0.090095
H	-3.274543	-4.310775	-0.224815
O	0.760560	-2.420527	-0.352734
N	-0.571469	-2.366297	-0.547019
Co	1.599573	-0.730640	-0.729819
C	4.836295	-2.251209	1.322779
H	4.761367	-2.709260	2.310711
H	5.195861	-2.985935	0.599089
H	5.586366	-1.454875	1.378040
C	3.517976	-1.654760	0.903514
O	3.351716	-1.510710	-0.368767
O	2.678372	-1.301328	1.756316
C	3.402676	3.017286	-1.332915
H	3.982969	2.796962	-2.233217
H	2.518786	3.576126	-1.659147
H	3.993084	3.650206	-0.668111
C	2.974391	1.743001	-0.644103
O	3.199404	1.568427	0.567421
O	2.361115	0.909513	-1.417058
C	0.438700	3.048403	1.620804
C	0.016350	1.904050	0.764150
C	-0.653422	2.125565	-0.452007
C	-1.263446	3.467177	-0.748448
H	1.230530	3.621548	1.119934
H	-1.850138	3.424194	-1.669284
C	-0.738806	1.114640	-1.454096
O	-1.260817	1.270528	-2.582818
H	-0.385368	3.741667	1.815805
H	0.840842	2.706909	2.578187
H	-1.929764	3.806021	0.055310
H	-0.512021	4.259081	-0.880306
H	0.664139	1.030769	0.815328
Ag	-2.869431	0.440195	0.694938
O	-1.212679	1.091869	2.061366
C	-0.538926	0.012706	2.641349
H	0.363141	0.343483	3.189107
H	-1.178505	-0.511087	3.368793
H	-0.201676	-0.739885	1.901337

TS8 (Doublet, S**2 after annihilation: 0.7505)

E= -1513.10864922 a.u.

Imaginary Frequency -- -1253.9268

N	-0.032659	1.273399	-0.047165
C	0.486605	2.517475	-0.128422
C	-0.115499	3.764386	0.126817
C	0.574724	4.939708	-0.097547
H	-1.132967	3.765068	0.493590
C	2.467111	3.680552	-0.835274
C	1.885471	4.902251	-0.588967
H	0.092341	5.892016	0.100431
H	3.471633	3.536144	-1.213132
H	2.454183	5.803996	-0.781969
O	2.389917	1.361524	-0.839682
N	1.785248	2.534813	-0.598437
Co	1.194575	-0.132648	-0.419066
C	3.515671	-3.493206	-1.414598
H	3.183710	-4.224683	-2.153466
H	4.418946	-2.989791	-1.759118
H	3.753189	-4.040665	-0.496143
C	2.434190	-2.516145	-1.112117
O	2.703213	-1.302469	-0.918010
O	1.233667	-2.961074	-1.032358
C	2.354591	-1.050352	3.799486
H	3.109411	-0.269445	3.927785
H	1.471480	-0.730435	4.364048
H	2.719099	-1.982867	4.236675
C	1.979201	-1.219664	2.338140
O	1.863101	-2.378962	1.872238
O	1.775607	-0.135767	1.695064
C	-1.378778	-2.726522	-0.356610
C	-0.824777	-1.299004	-0.431875
C	-1.477228	-0.477557	0.672181
C	-1.610056	-1.177965	2.016340
H	-0.953240	-3.293444	0.477762
H	-1.974228	-0.471072	2.766738
C	-1.045780	0.921451	0.826669
O	-1.496012	1.704379	1.693365
H	-2.472751	-2.678476	-0.226233
H	-1.173000	-3.292738	-1.268911
H	-2.296561	-2.028014	2.007603
H	-0.637267	-1.560337	2.367866
H	0.538270	-2.023688	-0.684986
Ag	-3.561829	-0.152252	-0.103134
O	-1.305512	-0.739736	-1.700560

C	-0.562009	-1.115973	-2.839021
H	-0.515033	-2.205156	-2.986562
H	-1.058889	-0.673639	-3.707108
H	0.473529	-0.732690	-2.795250

TS9 (Doublet, S**2 after annihilation: 0.7549)
E= -865.280755281 a.u.
Imaginary Frequency -- -166.9957

Co	0.532722	-0.819349	0.754748
C	3.821480	-1.188044	-1.173760
H	3.777715	-1.957200	-1.946611
H	4.003310	-0.228717	-1.669494
H	4.649590	-1.380903	-0.490712
C	2.535010	-1.105166	-0.441725
O	1.415830	-1.287307	-1.023892
O	2.493521	-0.809098	0.805863
C	-3.464520	-2.191573	0.109376
H	-3.569739	-2.601197	1.116350
H	-4.420390	-1.793836	-0.231956
H	-3.177050	-3.021213	-0.543894
C	-2.390205	-1.146081	0.072971
O	-2.606570	-0.074279	-0.555382
O	-1.309878	-1.439745	0.693860
O	1.152679	1.779178	0.048297
C	1.331857	2.400519	1.281741
H	0.835463	3.387140	1.364812
H	0.968602	1.808699	2.145856
H	2.406886	2.584349	1.473675
Ag	-0.851580	1.262923	-0.434847

TS10 (Singlet)
E= -1250.75312161 a.u.
Imaginary Frequency -- -1539.3560

C	-2.990884	1.505603	-0.058354
C	-1.564184	1.269048	-0.459363
C	-0.694010	2.309648	-0.596343
C	-1.069485	3.741585	-0.461554
H	-3.055423	2.049350	0.890345
H	-0.952893	4.075292	0.578251
C	0.758395	2.026994	-0.763220
N	1.034349	0.694480	-0.566409
O	1.600386	2.894628	-1.009345
C	2.259713	0.129419	-0.408970
C	3.534676	0.707659	-0.466470

C	4.656431	-0.075303	-0.261920
H	3.603850	1.766405	-0.674567
C	3.265969	-1.994309	0.058553
C	4.524486	-1.441121	0.002131
H	5.640345	0.380158	-0.304244
H	3.049890	-3.034713	0.264436
H	5.384247	-2.076866	0.174560
O	0.960723	-1.796966	-0.108903
N	2.177341	-1.219565	-0.148479
Co	-0.394829	-0.432974	-0.144117
H	-3.510999	2.110496	-0.813318
H	-3.550503	0.573640	0.051928
H	-0.415487	4.372394	-1.069209
H	-2.109522	3.920666	-0.743404
H	-1.696924	0.305106	-1.367099
C	-3.438613	-2.646686	-1.753951
H	-3.314874	-2.877264	-2.813751
H	-3.367170	-3.554977	-1.156608
H	-4.437562	-2.217030	-1.629596
C	-2.433636	-1.638485	-1.326138
O	-1.849883	-1.754376	-0.208358
O	-2.197367	-0.649429	-2.115472
C	-0.626885	0.090873	3.957560
H	-1.449705	0.391064	4.608087
H	-0.212681	-0.848720	4.335852
H	0.167826	0.838177	3.988048
C	-1.138499	-0.132704	2.556100
O	-2.321556	-0.379240	2.325278
O	-0.174142	-0.054695	1.675212

TS11 (Singlet)

E= -1283.84437822 a.u.

Imaginary Frequency -- -360.4812

N	-0.573472	-0.737191	0.497163
C	-1.647450	-1.512423	0.252919
C	-1.973616	-2.782640	0.769371
C	-3.144503	-3.416223	0.401321
H	-1.279101	-3.228359	1.467368
C	-3.695631	-1.562489	-0.997017
C	-4.025860	-2.799233	-0.492404
H	-3.377015	-4.393009	0.814467
H	-4.299866	-1.001623	-1.699086
H	-4.955922	-3.262516	-0.799351
O	-2.247387	0.234556	-1.199245

N	-2.540594	-0.947076	-0.646475
Co	-0.604647	1.017063	-0.208583
C	1.954655	2.390287	0.489704
C	1.174997	1.131318	0.396678
C	1.567668	-0.036410	1.153309
C	2.592059	0.080416	2.257261
H	1.830681	2.791912	1.503112
H	2.844318	-0.909796	2.647799
C	0.519807	-1.043599	1.305091
O	0.579573	-2.049721	2.043391
H	3.031103	2.235228	0.335215
H	1.608316	3.159016	-0.203643
H	3.523254	0.549739	1.926731
H	2.220753	0.670951	3.109392
Ag	2.634686	-1.200755	-0.583377
O	0.993437	0.659305	-1.281792
C	1.286211	1.613918	-2.293525
H	0.787457	2.574704	-2.125752
H	2.370572	1.778044	-2.347168
H	0.941994	1.210741	-3.251868
C	-2.334960	4.216996	0.967334
H	-3.026767	4.009369	1.783809
H	-1.575590	4.922401	1.318835
H	-2.863786	4.697062	0.141046
C	-1.668303	2.976225	0.500201
O	-0.969163	2.951466	-0.568025
O	-1.746104	1.882385	1.157333

TS12 (Doublet, S**2 after annihilation: 0.7833)
E= -1137.18958954 a.u.
Imaginary Frequency -- -166.4295

C	3.381879	1.714053	1.262961
C	1.981288	1.485356	0.874511
C	1.236426	2.405917	0.135827
C	1.838109	3.647964	-0.397822
H	3.958727	2.242345	0.500325
H	2.660455	3.405555	-1.083789
C	-0.200484	2.175026	-0.101030
N	-0.636501	0.857727	-0.083125
O	-0.955460	3.131684	-0.307584
C	-1.972177	0.585295	0.054075
C	-2.999615	1.395208	0.566974
C	-4.281362	0.895663	0.685493
H	-2.762918	2.405093	0.867355

C	-3.557770	-1.207454	-0.181891
C	-4.571748	-0.417409	0.299595
H	-5.063180	1.530485	1.088915
H	-3.654437	-2.239083	-0.494600
H	-5.568670	-0.832488	0.380569
O	-1.347868	-1.491854	-0.786026
N	-2.309452	-0.695698	-0.293872
Co	0.357169	-0.737488	-0.586212
H	3.385030	2.342204	2.165066
H	3.884537	0.784573	1.539056
H	1.102374	4.251676	-0.928280
H	2.283091	4.252976	0.401136
H	1.443145	0.710720	1.415999
C	1.615092	-3.853096	1.152003
H	0.816233	-4.601084	1.134744
H	2.480035	-4.276643	0.637599
H	1.858216	-3.623411	2.189439
C	1.152892	-2.648153	0.444164
O	1.131692	-2.534674	-0.823938
O	0.724450	-1.607985	1.075256
O	2.006519	0.138022	-0.807111
C	3.216728	-0.573129	-0.766097
H	3.223034	-1.314247	-1.575302
H	4.046109	0.124027	-0.935533
H	3.376135	-1.097698	0.186643

TS13 (Doublet, S**2 after annihilation: 0.7561)
E= -1365.91203501 a.u.
Imaginary Frequency -- -171.5320

C	2.611304	2.974549	-0.251461
C	1.368864	2.122797	-0.220196
C	0.085150	2.757365	-0.268280
C	-0.028348	4.166374	0.148049
H	2.750488	3.448390	-1.227587
H	0.385296	4.808571	-0.643769
C	-1.185933	2.078368	-0.588108
N	-1.235969	0.742619	-0.290113
O	-2.129325	2.767006	-1.016215
C	-2.433005	0.109235	-0.080146
C	-3.742377	0.618512	-0.127715
C	-4.819103	-0.182181	0.202314
H	-3.878425	1.645300	-0.433411
C	-3.334001	-2.009095	0.604807
C	-4.615973	-1.514731	0.570877

H	-5.822407	0.228684	0.161561
H	-3.061950	-3.021793	0.872936
H	-5.438564	-2.172751	0.823876
O	-1.059367	-1.738785	0.351595
N	-2.287216	-1.210167	0.289406
Co	0.242882	-0.493133	-0.222767
H	2.538681	3.758012	0.503120
H	3.494897	2.382808	-0.011345
H	-1.062581	4.456615	0.324151
H	0.575948	4.374947	1.036229
H	1.265148	1.648627	0.860775
C	1.778155	-1.413154	3.063804
H	0.954954	-2.014602	2.670348
H	2.682931	-2.018984	3.139905
H	1.496222	-1.091515	4.072009
C	2.015601	-0.194391	2.210366
O	3.156830	0.255762	2.025427
O	0.933935	0.347144	1.727866
O	1.420390	0.917532	-1.023673
C	2.708453	0.514033	-1.532915
H	2.495476	-0.323337	-2.195920
H	3.146202	1.326958	-2.113486
H	3.378377	0.201559	-0.728127
C	2.505072	-3.657967	-1.439684
H	2.655080	-4.180142	-0.492511
H	2.178555	-4.361068	-2.207380
H	3.474569	-3.248116	-1.743944
C	1.530009	-2.527646	-1.295128
O	0.751230	-2.207810	-2.217557
O	1.580353	-1.879992	-0.175852

TS14 (Triplet, S**2 after annihilation: 2.0006)
E= -1137.37675006 a.u.
Imaginary Frequency -- -485.5002

C	2.887688	2.374567	1.115073
C	1.575920	1.796455	0.720377
C	0.568851	2.639075	0.178225
C	0.879010	4.047192	-0.182788
H	3.328737	2.996230	0.331084
H	1.295041	4.608270	0.663176
C	-0.751293	2.126999	-0.117271
N	-0.891920	0.738177	-0.013632
O	-1.684975	2.877058	-0.472580
C	-2.137999	0.190671	0.051429

C	-3.382135	0.795171	0.330509
C	-4.528115	0.034231	0.434802
H	-3.408241	1.865456	0.467014
C	-3.265204	-1.939410	-0.005003
C	-4.477532	-1.353831	0.257436
H	-5.471816	0.522229	0.656933
H	-3.099175	-2.998699	-0.153615
H	-5.361367	-1.976381	0.322963
O	-0.991086	-1.802218	-0.351903
N	-2.148657	-1.177098	-0.100264
Co	0.466490	-0.619043	-0.442140
H	2.728044	3.018297	1.988506
H	3.602092	1.604917	1.412812
H	1.634862	4.098984	-0.979146
H	-0.016410	4.562082	-0.532157
H	1.239171	0.976648	1.353828
C	2.917186	-3.595725	0.515427
H	2.937438	-3.933740	1.551891
H	2.647545	-4.423535	-0.143698
H	3.927152	-3.275943	0.236721
C	1.981439	-2.442588	0.340916
O	1.595512	-2.160616	-0.855577
O	1.608676	-1.734773	1.310533
O	1.866257	0.648010	-0.722458
C	3.207659	0.204357	-0.822735
H	3.279907	-0.495413	-1.660751
H	3.862735	1.057644	-1.030464
H	3.555169	-0.303101	0.087433

TS15 (Triplet, S**2 after annihilation: 2.0423)
E= -1399.61188668 a.u.
Imaginary Frequency -- -196.7130

N	-0.229794	0.895565	-0.165196
C	-0.385908	2.245751	-0.045102
C	0.598473	3.232068	0.118300
C	0.255753	4.559655	0.298846
H	1.632778	2.916965	0.105401
C	-2.051162	3.954521	0.166299
C	-1.090422	4.927419	0.316574
H	1.033096	5.305462	0.428186
H	-3.118091	4.136179	0.170718
H	-1.404039	5.956319	0.448341
O	-2.688102	1.769748	-0.151085
N	-1.709191	2.653192	-0.009521

Co	-2.023751	-0.145390	-0.378960
C	-4.692626	-2.515678	1.744383
H	-4.509800	-2.268276	2.793769
H	-5.767602	-2.540124	1.559036
H	-4.282265	-3.517530	1.582444
C	-3.993384	-1.533539	0.841161
O	-4.615095	-0.936927	-0.063265
O	-2.732211	-1.371153	1.059592
O	-2.101572	-0.958458	-2.132242
C	0.535109	-3.242466	0.823404
C	0.560165	-1.778181	0.616225
C	0.942612	-1.148036	-0.557984
C	1.607568	-1.880801	-1.673085
H	-0.471715	-3.625210	0.608714
H	1.917846	-1.202262	-2.470071
C	0.919242	0.337281	-0.602516
O	1.932748	0.944530	-1.035864
H	1.239436	-3.772478	0.177401
H	0.750081	-3.490374	1.865971
H	2.490260	-2.438584	-1.329033
H	0.929549	-2.629752	-2.100196
H	0.026571	-1.173958	1.347893
Ag	3.820625	-0.327787	-0.083943
O	2.437135	-1.335565	1.548756
C	2.145397	-0.384638	2.527603
H	1.433870	-0.762557	3.278856
H	3.077445	-0.117413	3.051361
H	1.744083	0.557276	2.112556
C	-2.014374	-2.342012	-2.158736
H	-1.327581	-2.767679	-1.396899
H	-2.988696	-2.850432	-1.999319
H	-1.642313	-2.710799	-3.134260

TS16 (Triplet, S**2 after annihilation: 2.0016)
E= -1399.55779060 a.u.
Imaginary Frequency -- -1651.8668

N	-0.413013	0.960566	0.599445
C	-0.935803	2.159244	0.307954
C	-2.252550	2.640451	0.457730
C	-2.592743	3.896481	-0.000612
H	-2.970493	1.995383	0.947625
C	-0.352452	4.229704	-0.762323
C	-1.631524	4.712381	-0.615909
H	-3.612710	4.255825	0.120718

H	0.463933	4.768433	-1.231831
H	-1.866672	5.705823	-0.973753
O	1.228147	2.537907	-0.521373
N	-0.027374	2.995995	-0.320240
Co	1.474798	0.826194	0.359356
C	5.052578	-0.904167	-0.969713
H	5.098116	-1.649144	-1.765462
H	5.578172	0.003419	-1.267658
H	5.560633	-1.324531	-0.094500
C	3.640211	-0.605620	-0.591045
O	3.325435	0.564339	-0.256218
O	2.833575	-1.613321	-0.592470
O	1.700474	-0.040503	1.991610
C	0.394439	-3.077870	-0.887505
C	0.178162	-1.630391	-0.477969
C	-0.647936	-1.437277	0.704626
C	-0.591331	-2.480935	1.792253
H	0.940198	-3.636120	-0.121022
H	-1.273093	-2.211445	2.603109
C	-1.107657	-0.105791	1.146183
O	-2.027279	0.047769	1.983929
H	-0.572138	-3.581160	-1.054949
H	0.976302	-3.147209	-1.810748
H	-0.848624	-3.487785	1.453973
H	0.423476	-2.532164	2.220957
H	1.678237	-1.383014	-0.370813
Ag	-2.748078	-1.784257	-0.377137
O	-0.317640	-0.867020	-1.579841
C	0.645078	-0.499813	-2.549558
H	1.194875	-1.363627	-2.952418
H	0.100873	-0.026149	-3.370217
H	1.375074	0.220014	-2.145424
C	2.929420	-0.523289	2.432780
H	3.767232	0.178519	2.279518
H	2.880672	-0.721184	3.517511
H	3.219725	-1.479010	1.957359

R' (Singlet)

E= -648.344592926 a.u.

C	4.137570	1.579271	-0.431947
C	2.805722	0.933399	-0.350650
C	2.523047	-0.315448	0.071400
C	3.535396	-1.326383	0.505923
H	4.958020	0.940048	-0.100826

H	3.564274	-2.171993	-0.189035
C	1.134029	-0.838880	0.041212
N	0.129226	0.104151	0.088105
O	0.900292	-2.048377	-0.010029
C	-1.225372	-0.099251	0.001963
C	-1.907817	-1.286344	-0.246330
C	-3.294230	-1.289336	-0.310076
H	-1.328143	-2.188315	-0.381374
C	-3.294443	1.066424	0.117139
C	-3.991469	-0.098005	-0.124470
H	-3.822249	-2.217003	-0.502970
H	-3.746459	2.037532	0.272327
H	-5.073908	-0.056672	-0.164263
O	-1.286859	2.179833	0.407705
N	-1.935002	1.077597	0.182153
H	0.340960	1.080823	0.286369
H	4.347810	1.899077	-1.459498
H	4.153398	2.494757	0.171771
H	4.539761	-0.906241	0.568685
H	3.280800	-1.744889	1.485459
H	1.984098	1.556311	-0.704844

OAc'$^{-}$ (Singlet)
E= -228.681385608 a.u.

O	-0.745985	1.133303	0.002424
C	-0.189754	-0.000002	-0.012141
C	1.342311	-0.011649	-0.004455
H	1.701719	0.080699	1.027693
H	1.750406	-0.943906	-0.407018
H	1.758736	0.834950	-0.559987
O	-0.769790	-1.121032	0.002436

HOAc' (Singlet)
E= -229.152951055 a.u.

C	1.383495	-0.107697	-0.000326
H	1.676279	-0.662180	0.895313
H	1.671496	-0.718307	-0.859855
H	1.913587	0.843092	-0.030488
C	-0.089310	0.119518	-0.000794
O	-0.642787	1.204167	0.000151
O	-0.770789	-1.042671	-0.000161
H	-1.717865	-0.825503	0.001835

INT1' (Singlet)

E= -647.851992665 a.u.

C	4.167088	1.616878	0.149046
C	2.821468	0.992597	0.064961
C	2.515285	-0.317866	0.000108
C	3.531513	-1.416558	-0.008415
H	4.347607	2.279394	-0.707453
H	3.344354	-2.132483	0.799756
C	1.085914	-0.783826	-0.087698
N	0.166755	0.197471	0.000219
O	0.896554	-2.019873	-0.245534
C	-1.152556	-0.053281	0.008665
C	-1.848867	-1.277175	0.122719
C	-3.231768	-1.353190	0.163745
H	-1.240016	-2.169186	0.175870
C	-3.315470	1.021096	-0.028849
C	-3.982379	-0.181549	0.083214
H	-3.719391	-2.319772	0.257214
H	-3.809192	1.983379	-0.098056
H	-5.066943	-0.182315	0.103886
O	-1.410444	2.283948	-0.169158
N	-1.956773	1.110415	-0.065140
H	4.246615	2.257977	1.036299
H	4.987076	0.895339	0.186806
H	3.477614	-1.997302	-0.936144
H	4.554356	-1.048184	0.097053
H	1.977794	1.682586	0.051765

$Cp^{*}Co^{III}I$ (Doublet, S**2 after annihilation: 0.7641)

E= -547.557699836 a.u.

Co	0.224791	-0.001218	-0.560119
C	1.027582	-0.731445	1.218684
C	1.856495	-1.173306	0.162014
C	2.312227	-0.001166	-0.544933
C	1.856348	1.173494	0.157674
C	0.312121	-1.574227	2.211351
H	0.126737	-2.583124	1.830844
H	-0.653843	-1.139904	2.488371
H	0.892569	-1.675618	3.137813
C	2.163339	-2.579508	-0.212345
H	2.189766	-2.716398	-1.298403
H	1.425607	-3.278637	0.191933
H	3.145128	-2.890169	0.168110
C	3.205310	-0.003825	-1.731660
H	3.057956	0.883152	-2.354889

H	3.050848	-0.888631	-2.356284
H	4.260203	-0.008362	-1.426074
C	2.167508	2.578233	-0.218655
H	3.150865	2.885525	0.160443
H	1.432803	3.280398	0.185778
H	2.193049	2.714139	-1.304850
C	1.027774	0.735219	1.216208
C	0.312642	1.581735	2.205961
H	-0.652482	1.147611	2.486183
H	0.125723	2.588751	1.821327
H	0.894091	1.687581	3.131309
I	-2.418046	-0.000087	-0.349513

$Cp^*Co^{III}I_2$ (Singlet)

E= -559.027218804 a.u.

Co	-0.009109	0.319797	-0.001305
C	-0.379460	1.932827	-1.152206
C	-1.244803	1.969524	-0.011143
C	-0.410748	1.921466	1.152752
C	0.972291	1.955198	0.731513
C	-0.792758	1.842622	-2.568211
H	-1.843867	1.566173	-2.669750
H	-0.186309	1.112962	-3.113656
H	-0.648028	2.810452	-3.064943
C	-2.721848	2.063252	-0.030388
H	-3.173649	1.644896	0.871252
H	-3.159070	1.560095	-0.895706
H	-3.012635	3.119724	-0.082724
C	-0.864661	1.804165	2.554232
H	-0.227051	1.117917	3.119591
H	-1.896033	1.452225	2.619905
H	-0.807615	2.779258	3.054693
C	2.139189	1.975237	1.640773
H	2.275227	2.989650	2.036304
H	3.061777	1.688013	1.131840
H	1.996419	1.313738	2.499308
C	0.991205	1.961294	-0.693255
C	2.178801	1.980241	-1.574940
H	2.048767	1.321964	-2.437943
H	3.086783	1.681566	-1.047313
H	2.331794	2.994802	-1.963438
I	-1.941638	-1.471428	-0.003567
I	1.964207	-1.438438	-0.002413

I⁻ (Singlet)

E= -11.5903739874 a.u.

I	0.000000	0.000000	0.000000

INT11' (Doublet, S**2 after annihilation: 0.7504)

E= -647.687030035 a.u.

C	-3.735062	1.624097	1.129641
C	-2.551548	1.032691	0.466880
C	-2.441613	-0.197955	-0.076986
C	-3.521986	-1.227495	-0.130212
H	-4.594491	0.952362	1.176050
H	-3.198075	-2.160026	0.344256
C	-1.156904	-0.626342	-0.660493
N	-0.115865	0.296520	-0.649620
O	-1.025030	-1.728976	-1.204631
C	1.053639	-0.025103	-0.174903
C	1.400522	-1.179118	0.593503
C	2.663566	-1.361697	1.079796
H	0.614417	-1.902736	0.777993
C	3.360506	0.718623	0.077326
C	3.659854	-0.394397	0.816132
H	2.903348	-2.241357	1.667330
H	4.062212	1.505046	-0.168920
H	4.670207	-0.511448	1.191358
O	1.857447	1.962149	-1.115930
N	2.102206	0.923919	-0.424748
H	-3.485705	1.932620	2.152057
H	-4.043840	2.541246	0.613600
H	-4.437452	-0.896300	0.362308
H	-3.766864	-1.483344	-1.166631
H	-1.673320	1.675649	0.411564

[Cp*Co^{III}OEt]⁺ (Singlet)

E= -690.432702611 a.u.

Co	-0.291340	-0.054833	-0.397671
C	1.134887	-1.128316	0.453989
C	1.734935	-0.258287	-0.540444
C	1.432470	1.086973	-0.196742
C	0.620436	1.070502	0.986741
C	1.192445	-2.605253	0.451976
H	1.205726	-3.006922	-0.564245
H	0.348562	-3.045361	0.988088
H	2.111011	-2.944065	0.946890
C	2.468456	-0.727050	-1.736312

H	2.488960	0.024771	-2.527831
H	2.034799	-1.646060	-2.140036
H	3.508199	-0.954948	-1.471085
C	1.774540	2.298205	-0.979226
H	0.958900	3.026341	-0.975490
H	2.017615	2.061294	-2.017262
H	2.648607	2.794940	-0.541868
C	0.004616	2.246654	1.636999
H	0.684754	2.652466	2.396438
H	-0.928856	1.987459	2.142992
H	-0.197364	3.048804	0.923065
C	0.460990	-0.301018	1.413806
C	-0.315131	-0.759074	2.586308
H	-0.669055	-1.785170	2.462741
H	-1.179902	-0.116293	2.770762
H	0.311401	-0.730100	3.485559
O	-1.827581	-0.944483	-0.343395
C	-2.668495	-0.326952	-1.295277
H	-3.078631	-1.099624	-1.962337
H	-2.068734	0.342821	-1.950249
C	-3.767085	0.470373	-0.631796
H	-4.425088	0.923404	-1.379777
H	-4.376526	-0.170031	0.013233
H	-3.345934	1.272073	-0.015435

INT19' (Doublet, S**2 after annihilation: 0.7558)
E= -1338.20036259 a.u.

C	3.101789	2.457804	1.564935
C	1.764437	2.172073	0.997343
C	1.267547	2.563934	-0.193620
C	1.964439	3.479274	-1.149794
H	3.021209	3.112675	2.441873
H	1.575565	4.500621	-1.065293
C	-0.132107	2.234573	-0.540294
N	-0.667448	1.079830	0.034770
O	-0.770139	2.966589	-1.306577
C	-2.023341	0.939020	0.132240
C	-3.041868	1.897223	-0.024224
C	-4.365730	1.560143	0.186908
H	-2.762004	2.898794	-0.314171
C	-3.713220	-0.668939	0.716159
C	-4.714737	0.258811	0.554279
H	-5.132899	2.316573	0.057890
H	-3.858314	-1.702461	1.004364

H	-5.743204	-0.041703	0.712548
O	-1.485924	-1.253034	0.738801
N	-2.422206	-0.315954	0.519246
Co	0.261760	-0.688427	0.098822
H	3.790920	2.927077	0.858742
H	3.558899	1.529365	1.931624
H	1.803766	3.180636	-2.191216
H	3.040446	3.520641	-0.968868
O	0.968005	-0.735488	1.779625
C	1.854174	-1.941937	-0.498640
C	0.570952	-2.546612	-0.700022
C	-0.141077	-1.754960	-1.685977
C	0.691227	-0.667682	-2.053386
C	2.916235	-2.403180	0.414610
H	2.544241	-3.119000	1.149317
H	3.379690	-1.564929	0.943266
H	3.705980	-2.894925	-0.165925
C	0.070623	-3.768497	-0.038479
H	-1.020183	-3.802373	-0.023351
H	0.436896	-3.845453	0.988944
H	0.422550	-4.655454	-0.579161
C	-1.494699	-2.036320	-2.208439
H	-2.007223	-1.122800	-2.521363
H	-2.113454	-2.549655	-1.469221
H	-1.423324	-2.687656	-3.087725
C	0.355397	0.376659	-3.040834
H	0.592481	0.005981	-4.045701
H	0.931655	1.291342	-2.890012
H	-0.708856	0.623960	-3.031520
C	1.917413	-0.762771	-1.302248
C	3.102959	0.110350	-1.428313
H	3.578681	0.291113	-0.460726
H	2.870977	1.069870	-1.892738
H	3.847919	-0.391455	-2.059366
C	0.149993	-0.418004	2.876880
H	-0.517483	-1.265855	3.096311
H	-0.505250	0.436652	2.636465
C	1.020960	-0.106555	4.074617
H	1.670164	-0.952767	4.318329
H	0.397661	0.108271	4.947897
H	1.653295	0.766591	3.884225
H	1.105530	1.585746	1.631241

TS12' (Doublet, S**2 after annihilation: 0.7504)

E= -1338.17111839 a.u.

Imaginary Frequency -- -346.4685

C	-3.401323	-2.145625	1.147075
C	-1.945874	-2.005283	0.890687
C	-1.415881	-2.289152	-0.376453
C	-2.239639	-2.864405	-1.469537
H	-3.711955	-3.179231	0.945469
H	-2.334432	-3.950646	-1.329023
C	0.059598	-2.265000	-0.550491
N	0.648062	-1.166551	-0.001293
O	0.628027	-3.217393	-1.105500
C	1.978364	-1.087609	0.283952
C	3.036224	-1.951448	-0.029096
C	4.319852	-1.663832	0.405640
H	2.814661	-2.830613	-0.618148
C	3.520092	0.336485	1.442618
C	4.567307	-0.510523	1.151470
H	5.134014	-2.334991	0.151770
H	3.602382	1.261193	2.000028
H	5.561433	-0.251518	1.495393
O	1.267607	0.869598	1.327908
N	2.271768	0.039019	1.019652
Co	-0.178466	0.618910	0.007859
H	-3.991445	-1.507591	0.480937
H	-3.671429	-1.914974	2.175998
H	-1.783604	-2.715623	-2.451895
H	-3.256257	-2.464516	-1.475312
O	-1.600693	0.037963	1.222665
C	-1.407627	2.173030	-0.643775
C	-0.066095	2.615094	-0.540372
C	0.749920	1.775223	-1.396864
C	-0.121904	0.861908	-2.068450
C	-2.586273	2.682309	0.093875
H	-2.305641	3.258028	0.978903
H	-3.237414	1.860860	0.408048
H	-3.180339	3.338715	-0.553157
C	0.458479	3.680433	0.343420
H	1.362035	3.351625	0.865601
H	-0.276496	3.993033	1.088653
H	0.729922	4.562789	-0.247991
C	2.213816	1.911226	-1.595022
H	2.694038	0.947163	-1.788888
H	2.696278	2.366085	-0.725674
H	2.426070	2.555238	-2.456890

C	0.299696	-0.186038	-3.025910
H	0.289662	0.217101	-4.045542
H	-0.373626	-1.047934	-3.011061
H	1.315976	-0.538687	-2.827490
C	-1.446214	1.061415	-1.568044
C	-2.684172	0.359050	-1.981331
H	-3.209353	-0.070504	-1.120581
H	-2.480587	-0.442096	-2.694243
H	-3.377678	1.061850	-2.457792
C	-1.261342	0.278276	2.577817
H	-0.908331	1.322220	2.612706
H	-0.390854	-0.328519	2.870951
C	-2.415845	0.086046	3.530905
H	-3.317315	0.588779	3.165781
H	-2.160135	0.517268	4.503712
H	-2.646442	-0.969464	3.696571
H	-1.259842	-2.103709	1.729224

INT20' (Doublet, S**2 after annihilation: 0.7502)
E= -1338.19455454 a.u.

C	-3.465191	-1.792458	0.906557
C	-1.953182	-1.712411	0.823973
C	-1.403186	-2.156313	-0.512106
C	-2.234473	-3.027338	-1.379452
H	-3.795892	-2.830474	0.814271
H	-2.400097	-4.014741	-0.923490
C	0.042867	-2.152473	-0.765676
N	0.694225	-1.101856	-0.172909
O	0.590139	-3.053270	-1.426329
C	2.012880	-1.074953	0.134576
C	3.060711	-1.940526	-0.211400
C	4.338672	-1.704357	0.265872
H	2.834027	-2.777549	-0.857940
C	3.557299	0.244650	1.412221
C	4.594380	-0.601048	1.084356
H	5.145794	-2.374431	-0.012578
H	3.645002	1.128688	2.031360
H	5.587277	-0.382761	1.458455
O	1.307289	0.808677	1.307498
N	2.312105	-0.006976	0.953542
Co	-0.154763	0.621434	0.008752
H	-3.944353	-1.212984	0.112524
H	-3.827480	-1.415414	1.862672
H	-1.741440	-3.199138	-2.338694

H	-3.226478	-2.601418	-1.557141
O	-1.533997	-0.350721	1.204758
C	-1.496959	2.146964	-0.429004
C	-0.189452	2.662902	-0.276209
C	0.672355	1.978680	-1.226643
C	-0.149116	1.103853	-2.011158
C	-2.693468	2.468125	0.383044
H	-2.436319	2.978469	1.314409
H	-3.258559	1.563436	0.627961
H	-3.365869	3.125592	-0.180732
C	0.279622	3.634108	0.738221
H	1.219342	3.304466	1.191072
H	-0.453179	3.779314	1.535388
H	0.466177	4.609395	0.273902
C	2.127238	2.207032	-1.391960
H	2.644440	1.306568	-1.737577
H	2.590670	2.533125	-0.456858
H	2.308790	2.990625	-2.137416
C	0.325251	0.208005	-3.089912
H	0.274299	0.730036	-4.052770
H	-0.290642	-0.691482	-3.175588
H	1.364595	-0.096764	-2.939825
C	-1.476996	1.163714	-1.494407
C	-2.670125	0.472034	-2.034918
H	-3.337691	0.135050	-1.237765
H	-2.405189	-0.387423	-2.654667
H	-3.247580	1.163677	-2.661303
C	-1.112039	-0.228407	2.591741
H	-0.755394	0.803434	2.660238
H	-0.251256	-0.891736	2.747821
C	-2.200697	-0.486572	3.597476
H	-3.073424	0.149316	3.420359
H	-1.808202	-0.248959	4.590994
H	-2.520192	-1.531966	3.617060
H	-1.512688	-2.377626	1.586586

TS13' (Doublet, S**2 after annihilation: 0.7533)
E= -1566.86003985 a.u.
Imaginary Frequency -- -1876.5116

C	-1.664952	-3.262513	0.001195
C	-1.440742	-1.763983	0.098358
C	-1.337075	-1.188869	1.405553
C	-1.994227	-1.924253	2.522179
H	-2.603043	-3.546702	0.487407

H	-3.081797	-2.000791	2.376485
C	-0.981847	0.203243	1.676591
N	-0.192246	0.781220	0.706924
O	-1.379238	0.795658	2.704851
C	-0.200561	2.092095	0.402556
C	-0.837257	3.181174	1.027361
C	-0.734785	4.451363	0.492606
H	-1.394442	2.982271	1.932996
C	0.616023	3.598594	-1.286335
C	0.009378	4.671305	-0.672764
H	-1.225301	5.281793	0.990737
H	1.202512	3.650999	-2.195292
H	0.124795	5.658555	-1.103200
O	1.023901	1.317469	-1.427523
N	0.487912	2.356603	-0.767948
Co	1.206999	-0.199886	-0.206095
H	-0.861559	-3.843600	0.471245
H	-1.729260	-3.573525	-1.043193
H	-1.831624	-1.405990	3.468877
H	-1.621208	-2.951289	2.605522
O	-0.506148	-1.313217	-0.897180
C	2.426991	-1.890126	-0.382566
C	3.058125	-0.726817	-0.897048
C	3.175523	0.238858	0.184690
C	2.598178	-0.339366	1.346615
C	2.078917	-3.112242	-1.149997
H	1.838750	-2.881776	-2.192401
H	1.221074	-3.630563	-0.713104
H	2.919329	-3.817497	-1.158289
C	3.499595	-0.491510	-2.294373
H	3.217870	0.510175	-2.636133
H	3.061996	-1.218616	-2.984031
H	4.590099	-0.566119	-2.383000
C	3.781337	1.587875	0.064789
H	3.351449	2.295028	0.780471
H	3.645538	1.998421	-0.939741
H	4.861167	1.548545	0.255490
C	2.443235	0.298136	2.677381
H	3.175223	-0.105927	3.387168
H	1.451448	0.106680	3.100592
H	2.589370	1.380716	2.633677
C	2.088922	-1.639429	0.995212
C	1.516255	-2.619014	1.954722
H	0.785670	-3.279477	1.481416

H	1.030304	-2.125152	2.801054
H	2.309889	-3.257885	2.364755
C	-1.058468	-0.763959	-2.114531
H	-0.179718	-0.393845	-2.650661
H	-1.683593	0.102622	-1.859734
C	-1.807361	-1.764932	-2.956682
H	-1.183228	-2.631770	-3.197174
H	-2.091230	-1.286078	-3.899547
H	-2.725948	-2.110315	-2.476125
H	-2.645202	-1.314754	-0.234396
C	-4.205564	0.138648	-0.375933
C	-5.653906	0.459511	-0.654425
H	-6.317839	-0.284073	-0.206450
H	-5.911863	1.454101	-0.286535
H	-5.829809	0.433595	-1.734789
O	-3.904365	-1.115532	-0.548196
O	-3.405756	1.022082	-0.037364

INT18' (Doublet, S**2 after annihilation: 0.7517)
E= -1337.74570042 a.u.

C	3.706387	-1.505736	-0.548505
C	2.237529	-1.473794	-0.269962
C	1.703770	-1.761493	0.942173
C	2.595162	-2.215416	2.061197
H	4.087122	-2.534672	-0.572098
H	2.879114	-3.271892	1.966304
C	0.247392	-1.828048	1.225572
N	-0.545917	-0.934791	0.552503
O	-0.164500	-2.685666	2.031815
C	-1.868830	-1.182666	0.346628
C	-2.744055	-2.105373	0.948660
C	-4.065178	-2.201829	0.545491
H	-2.349708	-2.723862	1.742040
C	-3.692570	-0.481435	-1.063442
C	-4.552881	-1.376805	-0.470736
H	-4.721637	-2.914754	1.034727
H	-3.958569	0.199786	-1.862800
H	-5.584973	-1.413007	-0.798582
O	-1.590265	0.439906	-1.324597
N	-2.399838	-0.405872	-0.672542
Co	-0.038529	0.817086	-0.217878
H	4.290783	-0.977497	0.213236
H	3.915110	-1.055626	-1.521456
H	2.090031	-2.110827	3.024138

H	3.524846	-1.638912	2.102133
O	1.486097	-1.128832	-1.376675
C	1.543335	2.205465	-0.338296
C	0.306358	2.751558	-0.793036
C	-0.614813	2.809444	0.337210
C	0.015823	2.208668	1.436073
C	2.779658	2.019794	-1.145951
H	2.555548	1.750408	-2.183644
H	3.413682	1.231187	-0.728449
H	3.385246	2.934940	-1.178564
C	0.012667	3.285161	-2.150865
H	-1.018743	3.073381	-2.452901
H	0.675038	2.850862	-2.907077
H	0.138212	4.374870	-2.196798
C	-2.015277	3.300649	0.242760
H	-2.587805	3.078673	1.148645
H	-2.542151	2.839716	-0.601036
H	-2.056405	4.385784	0.085491
C	-0.550243	1.942197	2.785451
H	-0.134579	2.617486	3.545243
H	-0.325046	0.921776	3.119069
H	-1.638654	2.059945	2.804315
C	1.343517	1.783154	1.011813
C	2.373667	1.219893	1.924875
H	3.147606	0.667141	1.383175
H	1.936198	0.537714	2.662568
H	2.875092	2.017882	2.490203
C	0.706695	-2.182879	-1.975867
H	0.040418	-1.670949	-2.676288
H	0.081026	-2.666616	-1.214633
C	1.580432	-3.189760	-2.678941
H	2.204262	-2.712690	-3.440300
H	0.960709	-3.946366	-3.170446
H	2.236871	-3.708320	-1.971411

INT21$_{explicit}$ (Doublet, S**2 after annihilation: 0.7501)
E= -1944.67633115 a.u.

C	0	-4.11590700	-0.44064500	0.17402400	H
H	0	-4.11168000	0.64316100	0.27032200	H
H	0	-4.92975500	-0.71227000	-0.49881700	H
H	0	-4.21345400	-0.91760100	1.15009900	H
O	0	-2.86110900	-0.78097800	-0.43786300	H
C	0	-2.69283100	-2.15617700	-0.95023600	H
C	0	-4.01628900	-2.83473500	-1.21842300	H

H	0	-4.60745300	-2.31997500	-1.98103100	H
H	0	-3.81966100	-3.84753200	-1.57451200	H
H	0	-4.60435200	-2.92080300	-0.30520900	H
H	0	-2.18576100	-2.72315500	-0.15949400	H
C	0	-1.80247600	-2.06555800	-2.15121500	H
C	0	-2.00700800	-3.00669600	-3.27837700	H
H	0	-3.05342300	-3.06328600	-3.58874100	H
H	0	-1.39314300	-2.70047800	-4.12332500	H
H	0	-1.69047200	-4.02177000	-2.98710100	H
C	0	-0.58669900	-1.26063800	-2.22779200	H
O	0	0.07780800	-1.24365000	-3.27507300	H
N	0	-0.17562800	-0.57662300	-1.08787200	H
C	0	1.10928700	-0.14526400	-0.96355600	H
C	0	2.21655000	-0.32635300	-1.81933400	H
H	0	2.05869700	-0.82135000	-2.76337400	H
C	0	3.47228100	0.10794600	-1.45247800	H
H	0	4.29997700	-0.08083400	-2.12630600	H
C	0	3.66651500	0.76876000	-0.23768600	H
H	0	4.63858200	1.13450500	0.06855700	H
C	0	2.58507100	0.94492100	0.59118100	H
H	0	2.60406800	1.42621600	1.55950900	H
N	0	1.36030000	0.49803000	0.22647800	H
O	0	0.35644300	0.69404200	1.09570300	H
C	0	-0.72895000	-3.43390900	2.87845800	H
H	0	-0.01162800	-2.98605300	3.56767100	H
H	0	-1.44785300	-4.01558300	3.45626000	H
H	0	-0.16477300	-4.08199900	2.20010800	H
C	0	-1.45768700	-2.36302300	2.09853700	H
O	0	-0.64265600	-1.70903700	1.30132700	H
O	0	-2.66448400	-2.18352400	2.20210800	H
C	0	-2.79092900	3.13341800	1.68827000	H
H	0	-1.99623400	3.87859200	1.75370700	H
H	0	-3.53852900	3.52545500	0.99712100	H
H	0	-3.23027700	2.98955200	2.67451700	H
C	0	-2.23106100	1.85691200	1.16870900	H
O	0	-2.14689600	0.82122900	1.90815800	H
O	0	-1.82344400	1.74269000	-0.03358900	H
H	0	-2.24031100	-9.52004500	3.56100100	L
C	0	-2.08523000	-10.58001600	3.27644000	L
H	0	-2.99698400	-11.15467100	3.54153100	L
H	0	-1.21291200	-10.97822100	3.83701700	L
O	0	-1.85020700	-10.65694500	1.90113400	L
H	0	-1.73091900	-11.61958800	1.69566200	L
H	0	-9.56319100	-9.20656300	-2.40321400	L

C	0	-9.72219300	-8.27894200	-1.81681900	L
H	0	-9.30122700	-7.42115700	-2.38392800	L
H	0	-9.19805200	-8.37840600	-0.84293700	L
O	0	-11.09205800	-8.09580700	-1.60736900	L
H	0	-11.18203600	-7.23818800	-1.11653500	L
H	0	1.26057100	-9.96710300	-2.31845600	L
C	0	0.91195200	-9.70864700	-1.29600600	L
H	0	0.20390900	-8.85594500	-1.34767100	L
H	0	1.78684000	-9.40751000	-0.68556800	L
O	0	0.29202600	-10.81107600	-0.70095600	L
H	0	-0.41653100	-11.10026300	-1.33212000	L
H	0	-3.63648800	-6.26771900	3.30290400	L
C	0	-4.25468400	-5.35909200	3.16468700	L
H	0	-3.81346100	-4.53439300	3.76292400	L
H	0	-5.28350900	-5.57364100	3.52012300	L
O	0	-4.27568600	-5.01806900	1.80926600	L
H	0	-4.76356900	-4.15684800	1.75400700	L
H	0	-6.97920400	-16.20524400	5.40847400	L
C	0	-6.71417000	-15.18083600	5.07605800	L
H	0	-5.61775800	-15.04047900	5.19110000	L
H	0	-7.25657700	-14.44797300	5.71101500	L
O	0	-7.08303600	-15.02302900	3.73709700	L
H	0	-6.82919400	-14.09591300	3.49259600	L
H	0	-8.03855000	-9.57730400	-8.48520800	L
C	0	-7.65014500	-10.34785500	-9.18511600	L
H	0	-6.81612800	-10.90499700	-8.70651400	L
H	0	-7.26548100	-9.84103600	-10.09365400	L
O	0	-8.67277500	-11.22944000	-9.54932200	L
H	0	-8.98230500	-11.65339600	-8.70738700	L
H	0	-7.48828100	-14.99095800	-0.71555700	L
C	0	-7.14923500	-14.43989200	0.18748800	L
H	0	-6.42095900	-13.65409700	-0.10459200	L
H	0	-6.64797300	-15.15228100	0.87306100	L
O	0	-8.24243000	-13.86597700	0.84279000	L
H	0	-8.65499200	-13.24259100	0.19116600	L
H	0	-11.41080100	-9.45239700	-4.27152700	L
C	0	-11.73857700	-10.51330300	-4.24791300	L
H	0	-10.88350700	-11.15650700	-4.53596600	L
H	0	-12.56278900	-10.66786100	-4.97649400	L
O	0	-12.16197300	-10.86020700	-2.96166600	L
H	0	-12.91876400	-10.25490000	-2.74931900	L
H	0	-4.18906300	-6.54711200	-4.67092900	L
C	0	-4.44718500	-5.53636800	-5.05135500	L
H	0	-4.53075400	-4.84369700	-4.19258100	L

H	0	-5.42663400	-5.56554300	-5.57164700	L
O	0	-3.44921500	-5.07868900	-5.91618300	L
H	0	-3.47908000	-5.67394700	-6.70921700	L
H	0	-5.25013000	-14.36104500	-2.70963800	L
C	0	-5.64187700	-13.35187500	-2.95328000	L
H	0	-5.10501400	-12.60347500	-2.33695400	L
H	0	-6.72457100	-13.30450500	-2.71214600	L
O	0	-5.43630400	-13.06748000	-4.30547800	L
H	0	-5.94823900	-13.75155600	-4.80858800	L
H	0	-6.98855800	-6.77603700	-1.35295500	L
C	0	-6.04578900	-6.98817400	-1.89928600	L
H	0	-5.37709800	-7.60480200	-1.26460400	L
H	0	-5.53588300	-6.03024100	-2.12017400	L
O	0	-6.32118200	-7.64928000	-3.09952000	L
H	0	-6.80136300	-8.47984900	-2.84879700	L
H	0	-1.31978300	-11.44224800	-3.94156800	L
C	0	-2.13676000	-11.96038400	-4.48730500	L
H	0	-3.11870300	-11.64364500	-4.07950700	L
H	0	-2.02534200	-13.05208400	-4.33931200	L
O	0	-2.06574000	-11.67560400	-5.85356800	L
H	0	-2.18945300	-10.69527500	-5.93831900	L
H	0	-6.83708900	-3.95980800	-1.55027000	L
C	0	-7.25221400	-3.67130300	-2.53946100	L
H	0	-6.42533500	-3.61768900	-3.27493500	L
H	0	-7.72553700	-2.67105500	-2.46619900	L
O	0	-8.18554200	-4.61897400	-2.96800700	L
H	0	-8.88644100	-4.64777400	-2.26719600	L
H	0	-0.47172300	-15.02214500	-4.32890500	L
C	0	-0.18570300	-15.63406300	-5.21070200	L
H	0	-0.29745100	-16.70636900	-4.95004000	L
H	0	0.87528400	-15.43755300	-5.47517600	L
O	0	-1.01611600	-15.33703000	-6.29558900	L
H	0	-0.87541400	-14.37503600	-6.49302200	L
H	0	-3.59921300	-16.70396600	-5.60862400	L
C	0	-4.05747600	-16.75491300	-4.59755300	L
H	0	-4.21551000	-17.81962000	-4.33031000	L
H	0	-5.04087600	-16.23804900	-4.60490900	L
O	0	-3.20957000	-16.16727600	-3.65444200	L
H	0	-3.12373500	-15.21602500	-3.92117400	L
H	0	-4.09227700	-9.39510600	-0.17512400	L
C	0	-3.52343900	-10.08350700	-0.83470100	L
H	0	-3.75025700	-11.13541100	-0.55953900	L
H	0	-2.44163500	-9.89937600	-0.68880000	L
O	0	-3.84664000	-9.85062300	-2.17410500	L

H	0	-4.81399100	-10.04823200	-2.26104900	L
H	0	-10.31775100	-11.58943000	4.20904800	L
C	0	-9.42484700	-12.14536600	3.85688200	L
H	0	-9.73843400	-12.85789900	3.06351700	L
H	0	-8.98983500	-12.70389500	4.71316000	L
O	0	-8.49044800	-11.23673900	3.35183700	L
H	0	-7.73926100	-11.78183800	3.00188600	L
H	0	-7.57260900	-5.39094800	5.54905000	L
C	0	-7.98901200	-6.03473000	4.74590400	L
H	0	-7.48856800	-7.02578200	4.76172300	L
H	0	-7.79281200	-5.55522100	3.76806200	L
O	0	-9.36905500	-6.18368700	4.90926300	L
H	0	-9.49739300	-6.59535300	5.80196100	L
H	0	-6.39948700	-9.24373900	3.56428500	L
C	0	-5.56642900	-8.93097200	2.90272900	L
H	0	-5.19970200	-9.81826900	2.34268000	L
H	0	-4.74776600	-8.52200900	3.53153900	L
O	0	-6.02900400	-7.95400800	2.01619100	L
H	0	-5.26203300	-7.73275800	1.42734800	L
H	0	-1.24527900	-13.56229700	4.98601200	L
C	0	-1.90207300	-13.41065500	5.86635200	L
H	0	-2.49151700	-12.48161600	5.71589400	L
H	0	-1.27013900	-13.30770600	6.77446300	L
O	0	-2.75582500	-14.51052600	5.99496100	L
H	0	-3.32749900	-14.32314100	6.78386000	L
H	0	1.95085600	-11.05333500	2.73451100	L
C	0	1.61109700	-9.99679200	2.71262400	L
H	0	1.15810200	-9.74434100	3.69216300	L
H	0	0.84409600	-9.86508500	1.92008300	L
O	0	2.69923000	-9.15218100	2.48120500	L
H	0	3.02290500	-9.36635200	1.56882500	L
H	0	-6.33689900	-8.93059100	-6.43488500	L
C	0	-5.22853800	-8.97483400	-6.50021700	L
H	0	-4.84432300	-7.96330500	-6.73734000	L
H	0	-4.92486300	-9.66694200	-7.31468900	L
O	0	-4.68999200	-9.39454500	-5.28061400	L
H	0	-5.03441800	-10.31264400	-5.13171900	L
H	0	-2.69046600	-7.31866200	-2.00632900	H
C	0	-2.52536700	-6.28093700	-1.69992700	H
H	0	-3.00657200	-6.13552600	-0.72419800	H
H	0	-3.04065600	-5.64191800	-2.43464400	H
O	0	-1.14433000	-6.02797600	-1.66053100	H
H	0	-0.98044300	-5.36826600	-0.96603900	H
H	0	-3.04319700	-13.75811200	-7.92987300	L

C	0	-3.97884600	-13.99700100	-7.38155200	L
H	0	-3.75500600	-14.13114300	-6.30157500	L
H	0	-4.39319300	-14.94491500	-7.78101000	L
O	0	-4.91532400	-12.97435500	-7.55986400	L
H	0	-4.51848100	-12.16484300	-7.14642700	L
H	0	-9.78991800	-12.59777300	-2.55734700	L
C	0	-9.82140400	-13.65958600	-2.88190200	L
H	0	-9.67608200	-14.32537000	-2.00433200	L
H	0	-8.99925100	-13.83496200	-3.60407300	L
O	0	-11.04174400	-13.94063300	-3.50326400	L
H	0	-11.74204700	-13.76974900	-2.82181200	L
H	0	-2.76912700	-14.00452700	2.29627200	L
C	0	-3.85405300	-13.87813900	2.48687000	L
H	0	-4.00212400	-13.66998100	3.56771400	L
H	0	-4.37959800	-14.81708200	2.21000600	L
O	0	-4.33391200	-12.81414000	1.71720800	L
H	0	-5.28584000	-12.70019100	1.97100600	L
H	0	0.53245400	-12.90782400	1.13840400	L
C	0	0.58966400	-13.57509700	2.02163800	L
H	0	0.11996500	-13.06001200	2.88665300	L
H	0	1.65848800	-13.78365800	2.24334200	L
O	0	-0.08607300	-14.76661000	1.74165900	L
H	0	-0.04257900	-15.30693900	2.57279100	L
H	0	-10.83436900	-7.30036800	2.87038500	L
C	0	-10.61707900	-8.23892900	2.31718400	L
H	0	-11.18489000	-8.22820600	1.36491300	L
H	0	-10.94266000	-9.11458400	2.91977700	L
O	0	-9.25104600	-8.32810800	2.03439400	L
H	0	-8.79213500	-8.35868000	2.91292300	L
H	0	-8.95468900	-16.51030300	-3.82031700	L
C	0	-8.02716800	-16.80436700	-3.28783900	L
H	0	-8.28157900	-17.04103600	-2.23205200	L
H	0	-7.31137700	-15.95530900	-3.31998000	L
O	0	-7.47476800	-17.92457100	-3.91620200	L
H	0	-6.66735800	-18.16223400	-3.39103800	L
H	0	-10.31923000	-7.50598600	-5.56585000	L
C	0	-9.22176600	-7.65673200	-5.60733300	L
H	0	-8.95386500	-8.07835800	-6.59946200	L
H	0	-8.92866000	-8.36787700	-4.80719500	L
O	0	-8.58319600	-6.42876400	-5.41778800	L
H	0	-7.61138000	-6.61657300	-5.47680500	L
H	0	-10.46991600	-11.03738800	-0.66845800	L
C	0	-11.03984200	-11.24227800	0.26188900	L
H	0	-11.91302200	-10.55752800	0.32439600	L

H	0	-10.37756800	-11.05911700	1.13128500	L
O	0	-11.46658700	-12.57343800	0.28319600	L
H	0	-12.05792600	-12.68293100	-0.50586700	L
H	0	-14.45754700	-6.50221900	-4.03667800	L
C	0	-14.16062600	-7.56809600	-3.93652900	L
H	0	-15.00287500	-8.13390700	-3.48856100	L
H	0	-13.27894100	-7.64965500	-3.26498800	L
O	0	-13.86572000	-8.10392800	-5.19363900	L
H	0	-13.05635900	-7.62643700	-5.51161300	L
H	0	-1.61188000	-13.36608500	-0.32833800	L
C	0	-2.18058500	-13.80890200	-1.17358000	L
H	0	-1.50166300	-14.41354200	-1.81223400	L
H	0	-2.60446200	-12.98777500	-1.78398300	L
O	0	-3.22214200	-14.60547900	-0.69021300	L
H	0	-2.79129400	-15.32513700	-0.16015400	L
H	0	-7.15401300	-11.64512600	-6.21206400	L
C	0	-8.14846900	-11.50226400	-5.73762000	L
H	0	-8.71834800	-12.44954300	-5.81108300	L
H	0	-8.70939400	-10.70595200	-6.26992900	L
O	0	-7.99888200	-11.16254700	-4.39111500	L
H	0	-7.45433900	-10.33423100	-4.37811500	L
H	0	-6.58433200	-15.62819300	-6.62110600	L
C	0	-7.68007200	-15.45480500	-6.67440100	L
H	0	-8.01369500	-14.88719100	-5.77978500	L
H	0	-8.19672000	-16.43624500	-6.68472000	L
O	0	-8.00193100	-14.75388600	-7.84090600	L
H	0	-7.54611400	-13.87582800	-7.76775400	L
H	0	0.49809500	-13.03149000	-2.81687300	L
C	0	1.48023100	-13.42984100	-2.49168500	L
H	0	2.16003600	-12.57612300	-2.28710600	L
H	0	1.89985100	-14.05702900	-3.30719800	L
O	0	1.30533700	-14.19606900	-1.33559300	L
H	0	2.20996500	-14.50872600	-1.07453700	L
H	0	-1.23594300	-6.74904300	-4.62616400	L
C	0	-1.35693400	-7.74777600	-5.09797000	L
H	0	-0.40915000	-8.04890000	-5.59358300	L
H	0	-2.15440400	-7.68878900	-5.86471200	L
O	0	-1.72172100	-8.69153600	-4.13502400	L
H	0	-0.96271900	-8.74040400	-3.49938100	L
H	0	-12.33935900	-12.99346300	-7.91928700	L
C	0	-11.71007500	-13.13538700	-7.01699300	L
H	0	-12.35101400	-13.03724600	-6.11434300	L
H	0	-10.92273300	-12.35262500	-7.00589300	L
O	0	-11.12663600	-14.40521300	-7.05879300	L

H	0	-10.58287900	-14.48289800	-6.23263000	L
H	0	-0.47962500	-11.29734300	-9.89337400	L
C	0	-0.97933300	-11.40449300	-8.90642700	L
H	0	-0.51953200	-10.69039300	-8.19379900	L
H	0	-0.83281700	-12.43642300	-8.52051600	L
O	0	-2.34373700	-11.12052300	-9.02194800	L
H	0	-2.70529000	-11.77465700	-9.67448700	L
H	0	-9.07747500	-4.20200000	0.38034800	L
C	0	-8.82677200	-5.22370800	0.73475100	L
H	0	-9.15243000	-5.95543400	-0.03012000	L
H	0	-9.36513900	-5.43559400	1.68224200	L
O	0	-7.44792600	-5.34329500	0.92746300	L
H	0	-7.20287900	-4.63533300	1.57670800	L
H	0	-2.64329700	-7.76907400	1.42648900	H
C	0	-1.61625100	-7.46133400	1.18173700	H
H	0	-1.38355600	-7.79928100	0.17098400	H
H	0	-0.92829100	-7.96774000	1.87186000	H
O	0	-1.44960400	-6.06030000	1.17526700	H
H	0	-1.57525300	-5.73400900	2.06996700	H
H	0	-6.20956800	-17.86082100	-0.64510000	L
C	0	-5.22036300	-17.37461500	-0.76892200	L
H	0	-4.89043700	-16.97965000	0.21636000	L
H	0	-5.32223600	-16.53936800	-1.49398300	L
O	0	-4.30179500	-18.31527200	-1.24453800	L
H	0	-3.43614500	-17.83639200	-1.32246100	L
H	0	-10.95786900	-9.83674800	-7.10100100	L
C	0	-11.47387200	-9.32019200	-7.93751100	L
H	0	-10.81791200	-9.34558000	-8.83116000	L
H	0	-11.66266900	-8.26117400	-7.66171200	L
O	0	-12.67910700	-9.96351000	-8.23420100	L
H	0	-13.22479500	-9.91694100	-7.40689600	L
H	0	-7.88939700	-10.46661300	0.80082000	L
C	0	-7.15304000	-10.45714600	-0.02584100	L
H	0	-6.26880400	-11.05801100	0.27313000	L
H	0	-6.84761500	-9.40682000	-0.21686100	L
O	0	-7.74131400	-11.00496400	-1.16793400	L
H	0	-7.06408700	-10.92222700	-1.88614900	L
H	0	-8.68047800	-0.19515200	0.41458600	L
C	0	-7.89701200	-0.72870100	0.98779700	L
H	0	-8.32497000	-1.05843100	1.95735600	L
H	0	-7.04926400	-0.03371400	1.16960600	L
O	0	-7.46769000	-1.83401000	0.24913300	L
H	0	-6.75260500	-2.26082100	0.78712900	L
H	0	-7.86162800	6.40846600	-2.89012000	L

C	0	-7.82841700	5.30424400	-2.97385400	L
H	0	-8.86864500	4.92113700	-3.04403800	L
H	0	-7.33547600	4.88989000	-2.06784600	L
O	0	-7.10763000	4.95584500	-4.11917700	L
H	0	-7.10482200	3.96448000	-4.14939900	L
H	0	-4.80391300	4.72472400	-7.54481000	L
C	0	-5.04133000	5.80874600	-7.50298400	L
H	0	-6.14064300	5.95285800	-7.55085000	L
H	0	-4.57731700	6.31098700	-8.37603200	L
O	0	-4.53432600	6.36913700	-6.32730500	L
H	0	-4.98267300	5.89206300	-5.58221400	L
H	0	-7.82155800	9.87233200	-2.27811800	L
C	0	-8.88509500	10.15961800	-2.39227300	L
H	0	-9.19381700	10.74870800	-1.50309500	L
H	0	-9.49682000	9.23538800	-2.46118300	L
O	0	-9.03074500	10.92717700	-3.55151100	L
H	0	-9.99920000	11.12965200	-3.62564100	L
H	0	-6.87270200	-3.72697600	-10.74461000	L
C	0	-7.29103800	-2.81586000	-10.27222000	L
H	0	-8.22374500	-3.08803100	-9.73487000	L
H	0	-7.51721500	-2.07487400	-11.06844100	L
O	0	-6.35635000	-2.29183000	-9.37531300	L
H	0	-6.77222200	-1.47574000	-8.99502600	L
H	0	-14.51422000	1.91591300	2.13148700	L
C	0	-14.79175000	2.66845600	1.36525600	L
H	0	-13.86605100	3.15688700	0.99242800	L
H	0	-15.45345300	3.42895000	1.83316400	L
O	0	-15.45790400	2.03011700	0.31490100	L
H	0	-15.68497200	2.74294300	-0.33646300	L
H	0	-3.55412300	-4.03012300	-9.36895600	L
C	0	-3.49843300	-3.69104000	-10.42588000	L
H	0	-3.64130100	-2.59095600	-10.47613400	L
H	0	-4.30932200	-4.18223700	-11.00101600	L
O	0	-2.26543800	-4.04142600	-10.98333300	L
H	0	-1.57521400	-3.61557500	-10.41177700	L
H	0	-5.29728300	-0.15811700	-10.75595200	L
C	0	-4.77073200	0.08388500	-11.70228200	L
H	0	-3.70373100	-0.20862100	-11.61993300	L
H	0	-4.82853800	1.17773800	-11.87402100	L
O	0	-5.37225500	-0.58432200	-12.77283000	L
H	0	-5.29601000	-1.55231300	-12.56761600	L
H	0	-14.25023700	-0.30721700	-7.06030500	L
C	0	-13.77970600	0.65435700	-7.35386000	L
H	0	-14.56436400	1.38407300	-7.63990900	L

H	0	-13.12254800	0.47756900	-8.22858800	L
O	0	-13.01550500	1.16022700	-6.29927900	L
H	0	-13.64151900	1.28369300	-5.54071100	L
H	0	-7.24827300	1.46798100	-4.93384800	L
C	0	-7.52171800	0.39971600	-5.04840600	L
H	0	-7.49184700	0.13875700	-6.12730600	L
H	0	-8.54660300	0.24826000	-4.65044400	L
O	0	-6.61173700	-0.38592400	-4.33702700	L
H	0	-6.88840500	-1.32615000	-4.48242500	L
H	0	-11.63653800	4.08894800	-9.19161500	L
C	0	-11.30957200	3.03109900	-9.27258900	L
H	0	-11.50492000	2.52243600	-8.30787700	L
H	0	-10.22018700	2.99015900	-9.48061900	L
O	0	-12.01797700	2.38300800	-10.28804200	L
H	0	-11.81659200	2.87951200	-11.12298200	L
H	0	-14.16788400	-2.80374400	-5.28373100	L
C	0	-13.65756500	-2.05957300	-4.63849900	L
H	0	-13.56717800	-2.47696400	-3.61290400	L
H	0	-14.26828800	-1.13142300	-4.61094500	L
O	0	-12.38945700	-1.79185900	-5.16356500	L
H	0	-11.99038500	-1.09827400	-4.57755600	L
H	0	-6.94282500	8.06713300	-8.71323700	L
C	0	-7.78748600	7.65384200	-9.30323900	L
H	0	-8.44779800	7.05209200	-8.64323900	L
H	0	-7.37773900	6.99475200	-10.09377700	L
O	0	-8.51313400	8.68926100	-9.89941600	L
H	0	-8.82878000	9.26471600	-9.15525400	L
H	0	-12.45517900	-4.92631500	-3.02353400	L
C	0	-11.60125500	-4.95327400	-3.73081700	L
H	0	-10.97733300	-5.84390900	-3.50437600	L
H	0	-11.00135600	-4.02836600	-3.59866500	L
O	0	-12.08923500	-5.02527100	-5.03868200	L
H	0	-11.29229300	-5.09195200	-5.62540500	L
H	0	-12.79283800	-5.89725500	-7.72633700	L
C	0	-13.84115200	-6.02386900	-8.07234800	L
H	0	-14.15932800	-7.07901100	-7.92965600	L
H	0	-13.89129600	-5.77968200	-9.15314100	L
O	0	-14.68973200	-5.16437100	-7.36786700	L
H	0	-14.59290600	-5.40681000	-6.41094000	L
H	0	-9.37429600	1.39279100	-11.43137100	L
C	0	-8.27344800	1.42581000	-11.28361600	L
H	0	-7.92657800	0.42608700	-10.95299300	L
H	0	-8.02317400	2.16671100	-10.49495800	L
O	0	-7.63661900	1.76018200	-12.48242100	L

H	0	-7.98348600	2.65430900	-12.73634100	L
H	0	-10.20427900	-3.08386500	-12.76778000	L
C	0	-10.70570000	-2.77515400	-11.82517700	L
H	0	-10.40882800	-3.47528500	-11.01884100	L
H	0	-11.80886700	-2.82351300	-11.95109100	L
O	0	-10.31624300	-1.47943200	-11.47381900	L
H	0	-10.59903500	-0.89538000	-12.22438900	L
H	0	-8.75886100	2.90075200	-7.32926900	L
C	0	-8.29063600	3.90772100	-7.31607500	L
H	0	-8.04387000	4.22227100	-8.35286800	L
H	0	-7.35504000	3.85378700	-6.72544500	L
O	0	-9.15136400	4.83470300	-6.72273300	L
H	0	-9.95956100	4.86240600	-7.29612700	L
H	0	-15.44030600	4.81809100	-1.99567500	L
C	0	-14.40174400	5.18920000	-1.88084300	L
H	0	-13.78560400	4.79726600	-2.71720100	L
H	0	-13.99425500	4.82295300	-0.91528000	L
O	0	-14.40955000	6.58626100	-1.89889200	L
H	0	-13.46099900	6.86452600	-1.82062600	L
H	0	-13.18606400	10.05459400	-0.78957300	L
C	0	-12.60856500	9.95544200	-1.73421300	L
H	0	-12.06273000	8.98776700	-1.74065600	L
H	0	-11.87130900	10.78162900	-1.78816800	L
O	0	-13.46456800	10.03421400	-2.83682300	L
H	0	-14.12722400	9.30538000	-2.72054200	L
H	0	-12.00394500	6.25518700	-3.87890100	L
C	0	-11.10581800	6.88675400	-4.02999700	L
H	0	-10.42843800	6.37500600	-4.74651800	L
H	0	-10.59155200	7.01735200	-3.05403100	L
O	0	-11.49971800	8.12839500	-4.53720400	L
H	0	-10.66381800	8.63885100	-4.69299700	L
H	0	-6.69758400	2.09316100	-2.47934300	L
C	0	-7.25185400	1.55037600	-1.68534500	L
H	0	-6.93252000	1.91733400	-0.68809600	L
H	0	-7.01510900	0.46936000	-1.75668100	L
O	0	-8.62767800	1.73062500	-1.85125400	L
H	0	-8.79232000	2.70244800	-1.74200500	L
H	0	-4.67421100	3.74973800	-3.04012400	L
C	0	-4.28654200	4.05186400	-2.04650100	L
H	0	-4.70507000	3.36975700	-1.27699900	L
H	0	-4.60892300	5.09547300	-1.84152800	L
O	0	-2.89176200	3.96302500	-2.05333400	L
H	0	-2.58960600	4.33316700	-1.18472300	L
H	0	-8.35157000	-3.99429000	-7.21095300	L

C	0	-8.14164700	-3.28549900	-6.38537000	L
H	0	-7.84818700	-2.30754100	-6.82098600	L
H	0	-7.30838100	-3.68852400	-5.77113700	L
O	0	-9.29156600	-3.14041500	-5.60432700	L
H	0	-9.06318500	-2.48538200	-4.89563400	L
H	0	-13.16677600	2.47331300	-1.61112000	L
C	0	-12.57980300	1.53598000	-1.51916200	L
H	0	-12.68530500	1.12747100	-0.49242000	L
H	0	-11.51085000	1.76453900	-1.70240000	L
O	0	-13.01887900	0.60075000	-2.46070500	L
H	0	-13.95702300	0.38800700	-2.21797400	L
H	0	-9.07751100	6.08894500	-11.92209800	L
C	0	-9.78792700	5.41840700	-11.39199100	L
H	0	-9.52716900	5.37136400	-10.31302500	L
H	0	-9.70549000	4.39951800	-11.82202500	L
O	0	-11.09827400	5.87958200	-11.55069700	L
H	0	-11.11693900	6.78979600	-11.15609000	L
H	0	-14.42327900	3.34988000	-9.14040600	L
C	0	-15.02045700	4.05999900	-9.75050000	L
H	0	-15.82029500	4.51113600	-9.12435300	L
H	0	-15.49143100	3.50279600	-10.58590000	L
O	0	-14.19434700	5.06010500	-10.27252900	L
H	0	-13.79502500	5.52054400	-9.48961700	L
H	0	-9.47306600	2.78917400	-4.49860100	L
C	0	-10.54564300	2.57546400	-4.67903100	L
H	0	-10.76853000	1.54017100	-4.34459400	L
H	0	-10.75089600	2.66595100	-5.76623400	L
O	0	-11.32176000	3.49263100	-3.96575600	L
H	0	-12.26579600	3.23183100	-4.12291000	L
H	0	-4.31628200	1.05109100	-2.37265900	L
C	0	-3.84288700	0.71131800	-3.31427100	L
H	0	-4.32437100	1.23561400	-4.16654100	L
H	0	-3.99380200	-0.38455700	-3.41364300	L
O	0	-2.47927800	1.01098600	-3.27465300	L
H	0	-2.09300900	0.62454300	-4.10277700	L
H	0	-16.28872300	7.93901700	-3.63722900	L
C	0	-16.18624500	7.45895100	-4.63413400	L
H	0	-17.02772500	7.78788100	-5.27758500	L
H	0	-16.23170200	6.35392700	-4.52422700	L
O	0	-14.97906000	7.83902900	-5.22841300	L
H	0	-14.26205700	7.47449700	-4.64783500	L
H	0	-17.43927500	-1.82066900	-8.20599000	L
C	0	-16.38388800	-2.07471200	-8.43359000	L
H	0	-15.87983000	-2.38282500	-7.49208800	L

H	0	-15.88340600	-1.17526400	-8.85157300	L
O	0	-16.35377700	-3.11937100	-9.36261200	L
H	0	-15.39457000	-3.30345800	-9.53682400	L
H	0	-12.94928800	-2.99760800	0.14414400	L
C	0	-13.78938700	-2.27320100	0.16009600	L
H	0	-14.68210800	-2.77166600	0.59583600	L
H	0	-14.00763100	-1.95475100	-0.88222600	L
O	0	-13.42748300	-1.16802000	0.93594900	L
H	0	-14.20936100	-0.55751600	0.92411500	L
H	0	-14.88388000	2.61374700	-3.74499800	L
C	0	-15.90109600	2.16694400	-3.76109600	L
H	0	-16.28696600	2.07681800	-2.72331200	L
H	0	-15.83952000	1.15327200	-4.20705200	L
O	0	-16.76347700	2.95513500	-4.52895100	L
H	0	-16.77078200	3.84965300	-4.09998600	L
H	0	-8.81373900	0.52473200	-8.24742000	L
C	0	-9.70127200	-0.12891800	-8.37759600	L
H	0	-9.37500200	-1.16769200	-8.59504000	L
H	0	-10.29593000	0.23967000	-9.23684200	L
O	0	-10.49422900	-0.09664600	-7.22744900	L
H	0	-9.96948100	-0.54752100	-6.51799100	L
H	0	-10.64383900	7.62292600	-7.32487700	L
C	0	-11.46536900	7.56085000	-8.06707800	L
H	0	-11.46519500	6.54472200	-8.51586900	L
H	0	-11.29124000	8.31975900	-8.85966200	L
O	0	-12.68295400	7.80613900	-7.42523900	L
H	0	-13.38285000	7.72102600	-8.12331000	L
H	0	-6.34262800	-7.51247500	-9.06625800	L
C	0	-5.87187500	-6.51382000	-9.16829300	L
H	0	-6.16980300	-6.07680000	-10.14495700	L
H	0	-4.76779700	-6.63290800	-9.13128500	L
O	0	-6.30585900	-5.69517700	-8.12196300	L
H	0	-5.82139800	-4.83580700	-8.22660500	L
H	0	-12.81018400	-0.17215100	-10.79337300	L
C	0	-13.75759900	-0.44219800	-11.30620500	L
H	0	-14.19212800	-1.34990700	-10.83577100	L
H	0	-13.53504800	-0.66325500	-12.37009400	L
O	0	-14.66477900	0.61968000	-11.23768000	L
H	0	-14.81609500	0.78841000	-10.27193400	L
H	0	-6.00891800	2.70582700	-8.88186700	L
C	0	-5.59599800	1.77672800	-8.44307700	L
H	0	-4.61577300	1.56526600	-8.92001200	L
H	0	-6.30161100	0.94303500	-8.64128900	L
O	0	-5.43762800	1.95328300	-7.06645000	L

H	0	-5.02542500	1.11707000	-6.72913500	L
H	0	-10.16908600	-2.61753800	-1.13367000	L
C	0	-10.66862000	-1.71370200	-1.53466900	L
H	0	-10.89873800	-1.02739600	-0.69225300	L
H	0	-11.61040800	-2.02063200	-2.03662300	L
O	0	-9.81784000	-1.08667900	-2.44837000	L
H	0	-10.31605900	-0.30082300	-2.78940200	L
H	0	-18.46501600	2.16442100	-8.26387800	L
C	0	-17.49240600	1.78203300	-7.89238000	L
H	0	-16.90483000	2.63436700	-7.48874100	L
H	0	-16.94494800	1.31564200	-8.73983500	L
O	0	-17.72407600	0.83737700	-6.88764100	L
H	0	-16.82945800	0.52398100	-6.59456100	L
H	0	-7.93144900	8.27009900	-4.55327000	L
C	0	-7.77998000	8.19093500	-5.65025700	L
H	0	-8.36804900	7.33241500	-6.02951200	L
H	0	-6.70666000	8.01158800	-5.86961700	L
O	0	-8.21360600	9.36023400	-6.28190500	L
H	0	-7.64239300	10.08995200	-5.92732900	L
H	0	-4.84195000	-2.59101800	-5.57254000	L
C	0	-4.53619600	-1.98970000	-6.45426400	L
H	0	-5.39697500	-1.37324000	-6.78098300	L
H	0	-4.24563600	-2.66272200	-7.29001600	L
O	0	-3.47455000	-1.14816500	-6.11057300	L
H	0	-2.69161700	-1.73934100	-5.96603000	L
H	0	-13.31225800	-2.61478600	-7.91473000	L
C	0	-12.43057100	-2.75515700	-8.57138200	L
H	0	-12.12449200	-1.76769000	-8.97483200	L
H	0	-12.70906600	-3.43008500	-9.40874200	L
O	0	-11.38978200	-3.31546500	-7.82637100	L
H	0	-10.62430900	-3.40414600	-8.45060900	L
H	0	-9.21208000	-7.23715500	-9.12981900	L
C	0	-9.67915700	-6.22919400	-9.10064000	L
H	0	-8.89865900	-5.47077000	-9.30942400	L
H	0	-10.10242600	-6.03875600	-8.09084000	L
O	0	-10.68166800	-6.13166100	-10.07039100	L
H	0	-11.34722900	-6.83291500	-9.84845100	L
H	0	-14.77966500	3.70290400	-6.49600600	L
C	0	-14.18391600	4.60635100	-6.25732800	L
H	0	-14.24073800	4.79101700	-5.16357900	L
H	0	-14.60922900	5.47197900	-6.80836700	L
O	0	-12.85842400	4.39643200	-6.64572800	L
H	0	-12.35432700	5.19357600	-6.33947100	L
H	0	1.93540400	-3.39972500	8.15622100	L

C	0	2.22297700	-3.84248300	7.17883500	L
H	0	3.27368000	-3.56926600	6.95543000	L
H	0	1.57227400	-3.43638900	6.37610700	L
O	0	2.11616700	-5.23503100	7.22898000	L
H	0	1.17344000	-5.42994200	7.46449500	L
H	0	-4.13849800	-6.77187600	7.75380200	L
C	0	-4.04666500	-7.50944400	6.93060100	L
H	0	-5.03754800	-7.63275900	6.44272100	L
H	0	-3.71448400	-8.47942300	7.35758500	L
O	0	-3.11080500	-7.04618100	6.00298200	L
H	0	-3.04064300	-7.75283900	5.31044600	L
H	0	-6.64670400	-11.88084200	8.23305600	L
C	0	-7.16525200	-12.79698400	8.58659300	L
H	0	-7.96008600	-13.07835400	7.86271000	L
H	0	-7.63307400	-12.58451200	9.56914900	L
O	0	-6.25237500	-13.84586000	8.73322700	L
H	0	-5.82882000	-13.96725000	7.84448500	L
H	0	-8.12180300	-1.45700500	14.40941800	L
C	0	-7.11077400	-1.00234800	14.37621300	L
H	0	-6.89107300	-0.70058600	13.33021600	L
H	0	-6.36707200	-1.75619800	14.71376100	L
O	0	-7.08340500	0.11505800	15.21589500	L
H	0	-6.14863600	0.44624200	15.19885400	L
H	0	0.19088400	-2.01936300	14.95563600	L
C	0	1.14169800	-1.44575700	14.94238500	L
H	0	1.07898300	-0.60062400	15.66110600	L
H	0	1.29546800	-1.03870700	13.92260900	L
O	0	2.21561900	-2.27791600	15.27143600	L
H	0	2.02067700	-2.63050900	16.17818100	L
H	0	2.32522400	-12.92785400	4.84692000	L
C	0	1.85610700	-12.20749100	5.55150400	L
H	0	0.82158800	-11.97834100	5.21839600	L
H	0	2.44770700	-11.26937500	5.54848100	L
O	0	1.83958600	-12.73657600	6.84560900	L
H	0	1.31309600	-13.57578400	6.79098600	L
H	0	-5.84952700	-2.84147600	7.13386700	L
C	0	-4.89818300	-3.33716100	7.42422500	L
H	0	-5.06805100	-3.98832600	8.30743200	L
H	0	-4.15572000	-2.55981400	7.69308600	L
O	0	-4.40176400	-4.09334100	6.35979400	L
H	0	-5.11660300	-4.74130800	6.12930000	L
H	0	-7.06007600	-8.07255100	13.60829900	L
C	0	-6.88962900	-8.84555500	14.38686100	L
H	0	-5.82260200	-8.83743800	14.69682200	L

H	0	-7.12955800	-9.84076800	13.95977300	L
O	0	-7.71489800	-8.60639300	15.48985500	L
H	0	-7.45078900	-7.71657700	15.84081200	L
H	0	-9.44712200	-2.87463800	9.93077500	L
C	0	-9.64320100	-3.08059900	11.00456400	L
H	0	-9.05934500	-3.97166400	11.31010200	L
H	0	-10.72358200	-3.29337200	11.15471800	L
O	0	-9.25240500	-1.98811300	11.78427700	L
H	0	-9.80272700	-1.22234700	11.47635700	L
H	0	-0.50104400	3.30469700	5.06073400	L
C	0	-1.30748800	2.58746700	5.32139100	L
H	0	-2.28623000	3.06326500	5.11158400	L
H	0	-1.25599100	2.33580600	6.40225100	L
O	0	-1.18755600	1.42737300	4.55226300	L
H	0	-0.29367200	1.05138100	4.75907800	L
H	0	-5.76504000	-6.02273500	12.60536200	L
C	0	-4.81288000	-6.45119800	12.22791400	L
H	0	-4.65369000	-7.44055800	12.70023300	L
H	0	-3.96813400	-5.78712700	12.50445100	L
O	0	-4.86952200	-6.60830000	10.84067400	L
H	0	-4.99603700	-5.69896500	10.46670300	L
H	0	-3.63696600	-0.34739700	16.14850300	L
C	0	-2.86374400	-1.00906400	15.70168400	L
H	0	-2.95694100	-2.03314300	16.12281900	L
H	0	-1.86081400	-0.60865200	15.95468400	L
O	0	-3.00594200	-1.04451000	14.31191300	L
H	0	-3.90986000	-1.41420800	14.14075400	L
H	0	-0.24245000	-4.14828500	13.48166800	L
C	0	-1.27818100	-3.95749500	13.13117700	L
H	0	-1.89871600	-3.58788800	13.97454400	L
H	0	-1.70599700	-4.91085900	12.76493800	L
O	0	-1.27192500	-3.02492600	12.08995000	L
H	0	-0.86286100	-2.20156300	12.46302900	L
H	0	-4.07079400	1.92201400	14.63015500	L
C	0	-4.40267100	2.39859100	13.68266800	L
H	0	-3.75898200	3.27950600	13.48260600	L
H	0	-5.45662700	2.73697600	13.78255700	L
O	0	-4.28590100	1.49666900	12.62175900	L
H	0	-4.86148000	0.72393700	12.85526300	L
H	0	-11.93805700	-2.90544500	2.78473900	L
C	0	-10.88554500	-2.56723900	2.69721300	L
H	0	-10.80576800	-1.53710300	3.10265300	L
H	0	-10.59670700	-2.56987000	1.62424800	L
O	0	-10.06428900	-3.43355600	3.42412200	L

H	0	-9.13742300	-3.10571400	3.29389100	L
H	0	0.26023300	-5.70233500	11.12227600	L
C	0	1.11187300	-6.38880000	11.31513900	L
H	0	0.73560300	-7.36723600	11.68080700	L
H	0	1.75685200	-5.94848100	12.10001800	L
O	0	1.86158900	-6.56313900	10.14898400	L
H	0	1.25316500	-6.99702800	9.49668300	L
H	0	-0.96098900	-8.29474800	9.78224200	L
C	0	-1.94855700	-8.56308900	10.21267800	L
H	0	-2.09287500	-9.65733400	10.11575700	L
H	0	-2.75472100	-8.04301100	9.65429600	L
O	0	-1.99586300	-8.21384000	11.56451700	L
H	0	-1.87667700	-7.22999300	11.59716900	L
H	0	-1.83530100	-4.37179000	16.56303800	L
C	0	-0.77706300	-4.48236600	16.88156300	L
H	0	-0.10950300	-4.38833000	15.99973400	L
H	0	-0.64063500	-5.48975000	17.32524900	L
O	0	-0.45540400	-3.51218300	17.83564700	L
H	0	-0.59544600	-2.63550400	17.39256600	L
H	0	-0.85973900	-8.61783400	7.18985000	L
C	0	-0.79070400	-9.63681700	6.75103100	L
H	0	0.01853800	-9.66260100	5.99127700	L
H	0	-1.75216800	-9.87977800	6.25656900	L
O	0	-0.54016600	-10.58010500	7.75090700	L
H	0	0.31629400	-10.31010200	8.17084300	L
H	0	-3.46484200	-11.97141400	10.34849700	L
C	0	-3.10227500	-12.47340400	9.42619100	L
H	0	-3.40884900	-13.53881400	9.45560900	L
H	0	-1.99321800	-12.42320200	9.38050500	L
O	0	-3.66047300	-11.86726300	8.29726200	L
H	0	-3.33698100	-10.92974800	8.30040200	L
H	0	0.99832800	-7.25459000	5.10815000	L
C	0	0.13523100	-6.90255900	4.50594400	L
H	0	-0.55621000	-7.75231900	4.34036000	L
H	0	0.49144600	-6.53364000	3.52062500	L
O	0	-0.54131500	-5.88897100	5.18854700	L
H	0	0.08952100	-5.12580200	5.23668400	L
H	0	-9.63084400	-8.94727400	7.11886900	L
C	0	-8.97886200	-9.48907300	6.40004500	L
H	0	-9.32754400	-9.27190400	5.36984400	L
H	0	-9.04792800	-10.58404900	6.57901100	L
O	0	-7.65568400	-9.06043800	6.53513900	L
H	0	-7.38228400	-9.30304200	7.45688900	L
H	0	-3.84051100	-0.64446700	9.47027100	L

C	0	-2.73585300	-0.67843700	9.56649100	L
H	0	-2.28829700	-0.13549100	8.70993500	L
H	0	-2.38917400	-1.73427600	9.54269900	L
O	0	-2.33677400	-0.06422200	10.75675400	L
H	0	-2.73797200	-0.59925900	11.48881100	L
H	0	0.26859500	1.48496900	14.33542500	L
C	0	-0.66668100	1.43656100	13.74271400	L
H	0	-1.52294800	1.65481100	14.41572800	L
H	0	-0.77265800	0.41541800	13.31759700	L
O	0	-0.60614600	2.38105800	12.71486700	L
H	0	-1.46755100	2.30811200	12.22928400	L
H	0	-0.70001400	-4.25992800	8.86998100	L
C	0	-1.49685800	-4.98113700	8.58642500	L
H	0	-1.10706300	-6.01927500	8.65207300	L
H	0	-1.80418300	-4.78673200	7.53990100	L
O	0	-2.60476200	-4.82559700	9.42284100	L
H	0	-2.29008200	-5.04548500	10.33673200	L
H	0	-9.61610200	-7.40702200	13.38315000	L
C	0	-9.92657000	-7.00148000	12.39676000	L
H	0	-10.33044100	-5.97324400	12.51971100	L
H	0	-10.72530200	-7.64958500	11.98285300	L
O	0	-8.83977600	-6.99349300	11.51776000	L
H	0	-8.17180200	-6.37638900	11.91332600	L
H	0	-9.48018700	-10.75143200	9.23854100	L
C	0	-9.36547400	-9.98581700	10.03243600	L
H	0	-10.32230900	-9.43021600	10.13160700	L
H	0	-8.55424800	-9.28563900	9.73978300	L
O	0	-9.04745700	-10.61884000	11.23807600	L
H	0	-8.93357800	-9.89443700	11.90635700	L
H	0	-6.83505100	-0.44464900	8.13126800	L
C	0	-6.88143000	-0.65297800	9.21855200	L
H	0	-6.33977600	-1.59961200	9.42143900	L
H	0	-7.94483400	-0.76151300	9.51678500	L
O	0	-6.28965100	0.40427800	9.91563300	L
H	0	-6.37725400	0.17828500	10.87703100	L
H	0	-7.67063100	-5.53884600	14.31526100	L
C	0	-8.25839200	-4.70084000	14.74687600	L
H	0	-8.87122000	-4.22183600	13.95321500	L
H	0	-7.55605600	-3.94872500	15.15723700	L
O	0	-9.07934200	-5.16688500	15.77818500	L
H	0	-9.69216800	-5.82754700	15.36344500	L
H	0	-11.85735900	-1.49533200	7.86206500	L
C	0	-11.66382100	-0.70250800	8.61633700	L
H	0	-11.72518900	-1.15117900	9.62854900	L

H	0	-12.43420200	0.09369300	8.53068100	L
O	0	-10.38949800	-0.15901900	8.43159500	L
H	0	-10.37557100	0.19741800	7.50601500	L
H	0	0.01086300	-7.70738400	15.56192800	L
C	0	-0.62683500	-7.52171000	14.67371700	L
H	0	-0.66013500	-8.44551900	14.05775500	L
H	0	-0.18267200	-6.69250800	14.08333600	L
O	0	-1.91407600	-7.17841000	15.09723800	L
H	0	-2.43148500	-6.98686700	14.27296000	L
H	0	-4.27471100	-3.30826300	12.39680200	L
C	0	-4.91692200	-2.83713900	11.62333400	L
H	0	-4.44661300	-2.99245900	10.63207200	L
H	0	-4.99670200	-1.74687200	11.81614000	L
O	0	-6.18859400	-3.41774200	11.62246400	L
H	0	-6.54206200	-3.30663200	12.54225300	L
H	0	-12.12149100	-3.00286400	5.51176200	L
C	0	-12.89946900	-3.75962300	5.74590700	L
H	0	-13.78431900	-3.60069100	5.09237600	L
H	0	-13.20798700	-3.63619700	6.80414500	L
O	0	-12.38908400	-5.04869600	5.56346900	L
H	0	-12.12998700	-5.10780500	4.60741200	L
H	0	-6.60045000	-6.15642100	7.77660100	L
C	0	-7.40296700	-6.27669400	8.53346300	L
H	0	-7.17580500	-7.16404100	9.15738400	L
H	0	-8.37547900	-6.43852700	8.02410500	L
O	0	-7.46965700	-5.14569100	9.35165600	L
H	0	-7.61059000	-4.37481100	8.74410600	L
H	0	-4.64382700	-9.70433200	11.35527500	L
C	0	-5.65537100	-9.86529200	10.92558900	L
H	0	-6.36221700	-9.11108200	11.33273700	L
H	0	-6.00780800	-10.87745600	11.21105500	L
O	0	-5.60605800	-9.77267000	9.53239600	L
H	0	-5.27609800	-8.86007600	9.33026200	L
H	0	2.04449800	-5.88462100	16.04860200	L
C	0	2.55262200	-5.69323600	15.08153000	L
H	0	1.93018100	-4.99511700	14.48206300	L
H	0	2.66218200	-6.65662300	14.53922200	L
O	0	3.80897700	-5.13144800	15.32719300	L
H	0	4.20220800	-4.94379200	14.43629800	L
H	0	0.18754700	-0.45179200	8.83441600	L
C	0	0.73016800	-0.90714200	9.68942100	L
H	0	1.76601900	-1.15254100	9.38237600	L
H	0	0.21679000	-1.84482600	9.98350000	L
O	0	0.74615900	-0.02821900	10.77622500	L

H	0	1.16431600	0.80838300	10.44466000	L
H	0	-11.09369200	-6.39129300	7.94860800	L
C	0	-11.28098400	-6.18980500	9.02314300	L
H	0	-10.36335000	-5.75188800	9.47079300	L
H	0	-11.52563900	-7.14852000	9.52839200	L
O	0	-12.34831200	-5.29482300	9.14402600	L
H	0	-12.45958900	-5.13262200	10.11668500	L
H	0	4.27903600	-9.18445700	7.85641500	L
C	0	3.19909600	-9.27245700	8.09777800	L
H	0	3.04664400	-8.96993600	9.15256600	L
H	0	2.87328600	-10.32743600	7.97885500	L
O	0	2.44889400	-8.43553700	7.26654600	L
H	0	2.62283600	-8.74371500	6.33987000	L
H	0	-0.18922600	-1.64561800	5.75623200	L
C	0	-1.11734100	-2.03641700	6.22520200	L
H	0	-0.96790500	-2.09571100	7.32152000	L
H	0	-1.33401800	-3.05531700	5.84165400	L
O	0	-2.18946700	-1.18400800	5.95166000	L
H	0	-2.28375000	-1.17216100	4.96409400	L
H	0	1.06590800	-9.68415100	10.55188100	L
C	0	0.98811500	-10.42726100	11.37352500	L
H	0	0.47583000	-9.95311300	12.23528000	L
H	0	2.00551500	-10.74307200	11.69043800	L
O	0	0.24726000	-11.53576500	10.95269400	L
H	0	0.74789500	-11.92582700	10.18982800	L
H	0	-5.07921400	-12.55870100	5.80519400	L
C	0	-5.61689700	-11.68825500	5.37473700	L
H	0	-6.69453500	-11.77771200	5.61834800	L
H	0	-5.50019400	-11.68364300	4.27101800	L
O	0	-5.12116700	-10.50032600	5.91804700	L
H	0	-4.16774700	-10.45312000	5.64989900	L
H	0	-3.18199600	-10.48027700	15.10970000	L
C	0	-3.28437400	-10.44281600	14.00589000	L
H	0	-3.38272600	-9.38157700	13.69436600	L
H	0	-4.19611700	-11.00603000	13.71024600	L
O	0	-2.15109700	-11.01476200	13.41962200	L
H	0	-2.29442600	-10.96188100	12.43940000	L
H	0	-8.29579700	-2.97762400	5.85085900	L
C	0	-9.08395000	-2.74204900	6.59324200	L
H	0	-9.82762300	-2.06475500	6.12326000	L
H	0	-8.61369500	-2.23794100	7.46272800	L
O	0	-9.70387300	-3.92583900	7.00068800	L
H	0	-10.39569000	-3.65786200	7.65894200	L
H	0	-4.02469300	-5.90955300	16.62438200	L

C	0	-4.81570800	-5.55450200	15.93281700	L
H	0	-5.70491300	-5.25857700	16.53012100	L
H	0	-5.08542100	-6.38258700	15.24336600	L
O	0	-4.33436400	-4.46041900	15.20768100	L
H	0	-5.07571100	-4.18018800	14.61161000	L
H	0	-4.40452700	11.88361000	0.80642200	L
C	0	-3.99922400	10.89483900	0.50368100	L
H	0	-2.90519600	10.99292400	0.36316500	L
H	0	-4.45794700	10.57358300	-0.45576000	L
O	0	-4.24824600	9.94326400	1.49529000	L
H	0	-5.22771600	9.95343200	1.64292600	L
H	0	-4.58810000	-2.09113200	4.26759600	L
C	0	-5.60403500	-1.69157300	4.06439200	L
H	0	-5.51849700	-0.82356800	3.37937900	L
H	0	-6.06957700	-1.35542400	5.01413900	L
O	0	-6.39736500	-2.67342000	3.46700600	L
H	0	-6.46558600	-3.40727500	4.13016900	L
H	0	-9.92224600	9.01349300	1.96879500	L
C	0	-10.52645500	9.52020400	1.18822100	L
H	0	-11.60169300	9.31645500	1.37646600	L
H	0	-10.24177700	9.11423700	0.19511800	L
O	0	-10.27948200	10.89536700	1.23447400	L
H	0	-10.86159700	11.30268200	0.54226300	L
H	0	-11.83506300	1.97328800	1.95604400	L
C	0	-10.90633600	1.36551600	1.95107500	L
H	0	-11.07779500	0.41739500	1.39852900	L
H	0	-10.63538600	1.12081100	2.99608300	L
O	0	-9.86357200	2.08723600	1.36475000	L
H	0	-10.13257200	2.23560300	0.42242300	L
H	0	-3.09263000	14.40740100	5.44126200	L
C	0	-3.05721300	15.23574600	6.18177400	L
H	0	-4.02574200	15.77993000	6.18271400	L
H	0	-2.25220000	15.94046400	5.89009500	L
O	0	-2.78652600	14.73549400	7.45913700	L
H	0	-3.48795800	14.05882400	7.64253000	L
H	0	-5.15708700	3.46538700	4.83106200	L
C	0	-5.07156800	4.54643700	5.06246800	L
H	0	-5.84366800	5.09714400	4.48501000	L
H	0	-4.06058600	4.89707500	4.76459300	L
O	0	-5.26606500	4.74040700	6.43316900	L
H	0	-5.14669000	5.71181800	6.59140700	L
H	0	-3.31527500	7.47261100	6.84038100	L
C	0	-2.24855200	7.16825900	6.90008100	L
H	0	-1.83922400	7.41763100	7.90253900	L

H	0	-1.67740900	7.72882900	6.13408700	L
O	0	-2.12445300	5.79789000	6.65465500	L
H	0	-2.60642200	5.34298100	7.39229400	L
H	0	-11.14556800	7.38965800	8.34140600	L
C	0	-11.00651300	8.42224200	8.72764200	L
H	0	-11.42272100	9.15192200	8.00101300	L
H	0	-9.92117200	8.61652300	8.84871100	L
O	0	-11.63796900	8.56312800	9.96685900	L
H	0	-12.59411900	8.34754800	9.81201200	L
H	0	-4.37492600	8.19703500	9.10122500	L
C	0	-4.32850300	7.35142700	9.81489400	L
H	0	-4.33298900	6.40186100	9.23895300	L
H	0	-5.21901100	7.39524100	10.47812400	L
O	0	-3.15661300	7.45386300	10.56997300	L
H	0	-3.16312300	6.68012800	11.19062900	L
H	0	-7.67089400	3.75912800	0.95733400	L
C	0	-6.70482800	4.09583700	1.38934000	L
H	0	-6.88812400	4.98517900	2.02477300	L
H	0	-6.00857500	4.38209200	0.57300700	L
O	0	-6.14576400	3.08208700	2.17291700	L
H	0	-5.95565700	2.32932800	1.55564700	L
H	0	-10.73570300	11.18909000	6.26440200	L
C	0	-9.87397800	10.72381200	5.74189300	L
H	0	-9.69288000	9.72397600	6.18160800	L
H	0	-8.96578100	11.34552600	5.88497400	L
O	0	-10.15183900	10.58274700	4.37985100	L
H	0	-10.33198200	11.49755900	4.04168900	L
H	0	-11.31805900	4.09697100	10.69474500	L
C	0	-10.80747600	5.03172100	11.00412400	L
H	0	-10.30607300	4.85848600	11.98100100	L
H	0	-11.56708600	5.83628200	11.10764100	L
O	0	-9.86483400	5.38230500	10.03360000	L
H	0	-9.42513000	6.20781900	10.36493500	L
H	0	-5.81095300	13.78465000	6.68557500	L
C	0	-6.43306100	13.90395600	5.77429500	L
H	0	-7.24042900	13.14260300	5.77283500	L
H	0	-5.79398200	13.74699100	4.88288500	L
O	0	-6.97954700	15.18985900	5.72567800	L
H	0	-7.54956300	15.27543100	6.53326100	L
H	0	-9.02437600	2.68157900	13.11912600	L
C	0	-8.78304000	1.79795700	12.49368600	L
H	0	-9.20439400	0.89073100	12.97743900	L
H	0	-7.67808400	1.70608000	12.42066100	L
O	0	-9.34126000	1.96956200	11.22328900	L

H	0	-9.07564400	1.17215800	10.69711400	L
H	0	-10.96857200	2.78979200	8.41892000	L
C	0	-11.44874400	3.09184300	7.46356400	L
H	0	-12.43010700	2.58069700	7.35990000	L
H	0	-10.79594600	2.78407800	6.62169100	L
O	0	-11.61889200	4.47871100	7.42759500	L
H	0	-12.23736400	4.69917500	8.17140100	L
H	0	-4.06652600	11.32715400	3.87524600	L
C	0	-4.19407500	11.06841500	4.94716600	L
H	0	-5.23874300	10.73832700	5.12564100	L
H	0	-3.99248100	11.97120100	5.55792500	L
O	0	-3.29992400	10.05868900	5.31460100	L
H	0	-3.47122600	9.30236100	4.69605700	L
H	0	-6.64002000	8.85257600	3.32346800	L
C	0	-7.03539600	8.21705100	4.14258700	L
H	0	-7.36625400	7.23767800	3.73433400	L
H	0	-7.90646600	8.72868500	4.59695700	L
O	0	-6.05450400	8.02653000	5.11992800	L
H	0	-5.29117900	7.59105600	4.66022300	L
H	0	-6.57554800	13.56907600	10.56760700	L
C	0	-5.66929700	13.67283300	9.93260900	L
H	0	-5.73492500	12.97571000	9.07099200	L
H	0	-4.77651100	13.40963000	10.53587700	L
O	0	-5.54494800	14.98998800	9.47982400	L
H	0	-6.35812600	15.17230200	8.94142800	L
H	0	-8.44793800	7.51323300	-0.76307600	L
C	0	-7.75240300	7.36419400	0.08942600	L
H	0	-6.87379900	6.76834700	-0.23930400	L
H	0	-8.28459700	6.80842900	0.88636200	L
O	0	-7.33884300	8.60019200	0.59307600	L
H	0	-6.83253800	9.03876500	-0.13811900	L
H	0	-0.78185900	5.89401900	10.39110400	L
C	0	-0.21621700	5.47111500	11.24614200	L
H	0	0.14154500	4.45697400	10.96739700	L
H	0	-0.89288700	5.40202400	12.12511000	L
O	0	0.86903000	6.30377000	11.53600200	L
H	0	1.34965700	5.87351100	12.28996000	L
H	0	-9.78754300	4.83212800	4.88670900	L
C	0	-9.31858200	4.31938400	4.02091800	L
H	0	-9.61535100	3.25290900	4.04139400	L
H	0	-8.21318900	4.38412500	4.09990000	L
O	0	-9.75407800	4.89374500	2.82351800	L
H	0	-9.45131000	5.83788100	2.84878600	L
H	0	-4.55989700	8.45829400	-4.25986200	L

C	0	-4.99133800	9.04242400	-3.41977500	L
H	0	-5.79597000	9.69434700	-3.81581000	L
H	0	-4.20832600	9.68503500	-2.96406800	L
O	0	-5.52743300	8.17778700	-2.46129800	L
H	0	-4.76627500	7.63945500	-2.12237900	L
H	0	-13.55738900	5.81307800	1.60666200	L
C	0	-14.44949100	6.46732500	1.71683600	L
H	0	-14.84703600	6.35793300	2.74605200	L
H	0	-15.23539400	6.15574800	0.99595400	L
O	0	-14.10147400	7.80414800	1.50074800	L
H	0	-13.70286500	7.83981200	0.59315300	L
H	0	-7.55537500	4.25529900	11.11695800	L
C	0	-6.45362700	4.29343400	11.00553400	L
H	0	-6.20879700	4.76914500	10.03200600	L
H	0	-6.05620600	3.25570000	11.02544800	L
O	0	-5.91502500	5.04018000	12.05717000	L
H	0	-4.93199800	5.02488300	11.92650300	L
H	0	-7.58386800	10.99460200	2.14089700	L
C	0	-7.33138400	11.84279700	2.80832200	L
H	0	-6.29617700	12.17849100	2.58631300	L
H	0	-7.39489700	11.49578700	3.86106100	L
O	0	-8.23580900	12.88571300	2.58589300	L
H	0	-7.96105400	13.62133200	3.19194000	L
H	0	-13.22002300	10.32497600	5.67202100	L
C	0	-13.60397800	11.01340600	4.88916800	L
H	0	-12.91514800	11.87820800	4.77931700	L
H	0	-14.60016100	11.38744200	5.20192900	L
O	0	-13.72854600	10.33877000	3.67094100	L
H	0	-12.82294700	9.99656300	3.45525000	L
H	0	-4.24745600	13.38878800	-4.17898000	L
C	0	-4.21325400	12.65522300	-5.01237300	L
H	0	-3.93469400	11.65372300	-4.62028800	L
H	0	-3.44229200	12.97792100	-5.74183100	L
O	0	-5.45565400	12.59855000	-5.65164100	L
H	0	-6.09481500	12.24776800	-4.97861000	L
H	0	-14.92497500	4.31752900	4.31149000	L
C	0	-13.96851200	4.03364900	4.79540700	L
H	0	-14.11085000	3.06808600	5.32694400	L
H	0	-13.68671900	4.82547300	5.52187100	L
O	0	-12.97882000	3.90459800	3.81695000	L
H	0	-12.15159300	3.64894300	4.29987900	L
H	0	-12.63024200	11.49680800	7.88866600	L
C	0	-13.32346300	11.50565700	8.75617100	L
H	0	-13.84285800	12.48686500	8.80988700	L

H	0	-12.73347400	11.36143500	9.68434000	L
O	0	-14.25579500	10.46988500	8.64049000	L
H	0	-14.76406500	10.65023400	7.80776300	L
H	0	-6.47205800	11.20817900	7.38597700	L
C	0	-6.56799500	10.17847300	7.78444100	L
H	0	-5.65969100	9.60220100	7.50808600	L
H	0	-7.46354500	9.70429200	7.33160500	L
O	0	-6.69909300	10.23385500	9.17534300	L
H	0	-6.85017700	9.29988800	9.47376700	L
H	0	-9.67462200	13.85465300	5.47902400	L
C	0	-10.69180800	14.29404400	5.45057100	L
H	0	-11.20227200	13.94949300	4.52512100	L
H	0	-10.60246300	15.40161400	5.44112300	L
O	0	-11.40314900	13.87851200	6.58051600	L
H	0	-12.31493400	14.25815500	6.48561200	L
H	0	-4.68909000	-0.22691100	6.72166100	L
C	0	-4.66323200	0.83654900	6.41151700	L
H	0	-5.70495700	1.20756500	6.32366200	L
H	0	-4.15273800	0.91332100	5.42683200	L
O	0	-3.97191400	1.57210100	7.37608600	L
H	0	-3.94269300	2.50410600	7.04049600	L
H	0	-10.20578900	7.52054500	4.74011000	L
C	0	-11.28248700	7.38127000	4.97625800	L
H	0	-11.73759200	8.35621000	5.24739600	L
H	0	-11.37425000	6.69517600	5.84120400	L
O	0	-11.94958300	6.82795400	3.88065100	L
H	0	-11.80475700	7.45698200	3.12806800	L
H	0	-12.17143100	1.23381200	5.06083400	L
C	0	-12.56275900	0.19939500	5.12492600	L
H	0	-13.24893800	0.12887000	5.99590200	L
H	0	-11.70600600	-0.49407200	5.26236600	L
O	0	-13.24899900	-0.10456900	3.94544400	L
H	0	-13.55794600	-1.04239000	4.04259700	L
H	0	-3.79330800	11.54225300	8.07094900	L
C	0	-3.01107700	11.02414500	8.66079000	L
H	0	-2.10150000	11.66184900	8.68649900	L
H	0	-2.77811000	10.05480800	8.17016300	L
O	0	-3.48412200	10.80712000	9.95828100	L
H	0	-2.75083900	10.34648400	10.44260400	L
H	0	-15.71646800	6.82364000	5.61984800	L
C	0	-15.00275200	7.64499500	5.83535900	L
H	0	-15.57630900	8.57216500	6.05230900	L
H	0	-14.36175400	7.80240800	4.94205300	L
O	0	-14.21768500	7.29337200	6.93782500	L

H	0	-13.60876600	8.06045100	7.09466600	L
H	0	-3.56825800	7.38792800	2.40747500	L
C	0	-4.25464700	6.68823500	1.88546600	L
H	0	-5.29139200	7.06532000	1.98522000	L
H	0	-4.19398200	5.68421400	2.35508400	L
O	0	-3.92639500	6.60893300	0.52929000	L
H	0	-3.00721000	6.23835500	0.49326800	L
H	0	-6.52430100	13.41812900	0.02856800	L
C	0	-6.88068300	12.70109900	-0.73928700	L
H	0	-7.87817700	13.02959700	-1.10213900	L
H	0	-6.96338200	11.69409400	-0.27633300	L
O	0	-5.96923900	12.67344400	-1.79905700	L
H	0	-6.29601700	11.97578300	-2.42379900	L
H	0	-11.54201600	4.38963000	0.21169600	L
C	0	-10.91699300	5.02631500	-0.45157900	L
H	0	-11.14626000	4.77264900	-1.50609200	L
H	0	-9.84020600	4.82728000	-0.26166300	L
O	0	-11.20199800	6.37708100	-0.23250700	L
H	0	-11.01838100	6.54232500	0.72821100	L
H	0	-2.82284300	3.93320300	10.78657100	L
C	0	-3.07185000	3.37075300	9.86184100	L
H	0	-3.75970900	3.98330600	9.24536100	L
H	0	-3.58597900	2.42151000	10.12388600	L
O	0	-1.91093700	3.10621200	9.12855200	L
H	0	-1.34828100	2.52425900	9.70202600	L
H	0	-9.22766600	5.91354700	7.31729400	L
C	0	-8.33692500	6.53298800	7.09225800	L
H	0	-8.67158000	7.56150400	6.84560000	L
H	0	-7.80795600	6.09346200	6.22042100	L
O	0	-7.49681500	6.55272500	8.20908800	L
H	0	-6.72340300	7.12038800	7.95801100	L
H	0	-6.75486300	2.72823200	8.31211000	L
C	0	-7.83789600	2.74121900	8.06131800	L
H	0	-8.38426200	3.28137200	8.85962100	L
H	0	-8.22679100	1.70344300	8.00408600	L
O	0	-8.04654700	3.39458300	6.84520000	L
H	0	-7.48499700	2.92169800	6.17968800	L
H	0	-8.84603100	-0.51257000	4.40079700	L
C	0	-8.49083400	0.38591000	4.94848300	L
H	0	-8.01553000	0.06201800	5.89597400	L
H	0	-9.35156900	1.04376400	5.19264700	L
O	0	-7.55329200	1.07994700	4.17800900	L
H	0	-8.01799500	1.32504900	3.33716500	L
H	0	-9.55252700	11.20206100	8.73511700	L

C	0	-9.45242500	12.23717000	9.11929200	L
H	0	-8.94398500	12.20383900	10.10686500	L
H	0	-10.46688300	12.67639500	9.23251200	L
O	0	-8.69823600	12.99104600	8.21557300	L
H	0	-8.66429000	13.91018000	8.58736200	L
H	0	3.87971000	-12.08375100	-8.63827200	L
C	0	3.88151400	-10.97832200	-8.52601200	L
H	0	2.83844500	-10.61076500	-8.41858800	L
H	0	4.32807000	-10.52659100	-9.43497700	L
O	0	4.64020300	-10.60952700	-7.41176300	L
H	0	4.17753900	-10.99829800	-6.62599900	L
H	0	6.91216200	-4.74331000	-4.84946700	L
C	0	5.89122200	-4.31163100	-4.83000900	L
H	0	5.96916600	-3.21020900	-4.92018200	L
H	0	5.40088200	-4.55950900	-3.86442000	L
O	0	5.13691000	-4.80605900	-5.89746000	L
H	0	5.02499700	-5.77575300	-5.72507600	L
H	0	16.23465400	-1.98496800	0.08287200	L
C	0	15.96126300	-3.05295500	0.20489400	L
H	0	15.15101100	-3.29958200	-0.51326900	L
H	0	15.60054900	-3.21309400	1.24298900	L
O	0	17.08469300	-3.84726100	-0.04355300	L
H	0	16.78596200	-4.78667000	0.07055300	L
H	0	10.44548900	-3.45477600	-5.92703000	L
C	0	9.63461200	-2.90575600	-5.40874700	L
H	0	10.07654800	-2.02606000	-4.89355300	L
H	0	9.16488100	-3.58140300	-4.66312900	L
O	0	8.68558500	-2.49375900	-6.34912800	L
H	0	8.01077600	-1.96797800	-5.84707000	L
H	0	13.52541700	-1.45009300	1.18429900	L
C	0	12.93924200	-0.79199300	0.51233000	L
H	0	12.44018800	-0.00991100	1.12333200	L
H	0	12.17286500	-1.40406300	-0.00856900	L
O	0	13.80278700	-0.20377400	-0.41624700	L
H	0	13.23533300	0.36686800	-0.99552100	L
H	0	10.05733400	-8.04614600	1.78083100	L
C	0	9.83978800	-8.19632500	0.70546200	L
H	0	10.77150000	-8.02271000	0.12727000	L
H	0	9.06612900	-7.46376300	0.39298900	L
O	0	9.38284000	-9.50314100	0.50992900	L
H	0	9.19132700	-9.58473700	-0.45966200	L
H	0	9.08522400	-4.92022200	-11.04697800	L
C	0	9.31808700	-5.77120500	-11.72292000	L
H	0	8.75434800	-5.65964000	-12.67389000	L

H	0	9.00568300	-6.71431200	-11.23030200	L
O	0	10.69110100	-5.82372100	-11.98187400	L
H	0	10.92470500	-4.95273000	-12.39578500	L
H	0	3.14271500	-14.55469000	-7.34678300	L
C	0	3.86742000	-14.54614400	-6.50704300	L
H	0	3.33194600	-14.80681100	-5.56911900	L
H	0	4.30012100	-13.52725900	-6.41885600	L
O	0	4.87809800	-15.47826000	-6.76091700	L
H	0	5.49708200	-15.42764700	-5.98695700	L
H	0	9.37770600	-8.79626300	5.33685900	L
C	0	9.64440300	-9.85215400	5.11971600	L
H	0	8.95912500	-10.53274600	5.66930600	L
H	0	10.68307600	-10.03443400	5.46374500	L
O	0	9.56605000	-10.09709500	3.74554800	L
H	0	8.63570000	-9.87593000	3.48128500	L
H	0	11.14463300	-9.25120500	-4.47724500	L
C	0	11.69882300	-9.34733200	-3.52017400	L
H	0	12.67496200	-8.82327700	-3.59679500	L
H	0	11.88705800	-10.42114900	-3.31982600	L
O	0	10.94151800	-8.81340900	-2.47443000	L
H	0	10.86472800	-7.84201700	-2.65834500	L
H	0	6.44071300	-4.57417800	-9.55885900	L
C	0	6.67588300	-5.53544500	-9.05646500	L
H	0	6.88861300	-6.29719200	-9.83185000	L
H	0	5.80404500	-5.87198000	-8.45578400	L
O	0	7.80018700	-5.38736800	-8.24047100	L
H	0	7.54565500	-4.73636800	-7.53737300	L
H	0	2.75874900	-11.87332900	0.26200300	L
C	0	3.86470100	-11.88667100	0.18850700	L
H	0	4.18133500	-12.85772400	-0.24879400	L
H	0	4.29094100	-11.77712000	1.20876900	L
O	0	4.28247600	-10.82913400	-0.62507700	L
H	0	5.27023800	-10.90127200	-0.68372100	L
H	0	6.92355100	-5.82453000	-1.70421600	L
C	0	6.38277100	-6.77507900	-1.89758600	L
H	0	6.09670200	-6.83326800	-2.96884200	L
H	0	5.46231800	-6.79174800	-1.28024600	L
O	0	7.18304100	-7.86859800	-1.55576900	L
H	0	8.00441700	-7.78931400	-2.10609300	L
H	0	10.61956300	-9.50245000	-7.26929700	L
C	0	11.66495600	-9.14789800	-7.38912600	L
H	0	12.33987000	-9.84186600	-6.84905800	L
H	0	11.76955200	-8.13508300	-6.94694300	L
O	0	12.01573100	-9.12361500	-8.74223000	L

H	0	11.39685000	-8.48036700	-9.17493300	L
H	0	14.04831900	-2.79671800	-7.44214800	L
C	0	13.81585600	-1.97851300	-6.72810400	L
H	0	14.42079000	-2.12856800	-5.81177800	L
H	0	12.73844400	-2.00926900	-6.45629600	L
O	0	14.13861400	-0.74034300	-7.29117800	L
H	0	13.58472800	-0.66040400	-8.10994400	L
H	0	9.40029600	-11.31463700	-2.78961800	L
C	0	8.34285000	-11.19153100	-3.10685900	L
H	0	7.71345600	-11.03649100	-2.20652500	L
H	0	8.00091800	-12.11048800	-3.62934100	L
O	0	8.21907100	-10.08201300	-3.94762000	L
H	0	8.74693000	-10.29189800	-4.76060500	L
H	0	11.44454200	0.71814700	-7.65778500	L
C	0	10.48265600	0.16230400	-7.67147900	L
H	0	10.69525200	-0.92150700	-7.77446400	L
H	0	9.87430500	0.49129500	-8.53977200	L
O	0	9.78127200	0.38295600	-6.48280800	L
H	0	9.63173400	1.36187500	-6.43161100	L
H	0	14.50864100	-12.97169600	-8.52950500	L
C	0	14.14260300	-11.99105400	-8.15621900	L
H	0	14.63339700	-11.77159900	-7.18613500	L
H	0	13.04351000	-12.03761600	-7.99895500	L
O	0	14.45915900	-10.97997800	-9.06874000	L
H	0	13.97425700	-11.20482200	-9.90484600	L
H	0	15.36780300	-5.82847800	-4.03910400	L
C	0	16.30714400	-5.27194800	-3.83441200	L
H	0	17.18038100	-5.88955400	-4.13592400	L
H	0	16.37042400	-5.06790300	-2.74597600	L
O	0	16.31264000	-4.05835000	-4.52907200	L
H	0	16.21846100	-4.29173200	-5.48878000	L
H	0	2.34170300	-12.13064800	-5.29190300	L
C	0	1.38535700	-12.00770200	-5.84275000	L
H	0	0.60631300	-12.64705500	-5.37896400	L
H	0	1.53073100	-12.33136300	-6.89333600	L
O	0	0.98536400	-10.66834300	-5.82497600	L
H	0	0.87491700	-10.42789300	-4.86883600	L
H	0	14.19458000	1.08165000	-3.62540400	L
C	0	13.28616300	1.61503300	-3.97860100	L
H	0	13.57918400	2.62449000	-4.33275600	L
H	0	12.56696100	1.72841200	-3.13997900	L
O	0	12.69295500	0.91397500	-5.03288200	L
H	0	12.43143300	0.03117300	-4.66358100	L
H	0	9.42815400	-5.46800900	-2.63405500	L

C	0	9.90386900	-4.99024000	-1.75380000	L
H	0	10.91053900	-5.43718100	-1.60330300	L
H	0	9.27165700	-5.17987500	-0.86048800	L
O	0	10.01408300	-3.61579000	-1.98395100	L
H	0	10.46633200	-3.23939600	-1.18550700	L
H	0	8.35594800	-2.67083900	2.40007200	L
C	0	9.28108600	-2.77175000	1.79415800	L
H	0	9.09820100	-3.46030700	0.94361600	L
H	0	10.07562000	-3.19671800	2.43678700	L
O	0	9.69663600	-1.52283300	1.32525800	L
H	0	8.94909400	-1.17707100	0.77452400	L
H	0	5.59930600	-12.71566500	-2.55757000	L
C	0	5.20921400	-13.48470300	-3.25423300	L
H	0	6.02199300	-13.78411000	-3.94961000	L
H	0	4.36551900	-13.04855700	-3.82987500	L
O	0	4.77312500	-14.59135700	-2.51964700	L
H	0	4.41958400	-15.23832300	-3.18375900	L
H	0	12.43364300	-2.65860300	-3.62598100	L
C	0	13.30936600	-2.76896500	-2.95441600	L
H	0	13.84125600	-3.70775600	-3.21229400	L
H	0	12.95454100	-2.82164800	-1.90391500	L
O	0	14.15753400	-1.67080100	-3.12271400	L
H	0	14.95304400	-1.84771800	-2.55732300	L
H	0	13.84461500	-10.08967300	2.21059800	L
C	0	13.00218700	-9.77592900	1.56085500	L
H	0	13.04347300	-8.67321000	1.43318700	L
H	0	12.04640000	-10.06146000	2.05130600	L
O	0	13.11748400	-10.41054700	0.31996400	L
H	0	12.33887600	-10.10862200	-0.21586400	L
H	0	5.36451800	-8.47523300	0.68448700	L
C	0	5.93260800	-8.46507700	1.63903900	L
H	0	5.33394700	-7.93101900	2.40368000	L
H	0	6.89244100	-7.92496200	1.50049500	L
O	0	6.17117400	-9.77242300	2.07247100	L
H	0	6.72268300	-10.19816100	1.36617500	L
H	0	12.02515700	-1.59079600	-9.97871700	L
C	0	12.60535100	-1.86627800	-10.88553800	L
H	0	12.70984500	-0.97025700	-11.53022100	L
H	0	12.06263300	-2.65132400	-11.45441400	L
O	0	13.87685000	-2.32282400	-10.52521700	L
H	0	13.73011600	-3.13797000	-9.97911900	L
H	0	2.64472900	-8.47930400	-5.99878900	L
C	0	2.55923600	-7.67493800	-6.75913100	L
H	0	2.55629200	-6.68272100	-6.25953200	L

H	0	1.60195800	-7.80164300	-7.30401200	L
O	0	3.61936000	-7.75169800	-7.66725000	L
H	0	4.44878300	-7.72075300	-7.12434100	L
H	0	14.89669400	-7.20111100	-9.19241700	L
C	0	14.24621000	-6.34980200	-8.89694000	L
H	0	13.47728000	-6.70050300	-8.17696600	L
H	0	13.73584800	-5.95700400	-9.80023300	L
O	0	15.01442000	-5.33201500	-8.32365700	L
H	0	15.42406400	-5.72459300	-7.50972000	L
H	0	7.81615100	-9.62332800	-7.44727000	L
C	0	7.48605500	-9.34044900	-8.47003200	L
H	0	6.62186900	-8.64562600	-8.40649300	L
H	0	7.16736600	-10.25569800	-9.00915000	L
O	0	8.53675200	-8.73924300	-9.16893100	L
H	0	8.80133400	-7.94584500	-8.63590100	L
H	0	-0.94930400	-8.06700300	-8.21401000	L
C	0	-1.81125800	-7.80509000	-8.86461500	L
H	0	-2.74158000	-8.17937300	-8.39113800	L
H	0	-1.69466800	-8.29093200	-9.85714700	L
O	0	-1.89796700	-6.41859800	-9.01583000	L
H	0	-1.03471300	-6.13196600	-9.41217800	L
H	0	5.48559100	-10.62002100	-4.38982700	L
C	0	4.94606900	-9.72429000	-4.01685300	L
H	0	5.09878200	-8.89225200	-4.73316000	L
H	0	5.35251900	-9.42771600	-3.02678600	L
O	0	3.57920000	-9.99376200	-3.91495100	L
H	0	3.49607700	-10.73952600	-3.26746600	L
H	0	14.81751200	-7.90544200	-0.52503300	L
C	0	14.15416200	-7.33028500	-1.20279200	L
H	0	13.10427000	-7.65293100	-1.03424500	L
H	0	14.44809400	-7.53909200	-2.25351900	L
O	0	14.29143500	-5.96693400	-0.92892400	L
H	0	13.61864100	-5.50590800	-1.49302600	L
H	0	3.17888900	-7.64625800	-2.30130300	L
C	0	2.95639500	-6.82136500	-3.01209800	L
H	0	3.60320900	-6.91459900	-3.90948700	L
H	0	3.17766400	-5.85438400	-2.51712800	L
O	0	1.60920300	-6.85353600	-3.38358100	L
H	0	1.46032100	-7.74654500	-3.78865200	L
H	0	7.60524500	-2.16661900	-1.03603200	L
C	0	6.75040000	-2.22375600	-1.74165900	L
H	0	5.83159700	-1.88960200	-1.21933700	L
H	0	6.61271200	-3.27485400	-2.07214800	L
O	0	6.97844000	-1.39548000	-2.84331800	L

H	0	7.79285600	-1.74878100	-3.28509300	L
H	0	6.57572100	-12.18512700	-6.01055500	L
C	0	7.34424500	-12.69452000	-6.62537000	L
H	0	7.63090700	-13.64436300	-6.12457000	L
H	0	8.23061000	-12.03108400	-6.70715100	L
O	0	6.81862400	-12.95577200	-7.89430000	L
H	0	7.53581800	-13.42255700	-8.39627200	L
H	0	8.10954100	-2.58892400	-9.12788100	L
C	0	8.33571700	-2.03834500	-10.06192400	L
H	0	7.83758000	-2.55362500	-10.91033200	L
H	0	9.43650700	-2.03611800	-10.21512400	L
O	0	7.85707900	-0.73028200	-9.94865100	L
H	0	8.15670600	-0.25791500	-10.76786000	L
H	0	5.69992100	-5.59330000	1.11873400	L
C	0	5.80658100	-4.49148600	1.04399300	L
H	0	6.73975200	-4.24564100	0.49450400	L
H	0	5.87169300	-4.06996200	2.06688700	L
O	0	4.69970600	-3.94133900	0.39067800	L
H	0	4.69047200	-4.34231900	-0.51687700	L
H	0	9.24759500	-7.77294900	-6.08028600	L
C	0	8.68763400	-7.14787700	-5.35426100	L
H	0	8.84483400	-6.07288800	-5.58475600	L
H	0	9.07407500	-7.35239200	-4.33696700	L
O	0	7.32703700	-7.46248100	-5.39768200	L
H	0	7.02534300	-7.24582800	-6.31681400	L
H	0	2.18017200	-3.95244500	-9.32326200	L
C	0	2.56234100	-4.94755000	-9.01492300	L
H	0	1.69814600	-5.58888200	-8.75163100	L
H	0	3.21717100	-4.83669600	-8.12391700	L
O	0	3.26934600	-5.54412400	-10.06241800	L
H	0	4.00563600	-4.91918600	-10.28502400	L
H	0	15.87703100	-7.80662200	-5.85998800	L
C	0	15.00448600	-8.48961100	-5.90760400	L
H	0	14.91101200	-8.87948200	-6.94423600	L
H	0	14.09023200	-7.92021100	-5.63817500	L
O	0	15.19675900	-9.54424400	-5.01002900	L
H	0	14.39613400	-10.12422300	-5.09453200	L
H	0	11.98811700	-4.13607300	-8.06651100	L
C	0	11.11667600	-4.45469400	-8.67586400	L
H	0	11.37910400	-4.35819700	-9.74699600	L
H	0	10.24794800	-3.79860400	-8.45962100	L
O	0	10.79579000	-5.78710100	-8.40295300	L
H	0	10.57724000	-5.82193800	-7.43634600	L
H	0	11.14733600	-11.68915200	-9.81494100	L

C	0	10.16477800	-12.06226100	-9.45345400	L
H	0	9.82677400	-11.45373300	-8.58849000	L
H	0	10.28138900	-13.11475400	-9.12341600	L
O	0	9.21816000	-12.00536800	-10.48102500	L
H	0	9.16622500	-11.05239400	-10.75266800	L
H	0	1.18729300	-7.35136000	1.17392800	H
C	0	1.50467000	-6.49223200	0.56589700	H
H	0	1.18349600	-5.57379200	1.07727900	H
H	0	0.95430100	-6.53930300	-0.37668900	H
O	0	2.88263600	-6.51249600	0.26480100	H
H	0	3.35672700	-6.51361500	1.09928800	H
H	0	4.53310200	-7.40596000	-11.97903700	L
C	0	5.12381600	-8.21485800	-11.50262600	L
H	0	4.65449800	-9.19179000	-11.74916100	L
H	0	5.11485600	-8.06205200	-10.40204500	L
O	0	6.43513900	-8.17311100	-11.98619700	L
H	0	6.91490300	-8.91814100	-11.53994500	L
H	0	2.85452100	-2.61761900	-5.30928800	H
C	0	2.61895200	-3.13335400	-4.37714300	H
H	0	2.72862100	-4.21216500	-4.55313800	H
H	0	1.57055100	-2.91847600	-4.12645200	H
O	0	3.52359500	-2.64659900	-3.40269300	H
H	0	3.40846100	-3.17886500	-2.61018800	H
H	0	-0.21881000	-5.59371400	-6.94742600	L
C	0	-0.07019500	-4.55010700	-6.59811000	L
H	0	1.01696500	-4.36071800	-6.50024900	L
H	0	-0.54622500	-4.41801500	-5.60410600	L
O	0	-0.60997100	-3.64908400	-7.52123900	L
H	0	-1.58281700	-3.83896900	-7.55495200	L
H	0	9.65533100	-0.72039800	-1.61831800	L
C	0	10.37241500	-0.09970800	-2.19498200	L
H	0	11.26461800	-0.71258000	-2.42888200	L
H	0	10.68893100	0.77313200	-1.58535100	L
O	0	9.78797400	0.33197500	-3.38863600	L
H	0	8.98934600	0.85772300	-3.12650000	L
H	0	10.13021400	-13.17202100	-5.02865600	L
C	0	10.95411300	-12.65699800	-5.56354000	L
H	0	10.68316600	-11.58739000	-5.69025100	L
H	0	11.08572900	-13.13030000	-6.55996500	L
O	0	12.12980300	-12.77448200	-4.81616900	L
H	0	12.81785800	-12.25355500	-5.30542600	L
H	0	1.05835800	-9.18262700	-9.50059700	L
C	0	1.44739200	-8.52293100	-10.30395600	L
H	0	2.54160100	-8.41348900	-10.17362600	L

H	0	0.97664500	-7.52079000	-10.22479300	L
O	0	1.18660400	-9.08231600	-11.55825200	L
H	0	0.19929100	-9.15797300	-11.62316400	L
H	0	6.17587900	-5.32048800	-12.27100600	L
C	0	5.84145200	-4.34864400	-12.69182700	L
H	0	6.67799000	-3.89946500	-13.26522200	L
H	0	5.56534900	-3.66081700	-11.86584500	L
O	0	4.74962800	-4.54071100	-13.54403800	L
H	0	4.03185200	-4.93629600	-12.98463600	L
H	0	11.50958000	-6.66720800	-4.94236800	L
C	0	12.16543800	-5.82378000	-4.65411600	L
H	0	12.85800100	-6.16825600	-3.85784200	L
H	0	11.53431200	-4.99606900	-4.26960300	L
O	0	12.88593900	-5.40327800	-5.77511600	L
H	0	13.45974500	-4.65543100	-5.46883500	L
H	0	-1.57679100	3.04769000	-13.65088600	L
C	0	-2.13058700	2.09484200	-13.79047400	L
H	0	-2.25694800	1.58853500	-12.80959800	L
H	0	-1.54141500	1.43554900	-14.45967500	L
O	0	-3.37832200	2.34162000	-14.37134900	L
H	0	-3.86501600	2.92850400	-13.73635500	L
H	0	5.81499400	3.97692000	-3.15197900	L
C	0	5.75831800	3.14667000	-2.42068500	L
H	0	4.70564100	2.79565700	-2.36135100	L
H	0	6.08890400	3.52123700	-1.42965900	L
O	0	6.59329400	2.10920400	-2.84149100	L
H	0	6.41207000	1.34788700	-2.23249000	L
H	0	-0.14450900	4.10347700	-7.24551400	L
C	0	0.41063600	3.37897700	-7.87737400	L
H	0	1.16425500	3.93227000	-8.47100100	L
H	0	-0.28945200	2.86853700	-8.57233900	L
O	0	1.06112900	2.44057300	-7.07268200	L
H	0	0.34501400	1.94661100	-6.59693100	L
H	0	8.90286200	11.23699000	-6.84201700	L
C	0	8.87968500	10.27545100	-6.29003800	L
H	0	8.13601900	9.60585000	-6.77070800	L
H	0	8.57947900	10.47114300	-5.23917200	L
O	0	10.14980200	9.69258500	-6.32957000	L
H	0	10.07570100	8.84219600	-5.82410600	L
H	0	4.64990800	13.69797400	-5.94063100	L
C	0	3.79143500	14.08684900	-6.52448900	L
H	0	3.19753300	14.76770700	-5.87746100	L
H	0	3.16137700	13.23023400	-6.84702400	L
O	0	4.27198300	14.77914300	-7.63996700	L

H	0	3.46993900	15.09057900	-8.13428000	L
H	0	8.62164800	0.85925900	-14.53393500	L
C	0	8.44453600	1.90998400	-14.22704900	L
H	0	7.39679400	2.18029700	-14.47917100	L
H	0	9.14764300	2.56873100	-14.78095400	L
O	0	8.65596700	2.02932600	-12.85027400	L
H	0	8.47042400	2.97793600	-12.62675900	L
H	0	1.75131200	7.65357000	-5.02535400	L
C	0	2.35475500	6.97936100	-5.66487100	L
H	0	3.37134500	6.88711000	-5.22780800	L
H	0	2.42394100	7.41756600	-6.68263500	L
O	0	1.73810700	5.72661200	-5.71893900	L
H	0	2.29134300	5.17736700	-6.33071500	L
H	0	-0.43182600	-0.62640500	-13.09873600	L
C	0	-1.12904800	-1.46868600	-12.91448500	L
H	0	-1.47546600	-1.41632700	-11.86105500	L
H	0	-0.59138000	-2.42595800	-13.08409200	L
O	0	-2.21642000	-1.36823000	-13.78734500	L
H	0	-2.81407700	-2.12911700	-13.56675100	L
H	0	0.58280200	3.29385800	-4.27986400	L
C	0	0.84671600	2.43638900	-3.62517100	L
H	0	0.22228000	1.55629800	-3.88850500	L
H	0	0.64396500	2.71185200	-2.57021800	L
O	0	2.20291000	2.12902000	-3.76736900	L
H	0	2.30798400	1.76752400	-4.68487300	L
H	0	3.33978700	5.18564200	-10.99771000	L
C	0	4.03586200	5.60179000	-10.23984500	L
H	0	4.15003800	6.69532800	-10.39644700	L
H	0	5.02757100	5.12199400	-10.36173300	L
O	0	3.55524500	5.34284500	-8.95324700	L
H	0	2.72092200	5.87133100	-8.86174700	L
H	0	8.99106100	5.12529200	-8.46833600	L
C	0	9.41519600	5.15493400	-7.44608500	L
H	0	10.02389100	4.23890300	-7.28610300	L
H	0	8.57716400	5.18573100	-6.71759500	L
O	0	10.21268100	6.29548200	-7.31388300	L
H	0	10.54690100	6.28789100	-6.37973400	L
H	0	-2.12180200	3.38157000	-4.68277700	L
C	0	-2.66823800	3.39021800	-5.64640500	L
H	0	-2.37733700	4.30024200	-6.21163300	L
H	0	-3.75888000	3.41383400	-5.44107400	L
O	0	-2.33989300	2.23852200	-6.36709200	L
H	0	-2.73103300	2.35854000	-7.27013900	L
H	0	-1.70737100	6.84485800	-9.09079400	L

C	0	-1.58402100	7.19132300	-8.04408300	L
H	0	-2.00264500	6.43637400	-7.34807400	L
H	0	-2.13912800	8.14147900	-7.91965700	L
O	0	-0.23364900	7.40663200	-7.75929700	L
H	0	0.22319000	6.54522100	-7.94054300	L
H	0	4.14263400	5.79197800	-13.93713600	L
C	0	4.47917500	6.79362500	-13.59600900	L
H	0	5.44202100	6.68133200	-13.05746300	L
H	0	3.72844100	7.22324900	-12.90095000	L
O	0	4.65886400	7.64234800	-14.69217300	L
H	0	3.77519500	7.70000600	-15.14012400	L
H	0	3.03065300	1.93617500	-8.84117600	L
C	0	4.08278500	1.74326400	-9.12723200	L
H	0	4.66669300	1.52061100	-8.20919700	L
H	0	4.48836300	2.65034400	-9.62235800	L
O	0	4.12773700	0.65561600	-10.00323700	L
H	0	5.08549800	0.52236200	-10.22396600	L
H	0	0.16765100	1.70536000	-11.89292300	L
C	0	1.10275200	1.70056700	-11.29375600	L
H	0	1.82278200	1.00629000	-11.76862700	L
H	0	0.89160800	1.34379100	-10.26401400	L
O	0	1.66261000	2.98004300	-11.25136800	L
H	0	0.94836100	3.58276600	-10.91996000	L
H	0	4.44998800	-2.11700600	-7.12462900	L
C	0	5.18654200	-2.04091800	-7.94825100	L
H	0	5.99845400	-2.77420500	-7.76392800	L
H	0	5.60818800	-1.01330900	-7.96512800	L
O	0	4.54740300	-2.31926700	-9.15883200	L
H	0	5.24087200	-2.22408000	-9.86076300	L
H	0	2.93741000	-2.94589300	-11.58548200	L
C	0	2.45381200	-1.97385000	-11.81782100	L
H	0	1.92423400	-2.04266800	-12.79168600	L
H	0	3.23744400	-1.19483100	-11.89566900	L
O	0	1.55978400	-1.62254800	-10.80181800	L
H	0	0.87328500	-2.33811900	-10.77924100	L
H	0	1.69504900	-1.95404200	-7.73069400	L
C	0	1.55174200	-0.87487000	-7.50926900	L
H	0	0.89000800	-0.41613100	-8.27370000	L
H	0	2.53634900	-0.36954200	-7.55072800	L
O	0	1.00397000	-0.71112200	-6.23318700	L
H	0	0.12738900	-1.17567700	-6.24919400	L
H	0	7.55980100	4.41859200	-16.30910200	L
C	0	7.06355400	5.05288500	-15.54301600	L
H	0	6.16177300	4.52423200	-15.17529600	L

H	0	6.75280800	6.01842000	-15.99686300	L
O	0	7.92882000	5.28108100	-14.46924000	L
H	0	8.72797900	5.72770400	-14.85167000	L
H	0	8.33985100	8.85294300	-10.46417500	L
C	0	9.11292500	9.45009100	-9.93582300	L
H	0	9.57939700	8.83532400	-9.13613500	L
H	0	8.62600600	10.33232200	-9.47265900	L
O	0	10.08788600	9.88404500	-10.83904600	L
H	0	10.46889400	9.06269800	-11.24528300	L
H	0	-5.52051700	3.57521200	-11.23902300	L
C	0	-6.01607800	4.56894500	-11.20013200	L
H	0	-5.40143800	5.27329600	-10.60142000	L
H	0	-7.00325500	4.45708400	-10.70949100	L
O	0	-6.19958700	5.06744700	-12.49298900	L
H	0	-5.29274300	5.13556300	-12.88915600	L
H	0	1.56964700	-6.07379000	-12.34277400	L
C	0	0.76004900	-5.31339100	-12.37720900	L
H	0	1.04110500	-4.52858200	-13.10853300	L
H	0	0.64090000	-4.84475000	-11.37681700	L
O	0	-0.44085600	-5.90644200	-12.77878500	L
H	0	-0.66505000	-6.57258100	-12.07830800	L
H	0	2.09080200	11.11404100	-8.63985600	L
C	0	1.81421800	10.27979100	-7.96326900	L
H	0	2.57400300	9.47535700	-8.05555800	L
H	0	0.81924200	9.89059600	-8.26470500	L
O	0	1.77048400	10.75096200	-6.64844900	L
H	0	1.51997600	9.97305300	-6.08695700	L
H	0	-0.14584200	12.52604400	-4.27199000	L
C	0	0.61581100	13.26473400	-4.59917400	L
H	0	1.60200800	12.76410600	-4.70173700	L
H	0	0.69762600	14.05790200	-3.82946900	L
O	0	0.23697100	13.84280500	-5.81464500	L
H	0	0.18817300	13.09831900	-6.46865500	L
H	0	-1.46034600	-1.36428100	-9.14571900	L
C	0	-1.99868400	-0.42424100	-8.90939500	L
H	0	-1.66637200	-0.06408200	-7.91258400	L
H	0	-3.09063400	-0.63058300	-8.89162000	L
O	0	-1.70409600	0.53082100	-9.88550100	L
H	0	-2.22477000	1.33834600	-9.64144100	L
H	0	4.66900800	11.20710000	-7.74979500	L
C	0	5.75374700	11.36551400	-7.91275500	L
H	0	6.21453200	10.39918500	-8.20837900	L
H	0	6.20688400	11.72588900	-6.96464800	L
O	0	5.93329500	12.31358000	-8.92425300	L

H	0	6.91446500	12.40881700	-9.03766100	L
H	0	7.73197400	7.40786100	-5.51760400	L
C	0	7.78172500	7.27016000	-4.42001400	L
H	0	7.77424600	8.26885700	-3.93306800	L
H	0	8.72437500	6.73638800	-4.17355200	L
O	0	6.67737500	6.52247400	-4.00266300	L
H	0	6.77779900	6.41234400	-3.02206400	L
H	0	-0.91177600	6.16463100	-5.07625900	L
C	0	-1.05294900	6.23248400	-3.97701300	L
H	0	-0.33669500	6.97337800	-3.57033500	L
H	0	-0.84695900	5.24649000	-3.51105600	L
O	0	-2.35571500	6.64494000	-3.68335100	L
H	0	-2.95437900	5.95238300	-4.06348100	L
H	0	7.12358600	2.07829900	-10.37628100	L
C	0	7.55237400	2.68562400	-9.55462400	L
H	0	7.00614600	3.65172800	-9.50261700	L
H	0	8.62457400	2.87195600	-9.77177600	L
O	0	7.41978000	1.98743300	-8.35095000	L
H	0	7.78795800	2.58225900	-7.64774200	L
H	0	5.58540500	3.98249400	-7.38041300	L
C	0	5.40805000	4.22767900	-6.31154500	L
H	0	4.97269100	5.24488000	-6.22870900	L
H	0	6.37809000	4.20908600	-5.77633600	L
O	0	4.54733900	3.29187600	-5.73221100	L
H	0	3.75429600	3.24275000	-6.32569900	L
H	0	-2.49781200	6.06663900	-13.27363600	L
C	0	-2.91602200	6.37785500	-14.25382700	L
H	0	-3.80189400	7.02929200	-14.09432800	L
H	0	-3.23506300	5.47208000	-14.80870800	L
O	0	-1.94697100	7.05750500	-14.99787300	L
H	0	-1.68428100	7.84275500	-14.45106900	L
H	0	9.08226200	8.52186500	-13.63384900	L
C	0	7.98105100	8.66521900	-13.67916100	L
H	0	7.50241600	8.16358400	-12.81139500	L
H	0	7.59831200	8.20487400	-14.61260800	L
O	0	7.67079400	10.02859100	-13.68092800	L
H	0	8.03139100	10.39248700	-12.83122300	L
H	0	1.76647500	5.14240700	-13.01072900	L
C	0	0.91137800	5.04424700	-13.71182300	L
H	0	-0.04192100	5.20812800	-13.16566000	L
H	0	0.91065100	4.01738900	-14.13013300	L
O	0	1.03633200	5.96696600	-14.75457700	L
H	0	1.06096000	6.86136600	-14.32626700	L
H	0	-1.55539400	3.36689700	-10.88819600	L

C	0	-2.25805300	4.22348000	-10.83212900	L
H	0	-2.96384300	4.16300000	-11.68391100	L
H	0	-1.69052500	5.17532300	-10.90548200	L
O	0	-2.98154200	4.17520600	-9.63739300	L
H	0	-2.31871200	4.29605400	-8.91024500	L
H	0	7.61407700	7.51214400	-8.08132200	L
C	0	6.57554900	7.25189400	-8.36563000	L
H	0	6.48766600	6.14643400	-8.41737400	L
H	0	6.35827100	7.69247800	-9.36170700	L
O	0	5.69548500	7.76114900	-7.40694400	L
H	0	4.78324800	7.54270500	-7.72876000	L
H	0	-3.60470800	7.00323400	-11.00178900	L
C	0	-3.80692400	8.09387000	-11.05965100	L
H	0	-3.20323700	8.54531100	-11.87610500	L
H	0	-3.51347400	8.56136700	-10.09924400	L
O	0	-5.16742200	8.32370800	-11.28303700	L
H	0	-5.38534300	7.86896800	-12.13739300	L
H	0	-2.65816600	11.32415100	-9.83228900	L
C	0	-1.63812900	10.95308000	-10.07106800	L
H	0	-1.59887600	10.68646100	-11.14704100	L
H	0	-1.41931300	10.04452500	-9.47078800	L
O	0	-0.68889900	11.94568400	-9.80850000	L
H	0	-0.76300400	12.14548100	-8.83936200	L
H	0	2.62947200	1.44694900	-15.36556500	L
C	0	1.86084900	0.79524500	-14.90308300	L
H	0	1.11274000	1.43511800	-14.38802600	L
H	0	2.35539200	0.12759700	-14.16597900	L
O	0	1.24262800	0.03709100	-15.90202000	L
H	0	0.56369600	-0.52100800	-15.44178300	L
H	0	-2.52654400	8.98222100	-5.62097300	L
C	0	-1.63562400	9.52016100	-5.23486300	L
H	0	-0.73030100	8.89102700	-5.37128600	L
H	0	-1.77935100	9.71080100	-4.15254300	L
O	0	-1.48113400	10.73856000	-5.90161200	L
H	0	-1.31485700	10.51022700	-6.85230100	L
H	0	0.71105000	6.29266300	-10.57747500	L
C	0	0.88679100	7.34610800	-10.88534400	L
H	0	1.56807300	7.37203300	-11.76069700	L
H	0	1.36723300	7.89051100	-10.04729600	L
O	0	-0.32552400	7.96564700	-11.20101500	L
H	0	-0.70598200	7.45138400	-11.95899500	L
H	0	9.69602900	4.80728400	-11.17838600	L
C	0	9.37628200	5.86623000	-11.28318900	L
H	0	9.60730300	6.22955100	-12.30719700	L

H	0	9.93882500	6.48087600	-10.55128500	L
O	0	8.00734900	5.98616100	-11.02610000	L
H	0	7.55251100	5.39798900	-11.68208100	L
H	0	0.38511200	9.31235200	-13.70459600	L
C	0	1.25692700	9.97539500	-13.53144200	L
H	0	1.31436000	10.71300700	-14.36096100	L
H	0	2.17983000	9.35662900	-13.51519900	L
O	0	1.09559400	10.63395700	-12.30846100	L
H	0	1.88103300	11.23271800	-12.21407200	L
H	0	9.21523700	-2.85218800	-13.06672100	L
C	0	9.86114000	-2.12814200	-13.60375700	L
H	0	9.37458500	-1.86001000	-14.56622600	L
H	0	10.84544000	-2.60560800	-13.80051600	L
O	0	10.02572600	-0.98595800	-12.81431800	L
H	0	10.63921700	-0.39038100	-13.31772500	L
H	0	4.02306200	5.26865400	-16.52793200	L
C	0	3.51247900	4.28749800	-16.63568700	L
H	0	3.00561600	4.02152600	-15.68410900	L
H	0	4.27183500	3.51131400	-16.86249200	L
O	0	2.58344600	4.34092400	-17.67902400	L
H	0	1.93451500	5.04929500	-17.43037300	L
H	0	10.44182500	4.69908000	-4.30632300	L
C	0	10.17513300	3.62282600	-4.23167500	L
H	0	10.35907300	3.25971400	-3.19838900	L
H	0	10.81572100	3.05140700	-4.93256200	L
O	0	8.83450500	3.42816100	-4.57551500	L
H	0	8.31158500	4.02602100	-3.98182100	L
H	0	4.26078400	0.30339500	-5.97245500	L
C	0	5.05728500	0.18892100	-5.21138200	L
H	0	5.10510800	-0.87549000	-4.90008000	L
H	0	4.80067400	0.82440700	-4.33968700	L
O	0	6.27912900	0.59082900	-5.76047700	L
H	0	6.96149100	0.43568900	-5.05740400	L
H	0	-4.65357200	8.83305900	-7.84179000	L
C	0	-4.72616800	9.89247900	-7.51852600	L
H	0	-3.74653500	10.39627400	-7.65791900	L
H	0	-4.99001200	9.91623800	-6.44238900	L
O	0	-5.71586200	10.55540300	-8.25034900	L
H	0	-5.44209600	10.48918100	-9.20185100	L
H	0	-2.23880800	3.76689100	-17.11209700	L
C	0	-1.17741000	3.92947200	-16.83390700	L
H	0	-1.14022900	4.36179200	-15.81200600	L
H	0	-0.72004400	4.63862200	-17.55689600	L
O	0	-0.50166700	2.70602500	-16.86591300	L

H	0	0.42498100	2.89875400	-16.56900700	L
H	0	11.09942300	2.61531300	-9.84674900	L
C	0	11.41934000	2.04626700	-10.74644400	L
H	0	11.23982100	2.66984000	-11.64597800	L
H	0	10.82271900	1.11243700	-10.83474700	L
O	0	12.78160900	1.74306700	-10.66319400	L
H	0	12.88933900	1.19282800	-9.84458800	L
H	0	4.03962700	1.71823600	-12.71599300	L
C	0	4.43580400	2.66200500	-13.14089900	L
H	0	3.61034100	3.40213600	-13.19708000	L
H	0	4.82705500	2.45821900	-14.16063600	L
O	0	5.45879600	3.13947400	-12.31758400	L
H	0	5.76910600	3.98580700	-12.73039300	L
H	0	7.05222100	-1.15650000	-13.33684900	L
C	0	6.12548700	-0.65866200	-12.98221200	L
H	0	6.36764000	0.38089100	-12.68520300	L
H	0	5.72941500	-1.19374000	-12.09432300	L
O	0	5.17044900	-0.63640200	-14.00299800	L
H	0	4.95510000	-1.58540300	-14.19680300	L
H	0	3.37603000	9.17013400	-11.22454200	L
C	0	4.21176500	9.68683900	-10.71337600	L
H	0	4.36422200	9.21674600	-9.71948600	L
H	0	3.94707100	10.75851800	-10.58554800	L
O	0	5.36928500	9.56376200	-11.48656800	L
H	0	6.08280700	10.03764200	-10.98585500	L
H	0	6.12842000	-6.08403900	8.63755300	L
C	0	5.03277700	-6.18000400	8.79045500	L
H	0	4.81935000	-7.04233200	9.45590900	L
H	0	4.55231300	-6.36084000	7.80845700	L
O	0	4.51654200	-5.00631500	9.34585000	L
H	0	5.00579700	-4.86944500	10.19755200	L
H	0	3.31707100	4.72828400	9.89909600	L
C	0	3.11417800	3.72937500	10.34166700	L
H	0	2.21060400	3.30166900	9.86194300	L
H	0	2.92373800	3.83439300	11.43151800	L
O	0	4.19817500	2.87483600	10.12130400	L
H	0	4.97402900	3.29601400	10.57396800	L
H	0	13.47906700	-2.97744200	3.48639600	L
C	0	12.83190300	-2.42477700	4.19673400	L
H	0	13.25268900	-2.52795800	5.21987500	L
H	0	11.81141100	-2.86102700	4.16486100	L
O	0	12.79166600	-1.07811300	3.82587900	L
H	0	12.23692800	-0.62298900	4.50963200	L
H	0	12.50856400	-6.02982200	5.09716800	L

C	0	12.53747800	-7.13613800	5.19343000	L
H	0	11.91079500	-7.45661600	6.05299700	L
H	0	13.58369200	-7.45264500	5.37841200	L
O	0	12.08158300	-7.73271700	4.01526700	L
H	0	11.15306500	-7.40857300	3.88940300	L
H	0	7.67309700	3.99417300	10.52822300	L
C	0	8.12475200	3.64374700	11.47807300	L
H	0	9.04815900	4.23026600	11.67478800	L
H	0	8.37867000	2.56741400	11.37379900	L
O	0	7.20685800	3.82601100	12.51682600	L
H	0	7.64755000	3.47721200	13.33460800	L
H	0	15.33422300	-6.87384300	3.45300100	L
C	0	15.26611300	-5.89315400	2.93497300	L
H	0	14.42040100	-5.30705600	3.35262000	L
H	0	15.07850700	-6.07094600	1.85647000	L
O	0	16.46336100	-5.18608600	3.08260600	L
H	0	16.56760100	-5.02346600	4.05571800	L
H	0	11.22841700	3.28717800	4.62104700	L
C	0	12.03694700	2.63039100	4.23730100	L
H	0	12.36871100	1.93878700	5.04053300	L
H	0	12.89413300	3.26337600	3.93159000	L
O	0	11.58774500	1.90485200	3.13056700	L
H	0	10.82486300	1.35900300	3.45265700	L
H	0	7.83739500	-10.09074600	8.80847500	L
C	0	7.10432700	-9.53918100	9.43215300	L
H	0	6.72010500	-8.67615000	8.84827400	L
H	0	7.61578000	-9.17422400	10.34893600	L
O	0	6.05506700	-10.39763100	9.77435600	L
H	0	5.42518300	-9.85647400	10.31723900	L
H	0	12.91710700	3.26541700	-1.04099200	L
C	0	12.10162300	3.30407100	-0.29036300	L
H	0	12.02489100	2.31409600	0.20649900	L
H	0	11.14702600	3.53383300	-0.80844900	L
O	0	12.39120700	4.29519800	0.65223900	L
H	0	11.63957000	4.28432700	1.29951300	L
H	0	10.42840300	-3.20672300	11.08351800	L
C	0	10.35252400	-2.56818000	11.98940200	L
H	0	11.34137100	-2.11680200	12.21386300	L
H	0	10.04549100	-3.20017800	12.84748400	L
O	0	9.40128000	-1.56349300	11.79474700	L
H	0	9.69348300	-1.06513500	10.98874600	L
H	0	8.60121300	1.32661900	7.82893000	L
C	0	7.96108800	0.83653700	8.58969200	L
H	0	8.59422600	0.53938900	9.44905600	L

H	0	7.49411900	-0.07146100	8.15348900	L
O	0	6.97779900	1.72506400	9.03148200	L
H	0	6.40446300	1.91208700	8.24479200	L
H	0	10.99050600	-0.10565600	8.78486900	L
C	0	11.96203200	-0.64224700	8.74451600	L
H	0	11.87656800	-1.57763400	9.33313900	L
H	0	12.76040400	-0.01038700	9.18832900	L
O	0	12.28364900	-0.95855700	7.42232800	L
H	0	12.35481800	-0.09239200	6.94490200	L
H	0	11.97685300	5.88219400	9.09005500	L
C	0	11.94931700	6.75391700	9.77701600	L
H	0	11.75361400	6.39025500	10.80655700	L
H	0	11.12974600	7.44119300	9.47800100	L
O	0	13.17489000	7.42612300	9.75035300	L
H	0	13.30324000	7.72240300	8.81195500	L
H	0	9.98241700	-5.69779800	10.43131800	L
C	0	9.57708000	-6.30698600	11.26738800	L
H	0	9.98398000	-5.91520800	12.22202200	L
H	0	9.89564100	-7.36493300	11.14782200	L
O	0	8.18156100	-6.22567100	11.29541000	L
H	0	7.87053800	-6.55193000	10.41194900	L
H	0	8.70128100	7.09483100	11.41248900	L
C	0	7.89161200	7.22327600	10.66146500	L
H	0	7.52803600	8.27016200	10.70371300	L
H	0	7.04574400	6.54295400	10.89896100	L
O	0	8.37652200	6.95748600	9.37774900	L
H	0	8.72861300	6.03052900	9.40529100	L
H	0	7.93670000	0.84093900	-0.35046600	L
C	0	8.30103300	1.82705300	0.00205400	L
H	0	7.44636200	2.38273800	0.44364300	L
H	0	8.70890300	2.38921200	-0.86502500	L
O	0	9.29804700	1.62976200	0.96110700	L
H	0	9.58382900	2.53500500	1.24693500	L
H	0	16.27477000	-2.44144500	8.49945100	L
C	0	15.39857800	-1.75788700	8.51990400	L
H	0	14.56901900	-2.25832000	9.05818700	L
H	0	15.06830900	-1.53842100	7.48202200	L
O	0	15.72526200	-0.57136100	9.18352300	L
H	0	16.47533600	-0.17001500	8.67295200	L
H	0	12.85756600	3.76208300	10.78059300	L
C	0	13.89702400	3.69529000	11.16647300	L
H	0	13.88545200	3.30890100	12.20820300	L
H	0	14.34099500	4.71186100	11.16590500	L
O	0	14.66565300	2.85867100	10.35096500	L

H	0	14.21315500	1.97580200	10.35839900	L
H	0	3.37820900	-3.94890800	12.16246300	L
C	0	2.67400300	-3.19143000	11.75716100	L
H	0	1.73063300	-3.20477300	12.34162400	L
H	0	2.44287300	-3.44663400	10.70319900	L
O	0	3.25073000	-1.91999000	11.80731400	L
H	0	3.46250800	-1.75391800	12.76162500	L
H	0	16.93085400	-0.49676100	3.87471700	L
C	0	16.48018800	-1.49439400	3.68287800	L
H	0	17.20277000	-2.13417400	3.13175900	L
H	0	15.57339600	-1.36121000	3.05905200	L
O	0	16.12139400	-2.10176000	4.88952200	L
H	0	16.95944000	-2.18637400	5.41392600	L
H	0	5.25505600	-7.21709000	13.48160700	L
C	0	4.82794900	-7.37575800	12.47043300	L
H	0	5.65126700	-7.64430800	11.77444600	L
H	0	4.34334600	-6.43377000	12.13544100	L
O	0	3.88689700	-8.40823900	12.52619300	L
H	0	3.51927800	-8.49163200	11.60868000	L
H	0	15.86137700	-6.39164700	6.29204000	L
C	0	15.48456700	-5.72568800	7.09835800	L
H	0	16.28105000	-5.60148500	7.86027500	L
H	0	14.59548300	-6.18581600	7.58122600	L
O	0	15.15348700	-4.47311700	6.57444800	L
H	0	14.45309300	-4.63910500	5.89240000	L
H	0	8.61205200	-7.00637800	3.65481300	L
C	0	8.24583000	-5.97764800	3.45194000	L
H	0	7.15784200	-5.94230500	3.66077500	L
H	0	8.41352600	-5.72261400	2.38382200	L
O	0	8.90129200	-5.05771400	4.27551100	L
H	0	9.86969400	-5.16439200	4.09019200	L
H	0	13.08150300	5.88464600	6.56283300	L
C	0	14.02970500	5.32074000	6.43090000	L
H	0	14.65761900	5.84673900	5.68317500	L
H	0	13.80978300	4.29859400	6.05613400	L
O	0	14.72143500	5.25080400	7.64377700	L
H	0	14.13006000	4.75392100	8.26619000	L
H	0	4.37268800	-2.24634800	9.22594300	L
C	0	4.57678600	-1.36731900	8.57935300	L
H	0	3.72698600	-1.20226800	7.88393500	L
H	0	5.48561100	-1.56834300	7.98018600	L
O	0	4.78738200	-0.23032300	9.36343800	L
H	0	3.94369200	-0.07796300	9.86121500	L
H	0	15.25730600	2.08198700	5.28612400	L

C	0	15.16303600	1.21673100	5.97658300	L
H	0	16.16828200	0.79143600	6.18594000	L
H	0	14.54197300	0.43704500	5.49110400	L
O	0	14.54958400	1.61705700	7.16717800	L
H	0	15.14976700	2.29761600	7.56797500	L
H	0	8.36443700	-3.81516100	9.73428900	L
C	0	7.78153500	-3.10535800	9.11167000	L
H	0	7.45947300	-2.23913600	9.72817300	L
H	0	6.88061200	-3.62397200	8.73027700	L
O	0	8.55172500	-2.67001900	8.03041700	L
H	0	9.32038200	-2.18167400	8.42372800	L
H	0	3.06400600	-0.45971900	3.39502200	L
C	0	2.25742000	-1.22007400	3.44305800	L
H	0	1.28348600	-0.69740400	3.53469700	L
H	0	2.40224500	-1.86692000	4.33569700	L
O	0	2.25616300	-1.99195500	2.27689200	L
H	0	3.11742800	-2.48330500	2.27262900	L
H	0	5.94562500	-2.90724100	11.76017500	L
C	0	6.47375300	-3.50323900	12.53522100	L
H	0	7.52228800	-3.65721200	12.21283700	L
H	0	5.98713000	-4.49543100	12.64319500	L
O	0	6.46549100	-2.82590400	13.75832200	L
H	0	5.51023100	-2.69782000	13.99388000	L
H	0	3.87815700	0.42999800	14.28152700	L
C	0	3.49313900	1.42872400	13.98195100	L
H	0	4.34327000	2.12026000	13.79469900	L
H	0	2.87708900	1.83538600	14.80977000	L
O	0	2.70223300	1.31580200	12.83512400	L
H	0	3.29918600	0.96535000	12.12487300	L
H	0	4.64673800	-5.73606400	3.66363700	L
C	0	3.68468700	-5.93404100	4.18059900	L
H	0	3.88214800	-6.07980200	5.26124000	L
H	0	3.22539300	-6.86054500	3.77664400	L
O	0	2.81831900	-4.84995900	4.01360300	L
H	0	2.62646000	-4.80186500	3.04182900	L
H	0	7.94864800	-6.98824200	7.09251200	L
C	0	8.97233500	-6.69678300	7.40016100	L
H	0	8.91010000	-5.78553200	8.03215400	L
H	0	9.56899900	-6.48195400	6.48915300	L
O	0	9.55308800	-7.74628500	8.11810500	L
H	0	10.46404400	-7.43928200	8.36321400	L
H	0	9.46810300	5.11617600	7.13853200	L
C	0	9.85475800	5.91714500	6.47844400	L
H	0	10.31704000	5.45272200	5.58201100	L

H	0	10.61814000	6.49978600	7.03464600	L
O	0	8.79455900	6.74375200	6.10034100	L
H	0	9.19577700	7.47105200	5.55946700	L
H	0	5.56726500	-0.38392600	12.43301400	L
C	0	6.43272300	0.24444500	12.13733900	L
H	0	6.07188500	1.23364600	11.78388000	L
H	0	6.96856900	-0.25870900	11.30828500	L
O	0	7.30415800	0.40428500	13.21860900	L
H	0	6.77931500	0.85831300	13.92772800	L
H	0	4.54949600	-0.41485000	5.47678500	L
C	0	5.14327200	0.50673500	5.65577100	L
H	0	6.20749200	0.22529700	5.77894000	L
H	0	4.79855800	1.00278600	6.58728800	L
O	0	5.01894100	1.38113700	4.57284900	L
H	0	4.05399800	1.59871500	4.50733400	L
H	0	14.98228900	-1.77327400	11.83067400	L
C	0	14.21943400	-1.06458200	12.21304900	L
H	0	13.74211400	-1.50499600	13.11500400	L
H	0	13.45428100	-0.90451600	11.42450700	L
O	0	14.83833100	0.14656800	12.53672700	L
H	0	14.11704900	0.74129700	12.86927400	L
H	0	12.24055700	2.49008800	8.63267000	L
C	0	11.37975900	2.88145000	8.05100000	L
H	0	11.07173300	2.11653600	7.31087700	L
H	0	11.68239100	3.79988700	7.50668100	L
O	0	10.30488800	3.15498100	8.90198100	L
H	0	10.62837900	3.83838200	9.54344500	L
H	0	9.80676600	-0.96046000	4.01158400	L
C	0	9.13838600	-1.12277200	4.88286200	L
H	0	9.05004500	-2.21259400	5.06764000	L
H	0	8.13168800	-0.71390800	4.65969000	L
O	0	9.66609800	-0.50471900	6.01887900	L
H	0	9.82456900	0.44127700	5.76674100	L
H	0	12.70914800	-3.76108000	7.88233200	L
C	0	11.78657600	-4.37831900	7.83723300	L
H	0	11.11475700	-4.11298600	8.68126800	L
H	0	12.06883600	-5.44653800	7.92870000	L
O	0	11.13105100	-4.17908300	6.61913100	L
H	0	10.90649200	-3.21404700	6.58423800	L
H	0	6.60885400	-3.44723400	4.40355300	L
C	0	6.02614800	-3.27639600	5.33311900	L
H	0	4.98456100	-3.01222900	5.05796400	L
H	0	6.47315500	-2.43390800	5.90168800	L
O	0	6.01607900	-4.43433600	6.11554100	L

H	0	6.96582500	-4.63408500	6.32004500	L
H	0	2.64990000	1.31459900	8.09617500	L
C	0	1.95598000	1.63092300	7.28856000	L
H	0	2.31990300	2.58862700	6.86415400	L
H	0	0.93932600	1.78472200	7.70918800	L
O	0	1.91736700	0.66642700	6.27750200	L
H	0	1.63977000	-0.17934800	6.71487000	L
H	0	15.18127500	2.73207200	2.76005100	L
C	0	15.01318800	1.81596100	2.15800900	L
H	0	14.36054100	2.07135400	1.29676500	L
H	0	14.51309400	1.05446100	2.79456800	L
O	0	16.24426200	1.33429800	1.70269700	L
H	0	16.04194500	0.51310100	1.18386900	L
H	0	11.95269300	-5.11652600	2.63216900	L
C	0	11.84862100	-5.31358800	1.54409400	L
H	0	10.78029700	-5.49959000	1.31776800	L
H	0	12.43617100	-6.21421600	1.26764100	L
O	0	12.28502300	-4.20786600	0.80972700	L
H	0	13.23150700	-4.06725800	1.06858900	L
H	0	6.68489600	-8.41221400	4.64720000	L
C	0	6.09456200	-8.72617700	5.53325200	L
H	0	5.61738900	-7.83737300	5.99889400	L
H	0	6.77830700	-9.19056900	6.27200100	L
O	0	5.12430200	-9.66046800	5.15866400	L
H	0	4.50369500	-9.18077600	4.55180500	L
H	0	13.09285500	-4.39558800	12.19727400	L
C	0	13.27886400	-4.48361300	11.10728500	L
H	0	12.97090300	-3.53620000	10.61613500	L
H	0	12.67599000	-5.32735700	10.70745300	L
O	0	14.63994100	-4.71758400	10.89018900	L
H	0	14.75404000	-4.76647600	9.90594100	L
H	0	5.91216400	-1.50540800	2.99221400	L
C	0	5.89619200	-0.97558800	2.01735900	L
H	0	4.84751100	-0.88748100	1.67864400	L
H	0	6.46190100	-1.55410300	1.25826500	L
O	0	6.44310100	0.30152400	2.15591000	L
H	0	7.38258700	0.16366100	2.44198200	L
H	0	9.05592300	2.93556800	5.88615100	L
C	0	8.09701100	2.98168500	5.33344100	L
H	0	7.82633800	4.04674400	5.17300000	L
H	0	7.31144000	2.48808200	5.94024700	L
O	0	8.24165000	2.32387100	4.10918000	L
H	0	7.39047200	2.46760800	3.62116500	L
H	0	9.96021300	1.49997600	13.24206900	L

C	0	10.98655700	1.37636100	12.84060600	L
H	0	11.39125400	0.40088700	13.18726200	L
H	0	11.62202800	2.20438100	13.22242200	L
O	0	10.93992400	1.41007200	11.44421500	L
H	0	11.87556800	1.29178200	11.13850400	L
H	0	8.54383000	5.82503400	3.51055000	L
C	0	8.97720500	5.28256300	2.64479000	L
H	0	9.70760700	4.53791400	3.01901200	L
H	0	9.50523200	5.99698300	1.98078400	L
O	0	7.96874800	4.62334600	1.93826800	L
H	0	7.34577300	5.32932600	1.62963300	L
H	0	-0.63772600	10.72293000	3.94356600	L
C	0	-1.00521500	10.73349900	2.89584600	L
H	0	-1.91909100	10.11083200	2.81458300	L
H	0	-0.22150200	10.30247500	2.24243300	L
O	0	-1.27782000	12.04172000	2.48639600	L
H	0	-1.93166800	12.40129800	3.13927400	L
H	0	4.92344800	14.58216500	-3.42111700	L
C	0	4.00085600	14.46267500	-2.81997800	L
H	0	4.28467300	14.32268800	-1.75512400	L
H	0	3.45295100	13.56770500	-3.18365100	L
O	0	3.21018500	15.60653300	-2.96356100	L
H	0	2.40430300	15.45277300	-2.40622300	L
H	0	2.00499900	5.79632500	-2.81696900	L
C	0	2.92876600	5.20556000	-2.64085600	L
H	0	3.29064700	4.77674400	-3.59947400	L
H	0	2.69267200	4.37399300	-1.94809300	L
O	0	3.91808100	6.01029900	-2.06995200	L
H	0	4.10173700	6.72773800	-2.72938700	L
H	0	10.48042200	8.84455000	-3.19217300	L
C	0	10.34936600	9.84658700	-2.72951900	L
H	0	9.27331300	10.02632200	-2.52278000	L
H	0	10.70809900	10.61872000	-3.44045000	L
O	0	11.08936700	9.93278000	-1.54639600	L
H	0	10.72463700	9.23368900	-0.94459800	L
H	0	9.67232700	8.46962200	1.21474400	L
C	0	9.40125400	9.19752500	2.00544800	L
H	0	8.71878600	9.95775700	1.57110900	L
H	0	10.32940900	9.68645800	2.37257000	L
O	0	8.76140800	8.52326500	3.04877900	L
H	0	8.58923400	9.20211000	3.75062600	L
H	0	-0.18364100	5.26484500	2.93747900	L
C	0	-0.73756900	5.96222100	3.59646300	L
H	0	-0.20205900	6.03603700	4.56695400	L

H	0	-1.76131500	5.56293200	3.76069900	L
O	0	-0.80174300	7.21667500	2.98500900	L
H	0	-1.29863600	7.80015400	3.61446400	L
H	0	4.40747800	10.80727400	7.02423300	L
C	0	4.39159100	11.90970500	7.15266700	L
H	0	3.50362000	12.33314700	6.63901600	L
H	0	5.30697800	12.33542500	6.69364400	L
O	0	4.36219100	12.23576100	8.51177400	L
H	0	3.52913200	11.83315500	8.87071100	L
H	0	10.96586400	12.17779500	9.54416600	L
C	0	10.10193400	11.49038400	9.43783700	L
H	0	9.86851700	11.37595500	8.35833400	L
H	0	9.22700300	11.92823100	9.96501400	L
O	0	10.42955300	10.25066700	9.99519400	L
H	0	9.64341100	9.66567700	9.84009000	L
H	0	9.27531100	7.13019800	-1.58747200	L
C	0	9.77587200	6.16606900	-1.35944400	L
H	0	10.20190300	6.19713500	-0.33501200	L
H	0	10.60268600	6.01486600	-2.08267700	L
O	0	8.86647300	5.11039900	-1.47312800	L
H	0	8.13734400	5.30430800	-0.82975100	L
H	0	5.06944300	15.16917700	2.42273400	L
C	0	5.95563000	14.50714400	2.31726700	L
H	0	6.24155900	14.13372800	3.32129000	L
H	0	6.80565300	15.08304900	1.89274300	L
O	0	5.65436200	13.41892000	1.49355800	L
H	0	5.34746900	13.80293400	0.63198200	L
H	0	3.55704600	7.44160600	9.56345900	L
C	0	4.20432000	8.04178300	8.89218700	L
H	0	5.26585200	7.82509100	9.13625400	L
H	0	3.99236600	9.11973000	9.05997200	L
O	0	3.93461200	7.69750700	7.56423600	L
H	0	4.53094800	8.26155500	7.00774000	L
H	0	1.27323700	9.01354300	3.98655300	L
C	0	1.54071000	8.67768900	5.00883400	L
H	0	2.11263800	7.73014800	4.93923400	L
H	0	0.61357400	8.49615700	5.59143100	L
O	0	2.32674700	9.64547500	5.63863800	L
H	0	1.75757400	10.45465200	5.70861600	L
H	0	5.81469700	5.24657800	8.91775300	L
C	0	6.05395600	4.97899500	7.86877200	L
H	0	6.98578000	4.37395100	7.85955300	L
H	0	6.21110800	5.91252700	7.28869000	L
O	0	4.99110400	4.24792300	7.32871500	L

H	0	5.26792100	4.00007300	6.40916300	L
H	0	1.83629100	4.55053300	5.16230700	L
C	0	2.47177800	4.40527800	4.26415400	L
H	0	3.49257500	4.09612300	4.57274500	L
H	0	2.53985900	5.36839700	3.71877300	L
O	0	1.90774900	3.43990700	3.42428200	L
H	0	1.82576600	2.61510000	3.96941000	L
H	0	2.26911000	5.43927300	7.51838500	L
C	0	1.26785400	5.81644800	7.81773000	L
H	0	0.84738200	6.45264700	7.01059100	L
H	0	1.37550100	6.43023800	8.73386000	L
O	0	0.41268500	4.74459200	8.08384800	L
H	0	0.30629600	4.26217400	7.22482100	L
H	0	9.92397000	11.88449400	0.50845300	L
C	0	9.26262100	12.53766300	-0.10034100	L
H	0	8.29371000	12.02097700	-0.25902500	L
H	0	9.73580100	12.72721100	-1.08731400	L
O	0	9.03516500	13.74643100	0.56419500	L
H	0	9.92779700	14.15908500	0.69586800	L
H	0	10.72025200	14.19402600	5.70426800	L
C	0	11.21674300	13.33457500	6.20443800	L
H	0	10.52090200	12.47025700	6.24041000	L
H	0	11.47668000	13.62575100	7.24266200	L
O	0	12.38379500	12.98448200	5.51856200	L
H	0	12.09596300	12.72126100	4.60608100	L
H	0	-4.12384800	13.65396900	2.92870000	L
C	0	-4.26910700	14.62572900	2.41115400	L
H	0	-3.83703400	15.44495000	3.02526300	L
H	0	-5.35720600	14.80398700	2.28895400	L
O	0	-3.66368300	14.59782000	1.15140200	L
H	0	-2.70684000	14.39260500	1.31395400	L
H	0	6.56434500	12.72663400	-2.44538700	L
C	0	7.04694600	12.25292500	-3.32613600	L
H	0	6.47246500	12.49575800	-4.24540600	L
H	0	7.04480300	11.15357400	-3.18002100	L
O	0	8.36606200	12.69716700	-3.45530200	L
H	0	8.31882400	13.68699000	-3.50117500	L
H	0	0.90668700	10.71980000	8.51640000	L
C	0	0.52988900	9.67655000	8.51903100	L
H	0	1.37131600	8.99247000	8.28005500	L
H	0	-0.26120200	9.58007700	7.74522800	L
O	0	0.00958800	9.37782300	9.78188100	L
H	0	-0.32169300	8.44423900	9.72703300	L
H	0	5.90743500	8.47732000	0.50710800	L

C	0	6.61205700	7.90359600	-0.13156800	L
H	0	6.07210900	7.04695900	-0.58205700	L
H	0	7.44457900	7.51189800	0.48923100	L
O	0	7.11272700	8.71800100	-1.15116500	L
H	0	7.58946500	9.46053300	-0.69803100	L
H	0	1.80237100	9.65985200	-1.34285400	L
C	0	1.71193400	9.01803800	-2.24377600	L
H	0	1.26817600	8.04721800	-1.94634200	L
H	0	2.71693100	8.82957200	-2.67559600	L
O	0	0.88479500	9.63238000	-3.18710000	L
H	0	1.35708600	10.45749300	-3.46876200	L
H	0	7.09470100	8.44156800	7.33874700	L
C	0	7.33139100	9.52378300	7.32779800	L
H	0	6.77979900	9.99895800	6.48971800	L
H	0	8.42366400	9.65088800	7.17579500	L
O	0	6.94394400	10.08732500	8.54704800	L
H	0	7.14236100	11.05622500	8.47668400	L
H	0	-2.16159900	16.21440600	-1.07673200	L
C	0	-1.14236200	16.37392000	-0.66261500	L
H	0	-0.80560500	15.45653700	-0.13488600	L
H	0	-1.17848500	17.21048900	0.06454000	L
O	0	-0.24794000	16.69641900	-1.68781000	L
H	0	-0.26734700	15.93093300	-2.31847800	L
H	0	7.00822300	15.92112700	-2.45198500	L
C	0	7.05514700	15.81936100	-1.34605000	L
H	0	8.01584000	16.23268200	-0.97021300	L
H	0	7.00444900	14.74345100	-1.08543200	L
O	0	5.97850800	16.48505600	-0.75200400	L
H	0	6.05536100	17.43390000	-1.03159100	L
H	0	2.77691000	12.41966000	4.25458300	L
C	0	2.06770200	12.47777900	3.40332500	L
H	0	1.08098100	12.83232800	3.76719600	L
H	0	1.94673500	11.46538400	2.96863600	L
O	0	2.56153700	13.34148300	2.42236800	L
H	0	2.63970000	14.22976200	2.85536800	L
H	0	1.04768500	12.74582400	6.45803200	L
C	0	0.06288400	13.22064400	6.65120400	L
H	0	0.09464900	14.26761700	6.28635700	L
H	0	-0.14185200	13.22723400	7.74343700	L
O	0	-0.94774500	12.53046700	5.97568600	L
H	0	-0.92419700	11.60167500	6.32300500	L
H	0	0.55038500	15.56302600	3.76961700	L
C	0	-0.37768300	15.33249500	3.20298800	L
H	0	-1.05110200	14.70631200	3.82577500	L

H	0	-0.10830700	14.76355600	2.28994300	L
O	0	-1.02401700	16.51548800	2.83298100	L
H	0	-1.24801500	16.98117200	3.67991000	L
H	0	-1.80343300	8.62003700	0.73669400	L
C	0	-1.07688400	8.67996900	-0.10048600	L
H	0	-0.19897100	8.05217300	0.14701500	L
H	0	-0.73813800	9.72914800	-0.23424500	L
O	0	-1.66127800	8.20970100	-1.27863900	L
H	0	-2.43773800	8.80169400	-1.45190700	L
H	0	-3.29826200	13.35647200	-1.77087300	L
C	0	-2.23817800	13.03360600	-1.78535400	L
H	0	-1.83538000	13.10211400	-0.75251300	L
H	0	-1.66762400	13.70803600	-2.45840300	L
O	0	-2.16379500	11.71669700	-2.24837100	L
H	0	-1.20770600	11.45886000	-2.18754700	L
H	0	1.80994000	8.85634100	1.53722300	L
C	0	2.44658200	7.95014000	1.59233500	L
H	0	1.80739700	7.07248300	1.82633200	L
H	0	3.19667500	8.08937000	2.39920700	L
O	0	3.08470900	7.76741700	0.36339800	L
H	0	3.68327700	6.98717100	0.48078900	L
H	0	4.34724200	14.34572600	5.06064300	L
C	0	3.72764000	15.18595200	5.44122100	L
H	0	2.70429900	14.82087700	5.67498400	L
H	0	3.65764700	15.96313600	4.65284600	L
O	0	4.31517200	15.74048600	6.58240500	L
H	0	4.36418700	15.00700700	7.24884600	L
H	0	9.79971400	12.06533500	3.41010600	L
C	0	8.77461800	12.41235100	3.65189700	L
H	0	8.11787600	12.22535400	2.77594600	L
H	0	8.80793600	13.50140100	3.87073900	L
O	0	8.30313700	11.71041800	4.76447600	L
H	0	7.38847400	12.05326500	4.93570700	L
H	0	3.44068200	15.66457200	0.37968800	L
C	0	2.54267300	16.27982600	0.59030000	L
H	0	1.65255700	15.61591700	0.60205400	L
H	0	2.43278000	17.04107100	-0.21172500	L
O	0	2.69455900	16.90563300	1.83137000	L
H	0	1.85582000	17.41294900	1.98583100	L
H	0	12.91670500	7.27009900	4.24886800	L
C	0	12.17426600	6.90006700	3.51360800	L
H	0	11.16870100	7.26906700	3.80696700	L
H	0	12.18341200	5.78886000	3.52273200	L
O	0	12.51263000	7.37707200	2.24355200	L

H	0	11.82523000	7.01938300	1.62429500	L
H	0	4.17079300	11.74405100	-1.45072200	L
C	0	4.64080800	10.93657400	-0.85192300	L
H	0	5.66298900	11.24333900	-0.54308100	L
H	0	4.71303600	10.02693200	-1.48134000	L
O	0	3.86305500	10.65330000	0.27364700	L
H	0	3.82110700	11.49437600	0.79646200	L
H	0	0.78259000	13.78455000	-1.20607600	L
C	0	1.36364200	12.91037800	-0.85216300	L
H	0	2.32079200	13.27009400	-0.41899000	L
H	0	1.56869400	12.24619800	-1.71799600	L
O	0	0.62267400	12.22872100	0.11674500	L
H	0	1.17765400	11.45399100	0.39109400	L
H	0	6.85573100	16.22091800	-6.45723300	L
C	0	7.34799100	15.39176200	-5.90897000	L
H	0	6.69390400	15.08479300	-5.06574500	L
H	0	8.32328800	15.75021100	-5.51433700	L
O	0	7.54598900	14.31812300	-6.78244300	L
H	0	7.97933800	13.60520000	-6.24563400	L
H	0	5.80437000	9.96720500	-5.13385300	L
C	0	4.77908500	9.68056800	-4.81723900	L
H	0	4.85423100	8.85295400	-4.08328800	L
H	0	4.20020900	9.33083600	-5.69811400	L
O	0	4.13448100	10.76781100	-4.22033300	L
H	0	4.06588200	11.46400600	-4.92304100	L
H	0	1.11593100	5.75507000	-0.23080400	L
C	0	0.25670800	5.05399000	-0.17545200	L
H	0	-0.07648000	4.79145700	-1.20138900	L
H	0	-0.57926000	5.55218500	0.35450900	L
O	0	0.62112800	3.90070400	0.52445200	L
H	0	1.34379800	3.47818500	-0.00669800	L
H	0	12.13961400	10.26222800	7.00415300	L
C	0	11.59164400	9.37897700	6.61088300	L
H	0	10.87696800	9.03046200	7.38326200	L
H	0	12.30778900	8.55763400	6.39326300	L
O	0	10.88514000	9.72490700	5.45500700	L
H	0	11.56633700	10.01066300	4.79266900	L
H	0	5.10312200	9.84786100	4.61204700	L
C	0	5.06364700	10.45889600	3.68792900	L
H	0	5.11655700	11.53396100	3.96420800	L
H	0	4.10644400	10.25085000	3.16689000	L
O	0	6.14044300	10.11792100	2.86567200	L
H	0	6.07368000	10.70244900	2.06742000	L
H	0	5.94164700	5.67291800	4.55210400	L

C	0	5.62152600	6.72919500	4.42627900	L
H	0	6.23833800	7.37289500	5.08517000	L
H	0	4.55501500	6.83645300	4.71900100	L
O	0	5.80142300	7.13017900	3.10030800	L
H	0	5.21825000	6.54113800	2.55668700	L
H	0	5.12877800	4.88019800	0.59965100	L
C	0	4.36017500	4.17207000	0.97469800	L
H	0	3.95162200	3.60967800	0.11320600	L
H	0	3.52988300	4.73180500	1.45422500	L
O	0	4.93066200	3.27668400	1.88454700	L
H	0	5.26098800	3.82776400	2.64046100	L
H	0	8.66613300	14.44382700	8.14419900	L
C	0	7.84075600	14.02691100	7.53150200	L
H	0	6.91074800	14.01671800	8.14022000	L
H	0	8.10712300	12.99016700	7.23677300	L
O	0	7.66591200	14.82148600	6.39422900	L
H	0	6.91897600	14.41114900	5.88628200	L
Co	0	-1.21632300	-0.09843700	0.45353200	H
O	0	0.01469400	-3.73730400	-0.50211100	H
C	0	1.19265100	-3.81534900	-1.27386700	H
H	0	2.05359600	-4.17794000	-0.69160400	H
H	0	0.99861500	-4.53744200	-2.07261600	H
H	0	1.44712000	-2.85535000	-1.74472400	H
H	0	0.06449400	-2.98104300	0.11009400	H

TS13$_{explicit}$ (Doublet, S**2 after annihilation: 0.7591)
E= -1944.64249827 a.u.
Imaginary Frequency -- -505.5116

C	0	-4.15020400	-0.16429100	0.26066800	H
H	0	-4.05086000	0.91777200	0.32986600	H
H	0	-4.98260000	-0.40326900	-0.40361200	H
H	0	-4.28907000	-0.61690300	1.24450200	H
O	0	-2.91334000	-0.59763700	-0.33482100	H
C	0	-2.82725100	-1.95596400	-0.81460600	H
C	0	-4.11496700	-2.73433500	-0.79765000	H
H	0	-4.83180500	-2.37265300	-1.54258600	H
H	0	-3.90656400	-3.78400400	-1.01253300	H
H	0	-4.55547800	-2.69278600	0.19731700	H
H	0	-2.10623100	-2.40588300	0.06214900	H
C	0	-1.90434900	-2.12089200	-1.88934700	H
C	0	-2.01708600	-3.29935600	-2.77286500	H
H	0	-3.05291100	-3.49118100	-3.05954600	H
H	0	-1.40178300	-3.13167800	-3.65531400	H
H	0	-1.63279200	-4.20910300	-2.28283200	H
C	0	-0.69769700	-1.29116500	-2.11799300	H

O	0	-0.14495000	-1.34445100	-3.22569600	H
N	0	-0.23105400	-0.57293200	-1.03625600	H
C	0	1.07283900	-0.16022600	-1.00247700	H
C	0	2.08286300	-0.26941900	-1.97629200	H
H	0	1.81728100	-0.68153100	-2.93584900	H
C	0	3.37701200	0.11728000	-1.69653700	H
H	0	4.13789600	-0.02358000	-2.45479400	H
C	0	3.69145900	0.67438000	-0.45489600	H
H	0	4.69048000	1.01837800	-0.21919300	H
C	0	2.69973700	0.79017500	0.48977600	H
H	0	2.81941400	1.19525900	1.48536300	H
N	0	1.43896100	0.36797700	0.22185400	H
O	0	0.53581300	0.45767500	1.19132500	H
C	0	-1.32420900	-2.26753500	3.62122600	H
H	0	-0.92116400	-1.26270700	3.77260200	H
H	0	-2.00169400	-2.51818900	4.43616900	H
H	0	-0.47767800	-2.95672000	3.61028400	H
C	0	-2.05030800	-2.29343100	2.30663600	H
O	0	-1.24639100	-2.18913600	1.26312900	H
O	0	-3.26845300	-2.38801600	2.22056000	H
C	0	-2.67271400	2.84753000	2.69114300	H
H	0	-1.82364800	3.35596500	3.15263200	H
H	0	-3.26226300	3.59841200	2.16639900	H
H	0	-3.25824500	2.38404700	3.48712500	H
C	0	-2.15807800	1.82274400	1.72862300	H
O	0	-1.98891800	0.61740400	2.16270100	H
O	0	-1.89248400	2.10293000	0.53804400	H
H	0	-1.47615000	-9.62907100	3.84530400	L
C	0	-1.42603800	-10.70515600	3.58387100	L
H	0	-2.37751300	-11.18993500	3.88799700	L
H	0	-0.57793900	-11.16589600	4.13270000	L
O	0	-1.23543300	-10.83451800	2.20548000	L
H	0	-1.20782300	-11.80825800	2.02189200	L
H	0	-9.52568900	-9.51480700	-1.50663300	L
C	0	-9.69127500	-8.56220000	-0.96387500	L
H	0	-9.31884300	-7.72477400	-1.59128200	L
H	0	-9.12791300	-8.59423000	-0.00803100	L
O	0	-11.05703400	-8.40670300	-0.70978600	L
H	0	-11.15153200	-7.53540000	-0.24435700	L
H	0	1.26347300	-10.18305800	-2.32710800	L
C	0	1.10818500	-9.79240400	-1.29875800	L
H	0	0.55673600	-8.83009300	-1.34548400	L
H	0	2.09610000	-9.61141400	-0.83094500	L
O	0	0.39692300	-10.72037400	-0.53214000	L

H	0	-0.45015300	-10.88651900	-1.02101400	L
H	0	-2.47655000	-5.08153500	3.61719100	L
C	0	-3.49117000	-5.37065100	3.27596000	L
H	0	-4.22126200	-4.63884100	3.67968100	L
H	0	-3.71902300	-6.38274800	3.66607100	L
O	0	-3.52805600	-5.36942800	1.87786300	L
H	0	-4.46538300	-5.57602900	1.62627900	L
H	0	-6.10582200	-16.27880300	5.97619600	L
C	0	-5.92583000	-15.23158500	5.65892700	L
H	0	-4.83358100	-15.03068700	5.69352300	L
H	0	-6.45631800	-14.55033700	6.35846500	L
O	0	-6.40854400	-15.05656500	4.35872000	L
H	0	-6.22087000	-14.11142000	4.12317600	L
H	0	-8.21124300	-9.94487600	-7.74470300	L
C	0	-7.87141200	-10.74701400	-8.43437700	L
H	0	-6.99346700	-11.27117400	-7.99915300	L
H	0	-7.56992000	-10.28376800	-9.39593400	L
O	0	-8.90856800	-11.65570600	-8.66661700	L
H	0	-9.14686800	-12.03135100	-7.77980800	L
H	0	-7.12169400	-15.08954600	-0.07696900	L
C	0	-6.78181300	-14.56237200	0.83986500	L
H	0	-6.14547900	-13.69674300	0.55933800	L
H	0	-6.17833200	-15.26216200	1.45229100	L
O	0	-7.88252800	-14.13201800	1.58657700	L
H	0	-8.38875100	-13.51465300	0.99796600	L
H	0	-11.39991000	-9.66738200	-3.45232400	L
C	0	-11.55603300	-10.76589000	-3.39815000	L
H	0	-10.59363200	-11.27090500	-3.61199000	L
H	0	-12.30185000	-11.07899700	-4.15945700	L
O	0	-11.99097800	-11.13013200	-2.12063100	L
H	0	-12.84706700	-10.65041000	-1.97519300	L
H	0	-4.56526500	-6.96467100	-4.26583800	L
C	0	-4.58470400	-5.85965500	-4.36696300	L
H	0	-4.47056500	-5.41318600	-3.35937700	L
H	0	-5.55809600	-5.53289500	-4.79025300	L
O	0	-3.53390400	-5.43203000	-5.18249500	L
H	0	-3.70025500	-5.83316200	-6.07460300	L
H	0	-5.11175900	-14.64623100	-2.05103400	L
C	0	-5.24066500	-13.58400500	-2.34500400	L
H	0	-4.41966500	-12.98946600	-1.89881300	L
H	0	-6.20827600	-13.20439500	-1.95782500	L
O	0	-5.19686800	-13.45801000	-3.73551500	L
H	0	-5.94481700	-14.00956700	-4.08228300	L
H	0	-6.92358100	-7.19567200	-0.40931600	L

C	0	-5.99289400	-7.34051000	-0.99598900 L
H	0	-5.30865800	-8.01956600	-0.44818000 L
H	0	-5.49360500	-6.36050800	-1.12223400 L
O	0	-6.29283200	-7.87127300	-2.25306100 L
H	0	-6.77686300	-8.71909000	-2.08029300 L
H	0	-1.10060800	-11.68162700	-3.64910000 L
C	0	-2.00542300	-12.15452700	-4.08716500 L
H	0	-2.91368700	-11.73058500	-3.61231100 L
H	0	-1.97413300	-13.24268800	-3.88505700 L
O	0	-2.04138800	-11.94967400	-5.46905800 L
H	0	-2.07078100	-10.96741000	-5.60181900 L
H	0	-6.86625400	-4.28938500	-0.92915900 L
C	0	-7.28342800	-4.09907700	-1.94034400 L
H	0	-6.44956900	-4.03779500	-2.66743200 L
H	0	-7.82864300	-3.13325100	-1.94273000 L
O	0	-8.14117700	-5.13775000	-2.31259700 L
H	0	-8.87242700	-5.14020400	-1.64286000 L
H	0	-0.27789200	-15.08699900	-3.87290000 L
C	0	-0.02138800	-15.75052500	-4.72611300 L
H	0	-0.08901400	-16.80486600	-4.38838100 L
H	0	1.01850700	-15.54658900	-5.06053000 L
O	0	-0.91575500	-15.54901300	-5.78186900 L
H	0	-0.81731900	-14.59811800	-6.04717400 L
H	0	-3.40930000	-17.00530600	-4.99379900 L
C	0	-3.86490700	-17.01597000	-3.98033400 L
H	0	-4.03797500	-18.06881400	-3.67792100 L
H	0	-4.84045200	-16.48476500	-4.00185500 L
O	0	-3.00442100	-16.41117000	-3.05971900 L
H	0	-2.90516200	-15.47029800	-3.35689800 L
H	0	-4.32512300	-10.29796100	0.26674200 L
C	0	-3.42242000	-10.37706600	-0.37393400 L
H	0	-3.09046600	-11.43482200	-0.42510000 L
H	0	-2.61362000	-9.77161900	0.07987500 L
O	0	-3.69104000	-9.89107300	-1.65673300 L
H	0	-4.45429300	-10.42397900	-1.99866600 L
H	0	-9.65282900	-11.66906100	5.08121600 L
C	0	-8.74990900	-12.17988400	4.68904400 L
H	0	-9.05173500	-12.84187500	3.84879700 L
H	0	-8.29925900	-12.78661200	5.50355300 L
O	0	-7.83669800	-11.21932400	4.24543900 L
H	0	-7.07777700	-11.72347400	3.85347300 L
H	0	-6.32251100	-5.42942000	5.90385200 L
C	0	-6.74917600	-6.20966500	5.23847700 L
H	0	-6.63268000	-7.20912400	5.70872600 L

H	0	-6.19528300	-6.20652400	4.27934800	L
O	0	-8.09813800	-5.94196200	4.98758100	L
H	0	-8.56538500	-6.04519700	5.85619800	L
H	0	-5.72864100	-9.29480500	4.16364600	L
C	0	-4.88373400	-9.19904400	3.45219200	L
H	0	-4.68070100	-10.19214300	2.99795400	L
H	0	-3.98784300	-8.85143400	4.01014900	L
O	0	-5.22273200	-8.27680200	2.45886900	L
H	0	-4.43303300	-8.21878900	1.86114500	L
H	0	-0.52011400	-13.60573800	5.25873800	L
C	0	-1.09752600	-13.46173800	6.19461500	L
H	0	-1.68896800	-12.52540200	6.10921900	L
H	0	-0.38890800	-13.37844600	7.04593900	L
O	0	-1.94796900	-14.55572100	6.38128500	L
H	0	-2.43325000	-14.38347300	7.22923600	L
H	0	2.73039100	-11.02917000	3.13910600	L
C	0	2.24065200	-10.04730000	2.97049500	L
H	0	1.58859600	-9.81716400	3.83598600	L
H	0	1.61548200	-10.09242500	2.05280500	L
O	0	3.20828100	-9.04757800	2.84947200	L
H	0	3.73896300	-9.28006500	2.04463400	L
H	0	-6.29339800	-9.37404700	-6.07099800	L
C	0	-5.18569800	-9.34144200	-6.14413900	L
H	0	-4.87482600	-8.30191400	-6.36931700	L
H	0	-4.84070300	-10.00157300	-6.96806100	L
O	0	-4.61357900	-9.73688400	-4.93208600	L
H	0	-4.86671000	-10.68777900	-4.80760900	L
H	0	-2.91057200	-7.03781400	-2.12040300	H
C	0	-2.49811300	-6.53606300	-1.24276000	H
H	0	-2.68471200	-7.17835600	-0.36982000	H
H	0	-3.07533500	-5.60881300	-1.08693600	H
O	0	-1.13731400	-6.29607900	-1.47654300	H
H	0	-0.79850800	-5.82880600	-0.69407700	H
H	0	-3.18336100	-14.24607000	-7.52666900	L
C	0	-4.02398500	-14.40536400	-6.81830600	L
H	0	-3.63593800	-14.42468400	-5.77731300	L
H	0	-4.49806500	-15.38323900	-7.03988600	L
O	0	-4.97497800	-13.39071800	-6.96307200	L
H	0	-4.51565700	-12.54748100	-6.71453100	L
H	0	-9.50712600	-12.77707700	-1.69528900	L
C	0	-9.45339200	-13.84246200	-2.00595100	L
H	0	-9.56877600	-14.50069100	-1.11811100	L
H	0	-8.46140800	-14.03011000	-2.46238700	L
O	0	-10.44859500	-14.12348000	-2.94633200	L

H	0	-11.31250700	-13.94155100	-2.49371000	L
H	0	-2.20115800	-14.17085500	2.65620400	L
C	0	-3.25812900	-13.99553200	2.94226200	L
H	0	-3.29121800	-13.70925800	4.01479100	L
H	0	-3.83301800	-14.93326600	2.78558600	L
O	0	-3.78516200	-12.97121000	2.14977600	L
H	0	-4.70305900	-12.80897400	2.48847600	L
H	0	1.10422500	-12.90114100	1.40638900	L
C	0	1.31026700	-13.50586400	2.31300400	L
H	0	0.94719700	-12.94786500	3.20198900	L
H	0	2.40661700	-13.66780300	2.39355100	L
O	0	0.64769600	-14.73274400	2.20943600	L
H	0	0.82926500	-15.21340300	3.05837400	L
H	0	-10.22996500	-7.57016200	3.85538600	L
C	0	-9.97260200	-8.43596200	3.20791900	L
H	0	-10.59452600	-8.39399400	2.29062700	L
H	0	-10.19141700	-9.38369700	3.74546000	L
O	0	-8.62266500	-8.38368000	2.84897400	L
H	0	-8.11160100	-8.45836300	3.69544000	L
H	0	-8.75468900	-16.78494100	-2.91484700	L
C	0	-7.77557800	-17.06010700	-2.47230300	L
H	0	-7.91532500	-17.24396200	-1.38514600	L
H	0	-7.06566500	-16.21922100	-2.62302200	L
O	0	-7.29631800	-18.21314800	-3.10123000	L
H	0	-6.43584100	-18.42898600	-2.65706100	L
H	0	-10.16144700	-7.61317900	-4.72431900	L
C	0	-9.12494200	-7.99384900	-4.82072100	L
H	0	-9.02732100	-8.52664500	-5.79018600	L
H	0	-8.92432100	-8.69622000	-3.98464100	L
O	0	-8.23337800	-6.91926000	-4.77216100	L
H	0	-7.32894100	-7.31192300	-4.87837600	L
H	0	-10.30372300	-11.30598500	0.29899600	L
C	0	-11.01961000	-11.58703300	1.10020900	L
H	0	-11.93839500	-10.96749800	1.01431000	L
H	0	-10.54893500	-11.39322100	2.08534600	L
O	0	-11.34043700	-12.94457300	1.00487400	L
H	0	-11.75068900	-13.06620600	0.10960800	L
H	0	-14.55726900	-6.92832700	-3.08143100	L
C	0	-14.22865000	-7.98327300	-2.96468200	L
H	0	-15.05144200	-8.56524300	-2.50135300	L
H	0	-13.34082900	-8.02750900	-2.29792500	L
O	0	-13.92585400	-8.53285300	-4.21397500	L
H	0	-13.14319600	-8.02613000	-4.55316200	L
H	0	-1.08515500	-13.75550300	0.18293700	L

C	0	-1.61795000	-14.18638600	-0.69096300	L
H	0	-0.96418300	-14.91917200	-1.21107300	L
H	0	-1.86203100	-13.36642700	-1.39393100	L
O	0	-2.80110000	-14.80505000	-0.27719700	L
H	0	-2.52573300	-15.54143300	0.32775000	L
H	0	-7.02983600	-11.92833800	-5.45660600	L
C	0	-7.95444700	-11.77148100	-4.86140600	L
H	0	-8.48577300	-12.73832600	-4.76978400	L
H	0	-8.61748000	-11.04726600	-5.37973600	L
O	0	-7.63701200	-11.30329600	-3.58393000	L
H	0	-7.13454200	-10.46012700	-3.72073000	L
H	0	-6.73996600	-15.84025400	-5.70215300	L
C	0	-7.83518900	-15.72417600	-5.84746800	L
H	0	-8.27609600	-15.19305800	-4.97715700	L
H	0	-8.29397000	-16.73168100	-5.91753400	L
O	0	-8.09338700	-15.01888500	-7.02706200	L
H	0	-7.68454200	-14.12340400	-6.90558600	L
H	0	0.76691300	-13.07478000	-2.40575500	L
C	0	1.79362500	-13.43538100	-2.19261000	L
H	0	2.45913900	-12.55659700	-2.06123300	L
H	0	2.14530100	-14.04662600	-3.05128200	L
O	0	1.77721000	-14.20709800	-1.02716500	L
H	0	2.71642800	-14.48388400	-0.86790700	L
H	0	-1.24937200	-7.02293500	-4.45660500	L
C	0	-1.46622500	-8.03632000	-4.85737000	L
H	0	-0.57521800	-8.43029500	-5.39122800	L
H	0	-2.30642200	-7.96613900	-5.57609400	L
O	0	-1.82636400	-8.89542900	-3.81661800	L
H	0	-1.04056400	-8.93938400	-3.21409800	L
H	0	-11.76980400	-12.98871300	-7.22370300	L
C	0	-11.56402100	-13.45786900	-6.23994100	L
H	0	-12.47596800	-13.38375100	-5.60868600	L
H	0	-10.72798600	-12.91295200	-5.75362900	L
O	0	-11.21901000	-14.79860400	-6.43484800	L
H	0	-11.03275000	-15.16753100	-5.53275100	L
H	0	-0.73361900	-11.74124900	-9.61899600	L
C	0	-1.17051800	-11.78101600	-8.59780000	L
H	0	-0.66909500	-11.02105500	-7.96528400	L
H	0	-0.99770500	-12.78482500	-8.15291200	L
O	0	-2.54013300	-11.50325500	-8.64678300	L
H	0	-2.94039800	-12.20082600	-9.22779500	L
H	0	-8.86229900	-4.37363800	0.98796800	L
C	0	-8.68789800	-5.37746000	1.42938000	L
H	0	-9.02195400	-6.14682300	0.70584000	L

H	0	-9.28027800	-5.48878100	2.36161500	L
O	0	-7.32844200	-5.55852100	1.69872000	L
H	0	-7.06753000	-4.81236500	2.29700900	L
H	0	-1.88666200	-7.85068100	1.95559200	H
C	0	-0.89752300	-7.65877900	1.51761100	H
H	0	-0.85225700	-8.14104800	0.53951600	H
H	0	-0.13543200	-8.12719200	2.15607000	H
O	0	-0.65488300	-6.28999700	1.28635800	H
H	0	-0.69137100	-5.81729000	2.12092900	H
H	0	-5.85324300	-18.10815500	0.09976400	L
C	0	-4.87490400	-17.61386400	-0.07024300	L
H	0	-4.50435800	-17.21255300	0.89789900	L
H	0	-5.01642900	-16.78234600	-0.79265200	L
O	0	-3.97087200	-18.54871200	-0.58372800	L
H	0	-3.11410700	-18.06268700	-0.70398800	L
H	0	-10.95090900	-10.35758000	-6.37131800	L
C	0	-11.51989500	-9.70449900	-7.06665800	L
H	0	-10.95756700	-9.62543900	-8.01893100	L
H	0	-11.62493000	-8.69005900	-6.62655500	L
O	0	-12.78065000	-10.25140300	-7.32339300	L
H	0	-13.24028600	-10.30313200	-6.44571600	L
H	0	-8.11550500	-10.82752900	1.50263300	L
C	0	-7.20688700	-10.69816500	0.88301800	L
H	0	-6.37748300	-11.27404300	1.34392900	L
H	0	-6.94615800	-9.61951300	0.85360500	L
O	0	-7.46090400	-11.16806700	-0.40777000	L
H	0	-6.63215200	-10.99939900	-0.92496300	L
H	0	-8.60193400	-0.20405400	0.80234500	L
C	0	-7.84306000	-0.80227200	1.34253700	L
H	0	-8.27710700	-1.14208600	2.30644000	L
H	0	-6.95427600	-0.16438000	1.53670100	L
O	0	-7.48497000	-1.89978000	0.55561600	L
H	0	-6.83858400	-2.42402300	1.09376200	L
H	0	-8.11523200	6.27982400	-2.72508400	L
C	0	-8.11562800	5.17426500	-2.80627900	L
H	0	-9.16723500	4.81679500	-2.83195300	L
H	0	-7.59777100	4.75078900	-1.91901600	L
O	0	-7.45102500	4.80127900	-3.97778900	L
H	0	-7.48137300	3.81018800	-4.00630300	L
H	0	-5.67145100	4.46117300	-7.57147400	L
C	0	-5.93584600	5.53950200	-7.55428200	L
H	0	-7.03198400	5.65319100	-7.42305400	L
H	0	-5.64268200	5.99524600	-8.52203700	L
O	0	-5.25928500	6.18719800	-6.51695900	L

H	0	-5.55119300	5.74241100	-5.68021200	L
H	0	-8.22907600	9.61826100	-1.74010100	L
C	0	-9.26353700	9.80678800	-2.08806300	L
H	0	-9.79680300	10.40185600	-1.31764200	L
H	0	-9.77414500	8.83078800	-2.23040000	L
O	0	-9.22001100	10.51385800	-3.29295400	L
H	0	-10.16406100	10.63931700	-3.57123700	L
H	0	-6.92100600	-3.64183100	-10.07792800	L
C	0	-7.35823500	-2.91493800	-9.36441400	L
H	0	-8.11659700	-3.43672600	-8.74403700	L
H	0	-7.84065200	-2.09661900	-9.94042300	L
O	0	-6.34505200	-2.40041200	-8.55124700	L
H	0	-6.77807800	-1.72081400	-7.97303900	L
H	0	-14.37929000	1.54999300	2.89157700	L
C	0	-14.80762600	2.30848700	2.20487800	L
H	0	-13.97645000	2.88890200	1.75070200	L
H	0	-15.46439800	2.98981900	2.78781900	L
O	0	-15.54474500	1.66187800	1.20842600	L
H	0	-15.92270900	2.38138600	0.63971200	L
H	0	-3.88482600	-4.16011100	-9.07576300	L
C	0	-3.83949800	-3.99677900	-10.17441700	L
H	0	-4.07176100	-2.93556900	-10.40586300	L
H	0	-4.59692700	-4.64268400	-10.66282700	L
O	0	-2.57257900	-4.32882400	-10.66306400	L
H	0	-1.93109900	-3.74850400	-10.17765500	L
H	0	-5.46185200	-0.74690800	-10.53708400	L
C	0	-5.49119300	-0.40811300	-11.59315800	L
H	0	-4.47905500	-0.49839100	-12.03724300	L
H	0	-5.80013500	0.65652500	-11.61924600	L
O	0	-6.40654000	-1.17334500	-12.32168400	L
H	0	-6.08588700	-2.11075200	-12.26364900	L
H	0	-14.26294200	-1.06915600	-6.64402200	L
C	0	-14.01449000	-0.06231600	-7.03831600	L
H	0	-14.89438400	0.35986800	-7.56542000	L
H	0	-13.18220000	-0.15685300	-7.76306000	L
O	0	-13.62375700	0.78054300	-5.99419700	L
H	0	-14.40612800	0.84531300	-5.38858200	L
H	0	-7.53195300	1.14240600	-4.57234200	L
C	0	-7.80417400	0.06723600	-4.57138400	L
H	0	-7.66815400	-0.33502000	-5.59740300	L
H	0	-8.86621800	-0.03472900	-4.26598600	L
O	0	-6.98362200	-0.61370500	-3.66937000	L
H	0	-7.21295100	-1.57314600	-3.75854200	L
H	0	-12.33468200	3.69685700	-8.77287200	L

C	0	-11.97470100	2.64857500	-8.83905200	L
H	0	-12.04763900	2.18303100	-7.83632100	L
H	0	-10.91223800	2.63542400	-9.15840700	L
O	0	-12.75692600	1.92438000	-9.74284900	L
H	0	-12.66341400	2.38476900	-10.61664500	L
H	0	-14.24503200	-3.11262800	-4.61629600	L
C	0	-13.73581400	-2.34927900	-3.99269900	L
H	0	-13.64836700	-2.73441900	-2.95409300	L
H	0	-14.34556300	-1.42000000	-3.99715000	L
O	0	-12.46600000	-2.09918800	-4.52197500	L
H	0	-12.06930300	-1.38573500	-3.95867400	L
H	0	-7.87610700	7.71192100	-8.66348500	L
C	0	-8.72446700	7.30255600	-9.25139800	L
H	0	-9.35392500	6.65480300	-8.60374100	L
H	0	-8.31757500	6.69081700	-10.08104500	L
O	0	-9.48978300	8.34445200	-9.78331000	L
H	0	-9.80088500	8.87849000	-9.00715000	L
H	0	-12.55687000	-5.24152100	-2.11169500	L
C	0	-11.73879600	-5.31502600	-2.85719400	L
H	0	-11.15562800	-6.23960300	-2.65963400	L
H	0	-11.08227300	-4.42619100	-2.75292400	L
O	0	-12.28951100	-5.35663500	-4.14119500	L
H	0	-11.52524100	-5.46497300	-4.76404400	L
H	0	-12.93701900	-6.41318000	-6.82948000	L
C	0	-13.98394500	-6.57456500	-7.16534000	L
H	0	-14.27811200	-7.63082400	-6.98498000	L
H	0	-14.04472400	-6.36869500	-8.25351400	L
O	0	-14.84799200	-5.71045300	-6.48595400	L
H	0	-14.74361000	-5.91990300	-5.52202600	L
H	0	-10.11407800	1.09467000	-11.17659200	L
C	0	-9.01061800	1.21470900	-11.11801100	L
H	0	-8.57284800	0.28639500	-10.69883800	L
H	0	-8.76038800	2.06284300	-10.44547600	L
O	0	-8.48113100	1.43593900	-12.39279500	L
H	0	-8.90938900	2.26626000	-12.72649700	L
H	0	-9.89712200	-3.74678700	-11.82942600	L
C	0	-10.57477300	-3.32536800	-11.05576400	L
H	0	-10.29226900	-3.74578500	-10.07004600	L
H	0	-11.62581800	-3.61089000	-11.27722800	L
O	0	-10.45180600	-1.93351700	-11.01272300	L
H	0	-10.70344100	-1.60791700	-11.91554700	L
H	0	-9.44004100	2.45980600	-7.00468000	L
C	0	-8.92560100	3.44345600	-7.02420000	L
H	0	-8.73698900	3.74844900	-8.07629600	L

H	0	-7.95464500	3.34468000	-6.49889100	L
O	0	-9.70129100	4.40706700	-6.37408500	L
H	0	-10.53097800	4.49677400	-6.90965100	L
H	0	-15.75905200	4.38722000	-1.13914500	L
C	0	-14.70545400	4.73249900	-1.14221900	L
H	0	-14.17838700	4.27512300	-2.00565900	L
H	0	-14.22018800	4.41072400	-0.19731000	L
O	0	-14.68015900	6.12537600	-1.24400000	L
H	0	-13.72189300	6.37980200	-1.25658200	L
H	0	-12.98309700	10.22379500	-0.50900200	L
C	0	-12.96326700	9.54976800	-1.39237800	L
H	0	-12.47955200	8.58741000	-1.12007400	L
H	0	-12.37041700	10.03236400	-2.19557700	L
O	0	-14.26245100	9.32252900	-1.85638900	L
H	0	-14.76307300	8.93772700	-1.09085900	L
H	0	-12.47701900	5.86223400	-3.42492700	L
C	0	-11.61166200	6.52866800	-3.61369800	L
H	0	-10.94408200	6.04212500	-4.35667900	L
H	0	-11.06434800	6.68325800	-2.65929600	L
O	0	-12.07492800	7.75164700	-4.10716400	L
H	0	-11.26744200	8.29874500	-4.28644900	L
H	0	-7.01489100	2.12985200	-2.18127200	L
C	0	-7.53576500	1.63641900	-1.33461900	L
H	0	-7.29685800	2.16201400	-0.38662100	L
H	0	-7.17910100	0.59016500	-1.25795700	L
O	0	-8.91521400	1.63587100	-1.55578100	L
H	0	-9.18931800	2.58821700	-1.57616000	L
H	0	-4.97728800	3.67354400	-2.72119600	L
C	0	-4.56305800	3.92927300	-1.72445700	L
H	0	-4.90714300	3.17257600	-0.98782600	L
H	0	-4.93383100	4.93606800	-1.43351400	L
O	0	-3.16742800	3.92625600	-1.79303000	L
H	0	-2.85256000	4.21159900	-0.89656200	L
H	0	-8.40130200	-4.51987500	-6.33077100	L
C	0	-8.31039600	-3.65588100	-5.64326300	L
H	0	-8.27947200	-2.72318900	-6.24440900	L
H	0	-7.37061000	-3.75959900	-5.06213200	L
O	0	-9.41092500	-3.63980400	-4.78222000	L
H	0	-9.31750600	-2.82152000	-4.22992900	L
H	0	-13.42065800	2.05881400	-0.91274200	L
C	0	-12.72508800	1.19755300	-0.83980500	L
H	0	-12.76475300	0.77196000	0.18436100	L
H	0	-11.69389700	1.55208600	-1.03852700	L
O	0	-13.06734600	0.22455800	-1.78302700	L

H	0	-13.96356300	-0.10919200	-1.51920600	L
H	0	-9.96240100	5.48600600	-11.96339500	L
C	0	-10.56575400	4.85280500	-11.27773200	L
H	0	-10.00379100	4.67895600	-10.33523800	L
H	0	-10.75338300	3.87490000	-11.76584500	L
O	0	-11.78947800	5.47032900	-11.00212000	L
H	0	-11.56692400	6.34154600	-10.58294600	L
H	0	-14.99047100	2.75411300	-8.13827300	L
C	0	-15.72987200	3.27036300	-8.78607800	L
H	0	-16.57330400	3.64390700	-8.16612800	L
H	0	-16.12242500	2.54343500	-9.52624600	L
O	0	-15.12056700	4.33229900	-9.46180900	L
H	0	-14.76160300	4.93381200	-8.75891700	L
H	0	-9.88145100	2.39039700	-4.21493900	L
C	0	-10.96850600	2.17287200	-4.23404300	L
H	0	-11.14053000	1.15593600	-3.82181700	L
H	0	-11.32265100	2.21286900	-5.28556900	L
O	0	-11.63826300	3.12683900	-3.46363000	L
H	0	-12.59763400	2.87756300	-3.49519900	L
H	0	-4.70987800	1.01414500	-2.51749500	L
C	0	-4.12512700	0.51509000	-3.31483000	L
H	0	-4.56087000	0.78657900	-4.29998500	L
H	0	-4.18851500	-0.58305600	-3.16933500	L
O	0	-2.79453200	0.93479200	-3.23805100	L
H	0	-2.31945300	0.46922300	-3.97493600	L
H	0	-16.76493800	7.52005000	-2.72630100	L
C	0	-16.71218700	7.03453500	-3.72468000	L
H	0	-17.63162800	7.28403000	-4.29275100	L
H	0	-16.65634700	5.93115800	-3.60256100	L
O	0	-15.59776700	7.50179800	-4.42799900	L
H	0	-14.80564400	7.22357900	-3.89954000	L
H	0	-17.61570100	-2.59720900	-7.68873700	L
C	0	-16.54655500	-2.79962400	-7.90489900	L
H	0	-16.02186000	-3.00369800	-6.94668000	L
H	0	-16.10633100	-1.90357900	-8.39241800	L
O	0	-16.45117500	-3.90679400	-8.75370700	L
H	0	-15.48247600	-4.04089000	-8.92132300	L
H	0	-12.81489100	-3.33603000	0.87053800	L
C	0	-13.68925100	-2.65428600	0.90850100	L
H	0	-14.53304400	-3.18364300	1.40155800	L
H	0	-13.97348600	-2.37881500	-0.13011400	L
O	0	-13.34775900	-1.50941700	1.63473300	L
H	0	-14.16069800	-0.94082500	1.64751100	L
H	0	-15.01240500	2.09031800	-3.23693300	L

C	0	-15.98837500	1.66817800	-2.91557200	L
H	0	-16.06863200	1.71338300	-1.80881900	L
H	0	-16.03960000	0.60699900	-3.23368800	L
O	0	-17.04066300	2.37634100	-3.50377300	L
H	0	-16.92940900	3.31970800	-3.21749400	L
H	0	-8.95548200	0.00779900	-7.75087100	L
C	0	-9.94441700	-0.47497400	-7.89461100	L
H	0	-9.80350500	-1.52957600	-8.21268900	L
H	0	-10.49482200	0.06170700	-8.69264700	L
O	0	-10.68285700	-0.41467900	-6.70908700	L
H	0	-10.23859700	-1.03437600	-6.07647600	L
H	0	-11.41367200	7.20184400	-6.92950400	L
C	0	-12.28577500	7.13479500	-7.61067300	L
H	0	-12.32277700	6.11195600	-8.04240500	L
H	0	-12.16369100	7.88097600	-8.42471100	L
O	0	-13.45369800	7.39782000	-6.88860800	L
H	0	-14.20171900	7.30114300	-7.53331900	L
H	0	-6.54528900	-7.75603500	-8.56020900	L
C	0	-6.10906500	-6.73934200	-8.64083400	L
H	0	-6.58190600	-6.21404600	-9.49823800	L
H	0	-5.01531100	-6.83167100	-8.81312900	L
O	0	-6.35251100	-6.04176800	-7.45443100	L
H	0	-5.94236400	-5.14652300	-7.57421600	L
H	0	-12.96410500	-0.78931900	-10.27825000	L
C	0	-13.83584500	-1.24753400	-10.79246100	L
H	0	-14.21520700	-2.10469000	-10.19617700	L
H	0	-13.50797600	-1.62105700	-11.78409100	L
O	0	-14.84632800	-0.29759800	-10.97100600	L
H	0	-15.10314700	0.00336500	-10.06128500	L
H	0	-6.72128700	2.42607600	-8.77718000	L
C	0	-6.19132700	1.48821600	-8.52337200	L
H	0	-5.19157600	1.50478100	-9.00626500	L
H	0	-6.78215100	0.63075700	-8.90702800	L
O	0	-6.05999100	1.39747300	-7.13542400	L
H	0	-5.54011400	0.57064500	-6.96404700	L
H	0	-10.15492200	-2.91528700	-0.55067000	L
C	0	-10.66200400	-2.00061700	-0.91790700	L
H	0	-10.86190000	-1.32955100	-0.05579500	L
H	0	-11.62117700	-2.29361000	-1.39542900	L
O	0	-9.83679300	-1.35881400	-1.84496100	L
H	0	-10.35387000	-0.58078800	-2.17652000	L
H	0	-18.65185200	1.22225800	-6.48425500	L
C	0	-17.65435100	0.73807700	-6.51131400	L
H	0	-16.88048000	1.50463500	-6.29215200	L

H	0	-17.48816600	0.31671800	-7.52625400	L
O	0	-17.61212400	-0.28000800	-5.55354300	L
H	0	-16.69631700	-0.65927300	-5.59659100	L
H	0	-8.58110100	7.98842800	-4.48928700	L
C	0	-8.51160800	7.89114800	-5.59282100	L
H	0	-9.05894200	6.98078900	-5.90582700	L
H	0	-7.44844600	7.78670500	-5.89435300	L
O	0	-9.08322200	9.00674500	-6.21131400	L
H	0	-8.53139700	9.78395500	-5.93667200	L
H	0	-4.83349100	-2.80846800	-4.62624500	L
C	0	-4.65306900	-2.36837500	-5.62880800	L
H	0	-5.41915900	-1.59093600	-5.81510300	L
H	0	-4.74599100	-3.15371000	-6.40983200	L
O	0	-3.38539800	-1.78168800	-5.67424600	L
H	0	-2.73621200	-2.52986700	-5.63204300	L
H	0	-13.47412100	-3.32907700	-7.36912500	L
C	0	-12.51139800	-3.40783100	-7.91166200	L
H	0	-12.27915700	-2.42683800	-8.37657200	L
H	0	-12.60794100	-4.18227800	-8.70229400	L
O	0	-11.50719800	-3.76317500	-7.00821000	L
H	0	-10.66719700	-3.79713900	-7.53420500	L
H	0	-9.31390700	-7.56704600	-8.34546400	L
C	0	-9.82610200	-6.58267100	-8.28733900	L
H	0	-9.08584100	-5.78368500	-8.49311600	L
H	0	-10.23927400	-6.43325100	-7.26642500	L
O	0	-10.84863600	-6.51275900	-9.23813600	L
H	0	-11.48832000	-7.23459900	-9.00628100	L
H	0	-15.35297900	3.42222500	-5.60380600	L
C	0	-14.67537800	4.29272500	-5.49839800	L
H	0	-14.58387100	4.54438200	-4.42066800	L
H	0	-15.11062700	5.15261400	-6.05031500	L
O	0	-13.42467500	3.96610500	-6.02878300	L
H	0	-12.83674900	4.74175600	-5.83962000	L
H	0	2.88091600	-3.12491800	8.22170900	L
C	0	3.17898300	-3.48649100	7.21472300	L
H	0	4.28300000	-3.55890200	7.17613200	L
H	0	2.84634600	-2.76453800	6.43952900	L
O	0	2.62759700	-4.74668000	6.97174100	L
H	0	1.64545200	-4.63296900	7.04447300	L
H	0	-3.17664800	-6.56167400	7.91246100	L
C	0	-3.12813800	-7.27084800	7.06115100	L
H	0	-4.12652600	-7.32202000	6.57579200	L
H	0	-2.84923100	-8.27324900	7.44911800	L
O	0	-2.17021100	-6.82542400	6.14673300	L

H	0	-2.17566500	-7.48456000	5.40530400	L
H	0	-5.49503400	-12.16385000	8.99064400	L
C	0	-6.03020200	-13.08034400	9.31654800	L
H	0	-6.86882100	-13.29116200	8.61871800	L
H	0	-6.44296500	-12.90973800	10.33168300	L
O	0	-5.14983100	-14.16586000	9.35693800	L
H	0	-4.78562000	-14.25743700	8.43876000	L
H	0	-7.36785500	-1.70187400	14.86147900	L
C	0	-6.47123400	-1.05746000	14.75958700	L
H	0	-6.35901600	-0.77053400	13.69206900	L
H	0	-5.57878100	-1.63062100	15.09091400	L
O	0	-6.63567900	0.08225600	15.55251400	L
H	0	-5.81262900	0.62180000	15.42989200	L
H	0	2.10388600	-2.04733300	14.67411700	L
C	0	2.52920100	-1.02754400	14.78217300	L
H	0	2.05337800	-0.51151900	15.64368500	L
H	0	2.31472300	-0.45305100	13.85854900	L
O	0	3.91310000	-1.09919900	14.96726900	L
H	0	4.05165400	-1.64345400	15.78522500	L
H	0	2.33852600	-13.57766300	5.49620500	L
C	0	2.56093200	-12.55273200	5.86467700	L
H	0	1.69405400	-11.88772900	5.66372000	L
H	0	3.44492600	-12.16072500	5.32159600	L
O	0	2.84192600	-12.58275300	7.23406000	L
H	0	2.02138300	-12.92305000	7.67562200	L
H	0	-5.20458100	-2.99893600	7.38642000	L
C	0	-4.19917200	-3.33726300	7.71803500	L
H	0	-4.30301700	-4.18500300	8.42710000	L
H	0	-3.69203800	-2.50060100	8.23961400	L
O	0	-3.42994300	-3.72431200	6.61730600	L
H	0	-3.93109500	-4.46064600	6.18164300	L
H	0	-5.91537400	-8.18057300	13.82898600	L
C	0	-5.58446400	-8.93075900	14.57759200	L
H	0	-4.55081200	-8.69689200	14.91181000	L
H	0	-5.58837700	-9.93355700	14.10324100	L
O	0	-6.45569600	-8.93440600	15.67082700	L
H	0	-6.40903500	-8.02267300	16.05985200	L
H	0	-8.53106300	-3.05550500	10.47088000	L
C	0	-8.88943700	-3.27178100	11.49929600	L
H	0	-8.35044200	-4.16073000	11.88414200	L
H	0	-9.97793500	-3.49449900	11.47855200	L
O	0	-8.63430800	-2.18232700	12.33732700	L
H	0	-9.12617300	-1.41500800	11.94550500	L
H	0	-0.25912100	3.23747000	5.35382900	L

C	0	-0.87899800	2.46254900	5.85423800	L
H	0	-1.89482900	2.87611400	6.01626700	L
H	0	-0.43737500	2.19888000	6.83895300	L
O	0	-0.96897100	1.32154000	5.05143400	L
H	0	-0.03574600	1.05152900	4.85010300	L
H	0	-4.81962900	-6.04454300	12.74752600	L
C	0	-3.84208300	-6.39128600	12.35079900	L
H	0	-3.61880000	-7.38899700	12.77668000	L
H	0	-3.03992700	-5.68851100	12.65871900	L
O	0	-3.89070400	-6.48784600	10.95728800	L
H	0	-4.10210600	-5.57724500	10.62725300	L
H	0	-3.54358000	-0.28544300	16.44461300	L
C	0	-2.58527700	-0.79411000	16.20410000	L
H	0	-2.60854100	-1.83774800	16.58537300	L
H	0	-1.75654200	-0.25004600	16.70177100	L
O	0	-2.37135200	-0.78833800	14.82326900	L
H	0	-3.11996200	-1.30592900	14.42891400	L
H	0	0.65954800	-3.80914600	13.66824800	L
C	0	-0.33810400	-3.50310000	13.28774500	L
H	0	-0.89103800	-2.95443500	14.07869100	L
H	0	-0.90994100	-4.41463800	13.02214200	L
O	0	-0.19508000	-2.70024700	12.15233100	L
H	0	0.30133400	-1.89390100	12.44889000	L
H	0	-3.45747100	2.03134600	14.92190400	L
C	0	-3.86633500	2.48513500	13.99322800	L
H	0	-3.24638400	3.36437000	13.72324100	L
H	0	-4.91091800	2.82165600	14.16858200	L
O	0	-3.82963600	1.56049900	12.94613800	L
H	0	-4.36558300	0.78294700	13.24842300	L
H	0	-11.70726300	-3.07360400	3.49128200	L
C	0	-10.68544100	-2.69698300	3.28242500	L
H	0	-10.63860900	-1.62066600	3.54876900	L
H	0	-10.47220100	-2.82292600	2.19938900	L
O	0	-9.76590300	-3.42044100	4.04661200	L
H	0	-8.87053900	-3.07533000	3.79742400	L
H	0	0.97337500	-5.39024600	11.35399100	L
C	0	1.92511900	-5.96033400	11.40123500	L
H	0	1.71335500	-7.04180400	11.53189400	L
H	0	2.50592200	-5.60715800	12.27551800	L
O	0	2.67172100	-5.75373000	10.23803400	L
H	0	2.08742600	-6.03166600	9.48693900	L
H	0	0.03827500	-8.10753000	9.84841300	L
C	0	-0.88767600	-8.47369400	10.34054900	L
H	0	-0.85316000	-9.58015200	10.38165000	L

H	0	-1.77664100	-8.16396300	9.75090100	L
O	0	-0.97271700	-7.97065200	11.64061800	L
H	0	-0.99711800	-6.98336000	11.55091400	L
H	0	-1.16913600	-3.92762200	17.56117000	L
C	0	-0.18945400	-4.16165100	17.09108800	L
H	0	-0.35165000	-4.59713700	16.08218300	L
H	0	0.33946100	-4.90819900	17.71812600	L
O	0	0.58985700	-3.00445600	16.99905600	L
H	0	0.08035700	-2.37626100	16.42437700	L
H	0	-0.09853400	-8.57029700	7.38779500	L
C	0	-0.05827500	-9.56028700	6.88519600	L
H	0	0.77288400	-9.56936500	6.14970700	L
H	0	-1.01379900	-9.72635600	6.35049000	L
O	0	0.12016900	-10.57871600	7.82493700	L
H	0	0.96312500	-10.36697100	8.30173100	L
H	0	-2.58387900	-11.98995100	10.82258700	L
C	0	-2.08839400	-12.38269900	9.90914200	L
H	0	-2.24002100	-13.48036900	9.86514400	L
H	0	-0.99766900	-12.17437500	9.95380900	L
O	0	-2.64597000	-11.79866800	8.76826300	L
H	0	-2.48667500	-10.82343800	8.85290200	L
H	0	1.61377100	-6.89013100	5.42172100	L
C	0	0.93102100	-6.73490200	4.56161200	L
H	0	0.16897900	-7.53896600	4.56458600	L
H	0	1.50426700	-6.79184500	3.61138500	L
O	0	0.29350100	-5.49553000	4.67161400	L
H	0	1.00503300	-4.81241700	4.56677700	L
H	0	-8.83390400	-8.55607800	8.00019300	L
C	0	-8.69627400	-9.20333900	7.10839500	L
H	0	-9.04514800	-8.65350200	6.21082600	L
H	0	-9.30208600	-10.12814000	7.22054600	L
O	0	-7.34526000	-9.52647700	6.95272900	L
H	0	-7.08962000	-10.03356600	7.76603200	L
H	0	-3.30861000	-0.45176500	10.15492200	L
C	0	-2.20025300	-0.42305500	10.16931600	L
H	0	-1.85665000	0.26150600	9.36919200	L
H	0	-1.79419600	-1.43914300	9.97512600	L
O	0	-1.74485400	0.05240700	11.40150300	L
H	0	-2.06673700	-0.59552800	12.07946500	L
H	0	0.95889500	1.78854600	14.60508600	L
C	0	0.00587000	1.67360400	14.04907400	L
H	0	-0.83793600	1.74401900	14.76834900	L
H	0	-0.00314300	0.67730100	13.55656200	L
O	0	-0.09050300	2.68840000	13.09245800	L

H	0	-0.94204200	2.52896200	12.60928800	L
H	0	0.18092500	-4.13894200	9.10788900	L
C	0	-0.71314600	-4.74320300	8.84244900	L
H	0	-0.48290700	-5.82442700	8.94709500	L
H	0	-0.98372200	-4.54422800	7.78640600	L
O	0	-1.78745400	-4.39325200	9.66411500	L
H	0	-1.51885500	-4.64072800	10.58571700	L
H	0	-8.56254500	-7.63060800	13.91342300	L
C	0	-8.96615100	-7.25428700	12.94933400	L
H	0	-9.41997300	-6.25003400	13.09533600	L
H	0	-9.75279000	-7.95181700	12.59635300	L
O	0	-7.94712300	-7.19176400	11.99473900	L
H	0	-7.29382700	-6.52695900	12.33338900	L
H	0	-8.47966600	-11.09837200	9.85352200	L
C	0	-8.42380300	-10.30715500	10.62888400	L
H	0	-9.44637600	-9.91259300	10.81162400	L
H	0	-7.76856900	-9.49058800	10.25830300	L
O	0	-7.90022600	-10.85235100	11.80537000	L
H	0	-7.83606900	-10.10145600	12.45068200	L
H	0	-6.42314800	-0.39327000	8.50318900	L
C	0	-6.36384300	-0.65165100	9.57930900	L
H	0	-5.71980600	-1.54785900	9.69378600	L
H	0	-7.38542900	-0.87683400	9.94956700	L
O	0	-5.82366900	0.42826600	10.28326100	L
H	0	-5.81763400	0.15527800	11.23633700	L
H	0	-6.63386000	-5.68911300	14.65209000	L
C	0	-7.19492200	-4.88248700	15.17005600	L
H	0	-7.93252800	-4.42966300	14.47333000	L
H	0	-6.47857500	-4.09946500	15.48922900	L
O	0	-7.84445800	-5.39346100	16.29769100	L
H	0	-8.47743500	-6.07971400	15.96156200	L
H	0	-11.41018500	-1.70118500	8.46110300	L
C	0	-11.14272900	-0.92245300	9.20753300	L
H	0	-11.12136000	-1.38665300	10.21446800	L
H	0	-11.90803500	-0.11652500	9.20106800	L
O	0	-9.88248400	-0.39083800	8.92066200	L
H	0	-9.95179200	0.00089700	8.01204500	L
H	0	1.13725500	-7.41718900	15.50544000	L
C	0	0.43796900	-7.31022200	14.65114200	L
H	0	0.59397300	-8.15921300	13.95234300	L
H	0	0.65353600	-6.35208300	14.13220200	L
O	0	-0.87534400	-7.31845800	15.13004000	L
H	0	-1.45498200	-7.14263000	14.34443900	L
H	0	-3.51275300	-3.24348900	12.59749400	L

C	0	-4.26538500	-2.78752100	11.92041800	L
H	0	-3.89969800	-2.87719500	10.87836500	L
H	0	-4.38917300	-1.71290500	12.17019400	L
O	0	-5.49134500	-3.44943500	12.03218700	L
H	0	-5.75345200	-3.38033800	12.98590100	L
H	0	-11.63487600	-3.35040000	6.21784300	L
C	0	-12.25139300	-4.23487000	6.48440200	L
H	0	-13.23886400	-4.16827200	5.97871100	L
H	0	-12.40956000	-4.23922900	7.58233400	L
O	0	-11.59429300	-5.41186800	6.11087800	L
H	0	-11.49170000	-5.36818300	5.12499300	L
H	0	-5.73789700	-6.33508800	8.32116600	L
C	0	-6.68619200	-6.34529200	8.89758000	L
H	0	-6.73595500	-7.28260800	9.48628800	L
H	0	-7.54985900	-6.31763800	8.20067100	L
O	0	-6.73262700	-5.25313200	9.76842200	L
H	0	-6.70092800	-4.44392900	9.19585700	L
H	0	-3.67398900	-9.51610300	11.18618700	L
C	0	-4.73467800	-9.68977600	10.90459600	L
H	0	-5.38876800	-8.95903400	11.42597000	L
H	0	-5.02179400	-10.71364000	11.21864300	L
O	0	-4.89272800	-9.57101600	9.52106900	L
H	0	-4.58553200	-8.65744900	9.28878300	L
H	0	2.37012800	-4.83414800	15.60698200	L
C	0	3.38001600	-4.97576000	15.17266500	L
H	0	3.46757500	-4.34621900	14.26314500	L
H	0	3.50693000	-6.04597400	14.90178400	L
O	0	4.34223200	-4.59846600	16.11403900	L
H	0	5.22110300	-4.71624800	15.66856300	L
H	0	0.54396900	0.58864900	8.87980500	L
C	0	1.18935200	0.16073600	9.67661700	L
H	0	2.22226300	0.03496200	9.29179300	L
H	0	0.79342400	-0.83434800	9.96312900	L
O	0	1.19003000	0.99318500	10.79898800	L
H	0	1.53133900	1.87125100	10.48848200	L
H	0	-10.45074900	-6.50953400	8.53443900	L
C	0	-10.42349000	-6.48280900	9.64283700	L
H	0	-9.50055300	-5.95600600	9.96544700	L
H	0	-10.41332900	-7.52615500	10.02502400	L
O	0	-11.55222500	-5.80609200	10.11459100	L
H	0	-11.47130200	-5.79732300	11.10365500	L
H	0	4.73481800	-8.86952800	7.70615700	L
C	0	3.69234100	-9.25041300	7.68876200	L
H	0	3.61321900	-10.09593700	8.40163500	L

H	0	3.43921700	-9.61381100	6.66937500	L
O	0	2.80639100	-8.24087000	8.07394900	L
H	0	2.90899800	-7.51849200	7.40276300	L
H	0	0.54336400	-1.08188900	6.13688100	L
C	0	0.01180900	-1.79345300	6.80219400	L
H	0	0.63604700	-1.97939300	7.69891400	L
H	0	-0.14379300	-2.75652800	6.27194700	L
O	0	-1.21538300	-1.25978100	7.20436300	L
H	0	-1.71578000	-1.05983400	6.37243200	L
H	0	2.18509000	-9.36259500	10.53220600	L
C	0	2.29195300	-10.09511500	11.35956400	L
H	0	1.80264100	-9.68277900	12.26513000	L
H	0	3.36963800	-10.25892200	11.57655100	L
O	0	1.67792800	-11.30353300	11.01589200	L
H	0	2.15703000	-11.63503600	10.21271600	L
H	0	-4.22335100	-12.59527900	6.51849100	L
C	0	-4.83105000	-11.79588300	6.04573700	L
H	0	-5.89086500	-11.93550300	6.33868300	L
H	0	-4.75367400	-11.87113600	4.94106500	L
O	0	-4.39596300	-10.53984700	6.47751000	L
H	0	-3.45453000	-10.45924700	6.17586600	L
H	0	-2.39033400	-10.51104900	14.92960800	L
C	0	-2.23165700	-10.49809200	13.83197300	L
H	0	-2.40642700	-9.46637500	13.46213700	L
H	0	-2.95677400	-11.19384900	13.35705600	L
O	0	-0.92053300	-10.89575100	13.55220100	L
H	0	-0.83708200	-10.88764100	12.56372900	L
H	0	-7.82249100	-3.29655200	6.33026300	L
C	0	-8.52059100	-2.95780100	7.12133100	L
H	0	-9.28621900	-2.29474400	6.66581700	L
H	0	-7.94553300	-2.39725900	7.88654200	L
O	0	-9.13345800	-4.07092300	7.70144700	L
H	0	-9.76612300	-3.71431600	8.37683400	L
H	0	-2.82767100	-5.95053200	16.59030200	L
C	0	-3.67043500	-5.44175600	16.08012400	L
H	0	-4.37964500	-5.06957900	16.85083100	L
H	0	-4.18279500	-6.17285300	15.41947600	L
O	0	-3.17402500	-4.37678300	15.32282600	L
H	0	-3.96733900	-3.92225000	14.93875700	L
H	0	-4.61540300	11.85006900	0.74655200	L
C	0	-4.22771400	10.87297500	0.38869600	L
H	0	-3.13356300	10.96045600	0.23715700	L
H	0	-4.70318700	10.60947100	-0.57992700	L
O	0	-4.48030600	9.87595400	1.33366300	L

H	0	-5.46181600	9.87001700	1.46803700	L
H	0	-4.44825900	-2.04421000	4.71285500	L
C	0	-5.45927900	-1.66241300	4.45989100	L
H	0	-5.35078100	-0.72855500	3.87116200	L
H	0	-6.01586100	-1.44138800	5.39599300	L
O	0	-6.15292400	-2.60690100	3.70056200	L
H	0	-6.27920600	-3.39193200	4.29187700	L
H	0	-10.14326100	8.74573100	2.34081300	L
C	0	-10.72233700	9.26508500	1.54981400	L
H	0	-11.80622900	9.13150300	1.75180700	L
H	0	-10.46911400	8.81409600	0.56763300	L
O	0	-10.39222200	10.62355600	1.55545800	L
H	0	-10.96029000	11.04771300	0.86151000	L
H	0	-11.77646300	1.86609700	2.34926700	L
C	0	-10.83331200	1.28822200	2.43733100	L
H	0	-10.94200100	0.30842600	1.92517100	L
H	0	-10.62652300	1.10653400	3.50975700	L
O	0	-9.77543800	2.01236700	1.88183100	L
H	0	-9.97985100	2.09382700	0.91545600	L
H	0	-3.08036700	14.40927300	5.40436300	L
C	0	-3.02148200	15.27364000	6.10067900	L
H	0	-4.00234100	15.79420400	6.13681200	L
H	0	-2.25526800	15.98210700	5.72557100	L
O	0	-2.65846000	14.84281200	7.38039600	L
H	0	-3.33903300	14.17251000	7.64685100	L
H	0	-4.95689100	3.28809700	5.29367400	L
C	0	-4.79534100	4.36952200	5.47582400	L
H	0	-5.54908800	4.94279800	4.89620800	L
H	0	-3.77292600	4.64006100	5.13623300	L
O	0	-4.93518200	4.63304600	6.84148100	L
H	0	-4.79983300	5.60947400	6.94675600	L
H	0	-3.14961600	7.58727700	7.01793500	L
C	0	-2.07203100	7.31998300	7.05047800	L
H	0	-1.62539500	7.66843700	8.00634200	L
H	0	-1.55402300	7.82669200	6.21200500	L
O	0	-1.91625600	5.93731400	6.91870500	L
H	0	-2.31483700	5.53802900	7.73416200	L
H	0	-10.78594100	7.26544900	8.77759600	L
C	0	-10.65226000	8.31313700	9.12267400	L
H	0	-11.15326800	9.00616500	8.41388800	L
H	0	-9.56824900	8.54692400	9.14683200	L
O	0	-11.18356900	8.47012600	10.40616100	L
H	0	-12.14111600	8.21893400	10.33776200	L
H	0	-4.06909400	8.26707300	9.34857200	L

C	0	-3.98417300	7.43644900	10.07664200	L
H	0	-4.03138800	6.47433200	9.52385300	L
H	0	-4.83097100	7.50144900	10.79315500	L
O	0	-2.76637000	7.54571300	10.75425300	L
H	0	-2.73945900	6.78562100	11.39125200	L
H	0	-7.53176700	3.74804300	1.49101900	L
C	0	-6.51496400	3.97744100	1.87415500	L
H	0	-6.58580400	4.82785200	2.58102500	L
H	0	-5.85212200	4.27264700	1.03350700	L
O	0	-5.99061600	2.86683800	2.54041800	L
H	0	-5.89793000	2.15517700	1.85559200	L
H	0	-10.62055600	10.92712900	6.62077500	L
C	0	-9.75126400	10.52857600	6.05681300	L
H	0	-9.45726400	9.55825800	6.50122000	L
H	0	-8.89593600	11.23127400	6.13684200	L
O	0	-10.09150100	10.33356700	4.71572100	L
H	0	-10.38960400	11.21716400	4.37814600	L
H	0	-10.74407200	4.06188400	11.20154400	L
C	0	-10.26314900	5.01051800	11.51583300	L
H	0	-9.70595600	4.83221300	12.46121700	L
H	0	-11.05326600	5.77398900	11.68262800	L
O	0	-9.38825800	5.43584200	10.51245300	L
H	0	-8.96443900	6.26620100	10.85235200	L
H	0	-5.67655200	13.72867400	6.77967500	L
C	0	-6.36104900	13.80518800	5.90938300	L
H	0	-7.15593100	13.03512900	5.99187800	L
H	0	-5.78210600	13.62100300	4.98298400	L
O	0	-6.92764600	15.08185800	5.84916800	L
H	0	-7.44895400	15.18942600	6.68646600	L
H	0	-8.50618000	2.60189400	13.49546200	L
C	0	-8.26413700	1.71543200	12.87457200	L
H	0	-8.65332900	0.80611500	13.38135500	L
H	0	-7.16005900	1.64508500	12.77142300	L
O	0	-8.86008200	1.85934500	11.61779200	L
H	0	-8.61388200	1.04782000	11.10373500	L
H	0	-10.59744000	2.57362300	8.98741000	L
C	0	-11.08198500	2.90131600	8.04260200	L
H	0	-12.08162500	2.42447700	7.94984400	L
H	0	-10.45360200	2.57991800	7.18739100	L
O	0	-11.20407700	4.29365700	8.02226300	L
H	0	-11.81227900	4.52721200	8.77060300	L
H	0	-4.18499100	11.13178600	3.83405800	L
C	0	-4.11707600	10.99557600	4.93436200	L
H	0	-5.10101900	10.66785200	5.32865700	L

H	0	-3.84926900	11.96613300	5.39798300	L
O	0	-3.13499400	10.05251100	5.25092200	L
H	0	-3.36002800	9.23762700	4.73220100	L
H	0	-6.29684300	8.72516300	3.60453300	L
C	0	-6.73912500	8.09410700	4.40429100	L
H	0	-7.05841700	7.11715100	3.98126800	L
H	0	-7.62814200	8.61279300	4.81589000	L
O	0	-5.80819600	7.89563200	5.42770300	L
H	0	-5.03679800	7.43330200	5.00951800	L
H	0	-6.33372600	13.57386300	10.67628600	L
C	0	-5.43466900	13.69299400	10.03382300	L
H	0	-5.48709100	12.98248200	9.18226700	L
H	0	-4.53146300	13.46218800	10.63463700	L
O	0	-5.34853800	15.00659900	9.56227900	L
H	0	-6.16967600	15.15961800	9.02687900	L
H	0	-8.69033200	7.14892300	-0.41484300	L
C	0	-7.95036900	6.94421400	0.38854900	L
H	0	-7.01475200	6.53818800	-0.05145400	L
H	0	-8.37750500	6.18918700	1.07647100	L
O	0	-7.67648800	8.11175900	1.10585800	L
H	0	-7.37160300	8.77717200	0.43696400	L
H	0	-0.39239400	5.96450600	10.36544800	L
C	0	0.20424100	5.60982200	11.23050400	L
H	0	0.59601800	4.59558000	11.00202500	L
H	0	-0.45436800	5.56396900	12.12474500	L
O	0	1.26119000	6.49713500	11.45428500	L
H	0	1.76161100	6.13525400	12.23103800	L
H	0	-9.58718100	4.69331200	5.37621300	L
C	0	-9.10550200	4.22595800	4.49228800	L
H	0	-9.37514200	3.15283600	4.47287800	L
H	0	-8.00186500	4.31480200	4.57491200	L
O	0	-9.55643300	4.83318900	3.31700300	L
H	0	-9.26100100	5.77871100	3.36818100	L
H	0	-5.82910700	7.62625300	-3.85840100	L
C	0	-5.63896400	8.54588900	-3.26417900	L
H	0	-6.38785500	9.31403200	-3.54494100	L
H	0	-4.62521700	8.93776300	-3.49173700	L
O	0	-5.75159200	8.26942200	-1.89859800	L
H	0	-5.08511400	7.55993100	-1.70649500	L
H	0	-13.58230300	5.53517200	2.24351500	L
C	0	-14.47541300	6.18258900	2.38127400	L
H	0	-14.79654400	6.12619100	3.44093400	L
H	0	-15.30547500	5.82019900	1.73772300	L
O	0	-14.16657400	7.50963200	2.06712200	L

H	0	-13.84573500	7.50169200	1.12851200	L
H	0	-7.11994700	4.22278900	11.55970000	L
C	0	-6.02947300	4.32442900	11.39236500	L
H	0	-5.86218200	4.81184200	10.40833300	L
H	0	-5.57355300	3.31092300	11.39102600	L
O	0	-5.48137700	5.10268000	12.41594800	L
H	0	-4.50674700	5.14217600	12.23615700	L
H	0	-7.72752400	10.70465500	2.51513100	L
C	0	-7.40553100	11.60224500	3.08068300	L
H	0	-6.42641000	11.94463500	2.68366000	L
H	0	-7.29988500	11.32714200	4.15085300	L
O	0	-8.36581000	12.60762600	2.93157300	L
H	0	-8.01892200	13.39432700	3.42666200	L
H	0	-13.13802200	10.06399500	6.21349200	L
C	0	-13.56610100	10.72938200	5.43373200	L
H	0	-12.90061700	11.60606600	5.28183900	L
H	0	-14.55745500	11.08842400	5.77786100	L
O	0	-13.72265900	10.02915300	4.23378500	L
H	0	-12.81746100	9.70846500	3.98581900	L
H	0	-5.17814000	13.03770100	-4.19654200	L
C	0	-5.17630300	12.24562200	-4.97539800	L
H	0	-4.78386300	11.29848900	-4.54681800	L
H	0	-4.51293700	12.56246900	-5.80588300	L
O	0	-6.47049100	12.05565700	-5.46944300	L
H	0	-7.01046100	11.72808000	-4.70452000	L
H	0	-14.63138900	4.13128600	5.02038500	L
C	0	-13.66722600	3.78933200	5.44822800	L
H	0	-13.82882800	2.81382500	5.95558600	L
H	0	-13.31652300	4.54385500	6.18433700	L
O	0	-12.73119600	3.64982900	4.41962900	L
H	0	-11.89858600	3.32923900	4.85223700	L
H	0	-12.42487600	11.31791700	8.34843300	L
C	0	-13.05777600	11.31831100	9.26098200	L
H	0	-13.60270400	12.28401500	9.33784000	L
H	0	-12.40216700	11.20555100	10.14857000	L
O	0	-13.96292200	10.25310800	9.22194100	L
H	0	-14.53379800	10.40743700	8.42532500	L
H	0	-6.29013800	11.16719400	7.59253300	L
C	0	-6.34744700	10.14297100	8.01168000	L
H	0	-5.42452100	9.59077500	7.73465700	L
H	0	-7.23267900	9.63156400	7.57951700	L
O	0	-6.46320300	10.22114900	9.40286100	L
H	0	-6.57939900	9.28871900	9.72122900	L
H	0	-9.61439600	13.70530400	5.72149100	L

C	0	-10.66025200	14.07242500	5.72230100	L
H	0	-11.17978600	13.66956900	4.82601500	L
H	0	-10.64888800	15.18289000	5.68305700	L
O	0	-11.29808600	13.63874300	6.88867200	L
H	0	-12.23631200	13.95463600	6.82198900	L
H	0	-4.19419600	-0.42965200	6.89623100	L
C	0	-4.27222800	0.65719200	6.69850600	L
H	0	-5.33853500	0.91371100	6.52580700	L
H	0	-3.67689700	0.90012800	5.79249300	L
O	0	-3.78243800	1.35512600	7.80443400	L
H	0	-3.86868900	2.31631200	7.58034800	L
H	0	-10.09392200	7.36965500	5.18784400	L
C	0	-11.15004000	7.18551700	5.47939600	L
H	0	-11.62603900	8.13914100	5.78821200	L
H	0	-11.16867800	6.48659800	6.33895000	L
O	0	-11.85415100	6.61891200	4.41375600	L
H	0	-11.78885500	7.26863100	3.66764800	L
H	0	-11.90086400	0.93440600	5.73422800	L
C	0	-12.28155800	-0.10426200	5.79646200	L
H	0	-12.92100800	-0.19656000	6.70042400	L
H	0	-11.41522700	-0.79579000	5.87500700	L
O	0	-13.02666100	-0.38911000	4.64841700	L
H	0	-13.33166000	-1.32802000	4.74678000	L
H	0	-3.58940300	11.63657000	7.96556800	L
C	0	-2.77637100	11.14830500	8.53928000	L
H	0	-1.88506200	11.81156300	8.53236400	L
H	0	-2.52909400	10.18050500	8.05331500	L
O	0	-3.20635900	10.93322300	9.85201100	L
H	0	-2.45165900	10.48751100	10.31703400	L
H	0	-15.50046500	6.47888900	6.37342200	L
C	0	-14.80986900	7.32788000	6.55503800	L
H	0	-15.40782100	8.22919100	6.81160300	L
H	0	-14.22787100	7.51719000	5.62816200	L
O	0	-13.94962100	7.00258800	7.60817900	L
H	0	-13.36737000	7.79482500	7.73920800	L
H	0	-3.55437800	7.55313400	2.50938300	L
C	0	-4.14283400	6.69271600	2.12594100	L
H	0	-5.18502600	7.02489600	1.94708300	L
H	0	-4.15123200	5.88156600	2.88270000	L
O	0	-3.59903500	6.22881700	0.92478800	L
H	0	-2.67380900	5.93970500	1.13816800	L
H	0	-7.09685100	13.21693400	0.28804100	L
C	0	-7.38312000	12.44985600	-0.46066300	L
H	0	-8.43330200	12.62910900	-0.77557400	L

H	0	-7.29930500	11.44513600	0.00728500	L
O	0	-6.52912800	12.54596600	-1.56350800	L
H	0	-6.75823500	11.78341800	-2.15513500	L
H	0	-11.69485300	4.06579900	0.67253100	L
C	0	-11.13880300	4.73183600	-0.02238700	L
H	0	-11.47830500	4.52769600	-1.05781900	L
H	0	-10.04960400	4.52254200	0.04387800	L
O	0	-11.39090900	6.07140400	0.28755300	L
H	0	-11.10307900	6.19282900	1.22923600	L
H	0	-2.43278100	4.06183800	11.01620100	L
C	0	-2.69712800	3.47223500	10.11261100	L
H	0	-3.45291400	4.03375600	9.52740100	L
H	0	-3.13733400	2.49834000	10.41518800	L
O	0	-1.56403000	3.26582600	9.31994800	L
H	0	-0.93001300	2.74723000	9.87924800	L
H	0	-8.88137000	5.79644800	7.70661700	L
C	0	-8.00935400	6.42340000	7.43345800	L
H	0	-8.37195900	7.42842600	7.13461700	L
H	0	-7.48183100	5.94922800	6.57912100	L
O	0	-7.15440400	6.52556200	8.53429300	L
H	0	-6.39459300	7.08965900	8.23795400	L
H	0	-6.40633100	2.71781800	8.75838400	L
C	0	-7.48915000	2.72650700	8.50648500	L
H	0	-8.02276300	3.36145200	9.24149100	L
H	0	-7.90026200	1.69724500	8.56105800	L
O	0	-7.68548800	3.24861600	7.22657900	L
H	0	-7.12045500	2.70515800	6.62073200	L
H	0	-8.68569300	-0.63371100	4.88534600	L
C	0	-8.35809800	0.30125000	5.38743300	L
H	0	-7.86331400	0.03981200	6.34459100	L
H	0	-9.23925000	0.93893000	5.60890600	L
O	0	-7.45297800	0.99242800	4.57690800	L
H	0	-7.92474000	1.15992400	3.72138000	L
H	0	-9.36793700	11.14372000	9.02543000	L
C	0	-9.23876000	12.18117000	9.39463600	L
H	0	-8.68061300	12.15147300	10.35509000	L
H	0	-10.24192700	12.63125500	9.55588400	L
O	0	-8.52509500	12.91959900	8.44659200	L
H	0	-8.46531700	13.84204500	8.80672300	L
H	0	3.81475200	-12.30282100	-8.56078200	L
C	0	3.75319100	-11.19516600	-8.49910100	L
H	0	2.69628700	-10.88624300	-8.34897600	L
H	0	4.12014700	-10.76206300	-9.45192800	L
O	0	4.54744900	-10.72775100	-7.44821700	L

H	0	4.15193200	-11.10141400	-6.61955700	L
H	0	6.57874700	-4.62018500	-5.36409300	L
C	0	5.52795600	-4.28241000	-5.24705900	L
H	0	5.48223200	-3.19052600	-5.42320400	L
H	0	5.18308500	-4.49421600	-4.21304800	L
O	0	4.70652200	-4.92881500	-6.17302300	L
H	0	4.77784200	-5.89652400	-5.97028100	L
H	0	16.27258200	-1.39097900	-0.66815100	L
C	0	16.04145300	-2.46795100	-0.53658400	L
H	0	15.19893100	-2.73808600	-1.20768300	L
H	0	15.74786000	-2.64340400	0.52018600	L
O	0	17.17309300	-3.22454000	-0.85492800	L
H	0	16.91238900	-4.17333600	-0.72582600	L
H	0	9.70694100	-3.54509800	-6.01106700	L
C	0	9.06406600	-2.74520700	-5.59447600	L
H	0	9.70344300	-1.87385400	-5.33487500	L
H	0	8.56217300	-3.12861800	-4.68132800	L
O	0	8.11099400	-2.38019600	-6.55123600	L
H	0	7.55780700	-1.67185900	-6.13137400	L
H	0	13.53699700	-0.99531700	0.53777100	L
C	0	12.88261700	-0.39620200	-0.12594600	L
H	0	12.33471800	0.34972800	0.48817300	L
H	0	12.15702400	-1.07637300	-0.62103200	L
O	0	13.67374800	0.25078300	-1.07972700	L
H	0	13.04432900	0.72047000	-1.68495300	L
H	0	10.46427700	-7.71293900	1.34882300	L
C	0	10.07027500	-7.82806100	0.31953200	L
H	0	10.86324300	-7.53312700	-0.39976600	L
H	0	9.18911200	-7.16195300	0.19885000	L
O	0	9.70493700	-9.16223200	0.11681200	L
H	0	9.33772200	-9.20893200	-0.80325000	L
H	0	8.54269200	-4.92888100	-11.37063200	L
C	0	8.85720300	-5.76483300	-12.03153900	L
H	0	8.35961000	-5.66771400	-13.02052700	L
H	0	8.54694900	-6.72321600	-11.56807900	L
O	0	10.24663200	-5.76104800	-12.18834200	L
H	0	10.47332500	-4.88519200	-12.59591300	L
H	0	3.27652300	-14.73904200	-7.15364400	L
C	0	4.01478500	-14.65697100	-6.33008100	L
H	0	3.50962300	-14.90106300	-5.37069800	L
H	0	4.39750600	-13.61500100	-6.29614400	L
O	0	5.06606800	-15.54880700	-6.56302000	L
H	0	5.69808200	-15.42965200	-5.80736400	L
H	0	10.04694100	-8.40424700	5.29790000	L

C	0	10.43662900	-9.40549200	5.01799900	L
H	0	9.81114200	-10.19511300	5.48762000	L
H	0	11.47609900	-9.49980700	5.39383200	L
O	0	10.43586500	-9.55266600	3.62769200	L
H	0	9.49218300	-9.44392900	3.34228500	L
H	0	11.18672300	-8.93758000	-4.96315900	L
C	0	11.78015000	-9.01429800	-4.02805800	L
H	0	12.73435000	-8.45776500	-4.14164100	L
H	0	12.01189400	-10.08087100	-3.83520300	L
O	0	11.04673500	-8.50511100	-2.95352600	L
H	0	10.90783900	-7.54315700	-3.14865900	L
H	0	5.92425700	-4.65330000	-9.84970000	L
C	0	6.24228100	-5.58955500	-9.34611400	L
H	0	6.44185400	-6.35745400	-10.12003000	L
H	0	5.43134100	-5.95643900	-8.68222400	L
O	0	7.40893500	-5.36583800	-8.61083900	L
H	0	7.17329400	-4.69678800	-7.91812800	L
H	0	3.17961600	-11.62020700	0.36957400	L
C	0	4.28197600	-11.67467900	0.26460700	L
H	0	4.54343700	-12.61897700	-0.25891000	L
H	0	4.73745100	-11.67015500	1.27800500	L
O	0	4.72914100	-10.57188600	-0.47029500	L
H	0	5.70932300	-10.68834900	-0.57121200	L
H	0	6.98106900	-5.47094800	-2.38334000	L
C	0	6.47024500	-6.45777600	-2.36209100	L
H	0	6.14929400	-6.73812800	-3.38691800	L
H	0	5.57078300	-6.38088600	-1.72116900	L
O	0	7.32008500	-7.43610000	-1.83917300	L
H	0	8.11647300	-7.45198900	-2.43000600	L
H	0	10.46410900	-9.28424900	-7.69899000	L
C	0	11.47825900	-8.87242600	-7.88579700	L
H	0	12.22600700	-9.54509900	-7.41988900	L
H	0	11.56653100	-7.86786600	-7.42036200	L
O	0	11.72202700	-8.79350500	-9.26014600	L
H	0	11.05110700	-8.15891000	-9.62216100	L
H	0	13.74035800	-2.52645100	-8.08619700	L
C	0	13.52281400	-1.70689700	-7.36914700	L
H	0	14.24182900	-1.77505000	-6.52814100	L
H	0	12.49103000	-1.81898500	-6.97051000	L
O	0	13.66809200	-0.46571100	-7.99571800	L
H	0	13.01124300	-0.45718800	-8.73868600	L
H	0	9.59432700	-11.04847600	-3.03464100	L
C	0	8.52034200	-10.97859900	-3.31009500	L
H	0	7.92153400	-10.83280600	-2.38766300	L

H	0	8.19876500	-11.92229200	-3.80037500	L
O	0	8.31294800	-9.89328100	-4.16606000	L
H	0	8.81880100	-10.09575600	-4.99482400	L
H	0	10.82380800	0.79524900	-8.23624400	L
C	0	9.91336700	0.17134800	-8.10943100	L
H	0	10.21226500	-0.89623300	-8.07362600	L
H	0	9.23324100	0.32398200	-8.97333600	L
O	0	9.26081600	0.50739700	-6.91998000	L
H	0	9.03524300	1.47005300	-6.99465700	L
H	0	14.40772400	-12.58502200	-9.21322900	L
C	0	14.02101500	-11.63357100	-8.78872000	L
H	0	14.58441200	-11.40029500	-7.86220300	L
H	0	12.94519400	-11.74762900	-8.53440100	L
O	0	14.19475700	-10.59175800	-9.70481700	L
H	0	13.65205600	-10.83328700	-10.49966100	L
H	0	15.33949200	-5.44282100	-4.64234300	L
C	0	16.26694900	-4.83575500	-4.56871300	L
H	0	17.14990900	-5.47493100	-4.78499800	L
H	0	16.35404500	-4.44140200	-3.53541500	L
O	0	16.21634900	-3.76410900	-5.46539300	L
H	0	16.10850600	-4.16585400	-6.36609800	L
H	0	2.44472000	-12.23912600	-5.13743500	L
C	0	1.45594400	-12.20715400	-5.64199700	L
H	0	0.72689600	-12.81303900	-5.06550000	L
H	0	1.55478000	-12.64183400	-6.65742100	L
O	0	1.00980000	-10.88689600	-5.74865000	L
H	0	0.94171000	-10.54463100	-4.82019200	L
H	0	13.78925900	1.52912800	-4.42899200	L
C	0	12.79695400	1.94874500	-4.70002700	L
H	0	12.93363500	2.99225900	-5.05011800	L
H	0	12.13649800	1.95701600	-3.80736800	L
O	0	12.21664400	1.19361600	-5.72344100	L
H	0	12.08053400	0.28448800	-5.35032300	L
H	0	9.57186700	-5.25374100	-3.08528000	L
C	0	10.01809900	-4.78360800	-2.18563300	L
H	0	11.00945500	-5.24745300	-1.99228300	L
H	0	9.34429800	-4.96084700	-1.32010000	L
O	0	10.16430700	-3.41225300	-2.41241700	L
H	0	10.56694500	-3.03864400	-1.58711300	L
H	0	8.57542900	-2.49460400	2.04940200	L
C	0	9.42412300	-2.56324700	1.33651300	L
H	0	9.27659400	-3.43445300	0.66491500	L
H	0	10.35967500	-2.70745000	1.91155100	L
O	0	9.52612900	-1.38738500	0.58886900	L

H	0	8.68680900	-1.32564300	0.06494700	L
H	0	5.84134700	-12.55789000	-2.55308300	L
C	0	5.46464900	-13.37110500	-3.20564600	L
H	0	6.25597100	-13.63949300	-3.93726900	L
H	0	4.56410900	-13.00734100	-3.74429600	L
O	0	5.14262600	-14.48070600	-2.41830800	L
H	0	4.78663800	-15.16479500	-3.04251000	L
H	0	12.39380100	-2.21109200	-4.27300900	L
C	0	13.30107700	-2.30665300	-3.64266100	L
H	0	13.85234200	-3.21972900	-3.94839500	L
H	0	12.99355100	-2.39680900	-2.57967800	L
O	0	14.10340700	-1.17592300	-3.81924000	L
H	0	14.92496200	-1.33542000	-3.28687700	L
H	0	14.20796600	-9.62890900	1.48050400	L
C	0	13.28438700	-9.37700400	0.91999400	L
H	0	13.21169600	-8.27215200	0.83261000	L
H	0	12.40761400	-9.76717200	1.48021600	L
O	0	13.34415800	-9.95903600	-0.35013600	L
H	0	12.50107500	-9.70514300	-0.80738400	L
H	0	6.58841300	-8.46887700	0.70406200	L
C	0	6.75282700	-8.56153300	1.79913800	L
H	0	5.92409500	-8.05267800	2.32925500	L
H	0	7.70506200	-8.06655200	2.08210500	L
O	0	6.78042200	-9.90830400	2.17301300	L
H	0	7.51821300	-10.32113900	1.65377700	L
H	0	11.39710900	-1.32845300	-10.60527600	L
C	0	11.96387900	-1.65130700	-11.50523700	L
H	0	12.04502100	-0.79461500	-12.20462400	L
H	0	11.42113300	-2.47559700	-12.01492500	L
O	0	13.24723400	-2.07094600	-11.14299300	L
H	0	13.11989800	-2.85553100	-10.54930700	L
H	0	2.57555400	-8.63822300	-6.01317900	L
C	0	2.35135900	-7.91251700	-6.82353300	L
H	0	2.30033800	-6.88513200	-6.40518000	L
H	0	1.36707400	-8.16515900	-7.26624700	L
O	0	3.33280100	-7.97601900	-7.81699500	L
H	0	4.19979800	-7.83764100	-7.35584700	L
H	0	14.29433200	-6.87343300	-10.01845800	L
C	0	13.73011400	-6.00061000	-9.62472400	L
H	0	13.08357100	-6.32448800	-8.78238300	L
H	0	13.08669400	-5.59443900	-10.43211400	L
O	0	14.61557900	-5.00672600	-9.19765600	L
H	0	15.14328700	-5.40946700	-8.46028800	L
H	0	7.67668900	-9.57408800	-7.68983600	L

C	0	7.26721700	-9.34776400	-8.69780300	L
H	0	6.37171200	-8.69785200	-8.60204400	L
H	0	6.96569400	-10.29820200	-9.18329500	L
O	0	8.23680500	-8.71851400	-9.48374200	L
H	0	8.48543500	-7.88917000	-8.99985100	L
H	0	-1.15007900	-8.39266000	-8.00444900	L
C	0	-2.03932300	-8.16686600	-8.63136300	L
H	0	-2.94506300	-8.53970200	-8.11254700	L
H	0	-1.95107000	-8.68416400	-9.61051100	L
O	0	-2.15652900	-6.78805400	-8.82514800	L
H	0	-1.31337900	-6.50086800	-9.26225000	L
H	0	5.59068200	-10.56747000	-4.43745300	L
C	0	5.00935500	-9.68601400	-4.09544900	L
H	0	5.03603500	-8.91175200	-4.88866800	L
H	0	5.46640000	-9.27158700	-3.17120900	L
O	0	3.68190200	-10.05162500	-3.85772000	L
H	0	3.70739800	-10.69593100	-3.10495200	L
H	0	14.89224600	-7.40905000	-1.10332900	L
C	0	14.23587400	-6.85026300	-1.80134600	L
H	0	13.17844200	-7.12266500	-1.59424900	L
H	0	14.49984000	-7.13208400	-2.84287800	L
O	0	14.42923100	-5.47943500	-1.61179700	L
H	0	13.76780100	-5.02706000	-2.19629700	L
H	0	3.21332600	-7.68175400	-2.53843800	L
C	0	2.91629500	-6.84094800	-3.19873100	L
H	0	3.47945500	-6.90108600	-4.15222500	L
H	0	3.16997200	-5.88455200	-2.69708000	L
O	0	1.54146300	-6.88179900	-3.44778100	L
H	0	1.36261000	-7.77805400	-3.83385000	L
H	0	6.38901200	-1.20873600	-1.42141100	L
C	0	6.50979600	-2.05989000	-2.12452400	L
H	0	5.70041300	-2.79348700	-1.93803200	L
H	0	7.48803400	-2.55750800	-1.95359400	L
O	0	6.42083500	-1.60572100	-3.44280600	L
H	0	7.16433600	-0.96017400	-3.56089500	L
H	0	6.61458900	-12.14733000	-6.09237800	L
C	0	7.39055600	-12.63063200	-6.71874300	L
H	0	7.75724300	-13.53995600	-6.19544900	L
H	0	8.22808600	-11.91573900	-6.86075600	L
O	0	6.83475200	-12.97481300	-7.95467200	L
H	0	7.56319900	-13.40816800	-8.47030500	L
H	0	7.56520300	-2.54031700	-9.43733100	L
C	0	7.71295100	-2.05030600	-10.41976700	L
H	0	7.21271300	-2.65837800	-11.20291200	L

H	0	8.80350200	-1.99128100	-10.62633700	L
O	0	7.15443700	-0.76995500	-10.38019800	L
H	0	7.39790500	-0.33911100	-11.23985400	L
H	0	6.27308600	-5.87537700	0.76539800	L
C	0	6.25430400	-4.77174300	0.87528900	L
H	0	6.95219500	-4.31368100	0.14157300	L
H	0	6.58811600	-4.50801100	1.89937700	L
O	0	4.95561100	-4.29105400	0.67885200	L
H	0	4.74018800	-4.46017200	-0.27439500	L
H	0	9.12048700	-7.52816900	-6.51665800	L
C	0	8.54255400	-6.98645500	-5.74023300	L
H	0	8.58844500	-5.89349300	-5.93154200	L
H	0	8.99591700	-7.19188300	-4.75170700	L
O	0	7.21541800	-7.42529800	-5.73594400	L
H	0	6.84651900	-7.18926400	-6.62546000	L
H	0	1.67444300	-4.29727300	-9.51677600	L
C	0	2.12841000	-5.24775100	-9.16838400	L
H	0	1.31726400	-5.91655300	-8.81807700	L
H	0	2.82053100	-5.04410500	-8.32336900	L
O	0	2.81435800	-5.87061300	-10.21455400	L
H	0	3.49959900	-5.22088100	-10.51597400	L
H	0	15.69606300	-7.30825900	-6.61508700	L
C	0	14.86985500	-8.04811300	-6.62899800	L
H	0	14.75312100	-8.43619800	-7.66393400	L
H	0	13.93357400	-7.54345500	-6.31128700	L
O	0	15.17409700	-9.09415200	-5.75255400	L
H	0	14.40417200	-9.71865500	-5.79486800	L
H	0	11.53919200	-3.78614300	-8.54254000	L
C	0	10.58091500	-4.10405400	-9.00388800	L
H	0	10.62057400	-3.89270900	-10.08982500	L
H	0	9.74164500	-3.52895500	-8.56036200	L
O	0	10.38375700	-5.47429200	-8.81282600	L
H	0	10.33523900	-5.60777200	-7.83160700	L
H	0	10.90957700	-11.39903300	-10.21182500	L
C	0	10.02460700	-11.90955000	-9.77402400	L
H	0	9.66194600	-11.34169000	-8.89149800	L
H	0	10.32488700	-12.92518900	-9.44445600	L
O	0	9.00789900	-12.01827900	-10.72763600	L
H	0	8.79715600	-11.09090900	-11.01024600	L
H	0	1.78976400	-7.62391100	1.00287100	H
C	0	2.16059300	-6.73477600	0.47351500	H
H	0	2.05455500	-5.87094000	1.14905700	H
H	0	1.49419000	-6.56681200	-0.37394100	H
O	0	3.46446500	-6.89527100	-0.03099100	H

H	0	4.03573000	-7.12640700	0.70379100	H
H	0	4.11835400	-7.66677800	-12.11728300	L
C	0	4.75258400	-8.44820100	-11.65126800	L
H	0	4.30806400	-9.44401400	-11.86669800	L
H	0	4.77777100	-8.27908600	-10.55340300	L
O	0	6.04373900	-8.36968800	-12.18222300	L
H	0	6.56017300	-9.10080900	-11.75432800	L
H	0	2.46487300	-2.64815600	-5.37463900	H
C	0	2.31676600	-3.18704400	-4.43787000	H
H	0	2.45234400	-4.25755300	-4.64415500	H
H	0	1.28293100	-3.01530900	-4.10700500	H
O	0	3.27007100	-2.68254500	-3.52211600	H
H	0	3.26103400	-3.25441700	-2.74975900	H
H	0	-0.38920000	-5.94803800	-6.71576500	L
C	0	-0.29796800	-4.86093800	-6.50793700	L
H	0	0.77235700	-4.57890500	-6.55902400	L
H	0	-0.67666200	-4.63854400	-5.48858000	L
O	0	-1.00981300	-4.12796800	-7.46325600	L
H	0	-1.96286200	-4.37465300	-7.34399600	L
H	0	9.33238100	-0.62995800	-2.25731400	L
C	0	10.09237300	0.03219800	-2.72126200	L
H	0	10.97378700	-0.57721500	-3.00078300	L
H	0	10.41288200	0.80629300	-1.99177100	L
O	0	9.56944400	0.63406200	-3.86892400	L
H	0	8.80937000	1.19155500	-3.56185200	L
H	0	10.29527700	-12.89295200	-5.30109100	L
C	0	11.05507400	-12.38256300	-5.92730800	L
H	0	10.74522600	-11.32600500	-6.07362800	L
H	0	11.11218200	-12.89549000	-6.91114900	L
O	0	12.29424400	-12.44044000	-5.28251700	L
H	0	12.92225900	-11.92368000	-5.85037000	L
H	0	0.80086000	-9.56206300	-9.43435700	L
C	0	1.11325300	-8.93015300	-10.29188800	L
H	0	2.21424300	-8.81387300	-10.26374800	L
H	0	0.64586700	-7.92685700	-10.20654300	L
O	0	0.74485600	-9.53424800	-11.49764000	L
H	0	-0.24367200	-9.61666800	-11.47068900	L
H	0	5.67835400	-5.53047100	-12.59242200	L
C	0	5.27639400	-4.56795100	-12.97417000	L
H	0	6.05406700	-4.07574600	-13.59317800	L
H	0	5.02666400	-3.90345800	-12.12097700	L
O	0	4.14015900	-4.79466900	-13.75691200	L
H	0	3.47534300	-5.22333700	-13.15778100	L
H	0	11.33894000	-6.23172000	-5.41055500	L

C	0	12.08770500	-5.44890900	-5.18172800	L
H	0	12.82425300	-5.86098300	-4.46035600	L
H	0	11.56920500	-4.57805400	-4.73068000	L
O	0	12.72693300	-5.06840200	-6.36464000	L
H	0	13.40716700	-4.39579400	-6.10535700	L
H	0	-2.68293700	2.85180300	-13.65695800	L
C	0	-3.09587500	1.82178000	-13.62182800	L
H	0	-3.08705700	1.45205700	-12.57444300	L
H	0	-2.45618600	1.15929600	-14.23953100	L
O	0	-4.39907500	1.80842700	-14.12798300	L
H	0	-4.92644600	2.40877600	-13.53953300	L
H	0	5.44455000	3.95517100	-3.60787100	L
C	0	5.45182000	3.10670600	-2.89556000	L
H	0	4.41257500	2.73217700	-2.77693500	L
H	0	5.83911700	3.46422200	-1.91859000	L
O	0	6.27570100	2.09665900	-3.39713300	L
H	0	6.20190500	1.34199100	-2.75820200	L
H	0	-0.74201200	3.54138700	-7.00010400	L
C	0	-0.22063000	2.83705600	-7.68290700	L
H	0	0.54134200	3.39801300	-8.25903500	L
H	0	-0.94899000	2.38897000	-8.39226300	L
O	0	0.41088100	1.83399300	-6.94421400	L
H	0	-0.31073500	1.36861500	-6.44771600	L
H	0	8.04789600	11.22849600	-7.78843400	L
C	0	8.05618500	10.33890200	-7.12636300	L
H	0	7.27576800	9.62970800	-7.47308300	L
H	0	7.83147900	10.66098500	-6.08784000	L
O	0	9.31613500	9.73585000	-7.18368500	L
H	0	9.27244000	8.95401100	-6.57469400	L
H	0	3.90916400	13.69386500	-6.42235700	L
C	0	3.05299000	14.11701300	-6.98540100	L
H	0	2.48663600	14.79981900	-6.31612600	L
H	0	2.39590600	13.28386900	-7.31544200	L
O	0	3.53552000	14.81911000	-8.09389300	L
H	0	2.73502900	15.16246000	-8.56920000	L
H	0	7.67183200	0.69536800	-15.03353400	L
C	0	7.44471100	1.74216100	-14.74666200	L
H	0	6.37019000	1.93979000	-14.94897600	L
H	0	8.07487200	2.42544500	-15.35585500	L
O	0	7.72006800	1.91694500	-13.38724300	L
H	0	7.48500800	2.85827400	-13.18018800	L
H	0	0.98558300	7.75830300	-5.74391800	L
C	0	1.56960800	6.90701600	-6.14813000	L
H	0	2.57551100	6.90730900	-5.67680100	L

H	0	1.66953100	7.03150300	-7.24665400	L
O	0	0.90197800	5.71382600	-5.86054300	L
H	0	1.45275700	4.99088800	-6.25573300	L
H	0	-1.10769300	-1.00294200	-13.12214000	L
C	0	-1.82926300	-1.82932200	-12.96041300	L
H	0	-2.24164700	-1.74188100	-11.93326500	L
H	0	-1.29846400	-2.79935800	-13.06831900	L
O	0	-2.85586700	-1.73730200	-13.90503400	L
H	0	-3.48832900	-2.47213000	-13.69305800	L
H	0	0.11520200	3.79755700	-3.21570600	L
C	0	0.04045800	2.71576100	-3.45204000	L
H	0	-0.68105800	2.56839600	-4.28151500	L
H	0	-0.32864300	2.17917200	-2.55365500	L
O	0	1.29419100	2.20863800	-3.80676800	L
H	0	1.59596700	2.74653700	-4.58445100	L
H	0	2.45454500	4.93546300	-11.37049900	L
C	0	3.10169100	5.39653400	-10.59575800	L
H	0	3.08009600	6.50218400	-10.70003000	L
H	0	4.14172100	5.04420800	-10.74664300	L
O	0	2.66933700	5.02273300	-9.32011700	L
H	0	1.75128700	5.38561300	-9.22507100	L
H	0	8.09891900	5.17989300	-9.20233400	L
C	0	8.61133400	5.25643600	-8.22415100	L
H	0	9.28468400	4.38017800	-8.10602200	L
H	0	7.84217000	5.25529400	-7.42259300	L
O	0	9.34896300	6.44326500	-8.18496600	L
H	0	9.77707600	6.46881700	-7.29034900	L
H	0	-3.12049400	3.39808900	-4.57154500	L
C	0	-3.54233600	3.30156400	-5.59129200	L
H	0	-3.24722600	4.19390000	-6.18377000	L
H	0	-4.64918700	3.24647800	-5.51451200	L
O	0	-3.04176000	2.13831400	-6.18338000	L
H	0	-3.40080700	2.12474400	-7.10728700	L
H	0	-3.05050000	6.54889200	-8.68610900	L
C	0	-2.36484000	7.04166700	-7.96548400	L
H	0	-2.64404900	6.76228100	-6.92827500	L
H	0	-2.46312300	8.13906900	-8.08391700	L
O	0	-1.04220900	6.67006200	-8.21669600	L
H	0	-1.02676100	5.67924700	-8.19538300	L
H	0	2.95407300	5.50482800	-14.19545000	L
C	0	3.36522100	6.51351000	-13.97906200	L
H	0	4.38449000	6.39896500	-13.55795900	L
H	0	2.72562500	7.02612600	-13.23134000	L
O	0	3.43211300	7.27062100	-15.15231900	L

H	0	2.50228900	7.32619700	-15.49453000	L
H	0	2.34470300	1.62608800	-8.96024000	L
C	0	3.35404000	1.50623900	-9.40074600	L
H	0	4.08357400	1.34690900	-8.57956100	L
H	0	3.61276900	2.43035300	-9.95920100	L
O	0	3.34993200	0.40750800	-10.26414800	L
H	0	4.27342300	0.33536200	-10.61875400	L
H	0	-0.66482100	1.52711700	-11.95725300	L
C	0	0.28272400	1.41993400	-11.38975100	L
H	0	0.93977600	0.71213200	-11.93227900	L
H	0	0.07579300	1.01080000	-10.37795000	L
O	0	0.92711100	2.65568100	-11.28735800	L
H	0	0.29825300	3.25707900	-10.81179500	L
H	0	3.93452700	-2.22648000	-7.37880800	L
C	0	4.63243100	-2.15299100	-8.23624300	L
H	0	5.48202400	-2.84568400	-8.06075700	L
H	0	5.01077900	-1.11138700	-8.30914600	L
O	0	3.95666200	-2.50487000	-9.40661300	L
H	0	4.61289200	-2.40001100	-10.14252000	L
H	0	2.41729900	-3.17545200	-11.73066900	L
C	0	1.81318600	-2.29527900	-12.03599300	L
H	0	1.25965700	-2.52878800	-12.97032300	L
H	0	2.49343000	-1.44295900	-12.23047900	L
O	0	0.92298100	-1.94288400	-11.01748300	L
H	0	0.31387400	-2.71714000	-10.90450200	L
H	0	1.12781900	-2.36199400	-8.00888100	L
C	0	0.94015200	-1.29648600	-7.76090800	L
H	0	0.19716000	-0.86809400	-8.46629700	L
H	0	1.88863500	-0.73476800	-7.86788400	L
O	0	0.48396800	-1.18212100	-6.44404900	L
H	0	-0.38938400	-1.65093900	-6.41352300	L
H	0	6.24218300	4.07001000	-16.83385600	L
C	0	5.79594100	4.75593900	-16.08166400	L
H	0	4.93038400	4.25017100	-15.60972100	L
H	0	5.44067300	5.68298200	-16.58093200	L
O	0	6.73521500	5.06905700	-15.09503500	L
H	0	7.49563900	5.49739900	-15.56691500	L
H	0	7.31949600	8.79803500	-11.22938200	L
C	0	8.08069600	9.46395800	-10.77054800	L
H	0	8.63314000	8.91214800	-9.97975800	L
H	0	7.56390200	10.33018200	-10.30967700	L
O	0	8.96976900	9.92438200	-11.74635800	L
H	0	9.38088500	9.11411700	-12.14544700	L
H	0	-6.24602100	3.24575500	-11.21097500	L

C	0	-6.66063200	4.27616200	-11.16484900	L
H	0	-5.84683000	4.99960200	-10.95080300	L
H	0	-7.40412600	4.32578100	-10.34404400	L
O	0	-7.28568100	4.60197000	-12.37186500	L
H	0	-6.59625000	4.48862300	-13.07594600	L
H	0	0.98386200	-6.46935900	-12.39514600	L
C	0	0.14413400	-5.74151400	-12.38074500	L
H	0	0.32456300	-4.97474500	-13.16138000	L
H	0	0.09825800	-5.23971600	-11.39075500	L
O	0	-1.06377300	-6.39347900	-12.64746100	L
H	0	-1.19213000	-7.04459600	-11.90973400	L
H	0	0.86690000	10.38949300	-9.25472800	L
C	0	0.92878700	9.88079300	-8.27139700	L
H	0	1.86808100	9.28996800	-8.23492900	L
H	0	0.05443400	9.20487400	-8.16171400	L
O	0	0.92595000	10.84062500	-7.25603100	L
H	0	0.96401000	10.33569300	-6.40310800	L
H	0	-0.75654100	12.51173200	-4.57033000	L
C	0	-0.01452600	13.25898900	-4.92224100	L
H	0	0.95690900	12.75721600	-5.11781300	L
H	0	0.12988600	14.01845400	-4.12855700	L
O	0	-0.47616100	13.89070900	-6.08100600	L
H	0	-0.57812100	13.17550400	-6.76089300	L
H	0	-2.31966900	-1.69843100	-8.64957400	L
C	0	-2.75023200	-0.67718000	-8.61860800	L
H	0	-2.28672200	-0.12261900	-7.77491200	L
H	0	-3.84698600	-0.75748300	-8.46084100	L
O	0	-2.48179100	-0.02863100	-9.82727100	L
H	0	-2.87158600	0.87932300	-9.74284300	L
H	0	3.72201700	11.19382300	-8.35542800	L
C	0	4.80191700	11.35709200	-8.54550300	L
H	0	5.26148800	10.39187000	-8.84644100	L
H	0	5.27665900	11.72548600	-7.61109900	L
O	0	4.95158000	12.29998300	-9.56668000	L
H	0	5.92929800	12.40569100	-9.69912300	L
H	0	6.84462700	7.42523800	-6.25486300	L
C	0	7.07536100	7.33740800	-5.17576800	L
H	0	7.04335400	8.34976000	-4.71943600	L
H	0	8.09260700	6.90476200	-5.06618900	L
O	0	6.12749100	6.50697100	-4.57248100	L
H	0	6.38171000	6.45317300	-3.61535200	L
H	0	-1.51033100	5.38610000	-4.65984700	L
C	0	-1.70448700	6.22022200	-3.95469900	L
H	0	-1.01228200	7.05290000	-4.18638200	L

H	0	-1.51818700	5.88464200	-2.91281300	L
O	0	-3.02114000	6.66870100	-4.09052000	L
H	0	-3.60007900	5.90029200	-3.85006100	L
H	0	6.27288400	1.94148700	-10.83674700	L
C	0	6.72338400	2.61977000	-10.08527200	L
H	0	6.10109300	3.53719300	-10.01481600	L
H	0	7.74783700	2.88786700	-10.41632300	L
O	0	6.77214700	1.96775200	-8.84959400	L
H	0	7.14085800	2.62665800	-8.20646500	L
H	0	4.93441300	4.00791700	-7.83920500	L
C	0	4.73122100	4.24319700	-6.77287900	L
H	0	4.30805900	5.26628500	-6.68843700	L
H	0	5.68679700	4.20156100	-6.21275100	L
O	0	3.84068800	3.31358600	-6.23011200	L
H	0	3.06639300	3.27971700	-6.84909800	L
H	0	-3.58034200	5.65514300	-13.07701500	L
C	0	-4.06670100	5.89898300	-14.04443100	L
H	0	-4.99229700	6.48589600	-13.86119600	L
H	0	-4.33917100	4.95280100	-14.55479500	L
O	0	-3.18880200	6.62433200	-14.85556200	L
H	0	-2.96926100	7.44966400	-14.35046100	L
H	0	7.85307100	8.38683100	-14.38835400	L
C	0	6.74661700	8.49170800	-14.38890000	L
H	0	6.32586700	8.02294400	-13.47409900	L
H	0	6.33794300	7.96907700	-15.27754800	L
O	0	6.38930900	9.84222800	-14.44757600	L
H	0	6.77361000	10.26252500	-13.63506000	L
H	0	0.70539600	4.76678000	-13.14842400	L
C	0	-0.20008200	4.66611600	-13.78222800	L
H	0	-1.10553000	4.89260100	-13.18008300	L
H	0	-0.26536000	3.62089000	-14.14553300	L
O	0	-0.11895400	5.53041900	-14.87812600	L
H	0	-0.04625000	6.44471800	-14.50056500	L
H	0	-2.14796600	3.15900200	-10.65157600	L
C	0	-2.92966200	3.94466900	-10.60508600	L
H	0	-3.63474500	3.79306000	-11.44566700	L
H	0	-2.46180900	4.94735700	-10.70342700	L
O	0	-3.63089900	3.85374000	-9.40066600	L
H	0	-2.96305900	4.01229600	-8.68529400	L
H	0	6.70315700	7.60220500	-8.81274900	L
C	0	5.67609000	7.24815700	-9.02884900	L
H	0	5.66956100	6.13857200	-8.99664600	L
H	0	5.38484500	7.59585900	-10.04259200	L
O	0	4.80040800	7.76365600	-8.06952300	L

H	0	3.89909200	7.43199000	-8.31584800	L
H	0	-4.24601500	6.92860300	-10.91563900	L
C	0	-4.56914600	7.98531600	-11.02726100	L
H	0	-4.17448000	8.40119100	-11.97931300	L
H	0	-4.15582900	8.57306500	-10.18381800	L
O	0	-5.96418900	8.06930900	-11.00563400	L
H	0	-6.28335900	7.52171900	-11.76899600	L
H	0	-3.25459500	11.17215000	-9.70309100	L
C	0	-2.30446700	10.90592100	-10.21429000	L
H	0	-2.52345100	10.67890500	-11.27775900	L
H	0	-1.86366700	10.00292000	-9.74103200	L
O	0	-1.40460500	11.97406000	-10.14730500	L
H	0	-1.24305800	12.13517100	-9.18155500	L
H	0	1.86614000	0.77911500	-15.60313000	L
C	0	0.95054600	0.38435800	-15.11789800	L
H	0	0.41454300	1.22397900	-14.62512400	L
H	0	1.24690700	-0.36784600	-14.35700300	L
O	0	0.13776300	-0.20796700	-16.08917500	L
H	0	-0.68166700	-0.50623800	-15.61618600	L
H	0	-3.19258500	9.07253700	-5.77197000	L
C	0	-2.27395000	9.63345600	-5.50046800	L
H	0	-1.37589200	9.02578900	-5.74404900	L
H	0	-2.29106900	9.82703600	-4.40973100	L
O	0	-2.23341600	10.85145800	-6.18448400	L
H	0	-2.15780800	10.62243500	-7.14642400	L
H	0	-0.10030200	6.03306000	-10.87948000	L
C	0	-0.00032200	7.10657200	-11.14803000	L
H	0	0.56324100	7.20224400	-12.09895500	L
H	0	0.56326100	7.62614600	-10.34674000	L
O	0	-1.26346000	7.69045200	-11.27510900	L
H	0	-1.71380200	7.20659100	-12.01464100	L
H	0	8.76134300	4.80078900	-11.87484600	L
C	0	8.37469100	5.82935900	-12.03926600	L
H	0	8.52675300	6.12307300	-13.09960400	L
H	0	8.94011300	6.52848600	-11.39013800	L
O	0	7.01742900	5.89480700	-11.71050600	L
H	0	6.56347200	5.22693900	-12.28576000	L
H	0	-0.79565800	8.88933700	-13.98482500	L
C	0	0.04192700	9.61480100	-13.94267800	L
H	0	-0.03495600	10.30010200	-14.81435300	L
H	0	1.00190400	9.05664300	-13.98752100	L
O	0	-0.03986800	10.33914700	-12.74946900	L
H	0	0.71494100	10.98287800	-12.76743600	L
H	0	8.48615000	-2.93199800	-13.41911900	L

C	0	9.07888500	-2.21801000	-14.02597700	L
H	0	8.53879100	-2.02243100	-14.97731100	L
H	0	10.07007000	-2.67173400	-14.24316200	L
O	0	9.23677100	-1.02762400	-13.30970400	L
H	0	9.80198300	-0.43992900	-13.87506100	L
H	0	2.60759500	4.77220500	-16.66002300	L
C	0	2.15670500	3.77128400	-16.83171400	L
H	0	1.63339700	3.43240600	-15.91282600	L
H	0	2.96553000	3.04945700	-17.06683400	L
O	0	1.26147400	3.82462900	-17.90421400	L
H	0	0.57134300	4.49103700	-17.65039600	L
H	0	9.88480700	5.01289000	-5.12368700	L
C	0	9.70885600	3.91837700	-5.04489600	L
H	0	10.03712900	3.55687700	-4.04792400	L
H	0	10.30548900	3.40568600	-5.82615700	L
O	0	8.35529800	3.62738100	-5.23743600	L
H	0	7.86315700	4.16383500	-4.56418100	L
H	0	3.42990200	0.31277300	-6.26303500	L
C	0	4.28716000	0.25129600	-5.56348000	L
H	0	4.37579900	-0.79402500	-5.19819000	L
H	0	4.09373100	0.93449200	-4.71050800	L
O	0	5.45500300	0.63220300	-6.23203300	L
H	0	6.19754400	0.49886000	-5.58829700	L
H	0	-5.42038300	8.54457900	-7.98987000	L
C	0	-5.52759200	9.56658200	-7.56991400	L
H	0	-4.60576900	10.15234000	-7.77001400	L
H	0	-5.66952500	9.48717800	-6.47296400	L
O	0	-6.63558000	10.20684700	-8.13287400	L
H	0	-6.46334900	10.24279300	-9.10939300	L
H	0	-3.57867400	3.36123800	-16.97681400	L
C	0	-2.48739600	3.51482200	-16.85042500	L
H	0	-2.31407700	4.07243000	-15.90601800	L
H	0	-2.10466400	4.10637600	-17.70961900	L
O	0	-1.85132400	2.27086000	-16.80216700	L
H	0	-0.89418100	2.46073600	-16.62393400	L
H	0	10.21633900	2.58550300	-10.32180400	L
C	0	10.53580000	2.05605200	-11.24562800	L
H	0	10.22840100	2.65597800	-12.12615600	L
H	0	10.04137200	1.06223000	-11.30147700	L
O	0	11.92580200	1.90605400	-11.25662300	L
H	0	12.14977000	1.37495100	-10.44902200	L
H	0	3.11329100	1.40779200	-13.00839100	L
C	0	3.44707300	2.36222000	-13.46250800	L
H	0	2.58035200	3.05394600	-13.51727000	L

H	0	3.82813900	2.15626700	-14.48555500	L
O	0	4.45823300	2.91542700	-12.67270200	L
H	0	4.72003700	3.76184200	-13.11763800	L
H	0	6.25680300	-1.30985100	-13.73524200	L
C	0	5.31366100	-0.87219300	-13.34631700	L
H	0	5.50122000	0.17900700	-13.05034000	L
H	0	4.98188900	-1.43575100	-12.44948000	L
O	0	4.32518500	-0.90362100	-14.33463300	L
H	0	4.16455400	-1.86304100	-14.53021300	L
H	0	2.31377700	9.05247700	-11.72712400	L
C	0	3.15315500	9.58182100	-11.23589000	L
H	0	3.33596900	9.11451000	-10.24593600	L
H	0	2.87461300	10.64937900	-11.10196200	L
O	0	4.29347000	9.47597900	-12.03679300	L
H	0	5.00974200	9.96736900	-11.55745200	L
H	0	6.65977300	-6.28585700	8.23623700	L
C	0	5.64301200	-6.05550600	8.61818300	L
H	0	5.32311200	-6.83898000	9.33688900	L
H	0	4.94083600	-6.04727500	7.76126600	L
O	0	5.62819800	-4.80322300	9.23814600	L
H	0	6.33819600	-4.83303600	9.92994500	L
H	0	4.06659800	5.38277000	9.66223700	L
C	0	3.83832800	4.46313800	10.24271900	L
H	0	2.83842400	4.09199300	9.94220700	L
H	0	3.82113900	4.69937700	11.32833100	L
O	0	4.79139000	3.47516900	9.98006000	L
H	0	5.66460200	3.86119000	10.24792900	L
H	0	13.79840100	-2.36241500	2.79165100	L
C	0	13.16157400	-1.86424700	3.54981000	L
H	0	13.66631800	-1.92459500	4.53783500	L
H	0	12.18332600	-2.38726300	3.59388000	L
O	0	12.97678200	-0.52850200	3.18452000	L
H	0	12.44487700	-0.11734900	3.91259500	L
H	0	13.02354500	-5.42481300	4.33737000	L
C	0	12.86422800	-6.44651400	4.74205300	L
H	0	11.86002900	-6.50862500	5.21253300	L
H	0	13.63353200	-6.64955600	5.51440600	L
O	0	12.97966300	-7.39243700	3.71954800	L
H	0	12.25519400	-7.18566700	3.07452500	L
H	0	8.28841700	4.57994600	9.98002500	L
C	0	8.74984000	4.24437500	10.93043100	L
H	0	9.69756700	4.80370100	11.08628400	L
H	0	8.96298900	3.15678600	10.85579000	L
O	0	7.86446400	4.49300500	11.98361600	L

H	0	8.31215400	4.15499000	12.80207500	L
H	0	15.67098800	-6.14669900	3.00091000	L
C	0	15.63275500	-5.29323300	2.29003000	L
H	0	14.81300000	-4.60291500	2.58028000	L
H	0	15.42704800	-5.68272700	1.27193100	L
O	0	16.85571700	-4.61546400	2.28457400	L
H	0	16.97144400	-4.25116400	3.20027100	L
H	0	10.81225700	3.25199200	4.28636600	L
C	0	11.76535600	3.10481200	3.73464500	L
H	0	12.43808600	2.43853000	4.31542300	L
H	0	12.25964200	4.08832300	3.61010800	L
O	0	11.51399300	2.55712500	2.47390300	L
H	0	11.05606700	1.69279800	2.63738600	L
H	0	8.73518200	-8.93954400	8.83352900	L
C	0	8.10934100	-8.75460800	9.73025100	L
H	0	8.01858900	-7.65847900	9.88354700	L
H	0	8.60280900	-9.21527800	10.61314700	L
O	0	6.84693200	-9.32218900	9.53250000	L
H	0	6.32480900	-9.12139000	10.35198400	L
H	0	12.59331500	3.41051900	-1.76872600	L
C	0	11.72441800	3.48548900	-1.08390300	L
H	0	11.70741900	2.59116500	-0.42647700	L
H	0	10.79251500	3.51904300	-1.68632200	L
O	0	11.84677800	4.64613800	-0.31503100	L
H	0	11.05404400	4.66252500	0.28024900	L
H	0	11.23807100	-2.41280800	10.48548300	L
C	0	11.08302000	-1.87005400	11.44216800	L
H	0	12.05317800	-1.47599700	11.81162200	L
H	0	10.67605500	-2.57885500	12.19190100	L
O	0	10.17669800	-0.82146000	11.26271100	L
H	0	10.55194200	-0.25666700	10.53932100	L
H	0	9.04529600	1.95868800	7.31895800	L
C	0	8.46767400	1.44462500	8.11432100	L
H	0	9.14339900	1.23411700	8.96779800	L
H	0	8.06971700	0.48242500	7.72535200	L
O	0	7.42344400	2.26564400	8.54813700	L
H	0	6.79754600	2.34199500	7.78229900	L
H	0	11.55823000	0.53724900	8.26400800	L
C	0	12.54090200	0.03774900	8.12696600	L
H	0	12.55019300	-0.89830600	8.72020400	L
H	0	13.35520300	0.69964300	8.49111800	L
O	0	12.74216200	-0.26999500	6.77912800	L
H	0	12.71381100	0.59477800	6.29496300	L
H	0	12.46051500	6.68272000	8.05345200	L

C	0	12.56464000	7.51075200	8.78579600	L
H	0	12.47469400	7.09455800	9.81003400	L
H	0	11.75466900	8.25430200	8.63057400	L
O	0	13.81367900	8.12309100	8.64522300	L
H	0	13.84519100	8.46814200	7.71506100	L
H	0	10.91850600	-4.94950800	10.23297800	L
C	0	10.74793500	-5.61107200	11.10914400	L
H	0	11.54231000	-5.41898100	11.85926300	L
H	0	10.80145000	-6.67526600	10.79288700	L
O	0	9.50155600	-5.34256700	11.68226200	L
H	0	8.83210500	-5.50315000	10.96777600	L
H	0	9.59431700	7.67646300	10.50728600	L
C	0	8.72562400	7.80940900	9.82656100	L
H	0	8.42898200	8.87804000	9.82845200	L
H	0	7.86822500	7.20188800	10.18799500	L
O	0	9.07065800	7.43180000	8.52579900	L
H	0	9.33662800	6.47777900	8.57901400	L
H	0	7.66113800	0.87364800	-0.76738000	L
C	0	8.00433700	1.86204300	-0.40271400	L
H	0	7.15881900	2.36297100	0.11516500	L
H	0	8.32794900	2.47067400	-1.27364600	L
O	0	9.07101400	1.67816600	0.48097600	L
H	0	9.35283200	2.58721500	0.75877000	L
H	0	16.87231300	-1.64288600	7.68883500	L
C	0	15.98006900	-0.98269700	7.75116700	L
H	0	15.21384700	-1.47845200	8.37958600	L
H	0	15.55893600	-0.82280200	6.73533300	L
O	0	16.32270700	0.24281700	8.33051800	L
H	0	17.02329800	0.63486300	7.74761500	L
H	0	13.57148400	4.63730500	9.78241600	L
C	0	14.59945300	4.53303900	10.18946800	L
H	0	14.55138600	4.15707900	11.23394900	L
H	0	15.08361500	5.53106300	10.18884200	L
O	0	15.34902400	3.65878200	9.39622700	L
H	0	14.86396400	2.79323800	9.40749900	L
H	0	4.01103000	-3.24391400	10.99956300	L
C	0	3.12971700	-2.66492900	11.34825500	L
H	0	2.80136500	-3.04043200	12.33903700	L
H	0	2.30097500	-2.80416900	10.62509000	L
O	0	3.44394700	-1.30635900	11.43149000	L
H	0	4.18810000	-1.23567900	12.08282200	L
H	0	17.17196800	0.25030600	2.91884100	L
C	0	16.71197800	-0.74997800	2.76720500	L
H	0	17.39421700	-1.38973600	2.16669400	L

H	0	15.75978400	-0.62316800	2.21340200	L
O	0	16.44876300	-1.35242500	4.00080600	L
H	0	17.32440000	-1.42964700	4.46100400	L
H	0	6.00436800	-6.18146100	13.51483200	L
C	0	5.62253900	-6.42709500	12.50290800	L
H	0	6.48608700	-6.65000500	11.84000700	L
H	0	5.06400500	-5.54976700	12.11134700	L
O	0	4.77898700	-7.53914300	12.58704200	L
H	0	4.46687600	-7.71600600	11.66195300	L
H	0	16.53440900	-5.59230300	5.57430600	L
C	0	16.20150000	-4.87993900	6.35987100	L
H	0	17.07528300	-4.59971500	6.98318000	L
H	0	15.44102300	-5.36534600	7.00852700	L
O	0	15.67003800	-3.72785100	5.77384400	L
H	0	14.91207900	-4.03247300	5.21172300	L
H	0	9.69081700	-6.60970300	3.58396500	L
C	0	9.07221000	-5.70148700	3.42494100	L
H	0	8.04213600	-5.90459300	3.77930200	L
H	0	9.03557700	-5.45843400	2.34196000	L
O	0	9.59912800	-4.62560000	4.14430700	L
H	0	10.52313800	-4.49785600	3.80860100	L
H	0	13.24365000	6.25489900	5.43107900	L
C	0	14.28447600	5.89786800	5.27746000	L
H	0	14.77701400	6.54791000	4.52591400	L
H	0	14.27104300	4.85557800	4.89390900	L
O	0	14.99965100	5.96037200	6.47732300	L
H	0	14.52033200	5.36810200	7.11271700	L
H	0	5.13934800	-1.76302400	9.09588400	L
C	0	5.14837100	-0.88249400	8.41888800	L
H	0	4.16907300	-0.79071300	7.90298800	L
H	0	5.93864500	-1.02800900	7.65574200	L
O	0	5.42193500	0.27855900	9.14535000	L
H	0	4.65877800	0.40386800	9.76553300	L
H	0	15.61702700	2.67092000	4.37338800	L
C	0	15.45248000	1.84254600	5.09482400	L
H	0	16.41786100	1.33514300	5.30683000	L
H	0	14.75558000	1.10755100	4.64431000	L
O	0	14.89606100	2.33751200	6.27782600	L
H	0	15.56334000	2.97163100	6.64775200	L
H	0	8.97709700	-3.24946400	9.51010500	L
C	0	8.44017700	-2.55908800	8.82598200	L
H	0	8.07592700	-1.67489100	9.39195500	L
H	0	7.56790000	-3.08962100	8.39712900	L
O	0	9.28470400	-2.15551200	7.78807900	L

H	0	10.01668300	-1.64134400	8.21616300	L
H	0	3.40753200	-1.69353600	3.54896400	L
C	0	2.30654400	-1.70890000	3.40890100	L
H	0	1.91817600	-0.67519200	3.51440100	L
H	0	1.84388800	-2.34658300	4.19048500	L
O	0	1.98815900	-2.19371300	2.13620300	L
H	0	2.32149900	-3.12708200	2.10594600	L
H	0	6.42328400	-2.39487800	11.39532300	L
C	0	7.02158000	-2.78303000	12.24712800	L
H	0	7.98989100	-2.24451200	12.27701800	L
H	0	7.21969800	-3.86550900	12.10324000	L
O	0	6.34284800	-2.57352500	13.45110400	L
H	0	5.50468100	-3.10024800	13.38668300	L
H	0	4.82582900	1.53026300	14.08378300	L
C	0	4.31782600	2.41153300	13.63591800	L
H	0	5.07143500	3.08275300	13.16955100	L
H	0	3.79211500	2.96709900	14.43914100	L
O	0	3.38517000	1.99597100	12.68161800	L
H	0	3.90299200	1.51619100	11.98440800	L
H	0	5.55873900	-5.94601200	3.99181900	L
C	0	4.44876000	-5.97019400	4.02497000	L
H	0	4.13088600	-6.58276800	4.89331400	L
H	0	4.05761100	-6.43260800	3.09506600	L
O	0	3.93901000	-4.67661100	4.16328200	L
H	0	4.29851800	-4.15833000	3.39835300	L
H	0	8.81894600	-6.69431800	6.91743300	L
C	0	9.62983600	-6.17144500	7.46182600	L
H	0	9.21597200	-5.74675700	8.40074600	L
H	0	10.01462100	-5.35251100	6.81973300	L
O	0	10.64941900	-7.08347800	7.75074100	L
H	0	11.36565900	-6.56206000	8.19652500	L
H	0	9.71667100	5.10757000	5.94627300	L
C	0	10.05855700	6.07852900	5.53567700	L
H	0	10.59900800	5.89448900	4.58331100	L
H	0	10.74319400	6.55600600	6.26732400	L
O	0	8.94850500	6.89629500	5.31254900	L
H	0	9.30411000	7.73956900	4.93164900	L
H	0	6.28082300	0.22348300	12.23238100	L
C	0	7.11964100	0.83542500	11.83786100	L
H	0	6.72778700	1.79878500	11.44790300	L
H	0	7.60266300	0.28493300	11.00580000	L
O	0	8.06196000	1.06717600	12.84390000	L
H	0	7.58589100	1.57273300	13.55265300	L
H	0	4.49688300	-0.30315900	5.40162300	L

C	0	5.15849700	0.51596700	5.04695000	L
H	0	6.17564500	0.10753000	4.88717800	L
H	0	5.21445700	1.31593200	5.81537200	L
O	0	4.67968100	1.03943000	3.84247900	L
H	0	3.74778400	1.32736800	4.01914700	L
H	0	15.60580800	-0.92817700	11.03354800	L
C	0	14.88974600	-0.20495900	11.47397900	L
H	0	14.55430100	-0.58945000	12.46146800	L
H	0	14.01666200	-0.10736400	10.79507800	L
O	0	15.52284400	1.03220300	11.62645300	L
H	0	14.84627400	1.63819600	12.02562900	L
H	0	12.65605000	3.09514400	7.98841900	L
C	0	11.79916700	3.50693600	7.41559900	L
H	0	11.39485300	2.71291900	6.75707700	L
H	0	12.14529900	4.35087600	6.78401800	L
O	0	10.79306900	3.93433400	8.28687200	L
H	0	11.20591300	4.62635200	8.86481600	L
H	0	9.98739500	-0.47096400	3.40486500	L
C	0	9.49490000	-0.75133800	4.36007800	L
H	0	9.69605300	-1.82164100	4.56660300	L
H	0	8.39918300	-0.60615700	4.26799300	L
O	0	9.99604800	0.02156700	5.41095900	L
H	0	9.84909500	0.96672800	5.14874300	L
H	0	13.41050600	-3.01577800	7.42438100	L
C	0	12.56302200	-3.72962100	7.35571900	L
H	0	11.87114400	-3.57925100	8.21215700	L
H	0	12.96450200	-4.76175600	7.39947400	L
O	0	11.88279100	-3.55255100	6.14837900	L
H	0	11.52076600	-2.62987500	6.17072700	L
H	0	7.16197100	-3.19982300	4.28646600	L
C	0	6.69153500	-3.03922100	5.27991200	L
H	0	5.66139200	-2.65695600	5.13179600	L
H	0	7.26984500	-2.28379900	5.85236800	L
O	0	6.63709800	-4.24426300	5.98533900	L
H	0	7.57861800	-4.52340500	6.12130700	L
H	0	3.68609400	2.02049500	7.77431200	L
C	0	2.76003100	2.39892100	7.29149700	L
H	0	2.98035900	3.37516800	6.81829800	L
H	0	1.96937800	2.54619700	8.05586500	L
O	0	2.31837300	1.50441400	6.31289200	L
H	0	2.22148600	0.62800600	6.76659200	L
H	0	14.60754100	3.43662900	2.08838200	L
C	0	14.67854300	2.52620400	1.45848500	L
H	0	14.07844300	2.68277100	0.53761600	L

H	0	14.27304100	1.66329400	2.02858200	L
O	0	16.01744600	2.29816600	1.12690500	L
H	0	16.02293800	1.48546400	0.55722300	L
H	0	12.26024100	-4.65843100	2.08101200	L
C	0	12.15013500	-4.91026000	1.00579300	L
H	0	11.09124200	-5.16541700	0.80699100	L
H	0	12.78324300	-5.78820300	0.75916800	L
O	0	12.51237300	-3.81641600	0.21621900	L
H	0	13.45228000	-3.61113500	0.45400200	L
H	0	7.46821300	-8.20229900	4.73835200	L
C	0	6.86619600	-8.51402400	5.61738800	L
H	0	6.30637200	-7.64008800	6.01490200	L
H	0	7.54963600	-8.88717300	6.40678900	L
O	0	5.98455600	-9.53689800	5.25538000	L
H	0	5.33262800	-9.12739200	4.63006000	L
H	0	14.01418000	-3.65555700	11.64174900	L
C	0	14.15522400	-3.72575700	10.54371700	L
H	0	13.75689100	-2.80127900	10.07437700	L
H	0	13.59734900	-4.60957600	10.16593600	L
O	0	15.51798600	-3.85955500	10.26185900	L
H	0	15.58801100	-3.91452800	9.27365300	L
H	0	5.89806400	-1.77186300	2.68656000	L
C	0	5.81016400	-1.29858300	1.68796300	L
H	0	4.73771800	-1.23637200	1.41670900	L
H	0	6.33240000	-1.91715800	0.93075000	L
O	0	6.35792900	-0.01374200	1.71277600	L
H	0	7.31442000	-0.13263500	1.94232700	L
H	0	7.76173900	4.29854000	4.60394600	L
C	0	7.42568500	3.25261600	4.46433700	L
H	0	6.35196300	3.26017700	4.18221900	L
H	0	7.55923900	2.70309200	5.41933600	L
O	0	8.18535000	2.65592500	3.45522300	L
H	0	7.85408600	1.72514800	3.38141800	L
H	0	10.73687100	2.31521500	12.62653100	L
C	0	11.75088400	2.18381500	12.19680700	L
H	0	12.13753000	1.18464800	12.49333100	L
H	0	12.41581200	2.97868000	12.59825600	L
O	0	11.67492200	2.27827500	10.80437100	L
H	0	12.59896300	2.14161000	10.47167200	L
H	0	8.27937800	6.08158800	2.82541200	L
C	0	8.78131400	5.56634900	1.98040700	L
H	0	9.56016200	4.88970400	2.38600700	L
H	0	9.26783200	6.31437300	1.32151800	L
O	0	7.85280300	4.81660600	1.25424400	L

H	0	7.16464300	5.45966300	0.94512500	L
H	0	-0.74672900	10.68575600	3.66225400	L
C	0	-1.18848600	10.71136600	2.64367700	L
H	0	-2.09937600	10.07932000	2.61502700	L
H	0	-0.44972900	10.30184900	1.92742300	L
O	0	-1.50345000	12.02428900	2.28147800	L
H	0	-2.10909300	12.36433400	2.98900200	L
H	0	4.35038300	14.55212200	-3.93252600	L
C	0	3.44427500	14.42449800	-3.30843900	L
H	0	3.75666100	14.27290600	-2.25348000	L
H	0	2.88764800	13.53282500	-3.66693800	L
O	0	2.64917900	15.56905300	-3.41806800	L
H	0	1.85930600	15.40894700	-2.84001200	L
H	0	1.75500600	5.85216200	-3.12083700	L
C	0	2.70967000	5.29069000	-3.03180600	L
H	0	3.04090100	4.94716700	-4.03457900	L
H	0	2.54155000	4.40335300	-2.39024600	L
O	0	3.69259600	6.09514400	-2.45046000	L
H	0	3.79720000	6.87677700	-3.05151500	L
H	0	10.14962900	8.89255500	-4.00664400	L
C	0	9.80534800	9.87201500	-3.60961500	L
H	0	8.69568100	9.89751900	-3.58702300	L
H	0	10.16523100	10.67751500	-4.28195800	L
O	0	10.31918600	10.08093400	-2.32615400	L
H	0	9.96096300	9.34509500	-1.76566300	L
H	0	9.41029000	8.75479300	0.63024100	L
C	0	9.13017600	9.50301500	1.39753300	L
H	0	8.35986700	10.17788400	0.96936100	L
H	0	10.03550600	10.08600900	1.67170800	L
O	0	8.61919800	8.84209800	2.51802500	L
H	0	8.42756500	9.54627000	3.18957100	L
H	0	-0.21193200	5.26008900	3.18768700	L
C	0	-0.68568600	6.06253200	3.78508900	L
H	0	-0.07375100	6.23633900	4.69570500	L
H	0	-1.70744700	5.73673700	4.07482800	L
O	0	-0.75152500	7.22798400	3.01705500	L
H	0	-1.20074200	7.90290000	3.58812200	L
H	0	4.63374100	11.13294800	6.46753800	L
C	0	4.51479100	12.23188000	6.56895700	L
H	0	3.61237200	12.56371500	6.01497600	L
H	0	5.40346800	12.72885000	6.13074800	L
O	0	4.40800000	12.58190400	7.91815600	L
H	0	3.59163200	12.12796400	8.25356900	L
H	0	11.25735700	12.95992900	7.88258900	L

C	0	10.43308700	12.21828300	7.90212000	L
H	0	10.14680900	11.97465100	6.85616000	L
H	0	9.56529700	12.66285300	8.43533900	L
O	0	10.86790400	11.06726900	8.56626400	L
H	0	10.10686100	10.43151100	8.53791500	L
H	0	8.77435200	7.08060400	-2.65231600	L
C	0	9.31662800	6.28427800	-2.10149200	L
H	0	9.56559600	6.64177200	-1.08074700	L
H	0	10.25939000	6.05655700	-2.63967500	L
O	0	8.53628100	5.12655400	-2.03048400	L
H	0	7.69388500	5.38968700	-1.57841900	L
H	0	4.68765800	15.23547800	1.75487200	L
C	0	5.56635400	14.56379900	1.64942600	L
H	0	5.85108100	14.19161000	2.65384900	L
H	0	6.42057200	15.12954700	1.22009300	L
O	0	5.25180400	13.47479000	0.83179500	L
H	0	4.94492400	13.85763700	-0.03002300	L
H	0	4.07189100	8.01394300	9.33977700	L
C	0	4.68143100	8.52371700	8.56633000	L
H	0	5.75207100	8.28398700	8.74061500	L
H	0	4.52311000	9.62006500	8.65313300	L
O	0	4.28625000	8.07720400	7.30182400	L
H	0	4.84749600	8.56864300	6.64854500	L
H	0	1.39964400	9.27885700	3.73333100	L
C	0	1.58621700	8.89274100	4.75666100	L
H	0	2.07286600	7.89870700	4.68326100	L
H	0	0.62007100	8.78133700	5.29281000	L
O	0	2.42547900	9.76646200	5.45165600	L
H	0	1.93524400	10.62673500	5.50768800	L
H	0	6.52201900	5.78060800	8.47900800	L
C	0	6.50868900	5.46942900	7.41519600	L
H	0	7.38103900	4.80831700	7.22525600	L
H	0	6.58006800	6.37482100	6.77700500	L
O	0	5.31606400	4.78933100	7.15409700	L
H	0	5.35134200	4.52863600	6.19824200	L
H	0	2.00535400	4.67373300	4.87031300	L
C	0	2.63522300	4.42728300	3.98920700	L
H	0	3.58579600	3.95939200	4.32356100	L
H	0	2.87019400	5.36656800	3.44957600	L
O	0	1.94942000	3.56253800	3.13006100	L
H	0	1.71425000	2.76570100	3.67189800	L
H	0	2.51536000	5.95444600	7.11801800	L
C	0	1.61231700	6.24747800	7.69557900	L
H	0	1.00940100	6.97242700	7.10887100	L

H	0	1.93261100	6.73246100	8.63937400	L
O	0	0.84716100	5.11887100	7.99907400	L
H	0	0.59221500	4.72217900	7.12721300	L
H	0	9.48333100	12.09254600	-0.21566200	L
C	0	8.77765100	12.77061200	-0.74161700	L
H	0	7.77530600	12.29535300	-0.77011500	L
H	0	9.12842300	12.93522400	-1.78247500	L
O	0	8.68874600	13.99166600	-0.06643600	L
H	0	9.60892400	14.36212800	-0.04768300	L
H	0	10.34037300	14.29100100	5.14638400	L
C	0	11.36228300	13.99722200	4.82310400	L
H	0	11.37860100	12.91616000	4.56641800	L
H	0	12.06649600	14.17494700	5.66134800	L
O	0	11.75872200	14.76146700	3.72119200	L
H	0	11.09893500	14.57248200	3.00460200	L
H	0	-4.34023100	13.50813800	2.84369200	L
C	0	-4.53845500	14.50765600	2.40158600	L
H	0	-4.07378200	15.29491200	3.03326700	L
H	0	-5.63522300	14.67165900	2.37254300	L
O	0	-4.02971000	14.57192300	1.10090300	L
H	0	-3.06214300	14.36638700	1.17640400	L
H	0	6.00713200	12.68249200	-2.99661400	L
C	0	6.43450400	12.21822700	-3.91033900	L
H	0	5.80890500	12.47683200	-4.79125800	L
H	0	6.43406500	11.11684400	-3.78064600	L
O	0	7.74652100	12.65748200	-4.10930300	L
H	0	7.70036700	13.64744800	-4.15298400	L
H	0	0.94575100	11.01960600	8.42245200	L
C	0	0.79015400	9.92152200	8.42650400	L
H	0	1.76298900	9.42253700	8.22940400	L
H	0	0.06583600	9.66060300	7.62572900	L
O	0	0.29239000	9.53190800	9.67335200	L
H	0	0.16366000	8.54953300	9.62155200	L
H	0	5.67984700	8.61261200	-0.04177100	L
C	0	6.37661900	8.04602400	-0.69507900	L
H	0	5.85028800	7.15507500	-1.09119500	L
H	0	7.25200000	7.70726400	-0.10302300	L
O	0	6.79617300	8.84409500	-1.76325000	L
H	0	7.26195200	9.62073600	-1.35799400	L
H	0	1.43187400	9.52110100	-1.81774800	L
C	0	1.17835800	8.92191000	-2.71729400	L
H	0	0.69139300	7.98032900	-2.39383200	L
H	0	2.10319300	8.66935600	-3.27643300	L
O	0	0.29913500	9.63594000	-3.53447200	L

H	0	0.79578300	10.44067700	-3.83253800	L
H	0	7.44889100	8.74507100	6.74685500	L
C	0	7.58507300	9.82228100	6.52881400	L
H	0	6.89737800	10.10628500	5.70528900	L
H	0	8.63573800	9.99483900	6.21515200	L
O	0	7.29653300	10.56194900	7.67926600	L
H	0	7.40882100	11.51430000	7.42657800	L
H	0	-2.67206100	16.20837000	-1.31124000	L
C	0	-1.63627400	16.37656800	-0.94450400	L
H	0	-1.27279000	15.46679100	-0.42124700	L
H	0	-1.64363700	17.22168500	-0.22640800	L
O	0	-0.78965600	16.69016600	-2.01223800	L
H	0	-0.82533300	15.91266300	-2.62727500	L
H	0	6.47285800	15.95615800	-3.15744200	L
C	0	6.57236200	15.85806700	-2.05470700	L
H	0	7.53590800	16.30118200	-1.72270100	L
H	0	6.56623600	14.78163100	-1.79139400	L
O	0	5.50373500	16.49209500	-1.41347100	L
H	0	5.53896900	17.44220300	-1.69706900	L
H	0	2.61629600	12.54678800	3.74822300	L
C	0	1.83611400	12.57888100	2.95978900	L
H	0	0.87610500	12.91662500	3.40234900	L
H	0	1.70119600	11.55966000	2.54630200	L
O	0	2.22548200	13.44064800	1.93153900	L
H	0	2.31932500	14.33561700	2.34710300	L
H	0	1.13375300	12.93087500	6.10974500	L
C	0	0.11864200	13.32083900	6.33432000	L
H	0	0.03522500	14.34965400	5.92863900	L
H	0	-0.03424400	13.35566100	7.43429700	L
O	0	-0.85400700	12.51284200	5.73825300	L
H	0	-0.71675700	11.60217400	6.10641000	L
H	0	0.28251200	15.65177100	3.38964900	L
C	0	-0.66687200	15.37887700	2.88018800	L
H	0	-1.27660600	14.73137800	3.54544000	L
H	0	-0.42858200	14.81325800	1.95652000	L
O	0	-1.38116800	16.53174300	2.54126600	L
H	0	-1.57483400	16.99500700	3.39693700	L
H	0	-2.05669500	8.55272500	0.56481800	L
C	0	-1.42986800	8.49037100	-0.34835200	L
H	0	-0.68981100	7.67714700	-0.21622000	L
H	0	-0.88818700	9.44921500	-0.49704300	L
O	0	-2.22796400	8.20912800	-1.46051900	L
H	0	-2.82864400	8.99006900	-1.57125100	L
H	0	-3.85047500	13.37203700	-1.99203800	L

C	0	-2.78826000	13.05672700	-2.02661200	L
H	0	-2.35966800	13.14694100	-1.00552200	L
H	0	-2.23928200	13.72235900	-2.72569400	L
O	0	-2.71515000	11.73256000	-2.46842300	L
H	0	-1.75873300	11.47564600	-2.40724200	L
H	0	1.31297200	8.59967100	1.22885800	L
C	0	2.16962500	7.89690500	1.26624800	L
H	0	1.82027300	6.92385000	1.67206600	L
H	0	2.95052500	8.32083000	1.93284200	L
O	0	2.67611200	7.72596100	-0.02450000	L
H	0	3.48042000	7.15508200	0.07207800	L
H	0	4.09656700	14.60357500	4.47060100	L
C	0	3.46714000	15.44760800	4.82485100	L
H	0	2.43918100	15.08378100	5.03847400	L
H	0	3.41548700	16.21395700	4.02461900	L
O	0	4.02649500	16.01801300	5.97219700	L
H	0	4.05103700	15.29598000	6.65229800	L
H	0	9.54908200	12.46918200	2.61091000	L
C	0	8.53560200	12.73524700	2.97368400	L
H	0	7.78794500	12.38237000	2.23189200	L
H	0	8.46993900	13.83962100	3.07613200	L
O	0	8.31651300	12.12429300	4.21127000	L
H	0	7.38215700	12.34375400	4.46158200	L
H	0	2.99987700	15.75530900	-0.17517600	L
C	0	2.09199400	16.34125800	0.07321100	L
H	0	1.22777800	15.64652800	0.13462900	L
H	0	1.91662100	17.08918300	-0.72966600	L
O	0	2.28138800	16.98616600	1.29923200	L
H	0	1.43338100	17.46503400	1.48971500	L
H	0	12.96834500	7.66434500	2.93816400	L
C	0	12.14995700	7.28979700	2.29065900	L
H	0	11.17800500	7.60242000	2.72750500	L
H	0	12.20433700	6.18082000	2.25111700	L
O	0	12.29836300	7.82783700	1.00862700	L
H	0	11.56840800	7.43754700	0.46183900	L
H	0	3.62216500	11.65414800	-1.94676900	L
C	0	4.13827800	10.87369500	-1.34946200	L
H	0	5.17277500	11.20330900	-1.11413500	L
H	0	4.18516900	9.94211700	-1.94944800	L
O	0	3.43439800	10.62311400	-0.16861500	L
H	0	3.41366500	11.48234600	0.32542600	L
H	0	0.25567800	13.75467400	-1.54265800	L
C	0	0.86770800	12.88228900	-1.24001800	L
H	0	1.83502000	13.24858700	-0.83569600	L

H	0	1.05028200	12.24784200	-2.13278800	L
O	0	0.18043200	12.15652300	-0.26369400	L
H	0	0.76296000	11.38719000	-0.03497300	L
H	0	6.24265900	16.17942700	-7.09561400	L
C	0	6.67719900	15.31764100	-6.54891500	L
H	0	6.01236700	15.06517400	-5.69595600	L
H	0	7.68192700	15.60258300	-6.16843100	L
O	0	6.78178300	14.22811000	-7.41887400	L
H	0	7.16505300	13.48653100	-6.88293900	L
H	0	5.09845400	9.98365600	-5.76501800	L
C	0	4.11728300	9.67529400	-5.34586500	L
H	0	4.28887600	8.88160300	-4.59116600	L
H	0	3.47515200	9.26975000	-6.15592200	L
O	0	3.48901100	10.76364400	-4.73384100	L
H	0	3.33227000	11.42802600	-5.45316400	L
H	0	1.13864800	5.47434000	-0.45254000	L
C	0	0.29218900	4.83137300	-0.13345900	L
H	0	-0.44195400	4.74614400	-0.96115600	L
H	0	-0.20609700	5.30017800	0.73705300	L
O	0	0.75882100	3.56497800	0.23075800	L
H	0	1.16941600	3.18047100	-0.58576000	L
H	0	12.38396000	10.32751300	5.99072700	L
C	0	11.74067600	9.52861400	5.56290800	L
H	0	11.03751900	9.17919200	6.34528700	L
H	0	12.36896500	8.67054200	5.24240900	L
O	0	11.01051900	10.02643400	4.47958900	L
H	0	11.68040200	10.30272200	3.80181100	L
H	0	5.00763900	10.03488400	4.12005700	L
C	0	4.91252100	10.63807000	3.19546100	L
H	0	5.02775000	11.71300400	3.45293400	L
H	0	3.90812800	10.45810200	2.76019700	L
O	0	5.90230900	10.24948800	2.28984600	L
H	0	5.78263300	10.82203900	1.48904200	L
H	0	5.78758100	5.94005200	4.13620700	L
C	0	5.56061700	7.00560400	3.92158500	L
H	0	6.30894600	7.64255900	4.43543600	L
H	0	4.54998500	7.25955500	4.30697500	L
O	0	5.62560300	7.24304800	2.54612600	L
H	0	4.94942400	6.64631800	2.13423400	L
H	0	4.89320100	4.93239300	0.02386600	L
C	0	4.17461800	4.22500600	0.48943200	L
H	0	3.74442100	3.58309900	-0.30392300	L
H	0	3.35031400	4.79021700	0.97281500	L
O	0	4.82675500	3.42125700	1.42916200	L

H	0	5.17827700	4.04029600	2.11964800 L
H	0	8.41839100	15.41388100	7.18931600 L
C	0	7.65628900	14.80474700	6.66158500 L
H	0	6.74139900	14.75024000	7.29056700 L
H	0	8.06454000	13.78417900	6.50412500 L
O	0	7.36267700	15.40152300	5.43164600 L
H	0	6.67915000	14.82347200	5.00446500 L
Co	0	-1.17804300	-0.07549600	0.56147900 H
O	0	0.02887900	-3.89358100	-0.43674800 H
C	0	1.22771700	-3.79239900	-1.16513200 H
H	0	2.11689500	-4.04498900	-0.56654100 H
H	0	1.15541600	-4.51889100	-1.98098400 H
H	0	1.37905500	-2.80079200	-1.61516300 H
H	0	0.01188400	-3.26230400	0.30031800 H

INT21$_{\mathbf{B3LYP}}$ (Doublet, S**2 after annihilation: 0.7501)
E= -1366.05619120 a.u.

C	3.494724	1.478298	1.791134
C	2.115684	1.181815	1.214649
C	1.589764	2.276276	0.321359
C	2.458130	3.384526	-0.163877
H	4.264712	1.511208	1.015676
H	3.392900	2.999523	-0.588020
C	0.157502	2.347132	0.028833
N	-0.526706	1.151649	0.120242
O	-0.384725	3.425810	-0.281985
C	-1.887990	1.093637	-0.026160
C	-2.861167	2.036067	0.352013
C	-4.205475	1.745623	0.182507
H	-2.533111	2.971741	0.781355
C	-3.634241	-0.395108	-0.726000
C	-4.602423	0.515483	-0.362637
H	-4.951626	2.472658	0.485733
H	-3.823958	-1.371425	-1.153802
H	-5.646212	0.259643	-0.498873
O	-1.399912	-0.975573	-0.989375
N	-2.327871	-0.080722	-0.565192
Co	0.281557	-0.545412	-0.290801
H	3.460151	2.452423	2.287216
H	3.778628	0.742296	2.544970
H	1.939562	3.978494	-0.918352
H	2.744990	4.062963	0.653431
H	1.407933	1.012065	2.035835
C	-0.873899	-2.609445	3.166564

H	-1.845056	-2.154094	3.391580
H	-0.926371	-3.678621	3.381075
H	-0.128992	-2.134141	3.810478
C	-0.546823	-2.386866	1.699747
O	-0.637260	-3.299327	0.871574
O	-0.196082	-1.152749	1.439512
O	2.112661	-0.081905	0.426152
C	2.793572	-1.185722	1.088349
H	2.568587	-2.074659	0.503871
H	3.868535	-1.001154	1.072832
H	2.436477	-1.302389	2.114122
C	1.988382	-1.781105	-3.505004
H	1.496711	-2.688065	-3.868895
H	1.970087	-1.007042	-4.273641
H	3.026859	-2.036184	-3.266531
C	1.310445	-1.308105	-2.262966
O	0.934404	-0.095209	-2.107080
O	1.083674	-2.088626	-1.275655

TS13$_{B3LYP}$ (Doublet, S**2 after annihilation: 0.7522)
E= -1366.03446402 a.u.
Imaginary Frequency -- -169.6118

C	2.631751	2.991802	-0.664720
C	1.429010	2.093344	-0.383300
C	0.135454	2.724893	-0.245919
C	0.031629	4.087773	0.305613
H	2.602240	3.370431	-1.689713
H	0.253819	4.783316	-0.522948
C	-1.168766	2.067706	-0.579777
N	-1.258033	0.767291	-0.215645
O	-2.044405	2.798245	-1.067399
C	-2.478222	0.160181	-0.006978
C	-3.769519	0.711383	-0.089217
C	-4.878874	-0.052826	0.238794
H	-3.867173	1.733939	-0.422067
C	-3.443729	-1.914047	0.697836
C	-4.716125	-1.384420	0.636071
H	-5.869564	0.384260	0.173801
H	-3.211505	-2.931308	0.985446
H	-5.560799	-2.014884	0.887232
O	-1.154962	-1.723146	0.463739
N	-2.367852	-1.154018	0.384809
Co	0.218107	-0.528158	-0.153582
H	2.627990	3.838471	0.022445

H	3.557467	2.442143	-0.497461
H	-0.970733	4.308500	0.670514
H	0.787448	4.276893	1.072423
H	1.510485	1.723306	0.707687
C	2.366133	-1.079990	3.304849
H	1.727521	-1.947521	3.114471
H	3.401968	-1.402269	3.431389
H	2.023752	-0.621100	4.239837
C	2.236141	-0.066598	2.174188
O	3.260799	0.470874	1.700500
O	1.033226	0.206563	1.798008
O	1.348834	0.879976	-1.151647
C	2.545328	0.423047	-1.832323
H	2.220322	-0.434890	-2.418092
H	2.912892	1.200194	-2.503093
H	3.312387	0.128180	-1.114384
C	2.342908	-3.884750	-1.329592
H	2.494544	-4.340967	-0.348032
H	1.971224	-4.629699	-2.036364
H	3.316629	-3.526636	-1.685287
C	1.391296	-2.707698	-1.246345
O	0.609525	-2.450941	-2.186299
O	1.487598	-1.980546	-0.179443

Alternative methanol-assisted proton transfer pathways

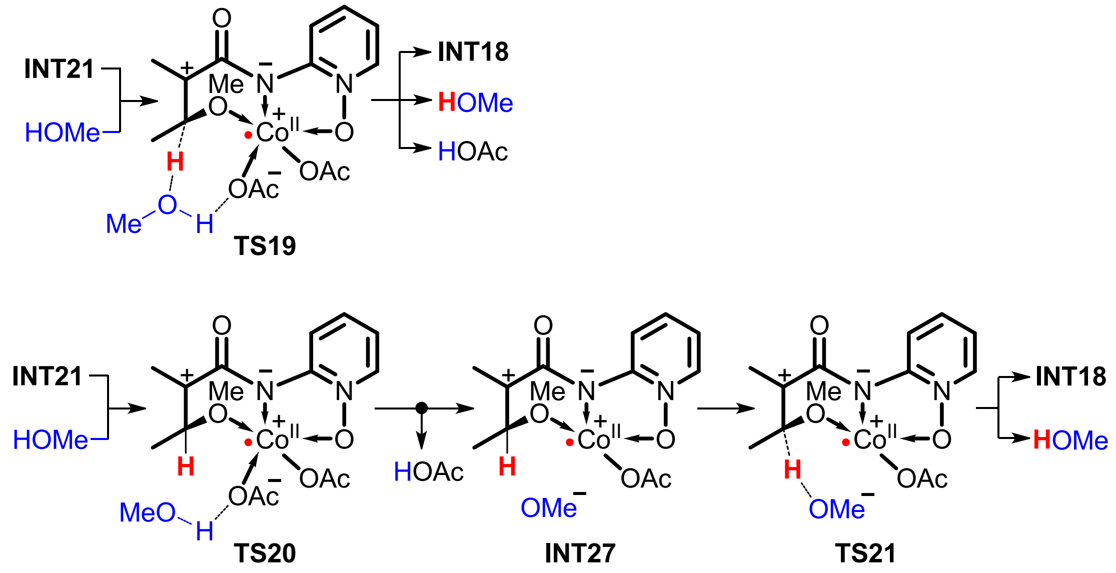

Scheme S1. Alternative methanol-assisted proton transfer pathways

TD-DFT Computation of INT2 and INT7 Excitation

Calculated excited state energies and associated molecular orbitals of **INT2** and **INT7**

INT2 (Excited State 4)

Energy: 1.3670 eV 906.95 nm Oscillator Strength=0.0026

89A -> 92A	-0.12800
90A -> 92A	0.39071
91A -> 92A	0.85483

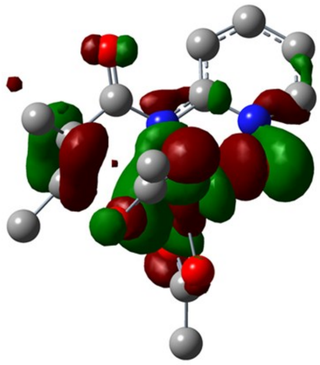

91A

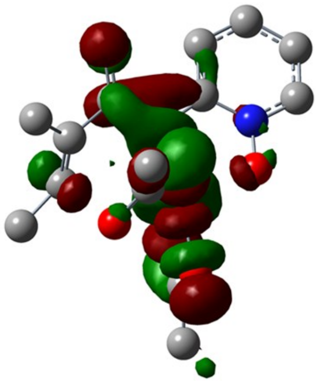

92A

INT7 (Excited State 5)

Energy: 1.6290 eV 761.09 nm Oscillator Strength=0.0012

82A -> 85A	-0.14334
83A -> 85A	0.56518
84A -> 85A	0.69556

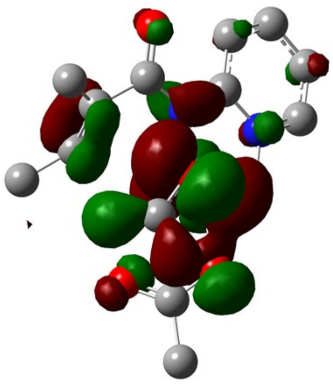

84A

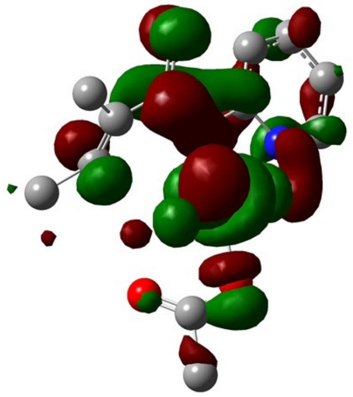

85A

Reaction Kinetic Isotope Effect Measurements

A 10 mL schlenk tube was equipped with a magnetic stir bar and charged with 2-benzamidopyridine 1-oxide **1a** (16.0 mg, 0.075 mmol), **1a-*d*$_5$** (16.4 mg, 0.075 mmol), Ag_2O (69 mg, 0.3 mmol, 2 equiv), NaOAc (12 mg, 0.15 mmol, 1 equiv), Cp*Co(CO)I_2 (14 mg, 0.03 mmol, 20 mol%), and EtOH (1.2 mL). Resulting mixture was heated at 70 ^{o}C for 2.5 h under an air atmosphere, and cooled to RT. Product was purified using column chromatography to give the desired product less than 20% yield. ^{1}H NMR (400 MHz, $CDCl_3$): δ 12.30 (s, 2.0H), 8.74 (dd, *J* = 8.5 Hz, *J* = 1.7 Hz, 2.0H), 8.31-8.28 (m, 2.9H), 7.55-7.51 (m, 1.0H), 7.38-7.34 (m, 2.0H), 7.13-7.09 (m, 1.0H), 7.07 (d, *J* = 8.3 Hz, 1.0H), 7.01-6.97 (m, 2.0H), 4.35 (q, *J* = 7.0 Hz, 4.1 H), 1.72 (t, *J* = 7.0 Hz, 6.0H). The KIE value was calculated as k_H/k_D = 1.0.

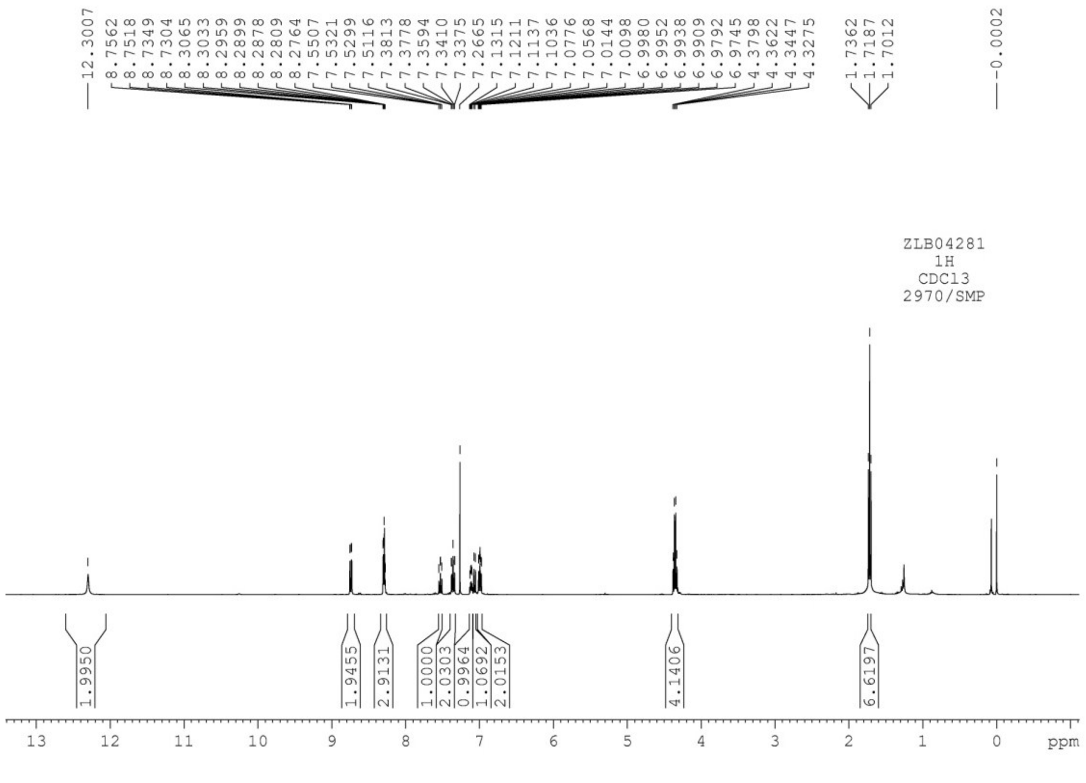

Reaction System under Added TEMPO

A 10 mL oven-dried two-necked Schlenk tube equipped with a magnetic stir bar and charged with 2-benzamidopyridine 1-oxide **1a** (32.1 mg, 0.15 mmol), TEMPO (35 mg, 0.23 mmol, 1.5 equiv), Ag_2O (69 mg, 0.3 mmol, 2 equiv), NaOAc (12 mg, 0.15 mmol, 1 equiv), Cp*Co(CO)I_2 (14 mg, 0.03 mmol, 20 mol%), and EtOH (1.2 mL). The vessel was heated at 70 ^oC for 12 h under an air atmosphere, and cooled to RT. The alcohol was evaporated under reduced pressure, and 2 N HCl (5 mL) was added to the residue. The mixture was extracted with CH_2Cl_2 and the organic layer was collected and monitored by TLC. The result showed that no reaction occurred.

Experimental EPR spectrum of the reaction system

A 10 mL two-necked schlenk tube was equipped with a magnetic stir bar and charged with aromatic amides **1a** (0.15 mmol, 32.1 mg), Ag_2O (69 mg, 0.3 mmol, 2 equiv), NaOAc (12.3 mg, 0.15 mmol, 1 equiv), $Cp^*Co^{III}(CO)I_2$ (51.0 mg, 0.15 mmol, 1 equiv), and ethanol (1.5 mL). The schlenk tube was stirred at room temperature for 24 h. Then CH_2Cl_2 (20 mmL) was added to the reaction system, and the mixture subsequently filtered through a pad of Celite. The residue was evaporated under reduced pressure, and detected by a Bruker E500 spectrometer at room temperature. For the experimental EPR spectrum of the reaction system, instrument settings were modulation frequency: 100.00 KHz; modulation amplitude: 2.00 G; sweep width: 6000.00 G; time constant: 40.960 ms; conversion: 80.000 ms; sweep time: 81.920 s; receiver gain, 5.02 x 10^3. The microwave power was 20.293 mW, and the frequency was 9.857 GHz, and the g value is labeled in the following EPR spectrum.

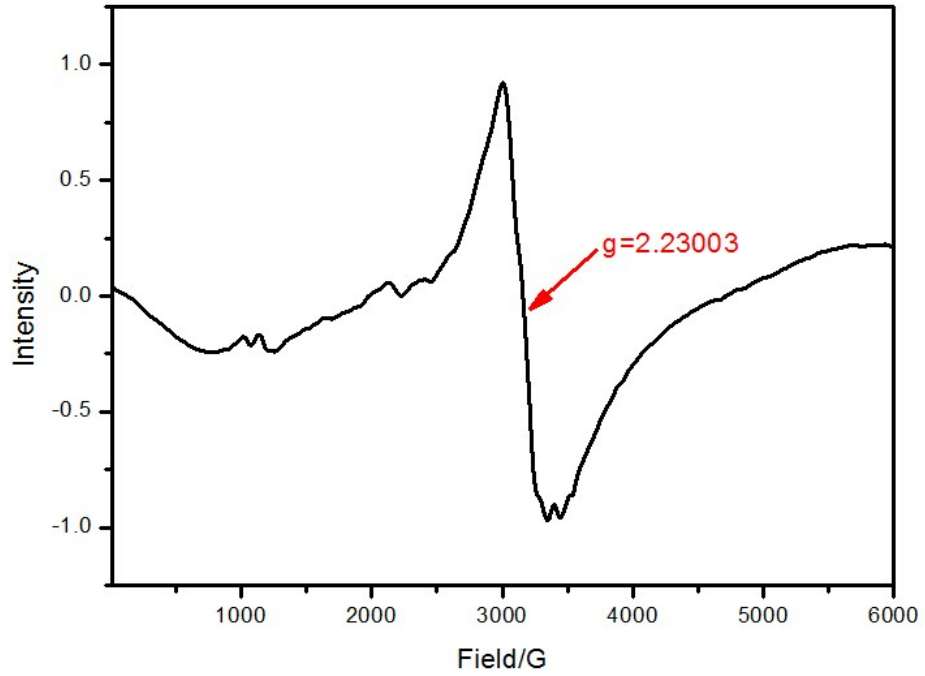

Characterization of Products

2-(2-ethoxybenzamido)pyridine 1-oxide (3aa): purified by analytical TLC on silica gel with CH_2Cl_2/acetone (6:1) as an eluent R_f = 0.35; white solid (33 mg, 85%), mp 140-141 ^oC (dichloromethane). ^1H NMR (400 MHz, $CDCl_3$) δ 12.31 (s, 1H), 8.74 (dd, *J* = 8.5 Hz, *J* = 1.8 Hz, 1H), 8.31-8.28 (m, 2H), 7.55-7.51 (m, 1H), 7.38-7.34 (m, 1H), 7.13-7.09 (m, 1H), 7.07 (d, *J* = 8.4 Hz, 1H), 7.02-6.98 (m, 1H), 4.35 (q, *J* = 7.0 Hz, 2H), 1.72 (t, *J* = 7.0 Hz, 3H). ^{13}C NMR (100 MHz, $CDCl_3$) δ 164.0, 157.5, 145.4, 137.4, 134.3, 132.7, 127.8, 121.1, 120.5, 118.5, 115.9, 112.4, 65.4, 14.8. HRMS (positive ESI) Calcd. For $C_{14}H_{15}N_2O_3$ (M + H) 259.1083, Found: 259.1081.

2-(2-ethoxy-4-methoxybenzamido)pyridine 1-oxide (**3ba**): purified by analytical TLC on silica gel with CH_2Cl_2/acetone (2:1) as an eluent R_f = 0.29; white solid (36 mg, 83%); mp 196-197 ^oC (dichloromethane). ^1H NMR (400 MHz, $CDCl_3$) δ 12.16 (s, 1H), 8.74 (dd, *J* = 8.5 Hz, *J* = 1.3 Hz, 1H), 8.29-8.27(m, 1H), 8.24 (d, *J* = 8.9 Hz, 1H), 7.36-7.32 (m, 1H), 6.99-6.96 (m, 1H), 6.63 (dd, *J* = 8.8 Hz, *J* = 2.2 Hz, 1H), 6.54 (d, *J* = 2.2 Hz, 1H), 4.31 (q, *J* = 7.0 Hz, 2H), 3.83 (s, 3H), 1.72 (t, *J* = 7.0 Hz, 3H). ^{13}C NMR (100 MHz, $CDCl_3$) δ 164.6, 163.7, 159.0, 145.6, 137.3, 134.4, 128.1, 118.1, 115.7, 113.5, 105.7, 99.1, 65.4, 55.6, 14.7. HRMS (positive ESI) Calcd. For $C_{15}H_{17}N_2O_4$ (M + H) 289.1188, Found: 289.1185.

2-(4-methyl-2-ethoxybenzamido)pyridine 1-oxide (3ca): Purified by analytical TLC on silica gel with CH_2Cl_2/acetone (3:1) as an eluent R_f = 0.32; white solid (30 mg, 74%), mp 162-163 ^oC (from 1:5 hexane/dichloromethane). ^1H NMR (400 MHz, $CDCl_3$) δ 12.17 (s, 1H), 8.66 (d, *J* = 8.4 Hz, 1H), 8.20-8.18 (m, 1H), 8.09-8.07 (d, *J* = 8.0 Hz, 1H), 7.26-7.23 (m, 1H), 6.91-6.88 (m, 1H), 6.83 (d, *J* = 8.0 Hz, 1H), 6.77 (s, 1H), 4.24 (q, *J* = 7.0 Hz, 2H), 2.32 (s, 3H), 1.63 (t, *J* = 7.0 Hz, 3H). ^{13}C NMR (100 MHz, $CDCl_3$) δ 164.0, 157.4, 145.5, 137.4, 132.5, 127.9, 122.0, 118.3, 117.8, 115.7, 113.0, 65.2, 21.9, 14.8. HRMS HRMS (positive ESI) Calcd. For $C_{15}H_{17}N_2O_3$ (M + H) 273.1239, Found: 273.1234.

2-(4-*tert*-butyl-2-ethoxybenzamido)pyridine 1-oxide (3da): Purified by analytical TLC on silica gel with CH_2Cl_2/acetone (3:1) as an eluent R_f = 0.42; white solid (39 mg, 83%), mp 127-128 ^oC (from 1:5 hexane/dichloromethane). ^1H NMR (400 MHz, $CDCl_3$) δ 12.27 (s, 1H), 8.75 (dd, J = 8.5 Hz, J = 1.7 Hz, 1H), 8.29 (dd, J = 6.5 Hz, J = 1.0 Hz, 1H), 8.20 (d, J = 8.3 Hz, 1H), 7.37-7.33 (m, 1H), 7.14 (dd, J = 8.4 Hz, J = 1.7 Hz, 1H), 7.05 (d, J = 1.6 Hz, 1H), 7.00-6.96(m, 1H), 4.37 (q, J = 7.0 Hz, 2H), 1.73 (t, J = 7.0 Hz, 3H). 1.36 (s, 9H). ^{13}C NMR (100 MHz, $CDCl_3$) δ 164.0, 158.6, 157.3, 145.5, 137.3, 132.3, 127.8, 118.4, 118.3, 117.8, 115.8, 109.4, 65.1, 35.4, 31.1, 14.8. HRMS (positive ESI) Calcd. For $C_{18}H_{23}N_2O_3$ (M + H) 315.1709, Found: 315.1705.

2-(3-ethoxy-[1,1'-biphenyl]-4-ylcarboxamido)pyridine 1-oxide (3ea): purified by analytical TLC on silica gel with CH_2Cl_2/acetone (3:1) as an eluent R_f = 0.36; white solid (31 mg, 62%), mp 174-175 ^oC (from 1:3 hexane/dichloromethane). ^1H NMR (400 MHz, $CDCl_3$) δ 12.32 (s, 1H), 8.76 (dd, J = 8.6 Hz, J = 1.7 Hz, 1H), 8.34 (d, J = 8.2 Hz, 1H), 8.31-8.29 (m, 1H), 7.64-7.62 (m, 2H), 7.50-7.46 (m, 2H), 7.43-7.41 (m, 1H), 7.36-7.32 (m, 2H), 7.25-7.24 (m, 1H), 7.02-6.98 (m, 1H), 4.43 (q, J = 7.0 Hz, 2H), 1.75 (t, J = 7.0 Hz, 3H). ^{13}C NMR (100 MHz, $CDCl_3$) δ 163.8, 157.7, 147.4, 145.4, 139.9, 137.3, 133.2, 129.0, 128.4, 127.9, 127.3, 119.9, 119.2, 118.5, 115.9, 111.1, 65.5, 14.8. HRMS (positive ESI) Calcd. For $C_{20}H_{19}N_2O_3$ (M + H) 335.1396, Found: 335.1392.

2-(2-ethoxy-4-fluorobenzamido)pyridine 1-oxide (3fa): purified by analytical TLC on silica gel with CH_2Cl_2/acetone (3:1) as an eluent R_f = 0.36; white solid (36 mg, 87%), mp 199-200 ^oC (chloromethane). ^1H NMR (400 MHz, $CDCl_3$) δ 12.16 (s, 1H), 8.71 (dd, J = 8.5 Hz, J = 1.6 Hz, 1H), 8.32-8.28 (m, 2H), 7.40-7.34 (m, 1H), 7.02-6.98 (m, 1H), 6.84-6.75 (m, 2H), 4.32 (q, J = 7.0 Hz, 2H), 1.73 (t, J = 7.0 Hz, 3H) . ^{13}C NMR (100 MHz, $CDCl_3$) δ 166.4 (d, J_{C-F} = 252.9 Hz), 163.0, 159.0 (d, J_{C-F} = 10.5 Hz), 145.3, 137.3, 134.8 (d, J_{C-F} = 11.0 Hz), 128.0, 118.6, 116.9 (d, J_{C-F} = 2.9 Hz), 115.8, 108.3 (d, J_{C-F} = 21.3 Hz), 100.4 (d, J_{C-F} = 26.1 Hz), 66.0, 14.6. ^{19}F NMR (376 MHz, $CDCl_3$) δ -102.8. HRMS (positive ESI) Calcd. For $C_{14}H_{14}FN_2O_3$ (M + H) 277.0988, Found: 277.0988.

2-(5-methyl-2-ethoxybenzamido)pyridine 1-oxide (3ga): purified by analytical TLC on silica gel with CH_2Cl_2/acetone (3:1) as an eluent R_f = 0.33; white solid (33 mg, 82%), mp 191-192 ^oC (from 1:5 hexane/dichloromethane). ^1H NMR (400 MHz, $CDCl_3$)

δ 12.32 (s, 1H), 8.74 (dd, *J* = 8.5 Hz, *J* = 1.8 Hz, 1H), 8.30-8.28(m, 1H), 8.08 (d, *J* = 2.2 Hz, 1H), 7.38-7.31 (m, 2H), 7.01-6.97 (m, 1H), 6.96 (d, *J* = 8.6 Hz, 1H), 4.31 (q, *J* = 7.0 Hz, 2H), 2.36 (s, 3H), 1.70 (t, *J* = 7.0 Hz, 3H). ^{13}C NMR (100 MHz, $CDCl_3$) δ 164.2, 155.5, 145.5, 137.3, 134.9, 132.7, 130.4, 127.8, 120.0, 118.4, 115.9, 112.4, 65.3, 20.4, 14.8. HRMS (positive ESI) Calcd. For $C_{15}H_{17}N_2O_3$ (M + H) 273.1239, Found: 273.1236.

2-(2-ethoxy-5-iodobenzamido)pyridine 1-oxide (3ha): purified by analytical TLC on silica gel with CH_2Cl_2/acetone (4:1) as an eluent R_f = 0.38; white solid (33 mg, 57%), mp 251-252 ^oC (dichloromethane). ^{1}H NMR (400 MHz, $CDCl_3$) δ 12.21 (s, 1H), 8.71 (d, *J* =8.4 Hz, 1H), 8.56-8.55 (m, 1H), 8.29 (d, *J* =6.0 Hz, 1H), 7.80-7.78 (m, 1H), 7.38-7.34 (m, 1H), 7.04-7.01 (m, 1H), 6.85-6.83 (m, 1H), 4.33 (q, *J* = 7.0 Hz, 2H), 1.71 (t, *J* = 6.9 Hz, 3H). ^{13}C NMR (100 MHz, $CDCl_3$) δ 162.4, 157.2, 142.7, 141.1, 137.5, 128.1, 122.5, 118.7, 115.9, 114.8, 83.2, 65.8, 14.6. HRMS (positive ESI) Calcd. For $C_{14}H_{14}IN_2O_3$ (M + H) 385.0049, Found: 385.0044.

2-(2-ethoxy-3,5-dimethoxybenzamido)pyridine 1-oxide (3ia): purified by analytical TLC on silica gel with CH_2Cl_2/acetone (4:1) as an eluent R_f = 0.45; white solid (36 mg, 75%); mp 116-117 ^oC (from 1:5 hexane/dichloromethane). ^{1}H NMR (400 MHz, $CDCl_3$) δ 12.60 (s, 1H), 8.68 (dd, *J* = 8.5 Hz, *J* = 1.3 Hz, 1H), 8.30 (d, *J* = 6.2, 1H), 7.37-7.33 (m, 1H), 7.28-7.27 (m, 1H), 7.02-6.98 (m, 1H), 6.71 (d, *J* = 3.0 Hz, 1H) 4.29 (q, *J* = 7.0 Hz, 2H), 3.89 (s, 3H), 3.86 (s, 3H), 1.53 (t, *J* = 7.0 Hz, 3H). ^{13}C NMR (100 MHz, $CDCl_3$) δ 163.9, 155.8, 153.8, 145.2, 141.7, 137.5, 127.6, 125.5, 118.7, 115.8, 105.7, 103.9, 70.6, 56.3, 55.7, 15.3. HRMS (positive ESI) Calcd. For $C_{16}H_{19}N_2O_5$ (M + H) 319.1294, Found: 319.1291.

2-(2-ethoxy-3,4,5-trimethoxybenzamido)pyridine 1-oxide (3ja): purified by analytical TLC on silica gel with CH_2Cl_2/acetone (3:1) as an eluent R_f = 0.45; white solid (37 mg, 71%); mp 98-99 ^oC (from 1:5 hexane/dichloromethane). ^{1}H NMR (400 MHz, $CDCl_3$) δ 12.58 (s, 1H), 8.69 (dd, *J* = 8.5 Hz, *J* = 1.7 Hz, 1H), 8.30 (dd, *J* = 6.5 Hz, *J* = 1.1 Hz, 1H), 7.53 (s, 1H), 7.39-7.34 (m, 1H), 7.03-6.99 (m, 1H), 4.37 (q, *J* = 7.0 Hz, 2H), 4.00 (s, 3H), 3.94 (s, 3H), 3.93 (s, 3H), 1.58 (t, *J* = 7.0 Hz, 3H). ^{13}C NMR (100 MHz, $CDCl_3$) δ 163.4, 149.3, 147.5, 146.8, 146.4, 145.2, 137.4, 127.7, 119.5, 118.6, 115.7, 107.9, 71.3, 61.4, 61.3, 56.1, 15.5. HRMS (positive ESI) Calcd. For $C_{17}H_{21}N_2O_6$ (M + H)

349.1400, Found: 349.1392.

2-(3-ethoxythiophene-2-carboxamido)pyridine 1-oxide (3ka): purified by analytical TLC on silica gel with CH_2Cl_2/acetone (4:1) as an eluent R_f = 0.27; white solid (25 mg, 63%); mp 131-132 ^oC (dichloromethane). ^1H NMR (400 MHz, $CDCl_3$) δ 11.64 (s, 1H), 8.56 (d, J = 8.4 Hz, 1H), 8.23 (d, J = 6.2 Hz, 1H), 7.47 (d, J = 5.5 Hz, 1H), 7.28 (t, J = 7.8 Hz, 1H), 6.93-6.89 (m, 1H), 6.85 (d, J = 5.5 Hz, 1H), 4.29 (q, J = 7.0 Hz, 2H), 1.59 (t, J = 7.0 Hz, 3H). ^{13}C NMR (100 MHz, $CDCl_3$) δ 160.2, 157.7, 145.2, 137.4, 131.7, 128.4, 118.1, 116.1, 115.8, 115.3, 68.5, 15.0. HRMS (positive ESI) Calcd. For $C_{12}H_{13}N_2O_3S$ (M + H) 265.0647, Found: 265.0643.

2-(2-(2-methoxyphenyl)-2-oxoethyl)pyridine 1-oxide (3ab): purified by analytical TLC on silica gel with CH_2Cl_2/acetone (4:1) as an eluent R_f = 0.23; white solid (28 mg, 76%); mp 133-134 ^oC (dichloromethane).^1H NMR (400 MHz, $CDCl_3$) δ 12.49 (s, 1H), 8.71(dd, J = 8.5 Hz, J = 1.6 Hz, 1H), 8.29-8.26 (m, 2H), 7.58-7.53 (m, 1H), 7.36 (t, J = 7.8 Hz, 1H), 7.15-7.11 (m, 1H), 7.07 (d, J = 8.4 Hz, 1H), 7.03-6.99 (m, 1H), 4.15 (s, 3H). ^{13}C NMR (100 MHz, $CDCl_3$) δ 163.7, 158.0, 145.6, 137.4, 134.4, 132.5, 128.2, 121.3, 120.5, 118.4, 115.6, 111.7, 56.3. HRMS (positive ESI) Calcd. For $C_{13}H_{13}N_2O_3$ (M + H) 245.0926, Found: 245.0926.

2-(2-butoxybenzamido)pyridine 1-oxide (3ac): purified by analytical TLC on silica gel with CH_2Cl_2/acetone (10:1) as an eluent R_f = 0.40; white solid (32 mg, 74%); mp 90-91 ^oC (from 1:5 hexane/dichloromethane). ^1H NMR (400 MHz, $CDCl_3$) δ 12.26 (s, 1H), 8.73 (dd, J = 8.5 Hz, J = 1.7 Hz, 1H), 8.30-8.27(m, 2H), 7.55-7.51(m, 1H), 7.37-7.33 (m, 1H), 7.13-7.07 (m, 2H), 7.01-6.97 (m, 1H), 4.29 (t, J = 7.1 Hz, 2H), 2.14-2.07 (m, 2H), 1.60-1.50 (m, 2H), 1.02 (t, J = 7.4 Hz, 3H). ^{13}C NMR (100 MHz, $CDCl_3$) δ 164.0, 157.6, 145.4, 137.4, 134.3, 132.7, 127.7, 121.0, 120.6, 118.5, 115.8, 112.5, 69.6, 30.6, 19.3, 13.8. HRMS (positive ESI) Calcd. For $C_{16}H_{19}N_2O_3$ (M + H) 287.1396, Found: 287.1401.

2-(2-(2,2,2-trifluoroethoxy)benzamido)pyridine 1-oxide (3ad): purified by analytical TLC on silica gel with CH_2Cl_2/acetone (4:1) as an eluent R_f = 0.45; white solid (40 mg, 85%); mp 152-153 ^oC (from 1:5 hexane/dichloromethane).^1H NMR (400 MHz, $CDCl_3$) δ 11.93 (s, 1H), 8.67 (d, J = 8.5 Hz, 1H), 8.30 (d, J = 6.3 Hz, 1H), 8.25 (d, J = 7.8 Hz, 1H), 7.60-7.56 (m, 1H), 7.36 (t, J = 8.0 Hz, 1H), 7.28-7.22 (m, 1H), 7.11 (d, J = 8.4 Hz, 1H),

7.04-7.00 (m,1H), 4.77-4.71 (m, 2H). ^{13}C NMR (100 MHz, $CDCl_3$) δ 163.1, 155.7, 145.1, 137.4, 134.3, 132.9, 127.8, 123.3, 123.0 (q, J_{C-F} = 277.6 Hz), 122.2, 118.7, 115.6, 113.4, 66.9 (q, J_{C-F} = 36.4 Hz). ^{19}F NMR (376 MHz, $CDCl_3$) δ -72.8. HRMS (positive ESI) Calcd. For $C_{14}H_{12}F_3N_2O_3$ (M + H) 313.0800, Found: 313.0799.

2-(2-phenethoxybenzamido)pyridine 1-oxide (3ae): purified by analytical TLC on silica gel with CH_2Cl_2/acetone (5:1) as an eluent R_f = 0.33; white solid (24 mg, 48%); mp 153-154 °C (from 1:5 hexane/dichloromethane). ^1H NMR (400 MHz, $CDCl_3$) δ 12.28 (s, 1H), 8.74 (dd, J = 8.5 Hz, J = 1.6 Hz, 1H), 8.32-8.26 (m, 2H), 7.52-7.48 (m, 1H), 7.39-7.27 (m, 5H), 7.24-7.20 (m, 1H), 7.13-7.10 (m, 1H), 7.03-6.99 (m, 2H), 4.42 (t, J = 7.8 Hz, 2H), 3.47 (t, J = 7.9 Hz, 2H). ^{13}C NMR (100 MHz, $CDCl_3$) δ 163.9, 157.3, 145.3, 137.5, 137.4, 134.3, 132.7, 129.2, 129.0, 128.5, 127.9, 126.6, 126.4, 121.3, 120.7, 118.6, 115.8, 112.6, 70.7, 35.5. HRMS (positive ESI) Calcd. For $C_{20}H_{19}N_2O_3$ (M + H) 335.1396, Found: 335.1392.

(*Z*)-2-(3-ethoxy-2-methylbut-2-enamido)pyridine 1-oxide (3la): purified by analytical TLC on silica gel with CH_2Cl_2/acetone (4:1) as an eluent R_f = 0.26; white solid (16 mg, 45%); mp 115-116 °C (dichloromethane). ^1H NMR (400 MHz, $CDCl_3$) δ 12.02 (s, 1H), 8.56 (d, J = 8.5 Hz, 1H), 8.15 (d, J = 6.2 Hz, 1H), 7.23-7.19 (m, 1H), 6.84 (t, J = 6.6 Hz, 1H), 4.15 (q, J = 7.0 Hz, 2H), 2.08 (s, 3H), 1.84 (s, 3H), 1.46 (t, J = 7.0 Hz, 3H), ^{13}C NMR (100 MHz, $CDCl_3$) δ 165.9, 159.8, 145.8, 137.2, 128.0, 117.5, 115.2, 108.3, 65.2, 15.9, 15.3, 13.8. HRMS (positive ESI) Calcd. For $C_{12}H_{17}N_2O_3$ (M + H) 237.1239, Found: 237.1238.

(*Z*)-2-(3-ethoxy-2-methylpent-2-enamido)pyridine 1-oxide (3ma): purified by analytical TLC on silica gel with CH_2Cl_2/acetone (4:1) as an eluent R_f = 0.33; white solid (10 mg, 27%); mp 124-125 °C (dichloromethane). ^1H NMR (400 MHz, $CDCl_3$) δ 12.05 (s, 1H), 8.57 (d, J = 8.1 Hz, 1H), 8.17 (s, 1H), 7.21 (s, 1H), 6.86 (s,1H), 4.13 (q, J = 6.9 Hz, 2H), 2.43 (q, J = 7.6 Hz, 2H), 1.86 (s, 3H), 1.46 (t, J = 6.9 Hz, 3H), 1.10 (t, J = 7.5 Hz, 3H). ^{13}C NMR (100 MHz, $CDCl_3$) δ 165.2, 163.6, 136.4, 127.1, 116.5, 114.2, 107.7, 63.9, 21.1, 14.3, 12.2, 10.6. HRMS (positive ESI) Calcd. For $C_{13}H_{19}N_2O_3$ (M + H) 251.1396, Found: 251.1394.

2-(2-ethoxycyclohex-1-enecarboxamido)pyridine 1-oxide (3na): purified by

analytical TLC on silica gel with CH_2Cl_2/acetone (4:1) as an eluent R_f = 0.25; white solid (16 mg, 40%); mp 111-112 °C (dichloromethane). ^{1}H NMR (400 MHz, $CDCl_3$) δ 11.97 (s, 1H), 8.56 (dd, J = 8.6 Hz, J = 1.8 Hz, 1H), 8.17 (dd, J = 6.5 Hz, J = 1.1 Hz, 1H), 7.25-7.20 (m, 1H), 6.86-6.82 (m, 1H), 4.15 (q, J = 7.0 Hz, 2H), 2.39-2.33 (m, 4H), 1.71-1.65 (m, 2H), 1.58-1.52 (m, 2H), 1.48 (t, J = 7.0 Hz, 3H). ^{13}C NMR (100 MHz, $CDCl_3$) δ 164.9, 161.0, 144.7, 136.2, 127.0, 116.5, 114.2, 108.5, 63.0, 25.3, 23.4, 21.6, 21.0, 14.3. HRMS (positive ESI) Calcd. For $C_{14}H_{19}N_2O_3$ (M + H) 263.1396, Found: 263.1393.

2-(2-methoxycyclohex-1-enecarboxamido)pyridine 1-oxide (3nb): purified by analytical TLC on silica gel with CH_2Cl_2/acetone (5:1) as an eluent R_f = 0.35; white solid (12 mg, 31%); mp 100-101 °C (dichloromethane). ^{1}H NMR (400 MHz, $CDCl_3$) δ 12.18 (s, 1H), 8.71 (s, 1H), 8.20-8.10 (m, 1H), 7.25-7.23 (m, 1H), 7.02-7.00 (m, 1H), 4.00 (s, 3H), 2.49-2.42 (m, 4H), 1.82-1.76 (m, 2H), 1.68-1.62 (m, 2H). ^{13}C NMR (100 MHz, $CDCl_3$) δ 165.7, 162.4, 138.1, 128.8, 116.9, 114.7, 109.5, 55.2, 25.5, 24.2, 22.5, 22.0. HRMS (positive ESI) Calcd. For $C_{13}H_{17}N_2O_3$ (M + H) 249.1239, Found: 249.1237.

2-(6-ethoxy-3,4-dihydro-2H-pyran-5-carboxamido)pyridine 1-oxide (3oa): purified by analytical TLC on silica gel with CH_2Cl_2/acetone (5:1) as an eluent R_f = 0.38; white solid (13 mg, 32%); mp 131-132 °C (dichloromethane). ^{1}H NMR (400 MHz, $CDCl_3$) δ 10.76 (s, 1H), 8.38 (d, J = 7.8 Hz, 1H), 8.17 (s, 1H), 7.27-7.26 (m, 1H), 6.98-6.95 (m, 1H), 4.21-4.16 (m, 2H), 3.64-3.56 (m, 2H), 2.07 (q, J = 7.7 Hz, 2H), 1.63-1.56 (m, 2H), 1.24 (t, J = 7.2 Hz, 3H). ^{13}C NMR (100 MHz, $CDCl_3$) δ 169.9, 167.6, 144.3, 137.4, 128.6, 119.0, 115.1, 62.0, 53.9, 29.9, 26.6, 14.1. HRMS (positive ESI) Calcd. For $C_{13}H_{17}N_2O_4$ (M + H) 265.1188, Found: 265.1185.

NMR and Mass Spectra

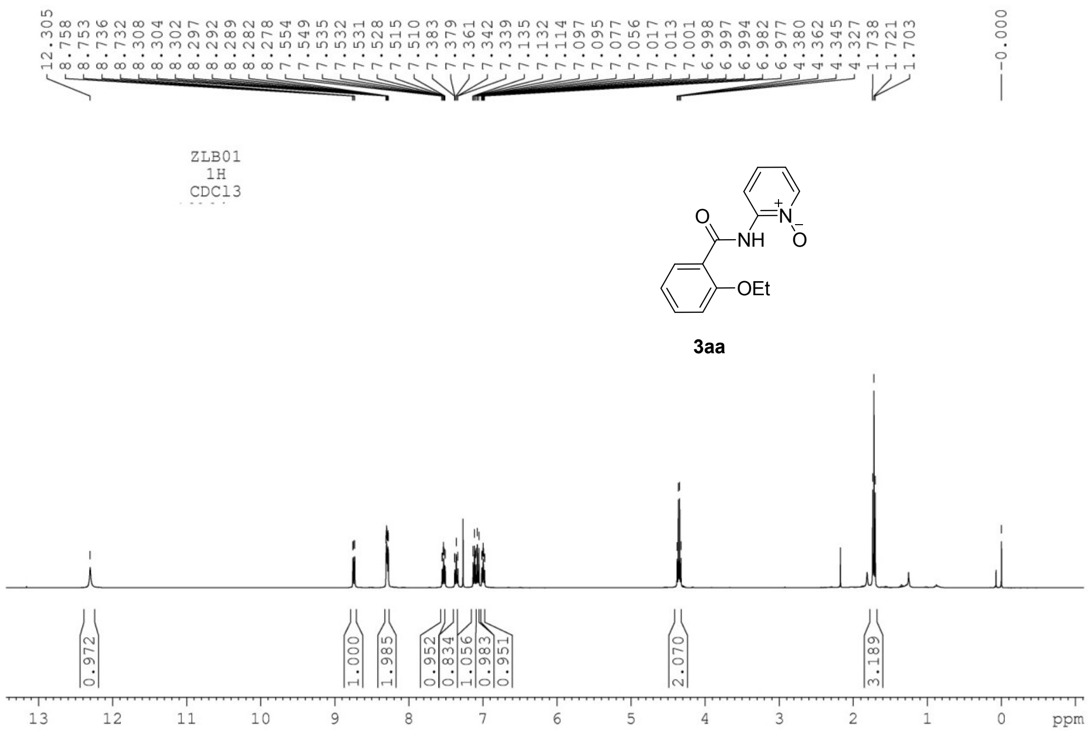

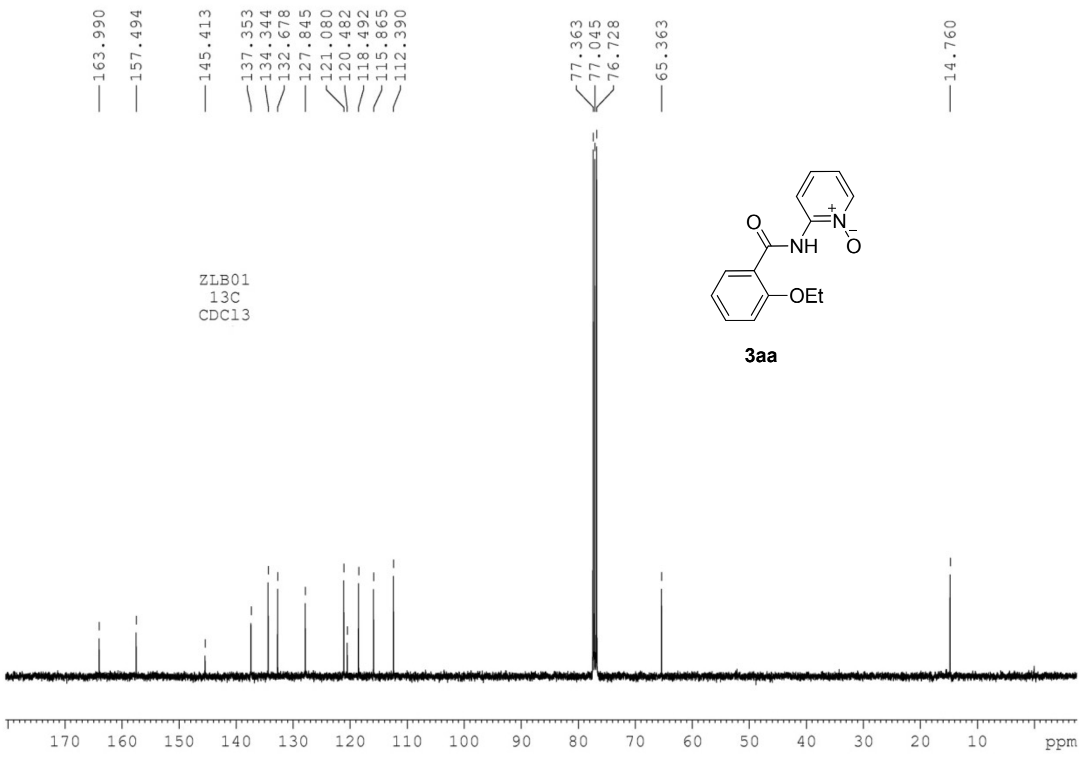

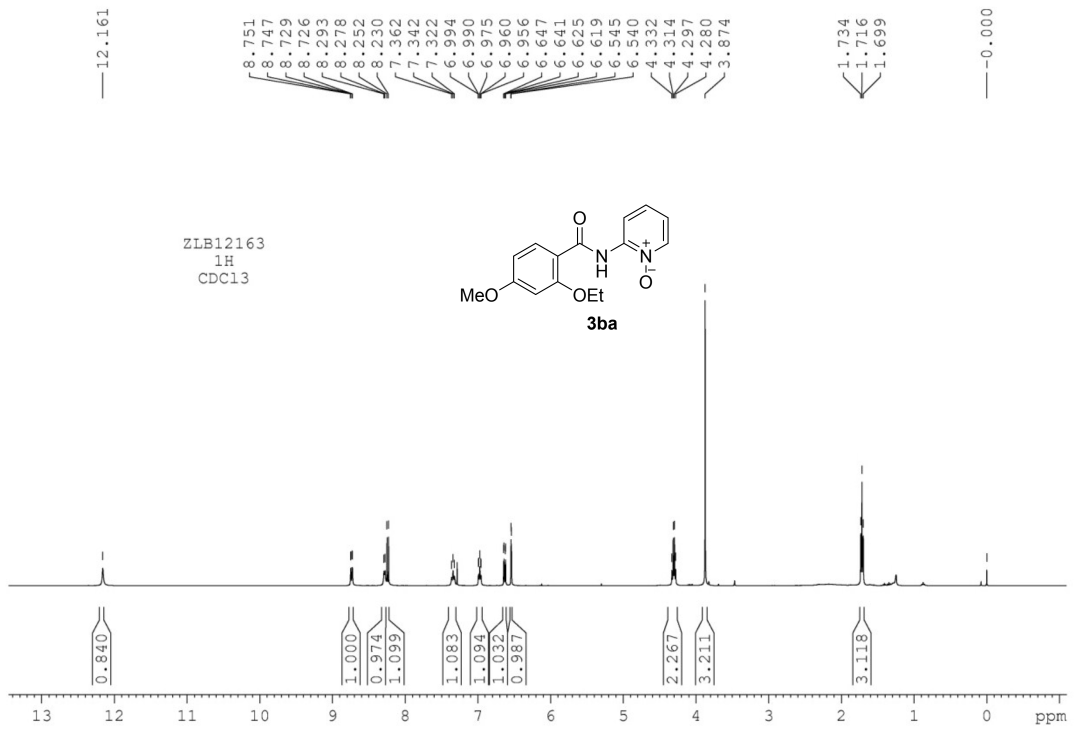
12.161
8.751
8.747
8.729
8.726
8.293
8.278
8.252
8.230
7.362
7.342
7.322
6.994
6.990
6.975
6.960
6.956
6.647
6.641
6.625
6.619
6.545
6.540
4.332
4.314
4.297
4.280
3.874
1.734
1.716
1.699
0.000
ZLB12163
1H
CDCl3
MeO
OEt
O
N
H
N
+
O
−
3ba
0.840
1.000
0.974
1.099
1.083
1.094
1.032
0.987
2.267
3.211
3.118
13 12 11 10 9 8 7 6 5 4 3 2 1 0 ppm

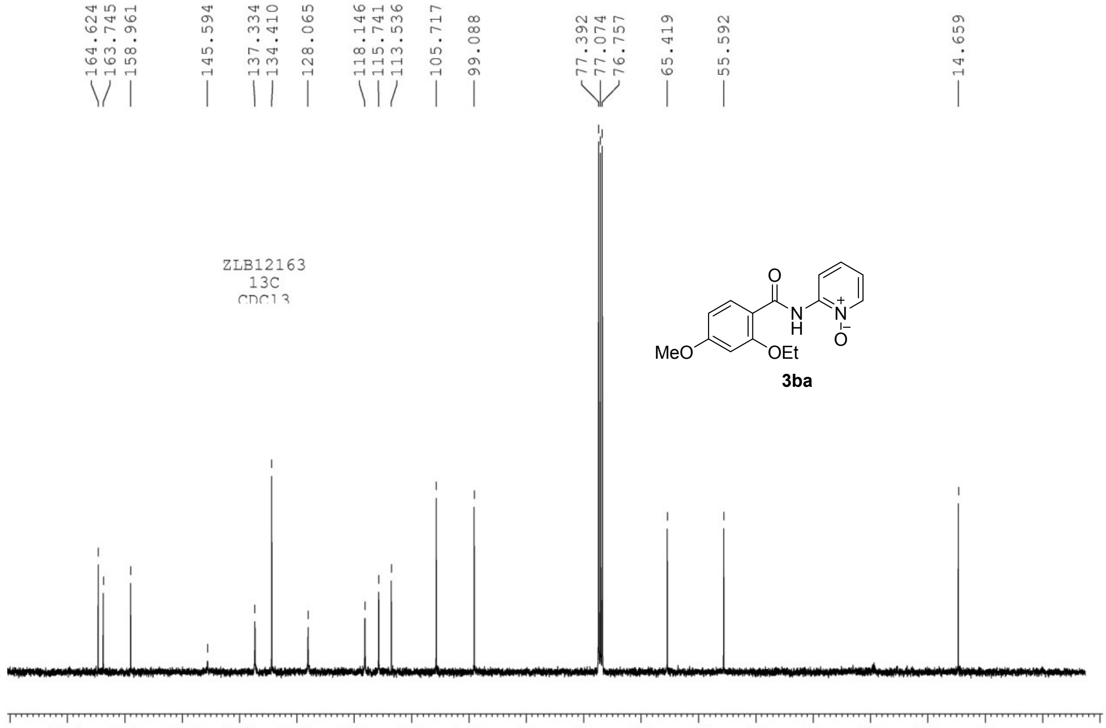
164.624
163.745
158.961
145.594
137.334
134.410
128.065
118.146
115.741
113.536
105.717
99.088
77.392
77.074
76.757
65.419
55.592
14.659
ZLB12163
13C
CDCl3
MeO
OEt
O
N
H
N
+
O
−
3ba
170 160 150 140 130 120 110 100 90 80 70 60 50 40 30 20 10 0 ppm

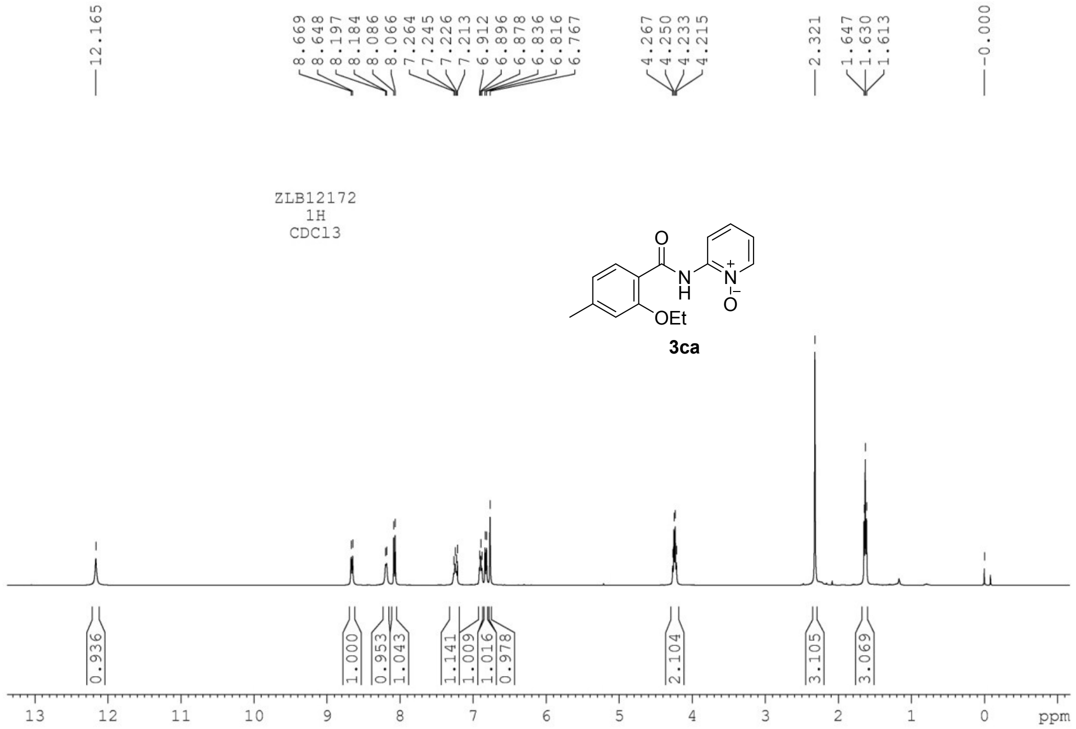
12.165
8.669
8.648
8.197
8.184
8.086
8.066
7.264
7.245
7.226
7.213
6.912
6.896
6.878
6.836
6.816
6.767
4.267
4.250
4.233
4.215
2.321
1.647
1.630
1.613
0.000
ZLB12172
1H
CDCl3
O
N
H
N
+
O
–
OEt
3ca
0.936
1.000
0.953
1.043
1.141
1.009
1.016
0.978
2.104
3.105
3.069
13
12
11
10
9
8
7
6
5
4
3
2
1
0
ppm

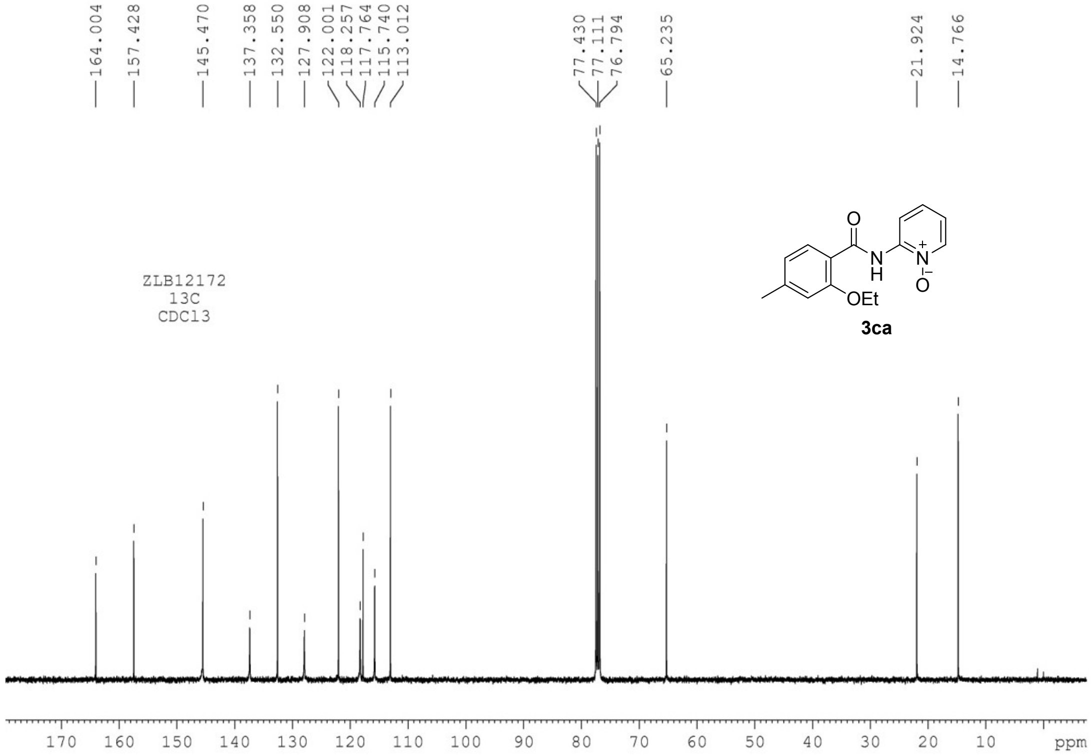
164.004
157.428
145.470
137.358
132.550
127.908
122.001
118.257
117.764
115.740
113.012
77.430
77.111
76.794
65.235
21.924
14.766
ZLB12172
13C
CDCl3
O
N
H
N
+
O
–
OEt
3ca
170
160
150
140
130
120
110
100
90
80
70
60
50
40
30
20
10
ppm

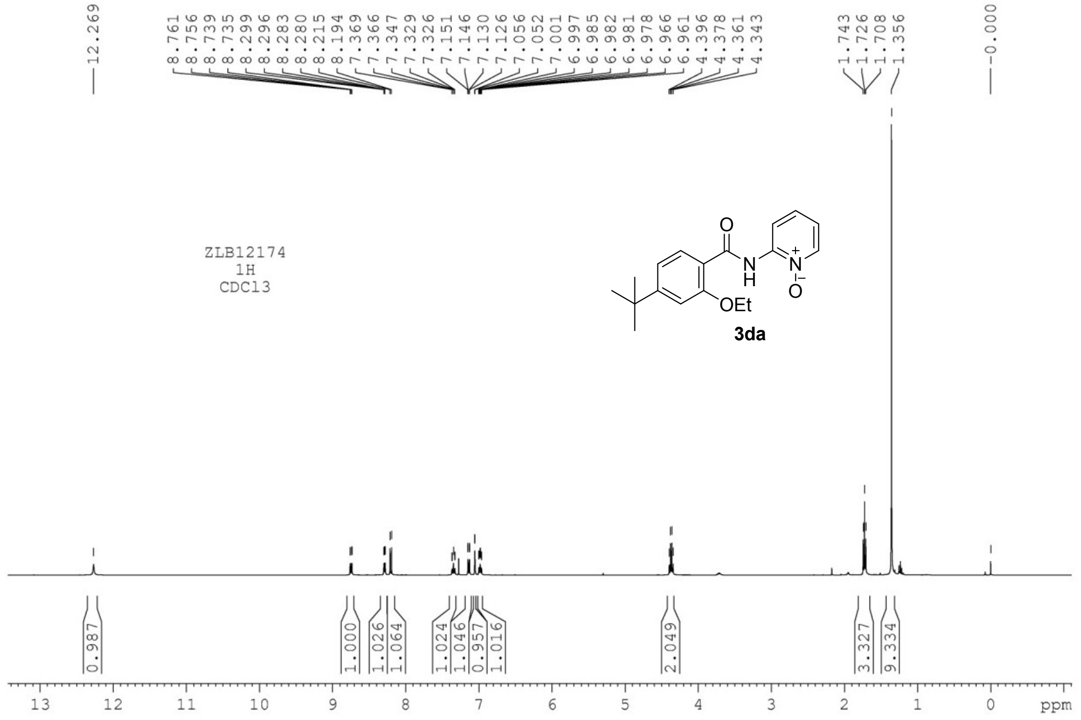
12.269
8.761
8.756
8.739
8.735
8.299
8.296
8.283
8.280
8.215
8.194
7.369
7.366
7.347
7.329
7.326
7.151
7.146
7.130
7.126
7.056
7.052
7.001
6.997
6.985
6.982
6.981
6.978
6.966
6.961
4.396
4.378
4.361
4.343
1.743
1.726
1.708
1.356
-0.000
ZLB12174
1H
CDCl3
O
N
H
N
+
O
–
OEt
3da
0.987
1.000
1.026
1.064
1.024
1.046
0.957
1.016
2.049
3.327
9.334
13
12
11
10
9
8
7
6
5
4
3
2
1
0
ppm

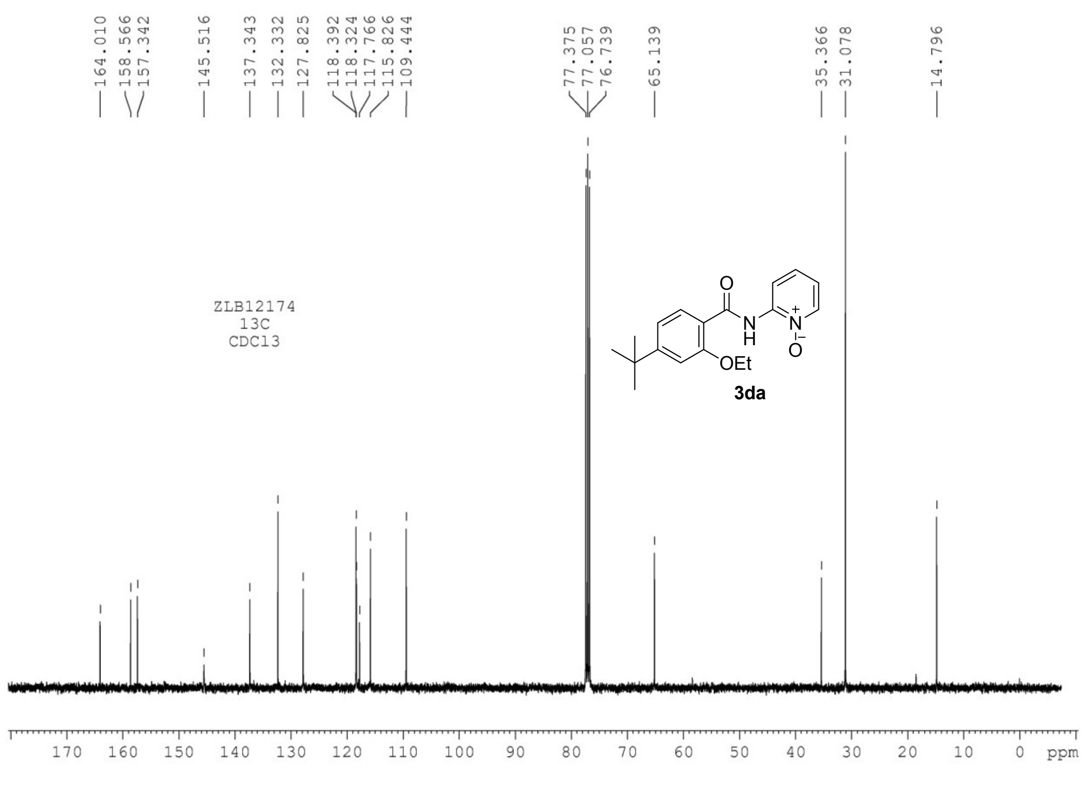
164.010
158.566
157.342
145.516
137.343
132.332
127.825
118.392
118.324
117.766
115.826
109.444
77.375
77.057
76.739
65.139
35.366
31.078
14.796
ZLB12174
13C
CDCl3
O
N
H
N
+
O
–
OEt
3da
170
160
150
140
130
120
110
100
90
80
70
60
50
40
30
20
10
0
ppm

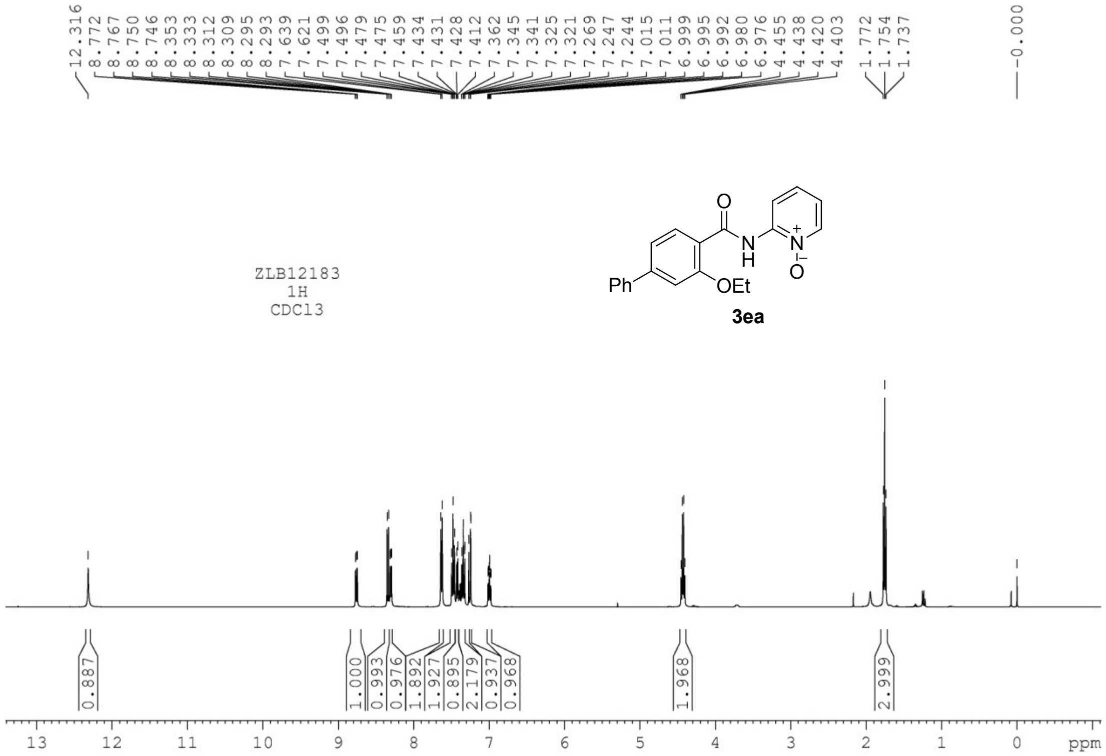
12.316
8.772
8.767
8.750
8.746
8.353
8.333
8.312
8.309
8.295
8.293
7.639
7.621
7.499
7.496
7.479
7.475
7.459
7.434
7.431
7.428
7.412
7.362
7.345
7.341
7.325
7.321
7.269
7.247
7.244
7.015
7.011
6.999
6.995
6.992
6.980
6.976
4.455
4.438
4.420
4.403
1.772
1.754
1.737
0.000
ZLB12183
1H
CDCl3
Ph
OEt
O
N
H
N+
O–
3ea
0.887
1.000
0.993
0.976
1.892
1.927
0.895
2.179
0.937
0.968
1.968
2.999
13 12 11 10 9 8 7 6 5 4 3 2 1 0 ppm

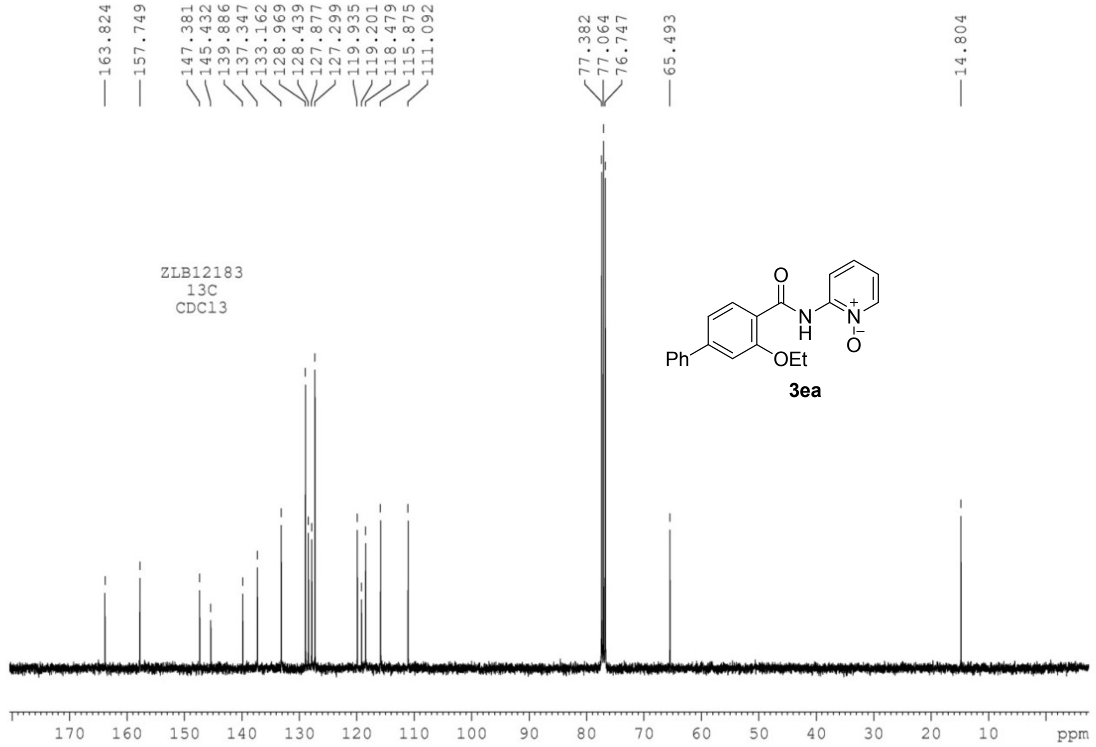
163.824
157.749
147.381
145.432
139.886
137.347
133.162
128.969
128.439
127.877
127.299
119.935
119.201
118.479
115.875
111.092
77.382
77.064
76.747
65.493
14.804
ZLB12183
13C
CDCl3
Ph
OEt
O
N
H
N+
O–
3ea
170 160 150 140 130 120 110 100 90 80 70 60 50 40 30 20 10 ppm

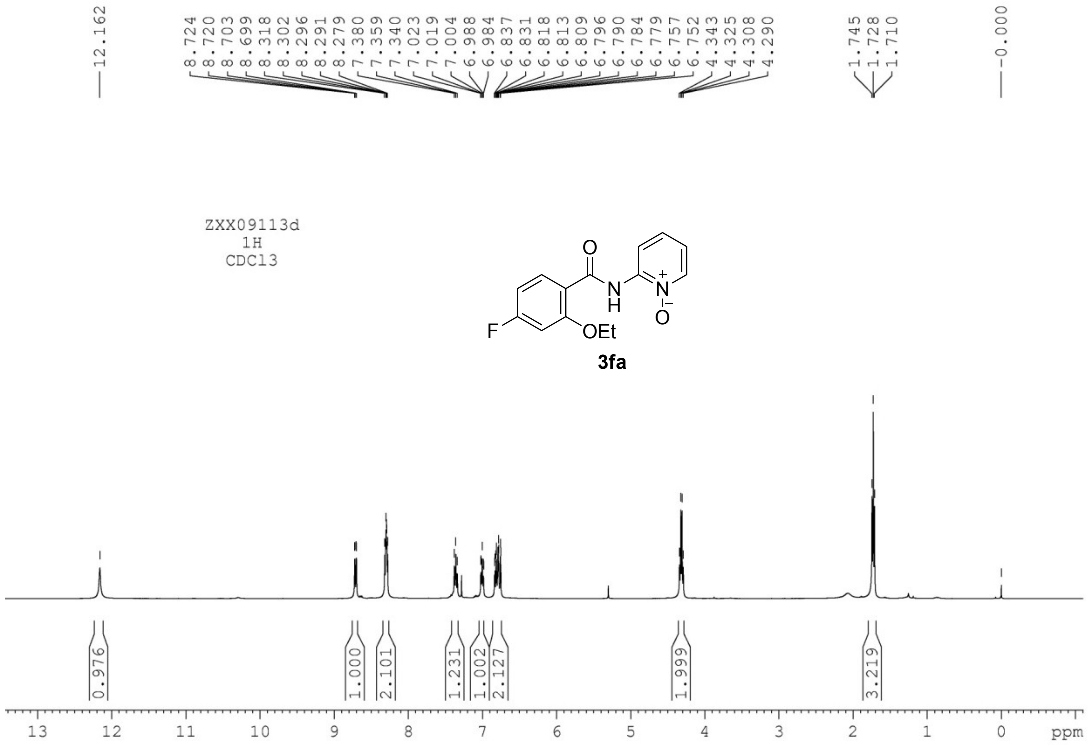

12.162
8.724
8.720
8.703
8.699
8.318
8.302
8.296
8.291
8.279
7.380
7.359
7.340
7.023
7.019
7.004
6.988
6.984
6.837
6.831
6.818
6.813
6.809
6.796
6.790
6.784
6.779
6.757
6.752
4.343
4.325
4.308
4.290
1.745
1.728
1.710
0.000
ZXX09113d
1H
CDCl3
O
N
H
N
+
O
−
F
OEt
3fa
0.976
1.000
2.101
1.231
1.002
2.127
1.999
3.219
13
12
11
10
9
8
7
6
5
4
3
2
1
0
ppm

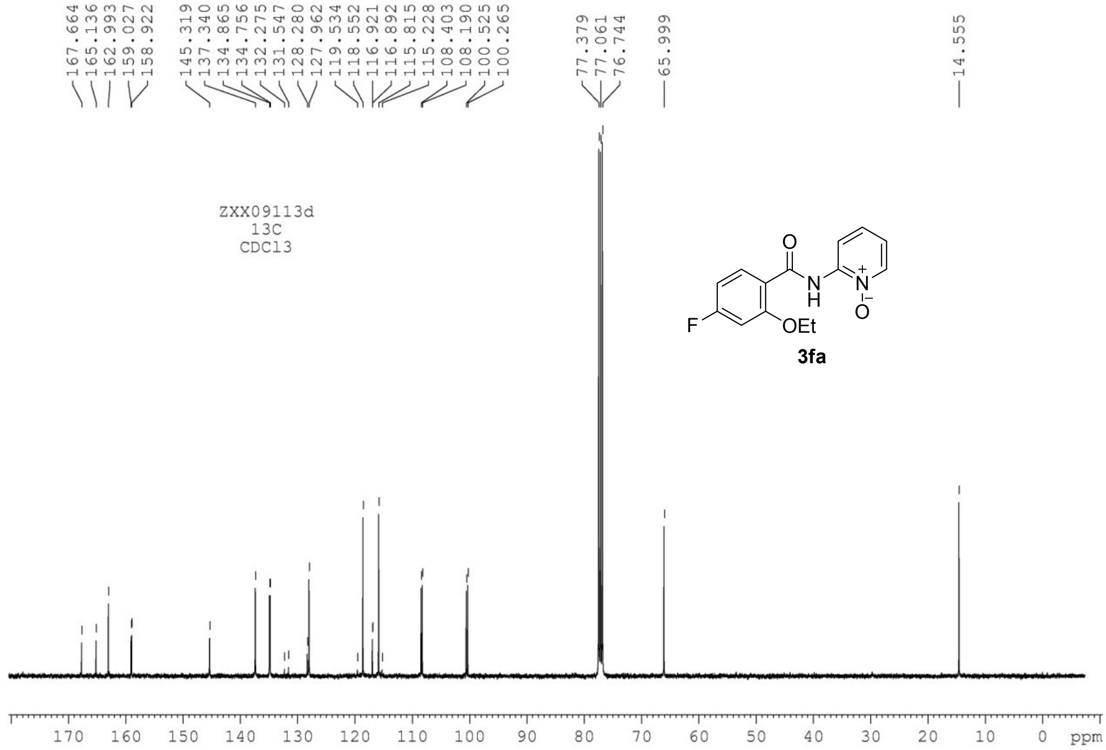

167.664
165.136
162.993
159.027
158.922
145.319
137.340
134.865
134.756
132.275
131.547
128.280
127.962
119.534
118.552
116.921
116.892
115.815
115.228
108.403
108.190
100.525
100.265
77.379
77.061
76.744
65.999
14.555
ZXX09113d
13C
CDCl3
O
N
H
N
+
O
−
F
OEt
3fa
170
160
150
140
130
120
110
100
90
80
70
60
50
40
30
20
10
0
ppm

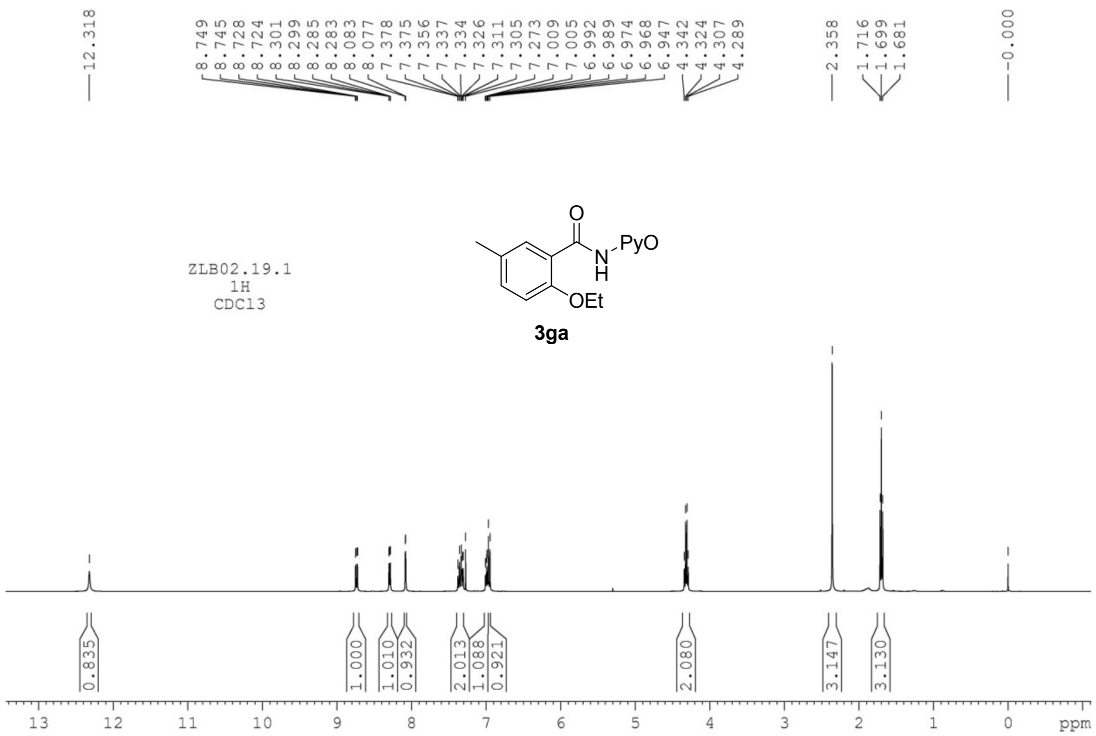
12.318
8.749
8.745
8.728
8.724
8.301
8.299
8.285
8.283
8.083
8.077
7.378
7.375
7.356
7.337
7.334
7.326
7.311
7.305
7.273
7.009
7.005
6.992
6.989
6.974
6.968
6.947
4.342
4.324
4.307
4.289
2.358
1.716
1.699
1.681
0.000
ZLB02.19.1
1H
CDCl3
O
N
PyO
H
OEt
3ga
0.835
1.000
1.010
0.932
2.013
1.088
0.921
2.080
3.147
3.130
13
12
11
10
9
8
7
6
5
4
3
2
1
0
ppm

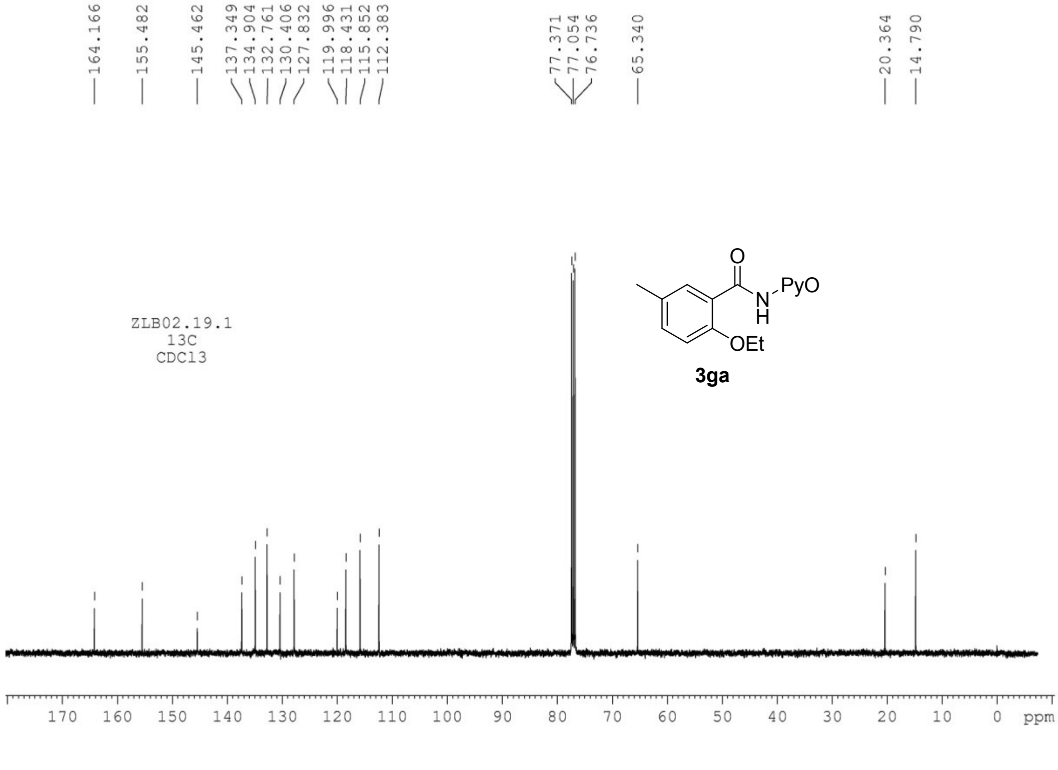
164.166
155.482
145.462
137.349
134.904
132.761
130.406
127.832
119.996
118.431
115.852
112.383
77.371
77.054
76.736
65.340
20.364
14.790
ZLB02.19.1
13C
CDCl3
O
N
PyO
H
OEt
3ga
170
160
150
140
130
120
110
100
90
80
70
60
50
40
30
20
10
0
ppm

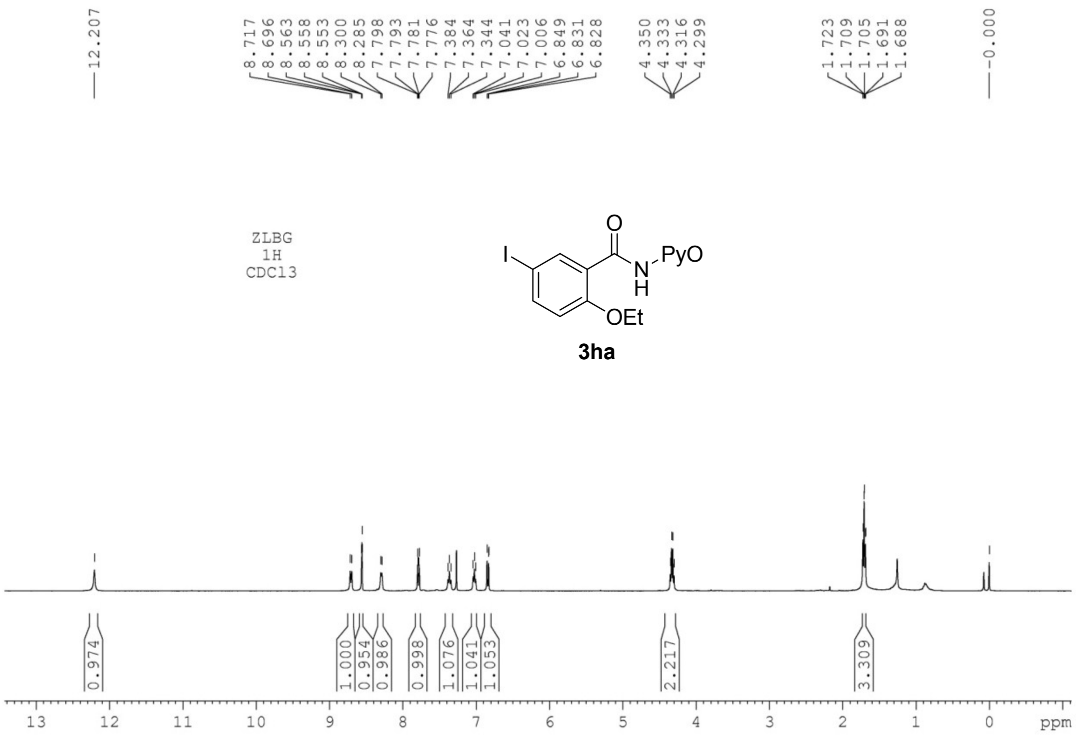
12.207
8.717 8.696 8.563 8.558 8.553 8.300 8.285 7.798 7.793 7.781 7.776 7.384 7.364 7.344 7.041 7.023 7.006 6.849 6.831 6.828
4.350 4.333 4.316 4.299
1.723 1.709 1.705 1.691 1.688
-0.000
ZLBG
1H
CDCl3
O
I
N
PyO
H
OEt
3ha
0.974
1.000 0.954 0.986 0.998 1.076 1.041 1.053
2.217
3.309
13 12 11 10 9 8 7 6 5 4 3 2 1 0 ppm

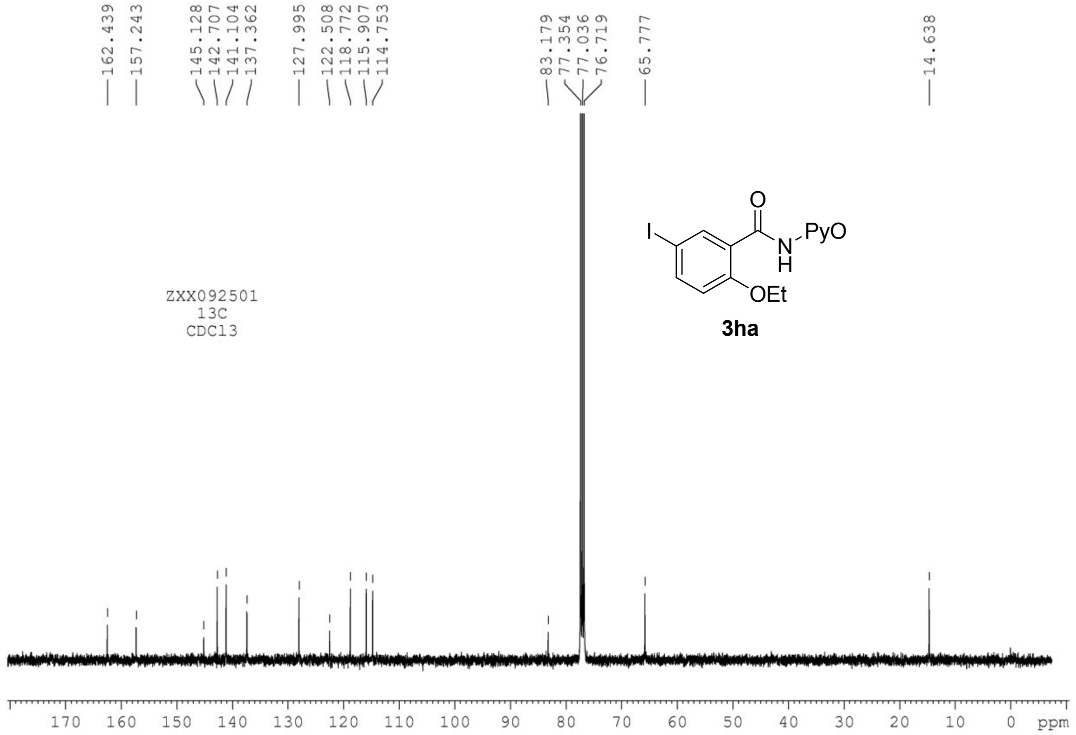
162.439 157.243
145.128 142.707 141.104 137.362
127.995 122.508 118.772 115.907 114.753
83.179 77.354 77.036 76.719
65.777
14.638
ZXX092501
13C
CDCl3
O
I
N
PyO
H
OEt
3ha
170 160 150 140 130 120 110 100 90 80 70 60 50 40 30 20 10 0 ppm

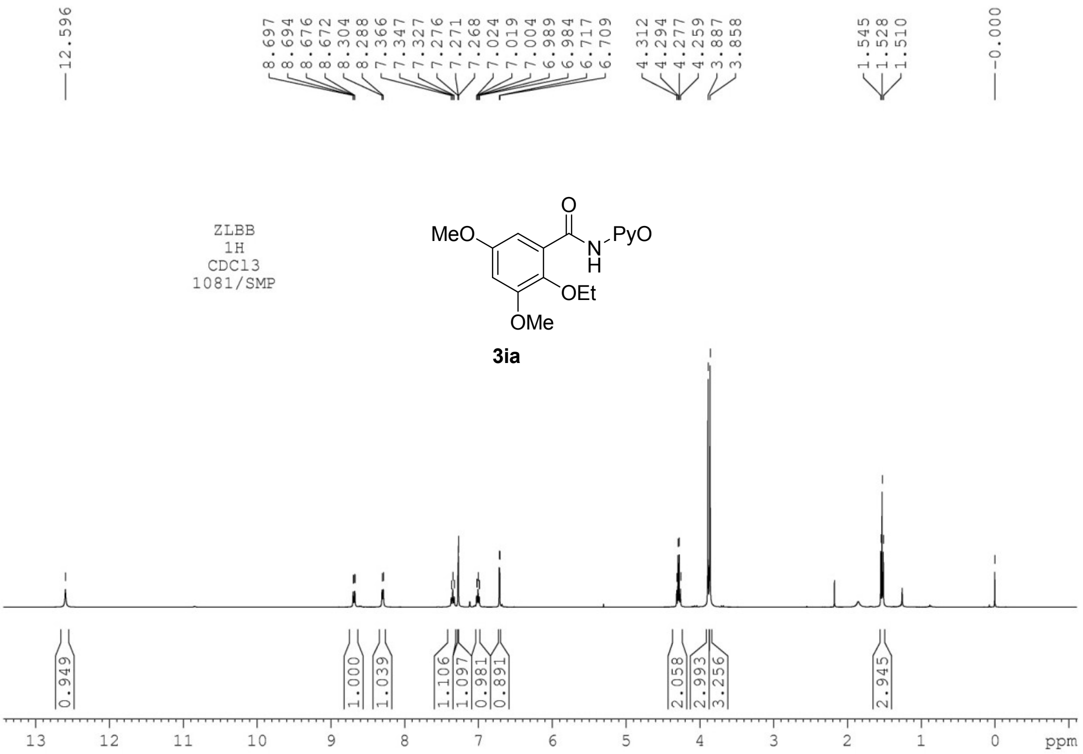

12.596
8.697
8.694
8.676
8.672
8.304
8.288
7.366
7.347
7.327
7.276
7.271
7.268
7.024
7.019
7.004
6.989
6.984
6.717
6.709
4.312
4.294
4.277
4.259
3.887
3.858
1.545
1.528
1.510
0.000
ZLBB
1H
CDCl3
1081/SMP
MeO
O
N
H
PyO
OEt
OMe
3ia
0.949
1.000
1.039
1.106
1.097
0.981
0.891
2.058
2.993
3.256
2.945
13 12 11 10 9 8 7 6 5 4 3 2 1 0 ppm

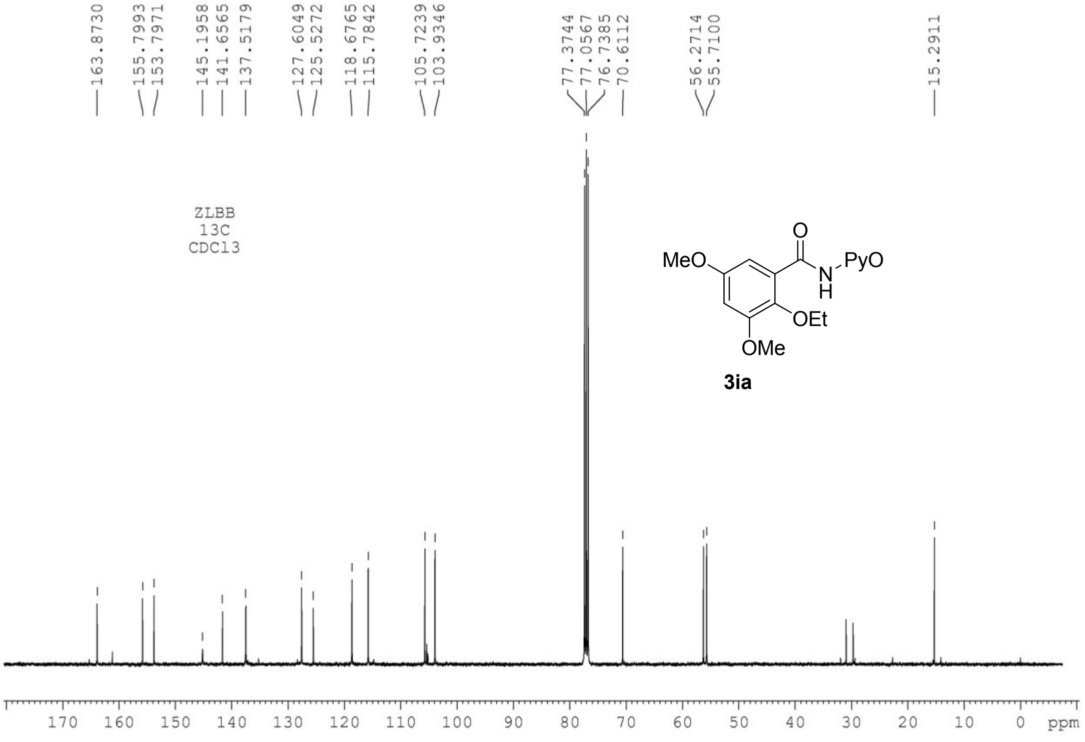

163.8730
155.7993
153.7971
145.1958
141.6565
137.5179
127.6049
125.5272
118.6765
115.7842
105.7239
103.9346
77.3744
77.0567
76.7385
70.6112
56.2714
55.7100
15.2911
ZLBB
13C
CDCl3
MeO
O
N
H
PyO
OEt
OMe
3ia
170 160 150 140 130 120 110 100 90 80 70 60 50 40 30 20 10 0 ppm

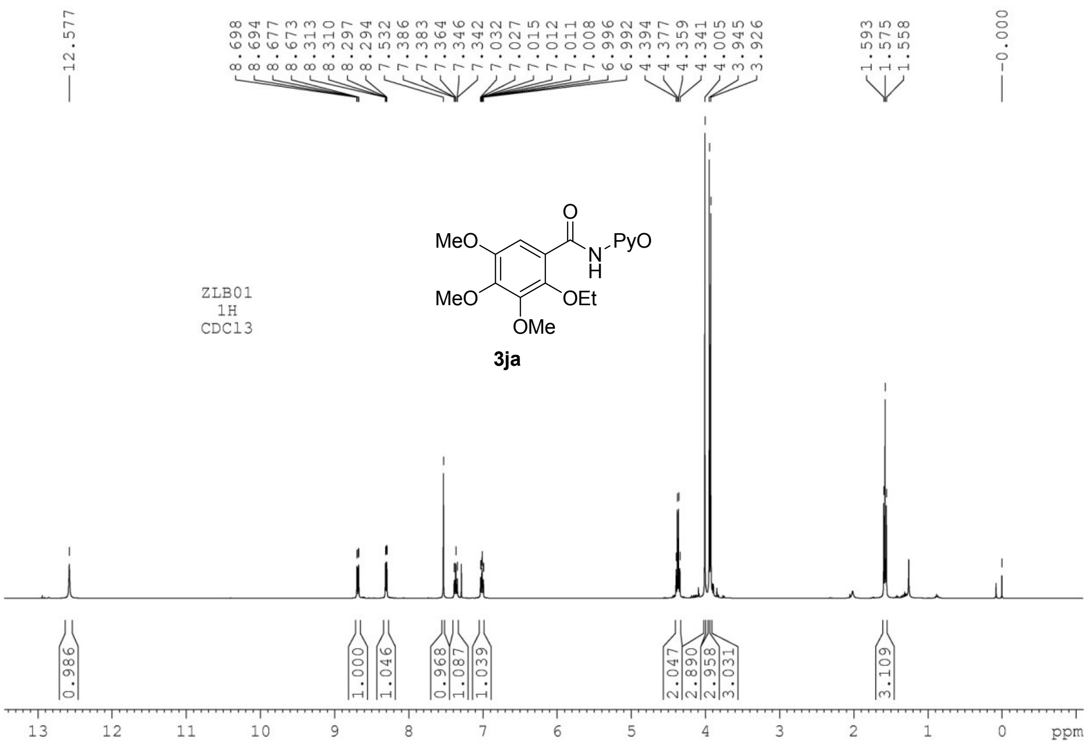
12.577
8.698
8.694
8.677
8.673
8.313
8.310
8.297
8.294
7.532
7.386
7.383
7.364
7.346
7.342
7.032
7.027
7.015
7.012
7.011
7.008
6.996
6.992
4.394
4.377
4.359
4.341
4.005
3.945
3.926
1.593
1.575
1.558
-0.000
ZLB01
1H
CDCl3
MeO
MeO
OMe
OEt
O
N
H
PyO
3ja
0.986
1.000
1.046
0.968
1.087
1.039
2.047
2.890
2.958
3.031
3.109
13 12 11 10 9 8 7 6 5 4 3 2 1 0 ppm

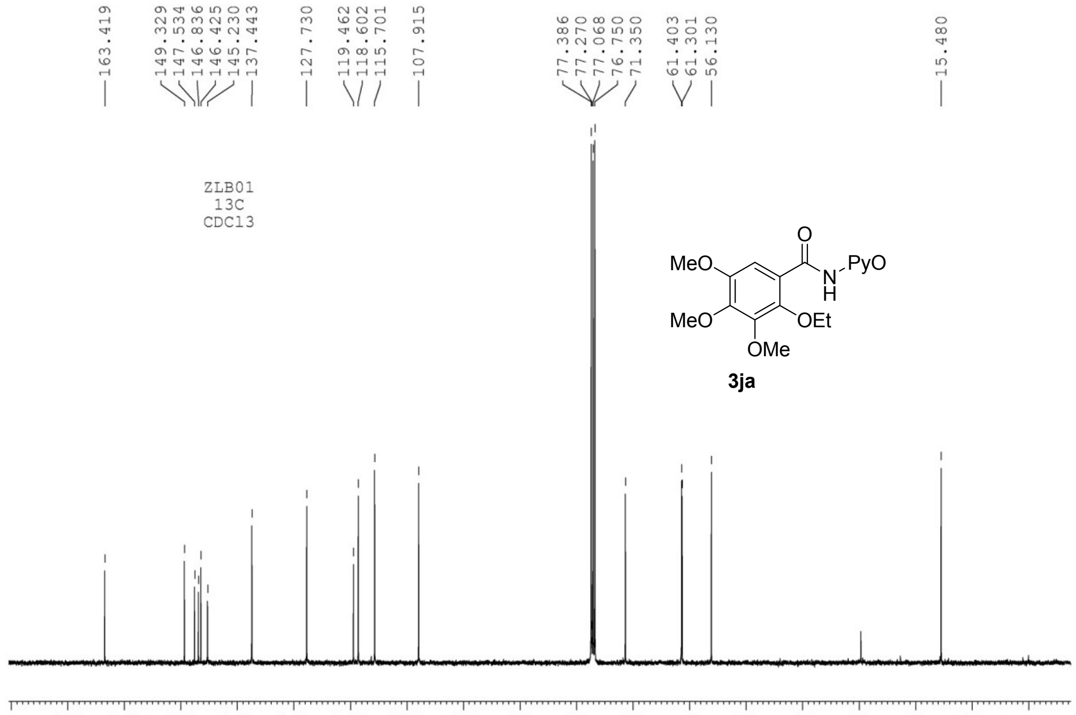
163.419
149.329
147.534
146.836
146.425
145.230
137.443
127.730
119.462
118.602
115.701
107.915
77.386
77.270
77.068
76.750
71.350
61.403
61.301
56.130
15.480
ZLB01
13C
CDCl3
MeO
MeO
OMe
OEt
O
N
H
PyO
3ja
170 160 150 140 130 120 110 100 90 80 70 60 50 40 30 20 10 ppm

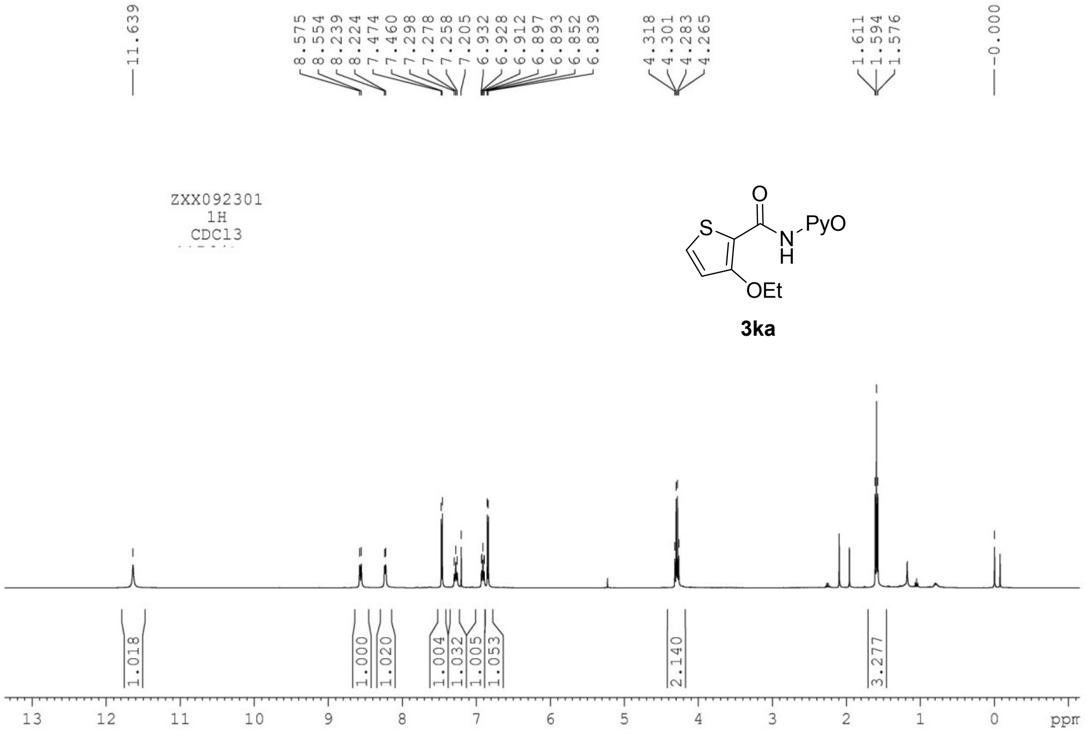
11.639
8.575
8.554
8.239
8.224
7.474
7.460
7.298
7.278
7.258
7.205
6.932
6.928
6.912
6.897
6.893
6.852
6.839
4.318
4.301
4.283
4.265
1.611
1.594
1.576
-0.000
ZXX092301
1H
CDCl3
O
S
N
PyO
H
OEt
3ka
1.018
1.000
1.020
1.004
1.032
1.005
1.053
2.140
3.277
13
12
11
10
9
8
7
6
5
4
3
2
1
0
ppm

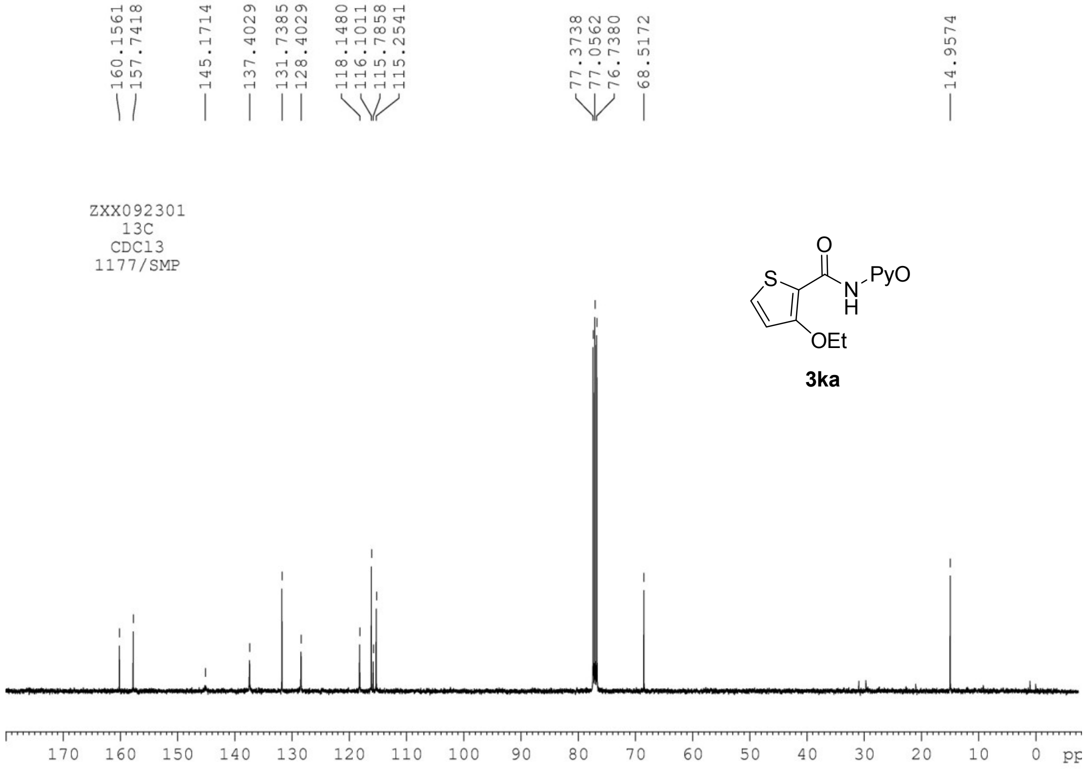
160.1561
157.7418
145.1714
137.4029
131.7385
128.4029
118.1480
116.1011
115.7858
115.2541
77.3738
77.0562
76.7380
68.5172
14.9574
ZXX092301
13C
CDCl3
1177/SMP
O
S
N
PyO
H
OEt
3ka
170
160
150
140
130
120
110
100
90
80
70
60
50
40
30
20
10
0
pp

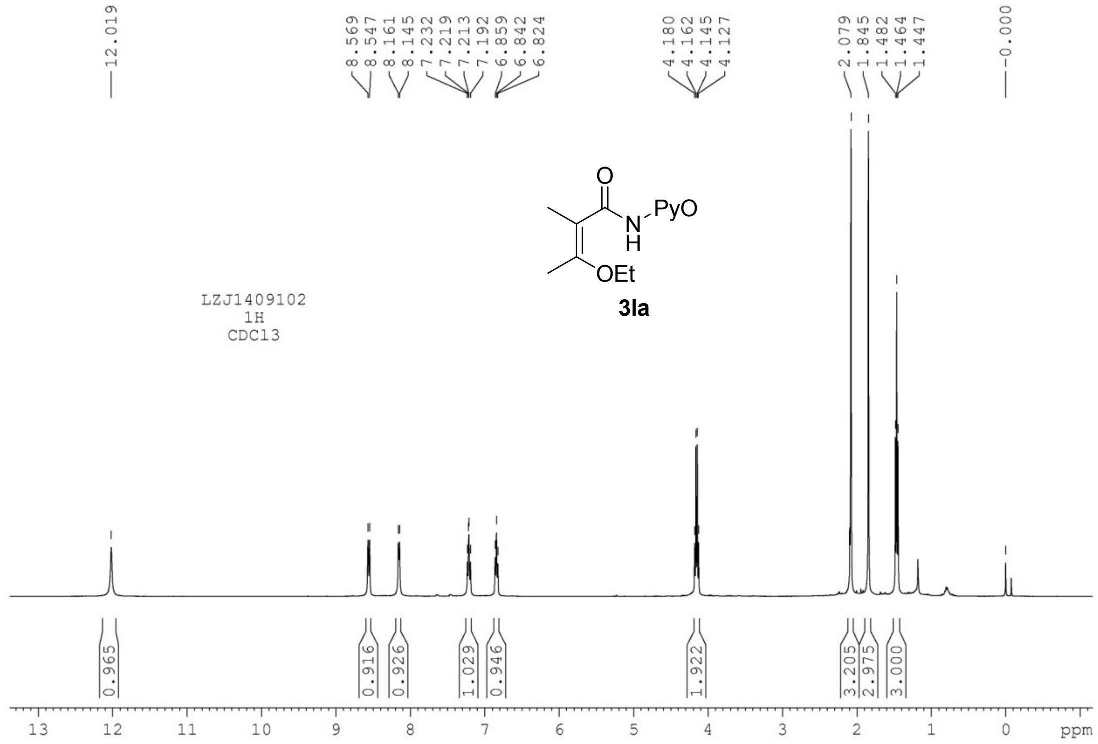
12.019
8.569
8.547
8.161
8.145
7.232
7.219
7.213
7.192
6.859
6.842
6.824
4.180
4.162
4.145
4.127
2.079
1.845
1.482
1.464
1.447
-0.000
O
N
PyO
H
OEt
3la
LZJ1409102
1H
CDCl3
0.965
0.916
0.926
1.029
0.946
1.922
3.205
2.975
3.000
13
12
11
10
9
8
7
6
5
4
3
2
1
0
ppm

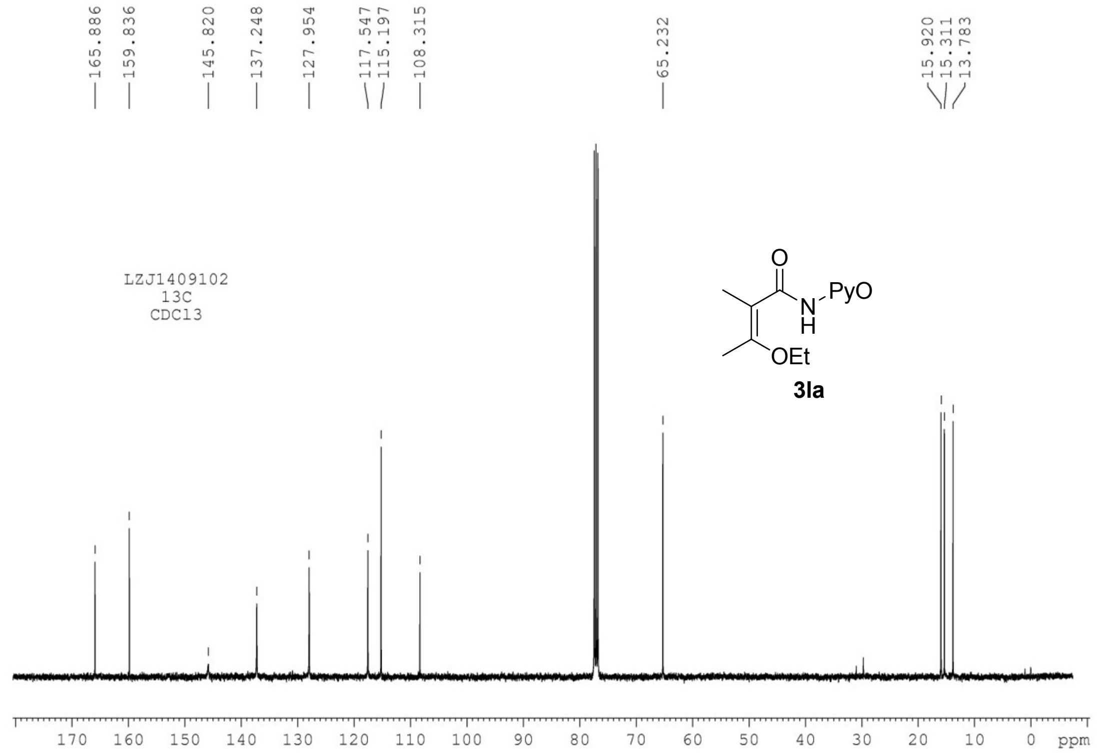
165.886
159.836
145.820
137.248
127.954
117.547
115.197
108.315
65.232
15.920
15.311
13.783
LZJ1409102
13C
CDCl3
O
N
PyO
H
OEt
3la
170
160
150
140
130
120
110
100
90
80
70
60
50
40
30
20
10
0
ppm

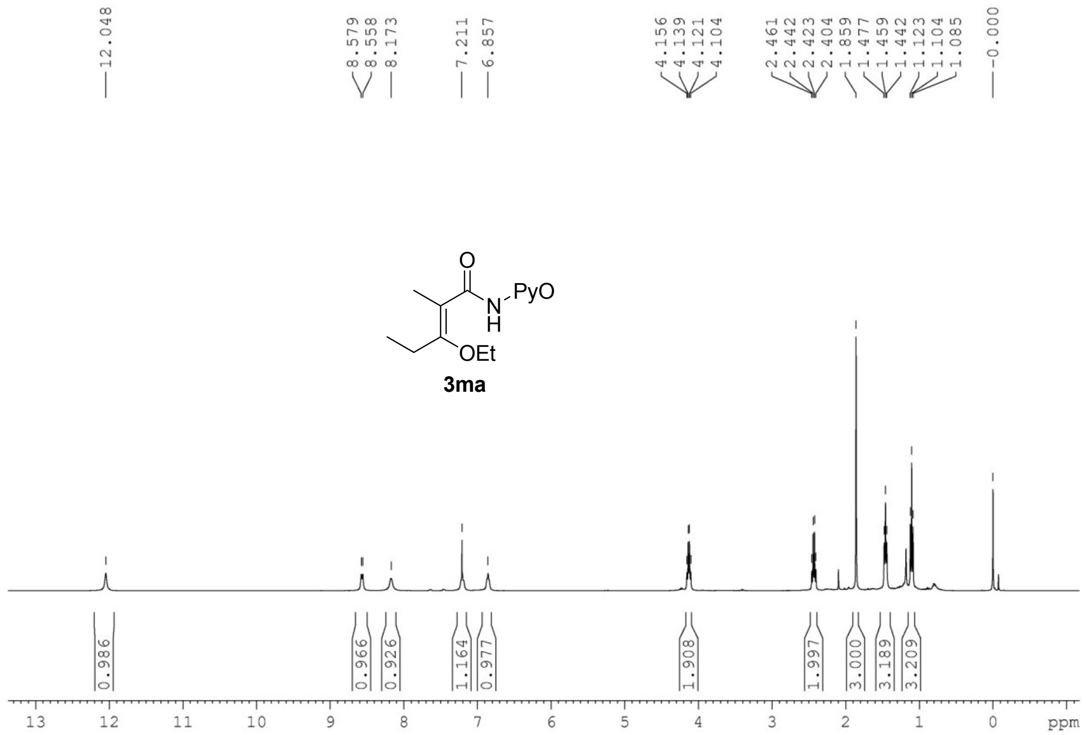
12.048
8.579
8.558
8.173
7.211
6.857
4.156
4.139
4.121
4.104
2.461
2.442
2.423
2.404
1.859
1.477
1.459
1.442
1.123
1.104
1.085
0.000
O
N
H
PyO
OEt
3ma
0.986
0.966
0.926
1.164
0.977
1.908
1.997
3.000
3.189
3.209
13 12 11 10 9 8 7 6 5 4 3 2 1 0 ppm

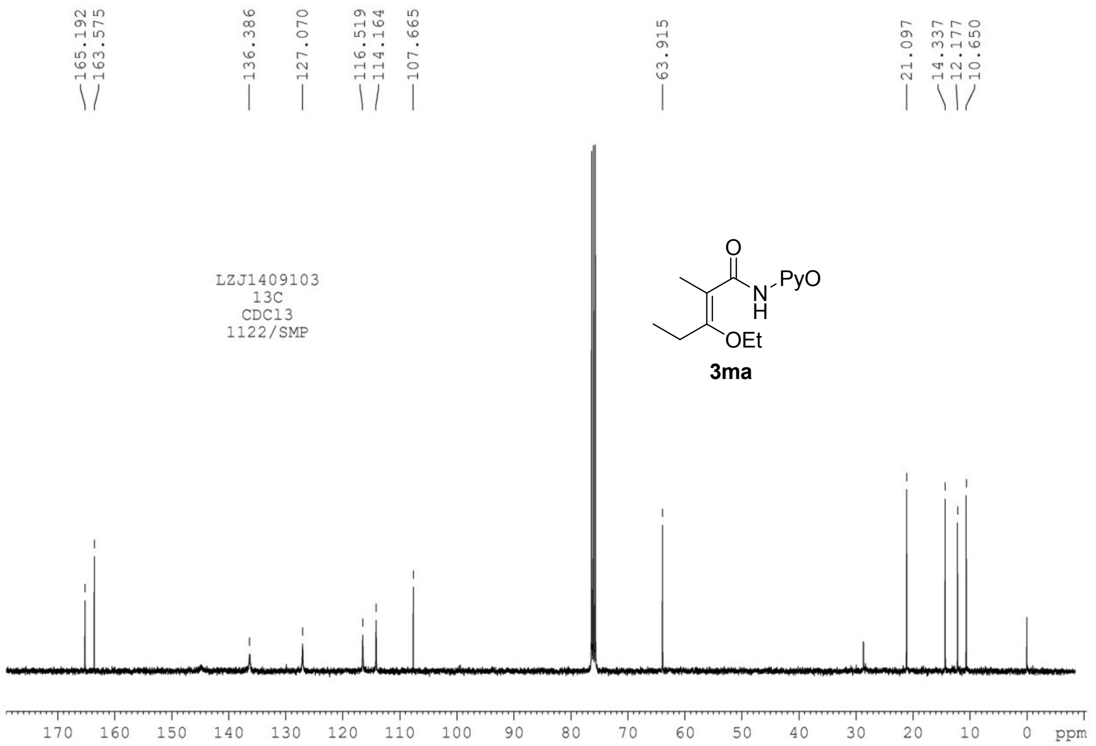
165.192
163.575
136.386
127.070
116.519
114.164
107.665
63.915
21.097
14.337
12.177
10.650
LZJ1409103
13C
CDCl3
1122/SMP
O
N
H
PyO
OEt
3ma
170 160 150 140 130 120 110 100 90 80 70 60 50 40 30 20 10 0 ppm

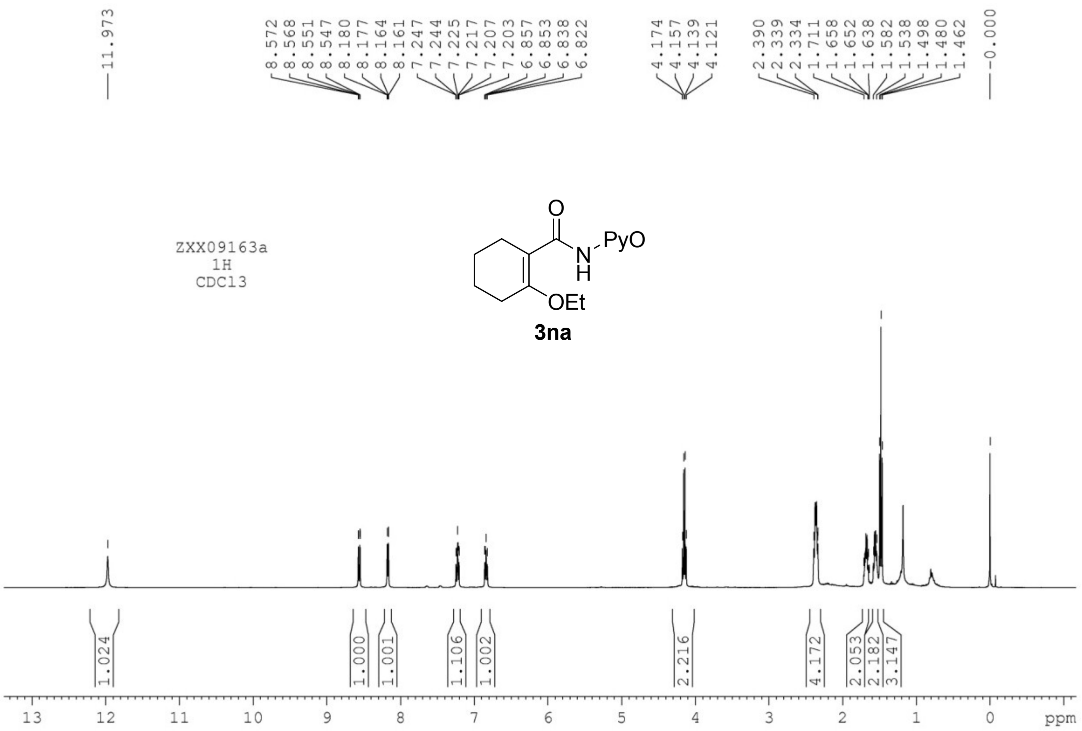
11.973
8.572
8.568
8.551
8.547
8.180
8.177
8.164
8.161
7.247
7.244
7.225
7.217
7.207
7.203
6.857
6.853
6.838
6.822
4.174
4.157
4.139
4.121
2.390
2.339
2.334
1.711
1.658
1.652
1.638
1.582
1.538
1.498
1.480
1.462
-0.000
ZXX09163a
1H
CDCl3
O
N
PyO
H
OEt
3na
1.024
1.000
1.001
1.106
1.002
2.216
4.172
2.053
2.182
3.147
13
12
11
10
9
8
7
6
5
4
3
2
1
0
ppm

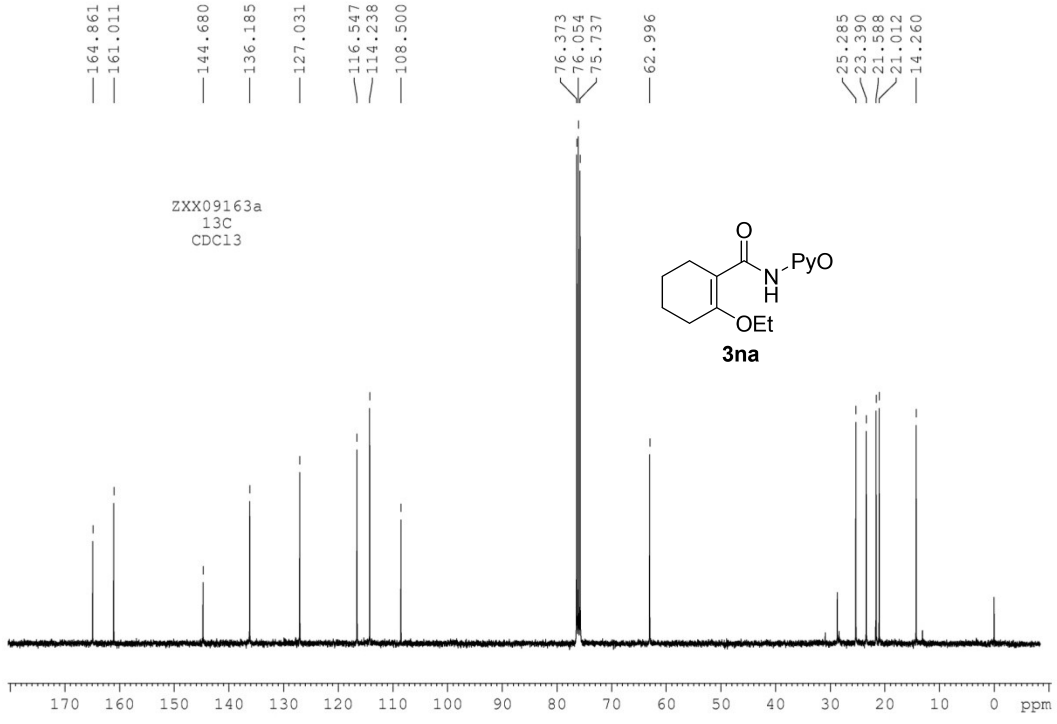
164.861
161.011
144.680
136.185
127.031
116.547
114.238
108.500
76.373
76.054
75.737
62.996
25.285
23.390
21.588
21.012
14.260
ZXX09163a
13C
CDCl3
O
N
PyO
H
OEt
3na
170
160
150
140
130
120
110
100
90
80
70
60
50
40
30
20
10
0
ppm

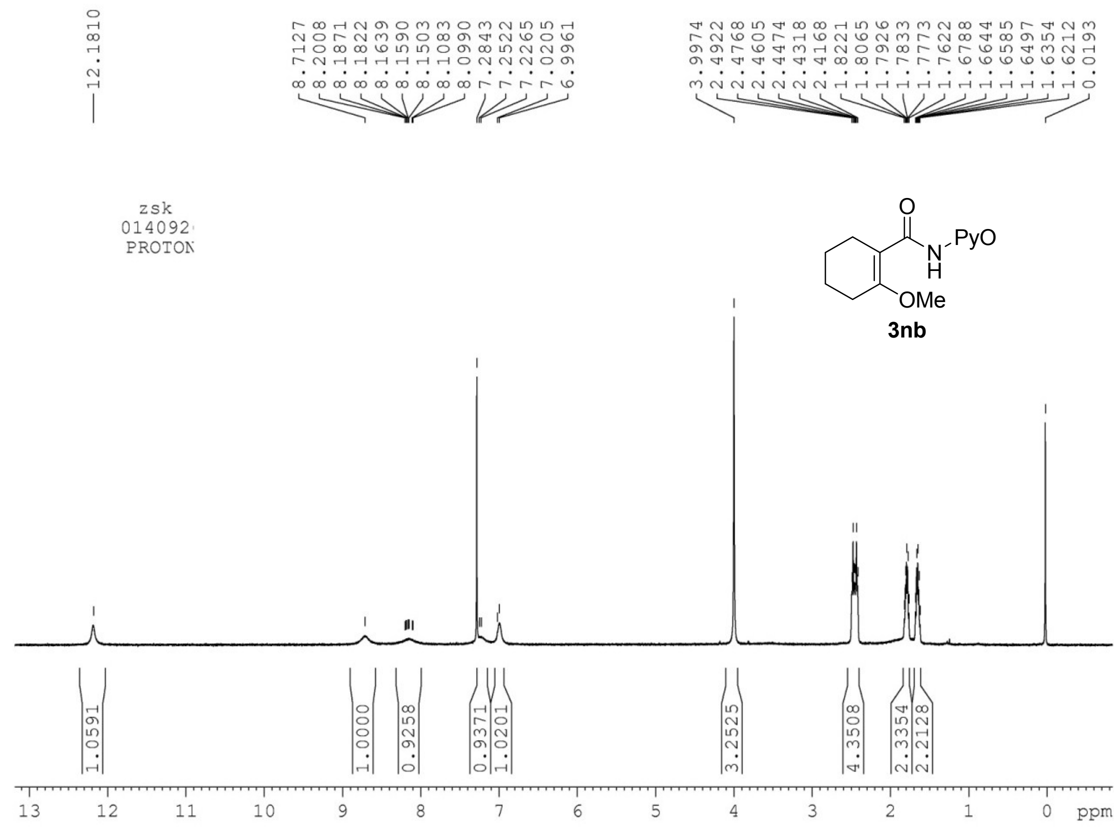
12.1810
8.7127 8.2008 8.1871 8.1822 8.1639 8.1590 8.1503 8.1083 8.0990 7.2843 7.2522 7.2265 7.0205 6.9961
3.9974 2.4922 2.4768 2.4605 2.4474 2.4318 2.4168 1.8221 1.8065 1.7926 1.7833 1.7773 1.7622 1.6788 1.6644 1.6585 1.6497 1.6354 1.6212 0.0193
zsk
PROTON
O
N
H
PyO
OMe
3nb
1.0591 1.0000 0.9258 0.9371 1.0201 3.2525 4.3508 2.3354 2.2128
13 12 11 10 9 8 7 6 5 4 3 2 1 0 ppm

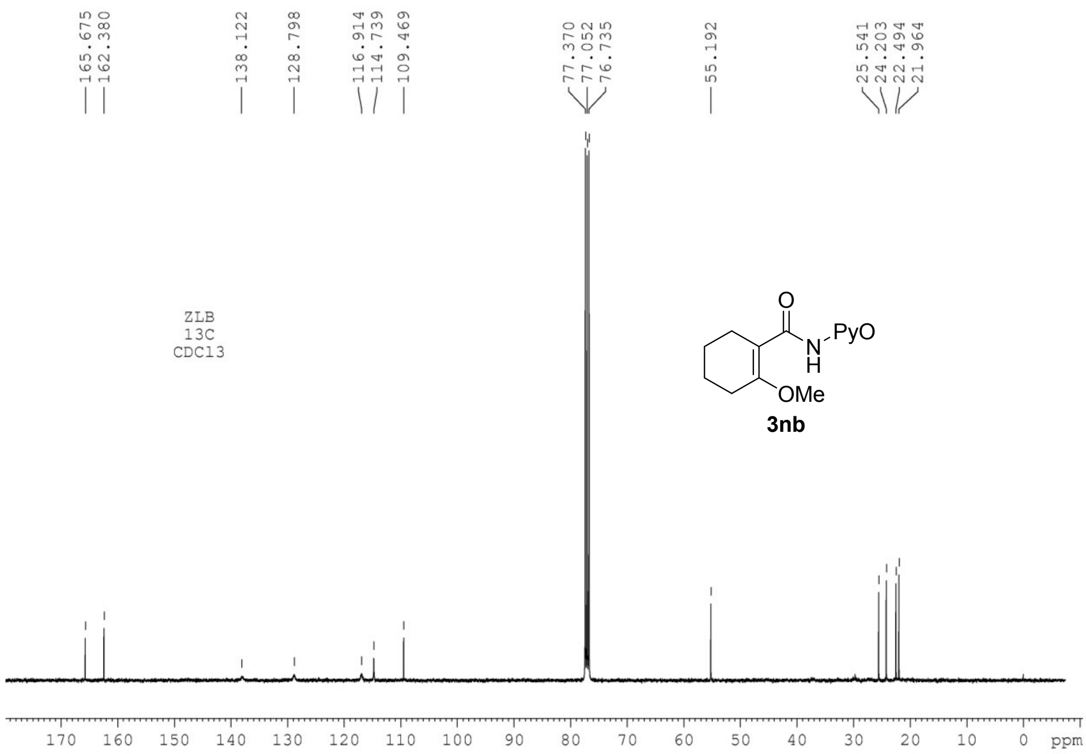
165.675 162.380 138.122 128.798 116.914 114.739 109.469
77.370 77.052 76.735
55.192
25.541 24.203 22.494 21.964
ZLB
13C
CDCl3
O
N
H
PyO
OMe
3nb
170 160 150 140 130 120 110 100 90 80 70 60 50 40 30 20 10 0 ppm

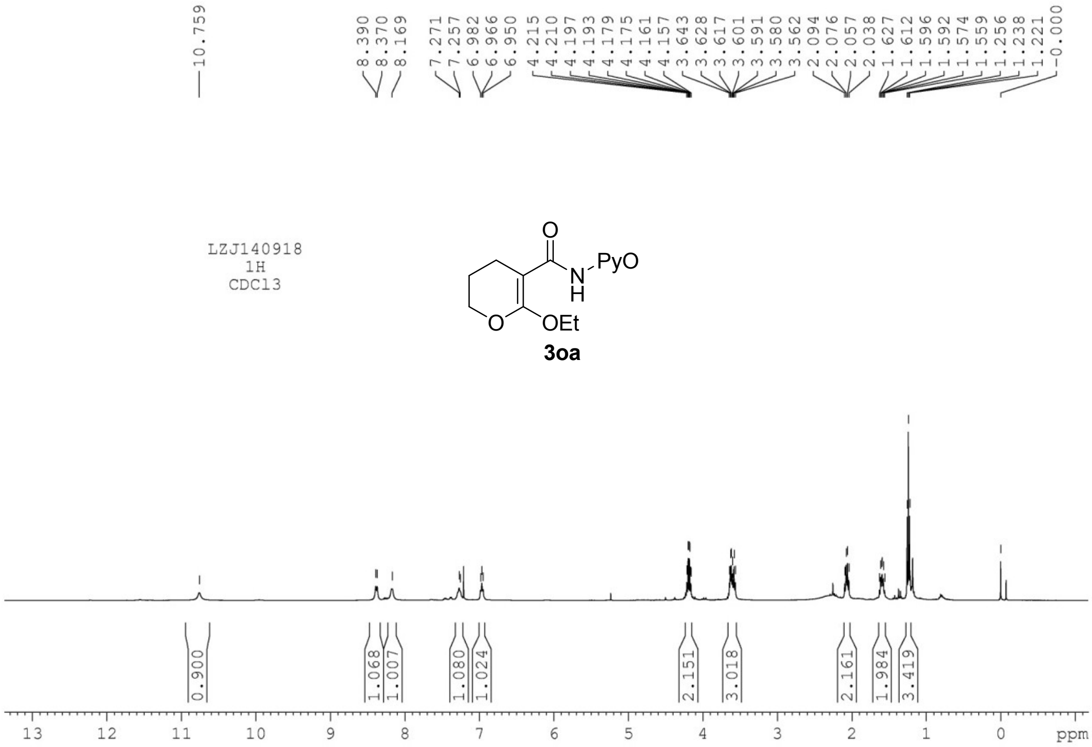
10.759
8.390
8.370
8.169
7.271
7.257
6.982
6.966
6.950
4.215
4.210
4.197
4.193
4.179
4.175
4.161
4.157
3.643
3.628
3.617
3.601
3.591
3.580
3.562
2.094
2.076
2.057
2.038
1.627
1.612
1.596
1.592
1.574
1.559
1.256
1.238
1.221
-0.000
LZJ140918
1H
CDCl3
O
N
H
PyO
O
OEt
3oa
0.900
1.068
1.007
1.080
1.024
2.151
3.018
2.161
1.984
3.419
13
12
11
10
9
8
7
6
5
4
3
2
1
0
ppm

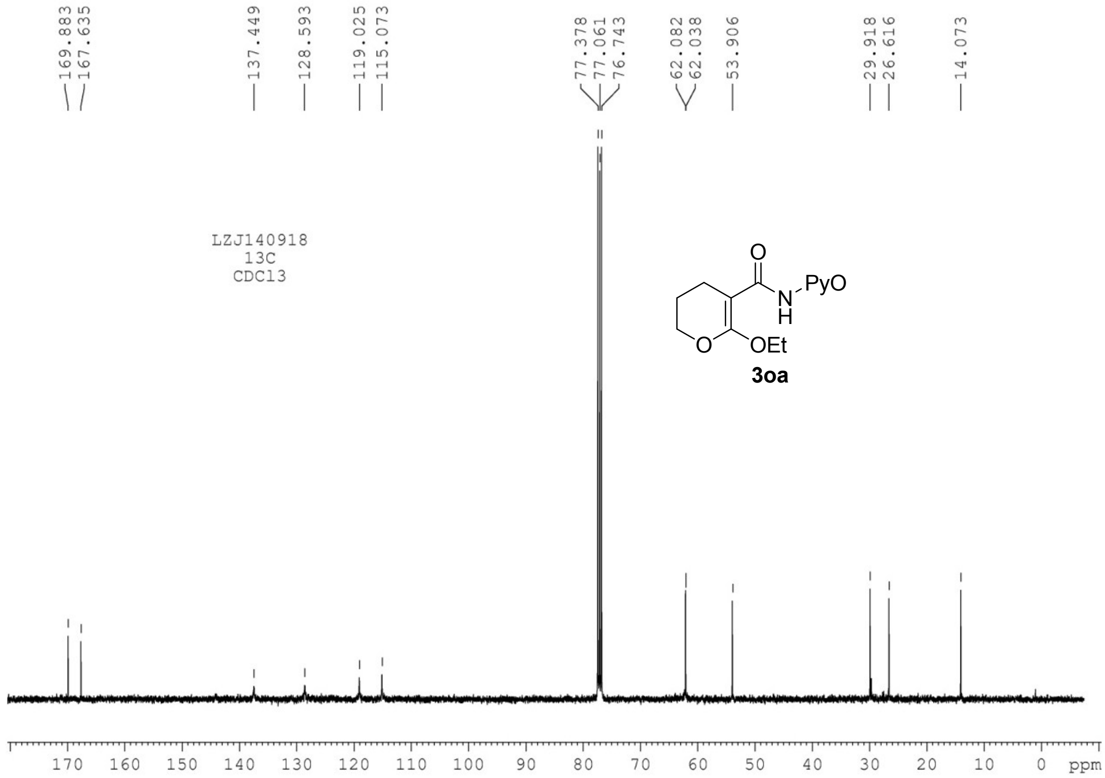
169.883
167.635
137.449
128.593
119.025
115.073
77.378
77.061
76.743
62.082
62.038
53.906
29.918
26.616
14.073
LZJ140918
13C
CDCl3
O
N
H
PyO
O
OEt
3oa
170
160
150
140
130
120
110
100
90
80
70
60
50
40
30
20
10
0
ppm

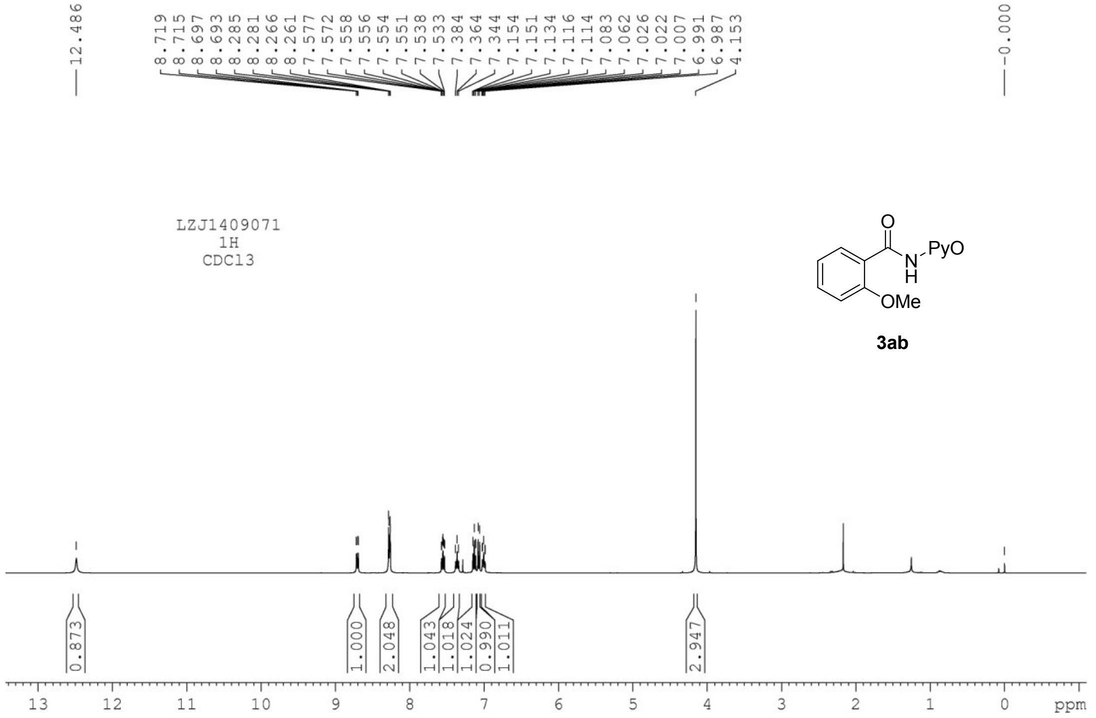
12.486
8.719
8.715
8.697
8.693
8.285
8.281
8.266
8.261
7.577
7.572
7.558
7.556
7.554
7.551
7.538
7.533
7.384
7.364
7.344
7.154
7.151
7.134
7.116
7.114
7.083
7.062
7.026
7.022
7.007
6.991
6.987
4.153
-0.000
LZJ1409071
1H
CDCl3
O
N
PyO
H
OMe
3ab
0.873
1.000
2.048
1.043
1.018
1.024
0.990
1.011
2.947
13 12 11 10 9 8 7 6 5 4 3 2 1 0 ppm

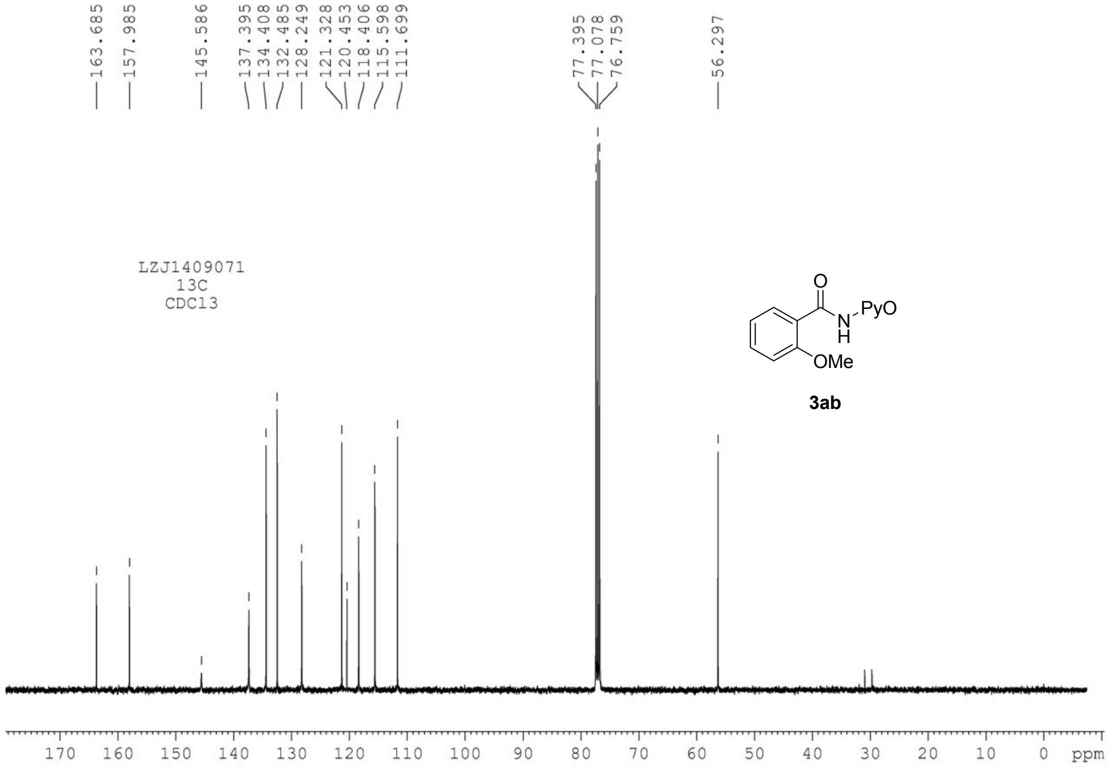
163.685
157.985
145.586
137.395
134.408
132.485
128.249
121.328
120.453
118.406
115.598
111.699
77.395
77.078
76.759
56.297
LZJ1409071
13C
CDCl3
O
N
PyO
H
OMe
3ab
170 160 150 140 130 120 110 100 90 80 70 60 50 40 30 20 10 0 ppm

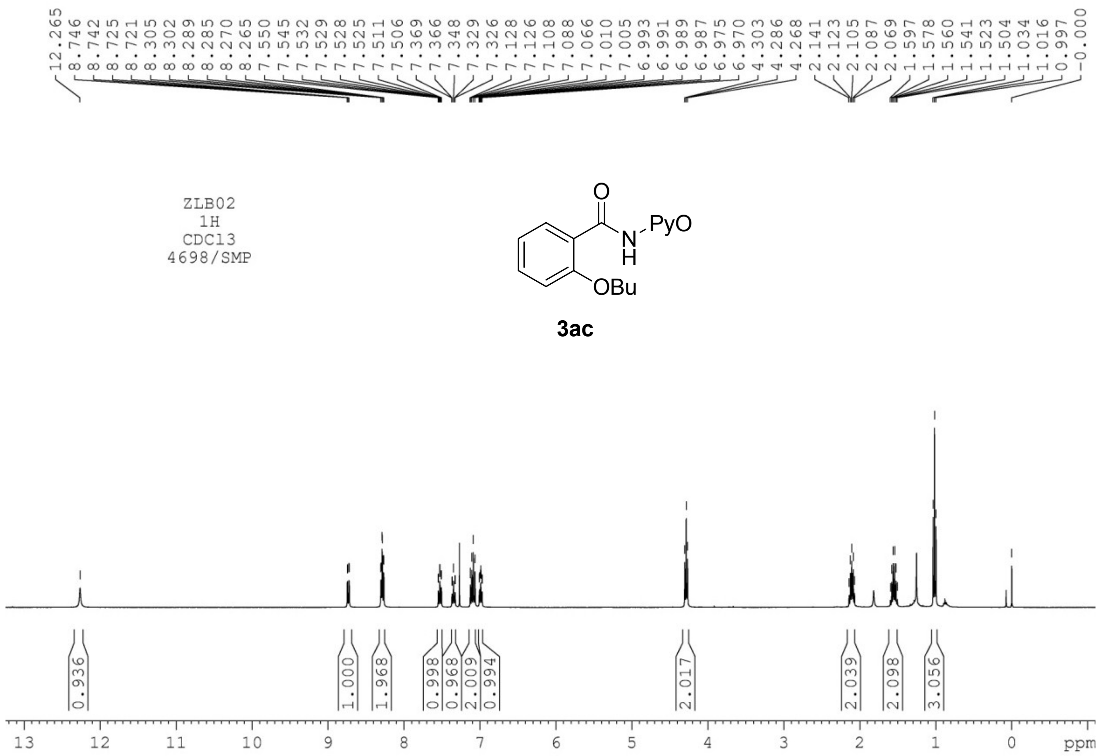
12.265
8.746
8.742
8.725
8.721
8.305
8.302
8.289
8.285
8.270
8.265
7.550
7.545
7.532
7.529
7.528
7.525
7.511
7.506
7.369
7.366
7.348
7.329
7.326
7.128
7.126
7.108
7.088
7.066
7.010
7.005
6.993
6.991
6.989
6.987
6.975
6.970
4.303
4.286
4.268
2.141
2.123
2.105
2.087
2.069
1.597
1.578
1.560
1.541
1.523
1.504
1.034
1.016
0.997
0.000
ZLB02
1H
CDCl3
4698/SMP
O
N
H
PyO
OBu
3ac
0.936
1.000
1.968
0.998
0.968
2.009
0.994
2.017
2.039
2.098
3.056
13
12
11
10
9
8
7
6
5
4
3
2
1
0
ppm

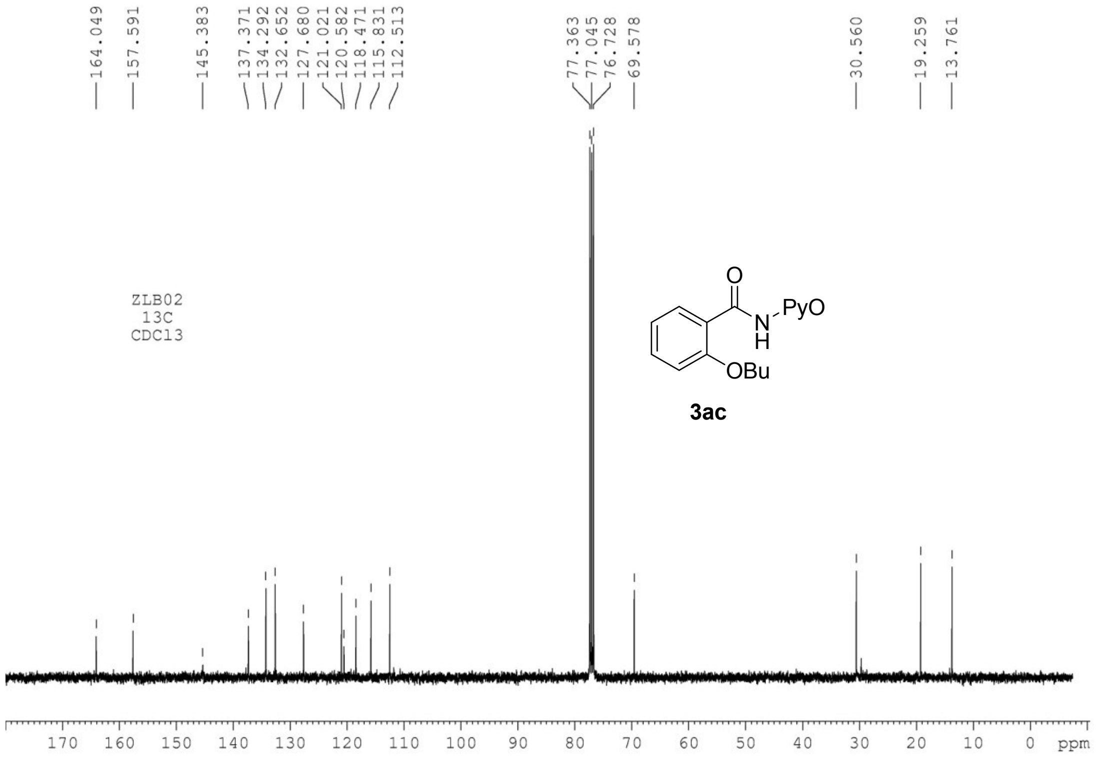
164.049
157.591
145.383
137.371
134.292
132.652
127.680
121.021
120.582
118.471
115.831
112.513
77.363
77.045
76.728
69.578
30.560
19.259
13.761
ZLB02
13C
CDCl3
O
N
H
PyO
OBu
3ac
170
160
150
140
130
120
110
100
90
80
70
60
50
40
30
20
10
0
ppm

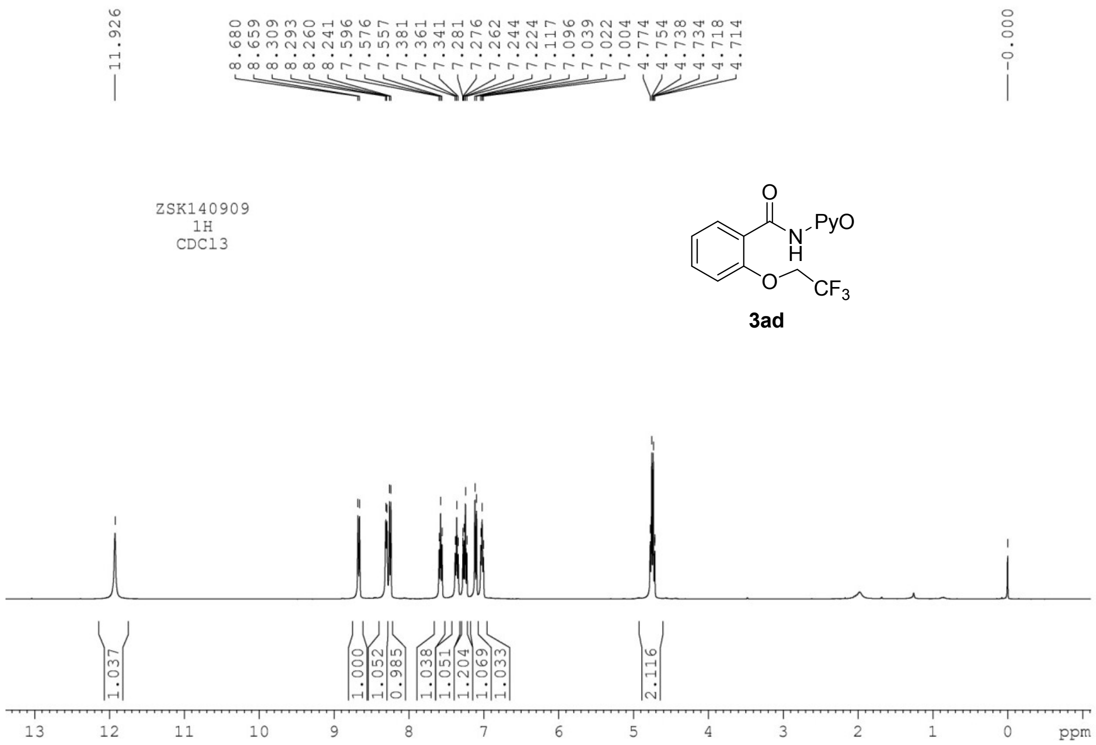
11.926
8.680
8.659
8.309
8.293
8.260
8.241
7.596
7.576
7.557
7.381
7.361
7.341
7.281
7.276
7.262
7.244
7.224
7.117
7.096
7.039
7.022
7.004
4.774
4.754
4.738
4.734
4.718
4.714
0.000
ZSK140909
1H
CDCl3
O
N
H
PyO
O
CF3
3ad
1.037
1.000
1.052
0.985
1.038
1.051
1.204
1.069
1.033
2.116
13
12
11
10
9
8
7
6
5
4
3
2
1
0
ppm

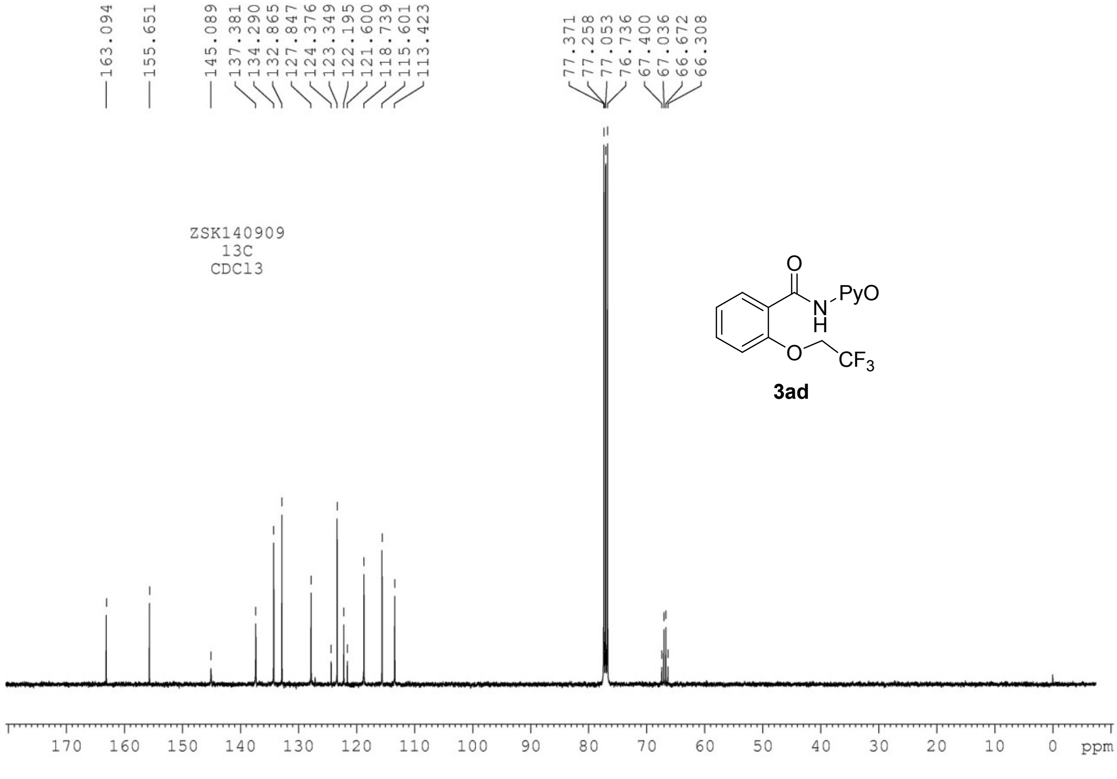
163.094
155.651
145.089
137.381
134.290
132.865
127.847
124.376
123.349
122.195
121.600
118.739
115.601
113.423
77.371
77.258
77.053
76.736
67.400
67.036
66.672
66.308
ZSK140909
13C
CDCl3
O
N
H
PyO
O
CF3
3ad
170
160
150
140
130
120
110
100
90
80
70
60
50
40
30
20
10
0
ppm

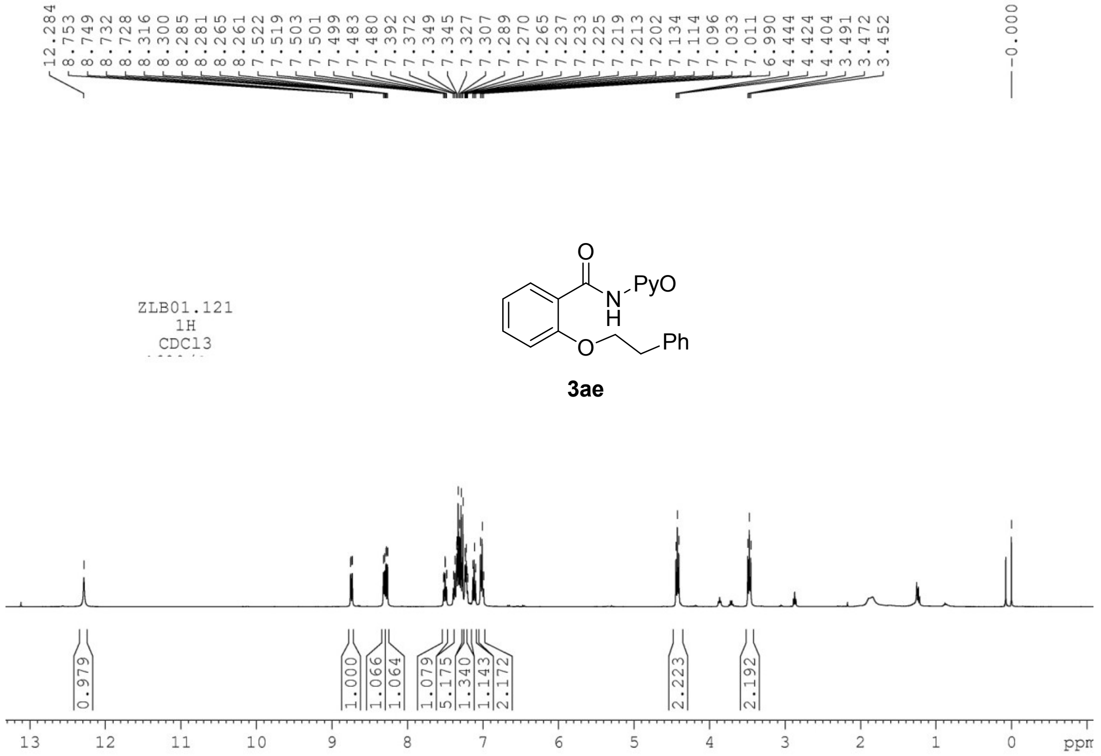
12.284 8.753 8.749 8.732 8.728 8.316 8.300 8.285 8.281 8.265 8.261 7.522 7.519 7.503 7.501 7.499 7.483 7.480 7.392 7.372 7.349 7.345 7.327 7.307 7.289 7.270 7.265 7.237 7.233 7.225 7.219 7.213 7.202 7.134 7.114 7.096 7.033 7.011 6.990 4.444 4.424 4.404 3.491 3.472 3.452
0.000
ZLB01.121
1H
CDCl3
O
N
PyO
H
O
Ph
3ae
0.979
1.000
1.066
1.064
1.079
5.175
1.340
1.143
2.172
2.223
2.192
13 12 11 10 9 8 7 6 5 4 3 2 1 0 ppm

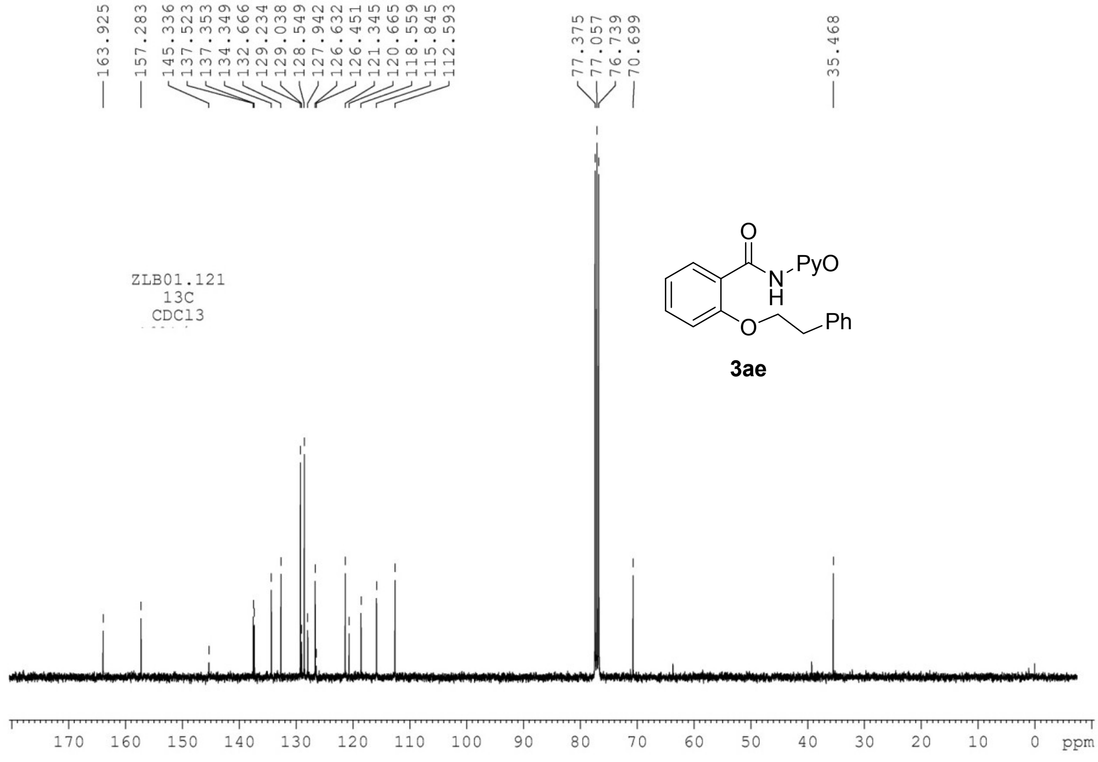
163.925
157.283
145.336 137.523 137.353 134.349 132.666 129.234 129.038 128.549 127.942 126.632 126.451 121.345 120.665 118.559 115.845 112.593
77.375 77.057 76.739 70.699
35.468
ZLB01.121
13C
CDCl3
O
N
PyO
H
O
Ph
3ae
170 160 150 140 130 120 110 100 90 80 70 60 50 40 30 20 10 0 ppm
